# Supplementary material for: Parent, patient and clinician perceptions of outcomes during and following neonatal care: a systematic review of qualitative research
Source: BMJ Paediatr Open. 2018 Oct 9;2(1):e000343. doi: 10.1136/bmjpo-2018-000343 (PMC6203019; doi:10.1136/bmjpo-2018-000343)
Supplement: Supplementary data [file bmjpo-2018-000343supp001.docx]

**Online only supplemental material**

eTable 1: Eligiblity criteria

eFigure 1: Search strategy

eTable 2: Summary of included studies

eTable 3: List of all outcomes

eTable 4: Outcomes within each study

eFigure 2: Results of permutation test analysis regarding gestational age

eFigure 3: Results of permutation test analysis regarding stakeholder group

**eTable 1: Eligibility Criteria**

Sample: Babies of any gestational age at birth receiving care in a neonatal unit.

Phenomenon of Interest: Care on a Neonatal Intensive Care Unit or Special Care Baby Unit. Care provided exclusively on labour or postnatal wards or in an outpatient/community environment was not included.

Design: Qualitative research (including phenomenology, ethnography, case studies and grounded theory) was included. Quantitative research was included if qualitative data had been gathered (e.g. surveys developed with parent or caregiver input).

Evaluation: Data relating to outcomes that former patients, parents or healthcare professionals feel are important.

Research type: Qualitative research or mixed methods research was included. Research that was exclusively quantitative was excluded.

**eFigure 1: Search Strategy**

The following search strategy was utilised to search the Medline database:

1. Medline; neonat*.ti,ab;

2. Medline; exp INFANT, NEWBORN/;

3. Medline; ((preterm OR "pre term" OR prematur*) AND (infan* OR neonat* OR baby OR babies)).ti,ab;

4. Medline; (infant* adj2 premature).af;

5. Medline; exp NEONATOLOGY/;

6. Medline; exp INTENSIVE CARE UNITS, NEONATAL/;

7. Medline; exp INTENSIVE CARE, NEONATAL/;

8. Medline; ("intensive care" adj2 neonat*).ti,ab;

9. Medline; NICU.ti,ab;

10. Medline; SCBU.ti,ab;

11. Medline; "special care baby unit*".ti,ab;

12. Medline; INFANT, PREMATURE, DISEASES/;

13. Medline; 1 OR 2 OR 3 OR 4 OR 5 OR 6 OR 7 OR 8 OR 9 OR 10 OR 11 OR 12;

14. Medline; (treatment adj2 outcome*).ti,ab;

15. Medline; exp TREATMENT OUTCOME/;

16. Medline; "key outcome*".ti,ab;

17. Medline; (important adj2 outcome*).ti,ab;

18. Medline; exp PATIENT OUTCOME ASSESSMENT/;

19. Medline; (outcome* adj2 set).ti,ab;

20. Medline; exp "OUTCOME ASSESSMENT (HEALTH CARE)"/;

21. Medline; "outcome assessment*".ti,ab;

22. Medline; (core adj2 outcome*).ti,ab;

23. Medline; "outcome measure*".ti,ab;

24. Medline; 14 OR 15 OR 16 OR 17 OR 18 OR 19 OR 20 OR 21 OR 22 OR 23;

25. Medline; ("semi-structured" OR semistructured OR unstructured OR informal OR "indepth" OR indepth OR "face-to-face").ti,ab;

26. Medline; ((structured OR guide) adj3 (interview* OR discussion*)).ti,ab;

27. Medline; ("focus group*" OR qualitative OR ethnograph* OR fieldwork OR "field work" OR "key informant*").ti,ab;

28. Medline; exp INTERVIEWS AS TOPIC/;

29. Medline; exp FOCUS GROUPS/;

30. Medline; exp NARRATION/;

31. Medline; exp QUALITATIVE RESEARCH/;

32. Medline; 25 O 26 OR 27 OR 28 OR 29 OR 30 OR 31 OR 32;

33. Medline; 13 AND 24 AND 32;

**eTable 2: Summary Of Included Studies**

| **Source** | **Country** | **Gestational age of neonates involved** | **Birthweight of neonates involved** | **Methodology** | **Stakeholders**  (numbers and further details given in brackets where available) |
| --- | --- | --- | --- | --- | --- |
| Ahern (2013) | Australia | Unspecified | Unspecified | Questionnaire (Delphi); Interviews (individual) | Doctors; Nurses; Others (social worker, clinical educator) |
| Armentrout (2009) | USA | Term; Preterm; Extreme preterm | Not specified | Interviews (individual); Interviews (group) | Parents (11 mothers, 4 fathers) |
| Arruda et al. (2010) | Brazil | Preterm; Extreme preterm | Very low | Interviews (individual); Interviews (group | Parents (8 mothers); Other family members (1 maternal grandmother) |
| Brinchmann (2000) | Norway | Preterm; Extreme preterm | Unspecified | Interviews (unspecified size); Direct observation | Doctors (8); Nurses (10); |
| Brinchmann et al. (2001) | Norway | Unspecified | Unspecified | Interviews (unspecified size); Direct observation | Doctors (8); Nurses (10); Parents (20) |
| Brødsgaard (2015) | Denmark | Preterm; Extreme preterm | Normal; Low; Very low; Extremely low | Questionnaire; Interviews (group) | Parents (15) |
| Callen et al. (2005) | Canada | Preterm; Extreme preterm | Very low; Extremely low | Direct observation | Parents (64 mothers) |
| Catlin (2008) | USA | Term; Extreme preterm | Normal; Low; Very low; Extremely low | Questionnaire | Doctors (42); Nurses (291); Others (4 social workers and 76 unspecified) |
| Cescutti-Butler (2009) | UK | Unspecified | Unspecified | Interviews (unspecified size) | Parents (4 mothers, 1 father) |
| Chang (2014) | USA | Unspecified | Unspecified | Interviews (group) | Doctors; Nurses; Other allied healthcare professionals (occupational therapist); Former neonatal patients (13); Parents (23 mothers, 5 fathers); Other family members (1 grandparent, 2 unspecified); Others (social worker, clinical co-ordinator) |
| Chia (2006) | Australia | Unspecified | Unspecified | Questionnaire; Interviews (unspecified size) | Nurses (34) |
| Chollat (2014) | France | Preterm; Extreme preterm | Low; Very low | Questionnaire | Parents (431) |
| Darcy (2008) | USA | Unspecified | Unspecified | Interviews (unspecified size) | Nurses (6) |
| Docherty (2007) | USA | Term; Preterm | Unspecified | Interviews (individual) | Parents (5 mothers) |
| Ericson (2013) | Sweden | Preterm; Extreme preterm | Low | Questionnaire | Nurses (45) |
| Gaíva (2001) | Brazil | Preterm | Unspecified | Questionnaire | Parents (11 mothers) |
| Garcia (2004) | UK | Term; Preterm | Unspecified | Interview (individual) | Doctors (30) |
| Gontijo (2008) | Brazil | Preterm | Low | Questionnaire; Direct observation | Other allied healthcare professionals (19 unspecified); Parents (13 mothers); |
| Green (2015) | Australia | Extreme preterm | Unspecified | Questionnaire; Interviews (individual); Interviews (group) | Nurses (414) |
| Gregory (2012) | USA and Canada | Preterm | Unspecified | Questionnaire | Nurses (70) |
| Griffin (1997) | USA | Preterm; Extreme preterm | Unspecified | Interviews (unspecified size) | Parents (6 mothers, 2 fathers); Others (5 parents of infants not admitted to NICU) |
| Hanrahan (2007) | USA | Unspecified | Unspecified | Interviews (group) | Doctors (12); Nurses (11); Parents (10 mothers, 2 fathers) |
| Hefferman (1999) | USA | Extreme Preterm | Unspecified | Questionnaire | Doctors (3); Nurses (27); Other allied healthcare professionals (2 nurse managers, 1 respiratory care supervisor) |
| Hentschel (2006) | Germany | Term; Preterm | Unspecified | Direct observation | Doctors; Nurses; Parents (31) |
| Holditch-Davis (2003) | USA | Preterm | Normal; Low; Very low | Interviews (unspecified size) | Parents (30 mothers) |
| Hussey-Gardner (1998) | USA | Preterm | Very low | Interviews (unspecified size); Direct observation; Other (artefact collection) | Parents (7 mothers, 4 fathers); Other family members (3 grandmothers) |
| Jirapaet (2006) | Thailand | Unspecified | Unspecified | Interviews (individual) | Nurses (27) |
| Johnson (2007) | USA | Preterm | Very low | Interviews (unspecified size) | Parents (18 mothers) |
| Jones (2015) | Australia | Preterm; Extreme preterm | Normal; Low; Very low; Extremely low | Interviews (individual); Interviews (group) | Nurses (12); Parents (27 mothers, 5 fathers) |
| Kavanaugh (1997) | USA | Unspecified | Extremely low | Interviews (individual) | Parents (5 mothers, 3 fathers) |
| Koh (2007) | Australia | Preterm | Low | Questionnaire; Interviews (individual) | Parents (200 mothers) |
| Leavy (2015) | Argentina | Preterm | Very low | Interviews (group) | Former neonatal patients (59) |
| Lee (2005) | USA | Preterm | Unspecified | Questionnaire; Interviews (unspecified) | Parents (30 mothers, 25 fathers) |
| Lou (2009) | Denmark | Extreme preterm | Very low | Interviews (individual) | Parents (11 mothers, 9 fathers) |
| Malhotra (2015) | USA | Unspecified | Very low | Interviews (unspecified size); Direct observation | Doctors (10); Nurses (16) |
| Miljeteig (2006) | India | Term; Preterm | Unspecified | Interviews (individual); Direct observation | Doctors (14); Nurses (8) |
| Miracle (2004) | USA | Preterm; Extreme preterm | Very low; Extremely low | Interviews (individual) | Parents (21 mothers) |
| Moyer (2010) | USA | Unspecified | Unspecified | Interviews (unspecified size); Direct observation; Other (Health care failure mode and effect analysis) | Doctors; Nurses; Parent (1); Others (discharge planners, social worker) |
| Orfali (2004) | USA | Preterm; Extreme preterm | Low; Very low | Interviews; Direct observation | Doctors (60); Parents (71) |
| Rasmussen (2016) | Canada | Unspecified | Unspecified | Interviews (individual) | Doctors (12) |
| Reyna (2006) | USA | Preterm | Unspecified | Interview (size unspecified) | Parents (27 mothers) |
| Rohsiswatmo (2014) | Indonesia | Unspecified | Low; Very low | Questionnaire; Interviews (group); Observation; Other (operational research) | Other allied healthcare professionals (unspecified) |
| Saigal (1999) | Canada | Unspecified | Normal; Extremely low | Questionnaire; Interviews (individual) | Doctors (100); Nurses (103); Former neonatal patients (264); Parents (275) |
| Saunders (2003) | USA | Unspecified | Very low | Interviews (group); Other (Internal process analysis, Collaborative learning) | Doctors; Nurses; Other allied healthcare professionals (respiratory therapists, pharmacists, physical and occupational therapists) Parents (76); Others (unit secretaries, social workers) |
| Sawyer (2014) | UK | Preterm | Unspecified | Questionnaire; Interviews (individual); Interviews (group) | Parents (145 mothers, 85 fathers) |
| Schneiderman (1997) | USA | Unspecified | Unspecified | Interviews (individual) | Other allied healthcare professionals (22 Emergency department directors, 43 NICU directors, 43 Ethics committee chairpeople) |
| Sharek (2003) | USA | Unspecified | Low; Very low; Extremely low | Interviews (group) | Other allied healthcare professionals (unspecified) |
| Short (2003) | USA | Term; Preterm; Extreme preterm | Normal; Very low | Questionnaire; Interviews (unspecified size) | Parents; Other (teachers) |
| Sisk (2010) | USA | Preterm; Extreme preterm | Low; Very low; Extremely low | Interviews (individual) | Parents (32 mothers) |
| Smith (2006) | USA | Preterm; Extreme preterm | Unspecified | Interviews (individual) | Parents (16 fathers) |
| Snowdon (1997) | UK | Term | Unspecified | Interviews (unspecified size) | Parents (21 mothers, 16 fathers) |
| Snowdon (2014) | UK | Term; Preterm; Extreme preterm | Normal; Low; Very low; Extremely low | Interviews (individual) | Other allied healthcare professionals (51 trial team members); Parents (51) |
| Squitieri (2013) | USA | Unspecified | Unspecified | Questionnaire; Interviews (individual) | Former neonatal patients (18); Parents (18) |
| Stevens (2014) | USA | Preterm; Extreme preterm | Low; Very low; Extremely low | Interviews (individual) | Parents (12 mothers, 8 fathers) |
| Thoren (2013) | Germany | Preterm | Unspecified | Other (Evaluation of Facebook groups and discussion content) | Parents; Other (representatives of online groups) |
| Thoyre (2000) | USA | Unspecified | Very low | Interviews (individual) | Parents (22 mothers) |
| Turrill (2003) | UK | Unspecified | Unspecified | Interviews (unspecified size) | Nurses (6); Other allied healthcare professionals (1 clinical services manager) |
| Turrill (2003) | UK | Unspecified | Unspecified | Interviews (unspecified size); Direct observation | Nurses |
| van Zuuren (2006) | Netherlands | Unspecified | Unspecified | Interviews (individual); Direct observation | Doctors (5); Nurses (6); Others (1 social worker, 1 pastoral care worker) |
| Vandenbussche (1999) | Netherlands | Unspecified | Unspecified | Questionnaire | Doctors (12); Parents (30 mothers) |
| Votteler (2005) | USA | Unspecified | Unspecified | Questionnaire; Interviews (individual) | Former neonatal patients (14); Parents; Other family members |
| Wielenga (2015) | Netherlands | Unspecified | Unspecified | Questionnaire (Delphi) | Nurses; Other allied healthcare professionals (75) |

**eTable 3: Outcomes Identified**

| **Outcome Domain Category** | **Outcome Domain** | **Outcome** |
| --- | --- | --- |
| Organ system | Respiratory | Asthma |
|  |  | Breathlessness |
|  |  | Chronic Lung Disease |
|  |  | Excessive secretions |
|  |  | Frequent respiratory illnesses |
|  |  | Mechanical ventilation |
|  |  | Nasal congestion |
|  |  | Oxygen dependence |
|  |  | Pneumothorax |
|  | Cardiovascular | Hypotension |
|  |  | Presence of Patent Ductus Arteriosus |
|  | Gastrointestinal | Breastfeeding |
|  |  | Choice of milk for feeding |
|  |  | Choking during feeding |
|  |  | Eating disorder |
|  |  | Feeding difficulties |
|  |  | Feeding practices |
|  |  | Fistulas |
|  |  | Frequency of defaecation |
|  |  | Initiating enteral feeds |
|  |  | Liver failure |
|  |  | Necrotising enterocolitis |
|  |  | Nutritional intake |
|  |  | Oral feeding |
|  |  | Other gastrointestinal malformations |
|  |  | Regurgitation |
|  |  | Short gut syndrome |
|  | Neurological | Brain damage (not further specified) |
|  |  | Neurological care |
|  |  | Neurological symptoms |
|  |  | Seizures |
|  |  | Significant IVH |
|  |  | Sleep disorders |
|  | Genitourinary | Urological disorders |
|  | Infection | Prevention of infection |
|  |  | Sepsis |
|  |  | Susceptibility to infection |
|  | Skin | Appearance of scars |
|  |  | Burns |
|  |  | Extravasation injuries |
|  |  | Pressure sores |
|  |  | Skin care |
|  | Surgical | Appearance of scars |
|  |  | Care for surgical babies |
|  |  | Need for ileostomy |
|  |  | Need for multiple operations |
|  | Developmental | Ability to feed themselves |
|  |  | Ability to undertake sport |
|  |  | Ability to walk |
|  |  | Difficulties with activities of daily living |
|  |  | Hearing impairment |
|  |  | Issues of development and motor skills |
|  |  | Language disorders |
|  |  | Need for physical therapy |
|  |  | Normal hearing |
|  |  | Retinopathy of prematurity |
|  |  | Social difficulties |
|  |  | Visual impairment |
| Holistic | Normality | Ability to lead a normal life |
|  |  | Being treated normally |
|  |  | Inability to create a normal life |
|  |  | Normal health |
|  |  | Normality |
|  |  | Thriving |
|  | Suffering | Comfort |
|  |  | Ex-patients separation from their suffering |
|  |  | Symptom control |
|  |  | Suffering |
|  | Survival | Survival |
|  |  | Survival with disability |
|  |  | Survival without disability |
|  | Pain | Chronic pain |
|  |  | Pain |
|  |  | Pain management |
|  | Growth | Growth |
|  | Other holistic outcomes | Overall Health State |
|  |  | Physical appearance |
|  |  | Physiological stability |
|  |  | Vitality |
| Parent focussed | Parental support | Balancing caring for themselves and their baby |
|  |  | Barriers to parental involvement |
|  |  | Care provided close to home |
|  |  | Coping with maternal illness |
|  |  | Culture differences |
|  |  | Maintaining hope |
|  |  | Online support |
|  |  | Parental ability to work |
|  |  | Parental competence |
|  |  | Parental involvement |
|  |  | Preparation for NICU admission |
|  |  | Support from faith |
|  |  | Support from family and friends |
|  |  | Support from fathers |
|  |  | Support from healthcare professionals |
|  | Other parent focussed outcomes | Long term effects on parents |
|  |  | Parental perception of uncertainty |
|  |  | Support beyond NICU |
| Healthcare delivery | Healthcare workers-Communication | Allowing time for conversation |
|  |  | Awareness of parental views |
|  |  | Candour |
|  |  | Communicating in challenging settings |
|  |  | Communicating information effectively |
|  |  | Communication about discharge |
|  |  | Communication with ex-neonatal patients |
|  |  | Communication with parents |
|  |  | Developing a caring relationship |
|  |  | Keeping parents informed |
|  |  | Language barrier |
|  |  | Treating parents with respect |
|  |  | Using aids to communication |
|  | Healthcare workers-Knowledge and Competence | Consistency of decisions |
|  |  | Ethical decision making |
|  |  | Expertise in palliative care |
|  |  | Healthcare professional competence |
|  |  | Healthcare professionals behaviour |
|  |  | Identifying who is responsible for care |
|  |  | Medical errors |
|  |  | Staff insecurity |
|  |  | Staffing levels |
|  |  | Iatrogenic harm |
|  | Other healthcare delivery outcomes | Inclusion in research |
| Economic | Healthcare utilisation | Frequent appointments |
|  |  | Frequent readmissions |
|  |  | Inappropriate treatments |
|  |  | Need for frequent treatments |
|  |  | Need for lifelong care |
|  |  | Recurrent sickness |
|  | Other economic outcomes | Duration of admission |
|  |  | Healthcare resources |
| Social | Psychiatric | Autism |
|  |  | Behavioural disturbances |
|  |  | Dyslexia |
|  |  | Mood disorders |
|  |  | Need for educational support |
|  |  | Psychiatric disorder |
|  | Relationships with others | Bonding with family and friends |
|  |  | Bonding with parents |
|  |  | Childhood happiness |
|  |  | Effects on family and friends |
|  |  | Family resources |
|  |  | Overprotective parent-child relationship |
|  |  | Peer acceptance |
|  |  | Psychological coping |
|  | Other social outcomes | Schooling |
|  |  | Self-identifying as premature |

**eTable 4: Outcomes Within Each Study**

| **Source** | **Outcome Domains and outcomes** |
| --- | --- |
| Ahern (2013) | Respiratory Breathlessness: *Education priorities: Managing symptoms (eg, secretions, breathlessness) in a dying baby*  Excessive secretions: *Education priorities: Managing symptoms (eg, secretions, breathlessness) in a dying baby* Gastrointestinal Feeding Difficulties: *Education priorities: Managing feeding in a dying baby* Parental Support Support from Healthcare Professionals: *Educational priorities identified included: How to provide emotional support to grieving parents Helping parents say "goodbye" Supporting parents when they are at different stages of grief Identifying and supporting families at high risk of complicated grief* Healthcare workers-Communication Communicating in challenging settings: *Education priorities: Communicating with parents of palliative neonates Educational priorities identified included: Knowing what to say; using the right words Communicating with parents about the palliative care plan Initiating difficult conversations* Pain Pain: *Educational priorities identified included: Assessing and managing pain in a dying baby* Suffering Symptom control: *Managing symptoms Educational priorities identified included: Managing symptoms (eg, secretions, breathlessness) in a dying baby* |
| Armentrout (2009) | Development-Special Senses Normal hearing:*"She got to hear her dad sing,"* Parental Support Support from healthcare professionals:*"the nursing staff, the doctors...they really know what they're doing...not only medically, but dealing with us personally...that helped a lot."* Healthcare workers-Communication Developing a caring relationship: *"such a great blessing...that there was no hindrance in the terms of any kind of an attitude or anything with the nurse or the attending doctor that made us feel uncomfort-able or gave us any additional grief."*  *"the nursing staff, the doctors they really know what they're doing not only medically, but dealing with us personally that helped a lot."* Healthcare workers-Knowledge and Competence Healthcare professional competence: *"the nursing staff, the doctors they really know what they're doing not only medically, but dealing with us personally that helped a lot."* Relationships with others Bonding with parents: *"She got to hear her dad sing, she got to...she knew we were holding her"*  *"my feeling is, if your child has to die, what better place than in mom's arms"*  Bonding with family and friends: *"The only thing we might have done...some of our closest friends...it would have been nice to have them there as well...none of our friends got to see or meet him while he was alive."*  *"I wanted them to spend time with her, hold her, and bathe her and do whatever" being able to have their infant with them as part of a family was something that all parents proclaimed they "would never replace for anything in the world."*  Effects on family and friends: *"I guess I feel like I lost about a year of my son's time. He turned 3 shortly after my daughter died, and it wasn't until he was a little after 4 that I turned around in the kitchen and saw this little boy and said "Who are you?" I wasn't all there for about a year and this little boy, even though he was the one who got me through it I really didn't know who he was."* Suffering Suffering: *"He was just suffering, and we didn't want him to suffer." "not withdrawing support would be... "some-thing to satisfy my empty arms."* Normality Normality: *"Personally my only regret was that I didn't bathe him" "And the baptism...why didn't we do it sooner...why did we wait for somebody on call to show up with a bottle of sterile water and a cotton ball...why didn't we do it the right way?"*  *"I think it's important to allow yourself to be a mother before you let go...of the baby...to be a parent to some degree...to change their diaper...feed 'em if you can...play with their toes and fingers and nose and...just be a mother to 'em...be a parent to 'em...before you turn it off, because that...is something that you will cherish for the rest of your life."*  *For some it was their only opportunity to hold and talk to their infant like "a normal baby without all the tubes and wires."* Other Support beyond NICU: *"the medical professionals who have become your support system and your family while your child was alive...all of a sudden they're gone...I think it's important to have some kind of support system or information...otherwise it's like stepping into this huge abyss."*  Long term effects on parents: *"would get up in the morning I would be normal after about 2 weeks, 3 weeks act normally take the little one to school, stay in the car for a while come back home and get into bed for the next 6 hours and cry,"*  *"The milestone of a year was really bad. And it is still bad. I mean I'm functioning. I put on make-up. I get dressed. I go out. I look normal but it lasts for a long time the bad thing."* |
| Arruda et al. (2010) | Respiratory Frequent respiratory illnesses: *all concerned and involved with numerous hospitalizations resulting from respiratory failure "He had a lot of pneumonia up to two years, was direct in hospital"* Need for supplemental oxygen: *"Even the three years he was in direct oxygen" Need for ongoing respiratory therapy "She has long respiratory therapy"* Neurological Seizures: *"I did not know he could have seizures. He started to drool, the twitch of the hand fingers, vomited, turned the eye and everything"*  Brain damage (not further specified): *"In tomography and magnetic resonance given spot in the brain. According to the doctor, these spots were the cardiac arrest he had in the NICU, the lack of oxygen." "When I took the pediatrician, she suspected hydrocephalus because the head circumference is growing very fast. Hence, the neuro said no, that his brain had suffered a lot, but would recover."*  Sleep disorder: *They had a lot of irritability, change in posture, sleep, among others*  Development-Gross motor Ability to walk *"He did not walk as a baby, just dragged." "He walked four, my mother never forgot, she says it was a miracle of God"* Development-Special senses Visual impairment *"The first thing I noticed at two years was he, all he would see closer eye toy [...] was all close, there came the glasses"*  *"I took the ophthalmologist with less than a year, he wears glasses since baby"*  *"We discovered his problem because one day was passing a plane and we show and he did not see"*  Development-Speech and Social Social difficulties: *"It does not seem that he is seven years old. So I think so, the teacher says he wants to play under the table, his actions are different, in front of people. People treat different, it feels different"*  Language disorders: *"how she learned to give kiss? He began to speak phonemes? Everything was the stimulus that we gave"* Parental Support Parental competence: *"In the first months after he left the ICU, everything was already appearing: it was too much crying, irritation, they were not normal, as I had no knowledge ... I did not know and then went back to seek this knowledge"* Healthcare utilisation Frequent  readmissions: *all concerned and involved with numerous hospitalizations resulting from respiratory failure* Psychiatric Behavioural disturbances: *They had a lot of irritability, change in posture, sleep, among others "because she did not behave like another child of her age, and I had three children, knew there was nothing right ... The difference was crying, irritation were not normal."*  *"Because she did not act like a child of her age, because beyond being premature, I did not deal with it in the crying, agitation."*  Need for educational support: *"Then I went behind the neuro pediatrician in Maringa, then came the diagnosis of hydrocephalus, motor problem ... and you'll have to put your child in a special school ."* Relationships with others Effects on family and friends: *" One day he fell ill super the other day and did not improve, went to the doctor and she said it was convulsing, I had never seen him so ... but one thing is that my mother did not let her take medicine because still believed in God, then I do not know if that's why "* Normality Normality: *"Then, after two years, he took more health ... no one says that was born premature."* Other Physical appearance: *"all World wanted to see, because he was too small" "My husband's aunt came to say that she was like lizard, I was very sad"* |
| Brinchmann (2000) | Other Vitality: *"All the pictures, X-rays, ultrasounds and test results indicated that this was an infant we were just keeping alive. But he himself showed signs that he was doing all right, and so things did not hang together."*  *"Putting a hand over his eyes was a sign of surplus resources for him" "The doctor said that, whatever we do, however good we are, it is mostly up to the infant himself. No matter how small they are, they can have something within them-selves."* |
| Brinchmann et al. (2001) | Healthcare workers-Knowledge and Competence Healthcare professional competence: *The health professionals said that the ability to sense whether an infant showed vitality came with experience.* Suffering Comfort: *"You can almost feel what it’s like in the incubator, lying on the lambskin, that it's how I would want to have laid and . . . Well, it looks very comfortable"* Other Vitality: *"They had understood that the baby was going to be small, but they had no idea how tiny he was, could not say in advance, 350g. Then the nurse who first took him in her hands after he came out said that he moved his arms, his feet, his body, and cried out, and showed a lot of strength, the moment he was born. She said she believed he could make it because he showed such vitality"*  *"There was more of a spark in her, they meant that she had more spark and seemed stronger. She had more go"* |
| Brødsgaard (2015) | Gastrointestinal Breastfeeding: *"I fully breastfed for four months-100%-and I am so proud of it."* Development-Gross motor  Issues of development and motor skills: *"Issues of development and motor skills, we both looked at my child, research, experience and what I could expect."* Development-Fine motor Issues of development and motor skills:  *"Issues of development and motor skills, we both looked at my child, research, experience and what I could expect."* Parental Support Parental competence: *"We learned everything we needed and knew what we had to do, I was quite comfortable when we went home." "Although initially it was scary, I developed the skills, it was a natural progression; we learned how to take care of him."* Healthcare workers-Communication Treating parents with respect: *"In NICU we are on the sidelines, at home we have the main role and you come and advise, [we can] clearly feel the difference."*  Allowing time for conversation: *"There was so much information, to take in before we could . . . know whether it [EDP] suited us. There was plenty of time to consider it really thoroughly and ask questions." "Felt very much that there was space and time,"* Relationships with others  Bonding with family and friends: *Only when at home on EDP did they feel like a proper family. "I felt divided, torn into two pieces, which were only assembled once I got home."*  *In particular, families with older siblings were very enthusiastic about EDP as it had a significant positive impact on siblings' involvement and participation.* |
| Callen et al. (2005) | Gastrointestinal Breastfeeding: *Mother experiencing difficulty assisting infant to latch and position correctly Mother reported having a low milk volume* Parental Support Coping with maternal illness: *Maternally Reported Barriers to Establishing and Maintaining Breastfeeding: Mother is on medications, has poor nutrition, infected incision, high blood pressure, cold, influenza, spinal headache Mother is exhausted, tired, fatigued Mother lacks confidence, is worried, anxious, stressed, depressed, frustrated, guilty* |
| Catlin (2008) | Respiratory Mechanical ventilation: *'This theme regarding ‘Pathophysiology’ described the inability of the children to breathe or eat independently. Over 30% of all infant descriptions were about babies who had tracheostomies and were unable to be weaned off a ventilator.'* Gastrointestinal Feeding difficulties: *This theme regarding ‘Pathophysiology’ described the inability of the children to breathe or eat independently....Thirty two percent of the anecdotal reports related to inability of the infants to eat, suck, swallow or absorb nutrition. Respondents to the survey reported nutrition categories as the most frequent reason for infants to remain hospitalized.*  Fistulas:  *Respondents to the survey reported nutrition categories as the most frequent reason for infants to remain hospitalized. One-third of all the children had a digestive issue, such as trachea-related malacias, fistulas, malformations, gut related perforations, absences, short gut and liver failures.'* Liver failure: *Respondents to the survey reported nutrition categories as the most frequent reason for infants to remain hospitalized. One-third of all the children had a digestive issue, such as trachea-related malacias, fistulas, malformations, gut related perforations, absences, short gut and liver failures.'* Other gastrointestinal malformations: *Respondents to the survey reported nutrition categories as the most frequent reason for infants to remain hospitalized. One-third of all the children had a digestive issue, such as trachea-related malacias, fistulas, malformations, gut related perforations, absences, short gut and liver failures.'* Short gut syndrome: *Respondents to the survey reported nutrition categories as the most frequent reason for infants to remain hospitalized. One-third of all the children had a digestive issue, such as trachea-related malacias, fistulas, malformations, gut related perforations, absences, short gut and liver failures.'* Development-Gross motor Issues of development and motor skills: *Respondents were concerned that children were not being taught to sit, crawl or talk as would occur outside of the intensive care unit. When child life personnel were available, they were described as very helpful. They would bring in developmentally appropriate toys and teach the staff how to care for ‘growing, 30 pound, NICU babies.’* Development-Speech and Social Language disorders: *Respondents were concerned that children were not being taught to sit, crawl or talk as would occur outside of the intensive care unit.* Parental Support Parental involvement: *"The little boy never left the hospital because his mother was young and single. She had no one to help with the care of this very sick boy. He ended up being in the unit 5 months longer than necessary because we had to get medical foster care to take care of him."* Healthcare workers-Communication Developing a caring relationship: *As the providers gave support to families, families also were described as supporting the staff....It was also difficult for the nurses and physicians to separate from the infants and families after a long period.*  Healthcare workers-Knowledge and Competence Identifying who is responsible for care: *Respondents described an absence of review or oversight of infants with extremely long hospitalizations. Just who was supposed to make decisions about children who were extremely and chronically ill? Was there ever an end point? What was the end point? Can interventions once started ever to be stopped? Who was to determine that the burden on the child was now greater than the benefit? .*  Expertise in palliative care: *Nurses and doctors talked about being able to clearly recognize that a child was going to eventually die, and the used words such as ‘torturing’ or ‘flogging the child to death’ with interventions rather than providing comfort care at the end of life.*  Ethical decision making: *Physicians and nurses were upset when other staff members were not willing to stop technological support, even when the interventions were doing nothing to change the prognosis.*  Relationships with others Bonding with parents: *Participants reported 78% of parents remaining involved with the child throughout the entire hospitalization, with 23% not staying involved.*  *Another problem described were parents who were not able to care for such sick infants. Twenty three percent of the respondents reported parents who were either never involved or who gave up on the children.'*  Suffering Suffering: *"This infant's short life was never comfortable. He struggled just to breath, day after day, month after month without relief unless he was paralyzed and heavily sedated. I frequently felt we were torturing the child just doing daily care."*  *"Finally I guided his parents to let him go. I hated myself and my colleagues for putting me in this situation, a task that should have been done so many months ago. There is nothing to be proud of, just grief and tragedy all around. We did a great disservice to this child."*  *"Parents never accepted the diagnosis of a lethal condition. Despite several neonatologists, cardiologists and an ethics committee referral, no one had the courage to discontinue respiratory support. In the last two months has had several cardiopulmonary arrests and was resuscitated because "everyone is scared of the parents"."*  Normality Inability to create a normal life: *"We are so busy in our ICU doing our tasks that the chronic kids are left for periods of time alone in their cribs with little interaction except mobiles."* Other healthcare resources: *Of note, the theme 'Cost of Care' was rarely mentioned.*  *Although respondents frequently discussed the emotional toll to all concerned, the monetary cost of long-term stays was very rarely (<1%) mentioned.* |
| Cescutti-Butler (2009) | Respiratory  Oxygen dependence: *"My babies did not fit into the criteria for going home early due to one of the twins still being dependent on oxygen. However if I had had an opportunity to take them home earlier I would have jumped at it"*  Gastrointestinal  Feeding difficulties: *"We kept on saying to the staff on neonatal unit that it was only Gray's feeding that was stopping him from going home, everything else was fine" Val explained how her twins were hospitalised for three weeks and for two of those weeks there were no major problems, as during this time her babies just needed to establish feeding. "I felt particularly sensitive around the issue of "topping up" and it upset me when my babies were topped up with formula milk through tube feeds. It made me feel inadequate and a failure for not being able to provide enough milk for my babies."*  Parental Support  Barriers to parental involvement: *"I felt at times that I had to ask permission to change my baby's nappy or check that it required changing although I understood that it was to protect Gray from too much "messing about" . I did not feel that myself or my husband could open the incubator and take Gray out. We had to wait for a nurse to say "oh, you going to have a cuddle?"*  *"You go in every day but you still feel guilty that someone else is taking care of your babies for you even though it's not your fault"*  Parental involvement: *"My husband and I were fully involved in the care of our babies and felt able to question and challenge decisions"*  *"During our baby's stay in the neonatal unit both myself and Peter were fully involved in our son's care and did most of the caring such as nappy changing and NGT feeds"*  *"give the parents the opportunity to care for their babies sooner than expected with help, which to me sounds like a good plan"*  *"In terms of being in control-I think it was more that mostly we weren't"*  Support from healthcare professionals: *"The care and support from the neonatal staff was fantastic, and without this support I would not have known what I was doing or how to care for my babies"*  Healthcare utilisation  Duration of admission: *"When I was told I couldn't bring them home until term I was mortified because that was two months down the road and no way did I want to wait that long"*  Healthcare workers-Knowledge and Competence  Consistency of decisions: *"One member of staff indicated Christmas and another New Year for the discharge of Gray. We knew that it was only the feeding that was delaying his discharge and so we kept on asking if we could take him home tube feeding. We did not always feel involved in decisions about Gray's discharge despite us asking repeatedly if we could take him home. Just as we had resigned ourselves to being in hospital over Christmas we were suddenly told on the Monday (a week before Christmas) that I could room in that night with a view to going home either on Tuesday or Wednesday at the latest. This was very frustrating as now all of a sudden he's now allowed home!"*  Relationships with others  Effects on family and friends: *"During the eight weeks of our baby's stay in hospital my husband and I did find it difficult travelling back and forth, because we were mindful of our other child who was only six years of age at the time and we tried to maintain a sense of normality for his sake"*  Other  Physical appearance: *"My first impression of her was as "a tiny red thing"* |
| Chang (2014) | Neurological  Neurological symptoms: *I can’t feel some - my left - this is numb right here.*  Development-Gross motor  Issues of development and motor skills: *I fall down easily.*  Development-Fine motor  Difficulty with activities of daily living: *It’s almost to the point where she can’t pick up a spoon with this hand.*  *You have to put the key in the ignition. So he would always have to reach around and it’s very hard.*  Healthcare utilisation  Need for frequent treatments: *Like I have to miss my Friday lunches to go and sit with a teacher for the whole lunch and tell her what’s happened. And like I have to I’m always getting called down to the office for another physical therapist to come and talk to me, and I’m always like it’s like it’s not it’s like both that I have to miss school work for it, number one.*  Need for lifelong care: *She’s been in therapy since she was 2 weeks old.*  Relationships with others  Peer acceptance: *Like this young girl telling me Friday, the other girls are getting asked out and boys won’t talk to her*  *I’ve had four year olds tell me the other kids don’t want to play with them cause they have a dumb arm.*  *It’s hard for fitting in. Like kids will make remarks. Like what’s that thing? Why is it like that?*  Bonding with family and friends: *My family is really helpful.*  Pain  Pain: *It like hurts when you wake up in the morning.*  Normality  Ability to lead a normal life: *I don’t like people doing stuff for me at all.*  *She would spend all day trying to do one thing so she could do it herself, you know.*  Other  Physical appearance: *Cause this arm’s a little bit shorter than the other one, like lengthwise.* |
| Chia (2006) | Parental Support Parental competence: *"it's a parent education thing too-explaining to them what it actually means and how they can go about doing it and how often and how long it does actually take-you need to sort of explain to parents what to wear."* Healthcare workers-Knowledge and Competence Healthcare professional competence: *"I think they [nurses] need to have a knowledge of it, I think that's fairly important and I am not sure that everybody does have"* Relationships with others Bonding with parents: *"I find it a great joy when the mums do hold the baby against their chest... irrespective of whether it's a primigravida or a multigravida. You get the same buzz out of it and so do the dads."* |
| Chollat (2014) | Respiratory  Asthma: *Table 1: -asthma*  Gastrointestinal  Eating disorders: *Table 1: -eating disorder*  Neurological  Sleep disorders: *'sleep disorder' measured*  Development-Gross motor  Issues of development and motor skills: *Neuromotor deficits CP, unable to walk or walks only with aid CP, but walks without aid No CP but dyspraxia or motor coordination disorder*  Development-Fine motor  Difficulties with activities of daily living: *(Other motor disorders were defined as coordination disorders, difficulties in cycling, drawing, cutting with scissors, playing with construction toys or puzzles, washing him/herself, dressing or tying shoes, receiving psychomotor or ergotherapy sessions.)*  Development-Special senses  Hearing impairment: *Table 1: -hearing and visual disorders were measured*  Visual impairment:  *Table 1: -hearing and visual disorders were measured*  Development-Speech and Social  Language disorders: *Language disorders were defined by treatment by a speech-language pathologist*  Healthcare utilisation  Frequent readmissions: *Table 1: -hospital admissions were measured*  Psychiatric  Autism: *Autism, pervasive development disorders, treated with risperidone Hyperactivity or attention deficit disorder or methylphenidate medication or overall SDQ >17 or SDQ hyperactivity subscale >7 or conduct disorder as reason for a visit to a psychiatrist or a psychologist at age 11 y*  Need for educational support: *Has repeated a grade and/or receives/needs special support at school, has schooling difficulties, language disorder*  Psychiatric disorder: *Autism, pervasive development disorders, treated with risperidone Hyperactivity or attention deficit disorder or methylphenidate medication or overall SDQ >17 or SDQ hyperactivity subscale >7 or conduct disorder as reason for a visit to a psychiatrist or a psychologist at age 11 y*  Normality  Normal health: *Table 1: -parents perception of child's general health was measured* |
| Darcy (2008) | Neurological  Sleep disorders: *The qualitative data show that nurses are aware that increased noise levels in a NICU have detrimental physiological effects for the newborn, including alterations in sleep/wake cycles*  Other  Growth: *The qualitative data show that nurses are aware that increased noise levels in a NICU have detrimental physiological effects for the newborn...problems with appropriate growth and development.* Physiological stability: *The qualitative data show that nurses are aware that increased noise levels in a NICU have detrimental physiological effects for the newborn, including…* *immediate changes in vital signs,* |
| Docherty (2007) | Surgical  Need for multiple operations: *the mother also worried that there would be more surgeries*  Appearance of scars: *Of particular concern were the scars on Nick’s body from medical procedures, surgery,*  Parental Support  Support from fathers: *Her support system was very limited; her relationship with Allison’s father was sporadic and filled with conflict.*  *Because he thought the mother had had an abortion, the father was in shock at the baby’s birth and erratically provided support.*  Support from family and friends*: In addition, there were issues with her parents, who had custody of two of her other children. Her support network included her mother and the paternal aunt. Others in the family were not accepting of her pregnancy or the baby.*  *The mother felt that the community blamed her for her son’s illness, and this eroded her support.*  *As one mother noted: “It would scare me at times because they, I wasn’t understanding some of what they was telling me. And I’d stay upset a lot. But then my mom she would call back and she would get understanding.”*  Barriers to parental involvement: *As with most mothers of critically ill infants, they also struggled with seeing their sick child surrounded by and dependent on technology and worried about the immediate and long-term outcomes. This made visits overwhelming and stressful.*  Parental involvement: *Lisa’s mother felt special because of her competence in caring for her child, her feelings of responsibility for the child’s well-being*  *Being a single parent meant that these mothers were often alone in making complex decisions for their infant during hospitalization. One mother deferred all decisions to the staff. Others struggled to learn more about the infant’s health problem and became competent in making decisions.*  Parental ability to work: *“She’s put my life on hold completely. Um, because, I could’ve started work at a plant that I’ve been tryin’ to get on for the past, longest. And uh, goin’ back to school is out, too.”*  Healthcare utilisation  Need for frequent treatments: *the mother also worried that there would be more surgeries*  Healthcare workers-Communication  Communicating information effectively : *she became upset because she could not understand what the staff told her about the child’s illness and treatments “It would scare me at times because they, I wasn’t understanding some of what they was telling me. And I’d stay upset a lot. But then my mom she would call back and she would get understanding.”*  *'Most of the mothers had limited exposure to complicated tertiary care hospitals and were challenged in communicating with highly educated health care providers, in understanding their infant’s complex condition, and in making complex decisions related to care.'*  Relationships with others  Bonding with parents: *she felt very guilty and reported difficulties attaching to her child during her hospitalizations*  Suffering  Suffering: *she reported feeling...distress at seeing him suffer*  Normality  Ability to lead a normal life: *the mother also worried that...Lisa would not have a normal life*  Survival  Survival: *the mother began worrying about whether her son would live*  Other  Physical appearance: *Of particular concern were the scars on Nick’s body from medical procedures, surgery, and the total parenteral nutrition line.* |
| Ericson (2013) | Gastrointestinal Breastfeeding: *They experienced estimated breastfeeding as a more difficult method because they felt insecure about the infants' intake of breast milk* Parental Support Parental involvement in care: *"The mothers are strengthened in their maternal role and dare to trust their own abil-ity. The mothers can read the infants signals at an earlier stage and in a better way."* Healthcare workers-Knowledge and Competence Staff insecurity: *..Some staff experienced a loss of control after estimated breastfeeding was implemented as they did not know the exact intake of breast milk after a breastfeeding session... ...Some staff did not trust what they saw and what the mother was telling them. The staff considered that sometimes the reduction was too big and the infant did not get sufficient milk and lost weight. Other times, the reduction was too small and the infant got too much milk and vomited. ...Some staff experienced the decision making in estimated breastfeeding as difficult as they had to predict and evaluate decisions made from a more complex viewpoint. ...They experienced estimated breastfeeding as a more difficult method because they felt insecure about the infants’ intake of breast milk and struggled to use amore person-centered care approach where the mothers’ and infants’ states and needs were acknowledged.'*  Relationships with others Bonding with parents: *'Staff experienced estimated breastfeeding as a more supportive method for the development of the mother/infant relationship because of the uninterrupted closeness.' 'The staff stated that the mothers stopped comparing how well or how poorly other mothers’ infants fed after estimated breastfeeding was implemented'.*  Suffering Comfort: *"The infant can lie securely in the mother's arms directly after breastfeeding and not on a hard scale."* |
| Gaíva (2001) | Respiratory Nasal congestion: *"If having nose congestion is something common for every premature baby"* Gastrointestinal Regurgitation: *"The other day I got really scared, the baby regurgitated, and milk came out of her nose, I went crazy"*  Frequency of defaecation: *"A handbook with instructions about elimination, like if it's normal for the baby to go two days without defaecation"*  Feeding difficulties: *"From the mothers' reports, it is observed that activities like...breastfeeding, become complex when taking care of a baby."* Healthcare utilisation Frequent readmissions: *"I'm afraid of the baby having to return, I think they could talk more about the premature baby, the risks"* Healthcare workers-Communication Keeping parents informed: *"They could provide a manual about what the baby might have" "Preparation for discharge refers to instructions about care with hygiene, feeding – focused on breastfeeding, administrating medications, and returning to the outpatient clinic for follow-up visits. The mothers reinforce their need to receive instructions at the moment of their child’s hospital discharge."*  *"In view of the statements, it is observed there is a need for educational material covering the care that should be offered to newborns in risk situations."* |
| Garcia (2004) | Healthcare workers-Communication Communicating uncertainty: *"It's difficult, you know. I think as medical practitioners it's, I think in medical practice we find it difficult to ... We have to accept that we don't have all the answers in terms of treatment ... and of course I'm sure that comes as a disappointment to the families."* Candour: *"It's about trust, you know. I will trust someone who was honest and said he didn't know (more) than someone who lied."* Healthcare workers-Knowledge and Competence Ethical decision making: *"It's difficult, you know. I think as medical prac-titioners it's, I think in medical practice we find it difficult to ... We have to accept that we don't have all the answers in terms of treatment ... and of course I'm sure that comes as a disappointment to the families."*  *"but when you actually mention this, say, "Well, in fact you know, we don't really know what's the best treatment," it is a delicate moment. I'm not really bothered from how they perceive me, as a person, because you know, there's lots of things I don't know in life, but their confidence of how their baby's going to be managed, that's the issue that comes up."* Other Iatrogenic harm: *"... I don't think we know...there are potential toxicities, very real toxicities associated with it, so there is this balance of...benefit and harm..."* |
| Gontijo (2008) | Healthcare workers-Knowledge and Competence Consistency of decisions: *'In interviews with mothers, it becomes more evident, because they claim that only become effectively adopt the kangaroo position when they are sent to Kangaroo infirmary. Even so, kangaroo position, It is not adopted continuously in the second stage.'*  Growth Growth: *evident satisfaction with the method, especially about the best development of your child, highlighting the weight gain which is the main factor* |
| Green (2015) | Parental Support Coping with maternal illness: *Understandably, the nurses were very concerned about the mental health and well-being of all mothers in the NICU, however, their level of concern was heightened for mothers of extremely premature IVF babies. They emphasised that it was only natural that mothers who had suffered multiple losses, and potentially another loss if the baby did not survive, to experience severe psychological distress. Another loss could be the loss of a ‘normal baby’. One nurse spoke of her belief that mothers could be diagnosed with depressive conditions, particularly when they took home a baby that had ongoing health problems or a disability, and the mother came to understand the reality of her situation.'*  Healthcare utilisation Inappropriate treatments; *For the nurses, all babies are precious, and while they recognised that pregnancies conceived through assisted reproductive techniques were precious to their parents, they rejected the idea that these babies were more precious than babies who were conceived naturally. The nurses were concerned that the metaphor of ‘precious’ used to described IVF babies, could create a view that mode of conception will affect the treatment options offered to parents of extremely premature babies.* Healthcare workers-Communication Communicating in challenging settings; *The nurses believed that when parents requested ‘everything’ (Nurse 13) to save the life of their baby, they would not know what ‘everything’ meant.* Healthcare workers-Knowledge and Competence Ethical decision making; *...all of the nurses who were interviewed had seen and worked professionally with many families with a history of infertility, and hence had first-hand experience witnessing the complex dilemmas and realities faced by infertile couples*  *The nurses understood the parents’ desperation to have a baby and experience parenthood. They believed on the other hand that such desperation should not be the sole determiner of whether the baby survived.* Suffering Suffering: *"It's hard looking after a baby where it's being kept alive just to meet the needs of the parents, because they want a baby."*  *Participants knew the baby would experience pain and suffer from intensive care therapies. All the nurses believed that they were affected by the baby's suffering, because it was "agonising for the staff who have to look after them"* Survival Survival: *"It's their one and only chance of having a child. They can only see that immediate sense of having a child. . .they want to take home a baby."*  Survival with disability: *"As hard as it is for us to understand, they are happy to have a child of their own regardless of what it is. They're happy to suffer the consequences, so to speak, just to say that it's their own; that it's something that they can love."*  *"It isn't up to us to say what is quality of life, because parents might think that even if the child was disabled, that it was better than not having a child at all."*  *"They want to take a baby home and often voice that they don't care what the infant is like."*  *"To have something that's alive, I can understand that. I can understand somebody saying, 'I want that infant in preference to nothing at all'."*  Other Long term effects on parents: *"We should be looking at Postnatal Depression after the baby goes home. . .Once they actually get a baby home, that's when the reality sets in. All the triggers are there."* |
| Gregory (2012) | Respiratory Oxygen dependence: *Oxygen status* Gastrointestinal Feeding practices : *Participants were asked about the clinical criteria that are used to determine initiation, delay, and advancement of enteral feeding and about the specifics of enteral feeding administration (ie, bolus vs continuous, frequency of feeding, type of feeding, and nutritional fortification added to feeding). Finally, participants were asked to report the volume at which patients were considered to be at “full feeds” and how PN was discontinued.'* Infection Sepsis: *Identified outcome: Sepsis* Healthcare workers-Knowledge and Competence Consistency of decisions: *Compliance with guidelines Practice patterns of the physician Practice patterns of the nurses* Growth Growth: *Time to return to birth weight* |
| Griffin (1997) | Parental Support Preparation for NICU admission: *Parents described benefits of the tour, including that it (a) decreased their fears, (b) inspired hope for their newborn’s prognosis, (c) provided reassurance about care in the NICU, and (d) prepared them for their newborn’s NICU hospitalization. All parents described at least one of these benefits, including five mothers who said the tour was overwhelming or difficult because of the appearance of the newborns.*  *'For several mothers, the tour inspired hope for their newborn’s prognosis, especially when the mothers saw very premature infants who were said to be progressing well.*  *Parents evaluated and provided suggestions on the way the tour was arranged and conducted and offered advice to other parents. In general, all parents recommended that parents in similar circumstances should be offered a prenatal tour of the NICU.*  *Parents advised that more health care providers suggest tours to parents diagnosed with a high-risk pregnancy. Two mothers also recommended that other perinatal health care providers should tour the NICU so that they can be supportive to parents. One mother perceived that her need to tour the NICU was not supported by the staff on the antepartum unit.*  *Parents who toured with their partners commented that having each other as a support person was helpful. They recommended that the tour be scheduled so that the partner or other support person could accompany the parent.* Healthcare workers-Communication Communicating information effectively: *Parents re-ported that it was important to receive detailed information on the following: ( a ) newborns who had a di-agnosis or gestational age similar to what was antici-pated for their newborn; ( b )a description of equipment for the newborn; (c) roles of staff members; and (d) a description of the parental role in the NICU, including the visitation polic It was important to parents to hear about the parental role....However, all parents did not perceive that they received adequate information on the parental role.*  *The need for more specific information became apparent to parents after their infants were cared for in the NICU. These parents indicated that they wanted more information on expectations for their role in the NICU, breastfeeding, sibling visitation, and the potential for the newborn to be transferred from the NICU to another unit before discharge. Two parents suggested that handouts could supplement or reinforce information that was given during the tour and assist parents to inform family and friends about the NICU.*  *Parents reported that the tour should be individualized to meet the specific needs of parents. Parents perceived the tour as individualized when they went as a couple or an individual rather than in a group, had an opportunity to ask questions, and saw newborns who had a diagnosis or gestational age similar to that expected for their newborn. Therefore, it was critical for the nurse conducting the tour to know the parents’ maternal-fetal diagnosis. Several parents made additional suggestions, such as having an opportunity to go on a second tour or changing the order in which the NICU patient care areas are shown; these demonstrate the parents’ individual needs.* Healthcare workers-Knowledge and Competence Healthcare professionals behaviour: *"Knowing they do care about them and they do realize they are human and not machines . . .you could feel that they really cared and worried"*  *Parents reported that the tour was comforting and reassuring because it gave them an opportunity to observe the type and quality of care that the infants received.*  *Hearing specific information about primary nursing also helped some mothers to feel more comfortable. These mothers explained that it was reassuring to know that their questions could be answered because the primary nurse would know their infant.*  *Most parents reported that the nurses who conducted the tours were knowledgeable and comforting. These nurses were described as compassionate, concerned, helpful, and considerate of the time parents needed to understand the information and ask questions.*  Staffing levels:  *It was especially helpful for the parents to see so many nurses and physicians in the NICU.* Relationships with others Effects on family and friends: *The tour gave mothers information about the NICU they needed to share with other family members. One mother indicated that she had gained an understanding of the unit and was better prepared to talk to her child about the NICU. Three of the four mothers who were not accompanied on the tour by the fathers reported that they shared information about the NICU with the fathers, which was comforting to them. One of these mothers described her husband’s reaction to their infant’s admission to the NICU, “My husband was calm because I had already told him what to expect.”* Normality Thriving: *"The tour gave me hope that he was going to be fine. Seeing babies younger than him thrive. . . and then seeing the babies approximately his age survive, thriving, and doing well."* Other Physical appearance: *"it's still frightening to see babies that small."* |
| Hanrahan (2007) | Gastrointestinal Choice of milk for feeding: *"it’s like they [scn providers] didn’t inform us when they were trying to feed my daughter [formula]"* Parental Support Care provided close to home: *"and nurses’ opinions would come into it, too. if they’re aware…that parents aren’t showing up, including in the evenings…then that may weigh into the decision-making process…make us want to get the baby close to home because we know the parents can’t get here."*  *"[i was glad our infant was transferred to the com-munity hospital] because of lack of transportation.Because then i [was] able to see him more."*  Healthcare workers-Communication Communicating information effectively: *"i don’t think that [it] is communicated as well that your baby’s well enough to go back to a unit that isn’t as intensive as the one that it’s coming from."*  *"they were originally using all these technical terms [about back transport], and he [the infant’s father] was like “What is that? slow down, we still have no idea what it is.”"*  Keeping parents informed: *"i mean, i found at [the nicu] they explain more to you what was happening with my daughter, what they were doing, how well she was doing. You know, at [the scn] you would go there and they’re just doing something. they’re not—they don’t have time. You know you have to approach them…and say, okay, what’s going on now? how well is [my infant] doing today? You know, stuff like that. i want them [scn providers] to, you know, talk to me, let me know, don’t sugar coat what’s, you know, going on."*  *"it’s like they [scn providers] didn’t inform us when they were trying to feed my daughter [formula] and she was on breast milk, so she threw it all up.…they didn’t tell us until we got up there."*  *"i asked so many questions and read all the charts every day, and i probably angered them. squeaky wheel gets the oil, as they say."*  Treating parents with respect *"i got yelled at by a nurse at [the scn] for rubbing my son’s foot [even though that was okay at the nicu]. and rubbing his hand while he had a hold of my finger. i was just rubbing his little palm, and then she told me not to do it because it would stimulate him." "i would’ve liked them to greet me as a parent—you know, acknowledge me."*  *"[My experience at the scn] could have been better; they could have made me feel comfortable there."* Healthcare workers-Knowledge and Competence Staffing levels: *"[at the scn] you’re not gonna have somebody there all the time, right next to your baby."*  *"one time we went [to the scn] and it was like eight-thirty at night. and there was only like three or four nurses in there, and it was a full room. and there were babies in every bed. and they were having a lot of problems with this one little boy, and they left all the other babies and went to him. so i ended up staying there real late because i wasn’t gonna leave my daughter by herself and have something happen to her while they’re just with this one child. You know, i think staffing is a really big issue [at the scn]."*  Identifying who is responsible for care: *"sometimes we’re not real clear who to follow up with."* |
| Hefferman (1999) | Gastrointestinal Other gastrointestinal malformations: *"One mother, left with one surviving twin who has an ileostomy, told me that she is glad the sibling died and does not have to endure what the living twin is enduring."* Surgical Requirement for ileostomy: *"One mother, left with one surviving twin who has an ileostomy, told me that she is glad the sibling died and does not have to endure what the living twin is enduring."* Healthcare utilisation Need for lifelong care: *"MDs are making such choices for parents and when the outcome is disastrous they just expect parents to take home severely handicapped babies and deal with life-long problems."* Suffering Suffering: *"Three weeks of high pressure only proved to prolong her death rather than improve viability...Being one of the nurses at that bedside every day was frustrating and often agonizing. I definitely feel that even with all our technical expertise, we failed to give good and sensitive care to both this particular infant and her parents."*  *"The problem [with] our high tech machines is not only do they prolong life, they often prolong death. I am not saying we should never attempt to salvage VLBW babies. We have some miraculous success stories. But we should have some guidelines to follow and the decency to stop before we begin to do harm."*  *"It often seems that when we resuscitate these edge of viability infants it is to please the parents, especially in the IVF environment that we have today. The concern is not for the comfort of the infant; the concern is to give the parents their child no matter what."*  *"One mother, left with one surviving twin who has an ileostomy, told me that she is glad the sibling died and does not have to endure what the living twin is enduring."*  *"We poke, prod, and torture them for weeks, give a lot of false hope to the parents, stress the staff, and then they are taken off and allowed to die . . . for us it is ethically the worst part of our job."*  Normality Normality: *"It is hard to care for very small sick infants whose potential as a normal child and adult is poor."* |
| Hentschel (2006) | Healthcare workers-Communication Awareness of parental views: *The data show that in 9 cases the clinical team had no knowledge of the parents’ wishes.* Pain Pain *: Table 3 -summary of medical staff's perceptions of parents' wishes concerning RIC* [Restriction of ongoing Intensive Care] *Agree to RIC: “Avoidance of pain/suffering”* Suffering Suffering: *Wishes of parents as percieved by medical staff “Avoidance of pain/suffering” “To let the infant die in peace”*  Normality Normality: *Wishes of parents: “A healthy child” “To take the child home”*  Survival Survival: *-summary of medical staff's perceptions of parents' wishes concerning RIC: “Survival of the infant” “Maximum therapy” “Therapy even in case of low chance”* |
| Holditch-Davis (2003) | Gastrointestinal Nutritional intake: *"Ever since he's been home, we've kept records of how much he was eating, on a daily basis."* Neurological Seizures: *"I thought I'll time it and see how long these things last-which was anywhere from 5 to 7 minutes. And his color would change, and the way he did his eyes. They rolled. And his eyes would water, and he would tense up. I explained this to the doctor. And he was the one that said it could possibly be seizures. So we're going to take him in and have him tested. But this is something that we were told could be a possibility, and I don't see it as anything major."* Infection Susceptibility to infection: *"particular about her germ thing. I don't want her chewing on every-body else's toys or what have you."*  *"The biggest worry right now is when he is going to get sick. I’m sure he's able to handle it, but at the same time, we’re overly protective and don't let people in his face"* Healthcare utilisation Recurrent sickness: *"We've only put him with other children for the past month. The biggest worry right now is when he is going to get sick. I’m sure he’s able to handle it, but at the same time, we’re overly protective and don't let people in his face."* Relationships with others Childhood happiness: *"But to me I look at her and I don't see it all. I see this little happy little thing!"* Normality Normality: *"And how helpless he was and that I couldn't hold him. That was really the hardest thing."*  *"But I don't look at her as a premature....But to me I look at her and I don't see it all. I see this little happy little thing!"*  *"Is he going to be his normal size? . . . If he was supposed to be small then that's fine. He can be small. But I guess I'll never know."*  Survival Survival *"It hurts. I didn't know, I didn't know if they were going to make it or not."*  *"I have a fear of them dying. I'm still scared because they were premature that that could happen to us. And grant-ed that could happen even if they weren't premature!"*  Growth  Growth *"The most difficult thing for me is the weight thing. Is he big enough? Is he not big enough? Should he have been bigger? Would he have been bigger? How big would he have been when he would, if he was born when he was supposed to be born?"*  *"just look at her as a little baby. . . . The doctor says she's 3 lb under weight. So that's like 20% bodyweight."* |
| Hussey-Gardner (1998) | Healthcare workers-Communication Developing a caring relationship: *"I couldn't get over how gentle she was. It seemed like she was truly concerned"* Normality Normality: *"Being reassured that he was on line for how old he was...Just reasurance he was doing well"* Growth Growth: *"She was born so early, it's nice to see that she's finally catching up with how she's growing"*  *"I asked more questions about where he was supposed to be..."* |
| Jirapaet (2006) | Respiratory Mechanical ventilation: *"I couldn’t ﬁnd time to change the adhesive tape, that was wet by saliva, of the endotracheal tube of another baby … it caused tube dislodgment …"*  *"I was ﬁlling [free ﬂow of] water into the humidiﬁer chamber and was distracted by a call to assist a physician in an emergency case. I for-got to close the line clamp … water ﬂowed into the baby’s lungs unnoticed."* Skin Extravasation injuries: *“The i.v. site was covered with cloth.... leaked for a long time, skin area was inﬂamed and edematous like popeye’s arm.”*  *“I was unsure if the baby had an IV leakage and asked a senior nurse to conﬁrm it. But she was busy …. I saw the baby again when there was tremendous edema."*  Burns; *"wasn’t lazy in changing the pulse-oximeter sensor site [recommended to relocate q3-4 h] … But I didn’t believe that it could really burn the skin until I had experienced it."*  Pressure sores: *"I had to skip changing a [hydrocephalus] baby’s posi-tion for that shift … Her head developed pressure sores."* Healthcare workers-Communication Communicating information effectively: *"Doctor wrote a new order on the patient chart and left it on the desk, but didn’t say a word …. The order should have been taken but no one noticed it."* Healthcare workers-Knowledge and Competence Medical errors: *“I had to draw an antibiotic from a vial that was diluted by another … without knowing that it con-tained the wrong preparation …"*  *“Doctor wrote 3 like 5.”*  *“I set the infusion rate at 12 mL/min instead of 1.2 mL/min. I couldn’t see the faint decimal point.”* Other Iatrogenic harm: *“I performed a venepuncture without using a radiant warmer properly … baby developed hypothermia.”* |
| Johnson (2007) | Parental Support Parental involvement: *Being involved in caring for the infant was described as being needed by every mother in the study.* Relationships with others Bonding with parents: *“made me realize that I know my little girl”*  *Many of the mothers spoke how holding helped them “understand” their baby’s needs better Mothers related feelings of “being needed.”* Suffering Comfort: *“I will make you feel so much better in my arms,”* Normality Normality: *“mommy will sing to you our favorite song for as long as you want”*  *“loud noises the rest of the time, we can escape to be alone for an hour.”*  *as one mother stated, “you can’t learn how to be a mother from a book. You have to learn with your baby.”* |
| Jones (2015) | Gastrointestinal  Breastfeeding: *“it was her first time and just explaining to her how, the right way to breastfeed and attachment and things like that (Nurse 9)”*  *"We went through from start to finish any questions she had. We ran through a breastfeed, we had all the props out, and just with the use of the props and things it was a bit easier …It actually worked out really, really well. After that, we got the baby to breastfeed beautifully and it was nice"*  Parental support  Culture differences: *But I think sometimes some of the Aboriginal Community can also be hard to talk to. I think that’s just their environment that they’ve been bought up in*  Support from healthcare workers: *They’d say “Well at the end of the day go with your feelings of what, if you feel she’s feeding really well when you do a certain thing, then keep doing it and if you do something else it and doesn’t work, then stop doing it,”… “You will know best because you just know how it works with your new baby”*  *‘So it's just about giving them lots of options and supporting them to make the choice that’s right for them’*  Support from family and friends: *For example, one woman requested that her friend be able to come into the nursery and hold her baby. The woman had no family members in Australia, and her friend was the only person providing support. The nurse caring for her baby refused her request on the grounds that the unit had a ‘policy’ that only parents and grandparents could hold the baby. This created distress and conflict.*  Healthcare workers-Communication  Communicating information effectively: *One of the most common and important issues for both parents and nurses was the concept of sharing ‘information’. Parents ‘wanted’ nurses to ‘provide’ information and nurses ‘wanted to provide’ parents with information. Interestingly, fathers had a tendency to mention information first compared to mothers. From the parents' perspective they wanted to know ‘everything’ from what they considered to be ‘general information’ about for example what ‘creams’ they should use on their baby to how equipment worked and, of course, the condition and progress of their baby.*  *I feel like I’m receiving all the information that I need, so I’m really extremely happy about that even though sometimes we’re not asking questions, they will give us additional information*  Communicating in challenging settings:*I think your very young mothers, very hard to talk to because they’re babies and they don’t understand, they see us more as a authority figure as in someone that could be their friend, and be helpful ……*  Developing a caring relationship: *Nurses who ‘listened’ and were ‘empathic’ were highly valued. Parents also used words such as ‘friendly’ ‘approachable’ and ‘caring’ to describe the communication style of supportive nurses; I just felt she had a way with words and made me feel really safe (Regional Mother 4).*  *I have been nursing for a while now, the most important thing you would do with any patient; whether it a*  *baby, whether it an adult, you build up a rapport*  Keeping parents informed: *She rang the previous room, and they told her, “Well, your baby’s not here anymore,” so they put her through to my room, I got the phone call, to me and she was like, well, just a bit cranky at the fact her baby had been moved without any prior knowledge to it, and I coped a brunt of it (Nurse 13).*  Treating parents with respect: *Yeah, I think the nurse was worried about the whole touching and whatnot … Like she’s very full on with protocol but she was very abrupt and didn’t explain things. So she upset mum and this upset me a bit too (Metropolitan father 26).*  Healthcare workers-Knowledge and Competence  Consistency of decisions:*‘Everybody had a different point of view but they were opinions, not facts. So that was huge, don’t even get me started on that, that was just a nightmare. (Regional mother 6)’*  Healthcare professionals behaviour: “*I asked “Can you come?” ‘Cause they had to check that it’s down the right tube, …. And she didn’t come and do it and I had to go and get her and say, “Can you help me?” ‘Cause I’m not allowed to do it by myself. And then she was talking and it took her 5 minutes to finish her conversation with the other nurse, and it wasn’t a nurse related conversation it was just a casual conversation … Like I felt a bit (sic) she wasn’t their priority.”*  Normality  Normality: *A major focus for parents was seeking information that told them that what was happening was ‘normal’ and that everything was going to be ‘fine’.* |
| Kavanaugh (1997) | Parental Support Support from family and friends: *Certain behaviors of family, friends, and health care providers were perceived by parents as supportive. Some behaviors that were described as helpful by the parents were not unique to wny specific relationship, whereas others were confined to relationships with health care providers. Three behaviors that were not specific to any particular relationship were accepting the parents’ feelings and behaviors, being there, and sharing the experience.*  *Parents reported that they did not attend a support group because they could not share their experience in a large group or because their family provided adequate support.*  Support from healthcare professionals:  *The other behaviors described only with respect to health care providers were giving information, providing competent care, and giving special attention.*  *To the parents, being there meant being available. Nurses were available to the parents by being physically present or by being close by if the parent needed the nurse, especially if the mother was alone during labor....Parents used the term “share” when they described individuals who genuinely understood their experience.*  *Only one mother recalled sharing the loss with someone who was not a bereaved parent. This mother described her experience with a nurse during the delivery.*  *Parents perceived that health care providers gave them special attention by breaking hospital rules, giving additional time, and making the parent a priority among other patients. Breaking hospital rules included expanding visitation to allow for unrestricted visits from family and friends.*  *Most parents could not identify the reason for inadequate pain relief. One mother stated that she was denied an epidural by an anesthesiologist, who told her that “women have been doing this for thousands of years without an epidural.* Healthcare workers-Communication Communicating in challenging settings: *"And I don't want some fluke off the wall, you know. You don't have to tell me. But what I'm saying is the things that have a reasonable chance of happening" the parents reported that when delivery was imminent, the obstetricians explained that the newborn would have had a better chance of survival had the pregnancy been maintained for an additional few weeks. In the other instance, the mother reported that the obstetrician told her about the likelihood of her twins’ survival when she was treated for preterm labor.*  *Only one couple perceived that they were given an opportunity to discuss how much aggressive care they wanted for their twins. This couple was the only one to report that they felt prepared for their twins' deaths even before they were born.*  *Certain decisions, such as choosing the type of burial, were especially difficult because of the parents’ emotional state or lack of experience. Most parents had no experience making funeral arrangements. All parents reported that it was helpful to have information, guidance, and additional time before making final decisions.*  Keeping parents informed: *All parents reported that they wanted health care providers to keep them informed of the mother’s and newborn’s condition and treatment plans. When health care workers’ provided honest, straightforward information in understandable language, parents gained a sense of control over the situation and their fears decreased.* Healthcare workers-Knowledge and Competence Health professional competence: *Most of the parents recalled specific incidents that they perceived as poor medical care; typically, these incidents involved technical procedures or medical knowledge...Parents expected expert knowledge, pain relief, and consistency in care: “Team” was the word used to describe staff who were organized and had communicated well among themselves, especially when transfer of care occurred between shifts or units.* Relationships with others Effects on family and friends: *Family and friends were perceived as available to parents when they visited or called. Parents stressed the importance of others’ acknowledging their loss and being willing and unafraid to talk to them. Comments that parents perceived as inappropriate were those that minimized the loss, provided unsolicited advice, or criticized the parents on their health care.*  *Breaking hospital rules included expanding visitation to allow for unrestricted visits from family and friends.* |
| Koh (2007) | Healthcare workers-Communication Using aids to communication: *Most of the mothers in both groups were positive about having their conversations with the neonatologist taped.*  *six of the mothers in the control group could not recall their conversations with the neonatologist* |
| Leavy (2015) | Skin Appearance of scars: *In addition, hospitalization and different interventions in their first days of life have left marks on their bodies. Such marks may become a stigma if their peers give them a negative meaning, leading to prejudice and discrimination*  *“I do not like the scar on my belly [...]; I was at the beach and everyone kept staring at me like `That is a big scar!´”*  *Being afraid of becoming the laughingstock may make them feel insecure, thus affecting the construction of their own identity: “I think about these things (scars) when it comes to girls; it makes things more difficult because girls think you have washboard abs but your stomach is actually all flabby because of a poorly healed scar resulting from a surgery” (FG 6), said one participant in relation to a keloid resulting from a surgery. However, some take ownership of these marks and give them a new, positive meaning: “When I was young, I felt embarrassed when I had to take off my t-shirt, I thought they would all stare at my scar, and I developed a body image complex. Now I have tackled this issue and it has become part of my story, thanks to this scar I am alive” (FG 9). In this case, the scar becomes the footprint of a surgery that allowed the participant to survive, and this is more significant than the stigmatizing vision of others.* Surgical Appearance of scars: *“I do not like the scar on my belly [...]; I was at the beach and everyone kept staring at me like `That is a big scar!´” “I think about these things (scars) when it comes to girls; it makes things more difficult because girls think you have washboard abs but your stomach is actually all flabby because of a poorly healed scar resulting from a surgery”*  *“When I was young, I felt embarrassed when I had to take off my t-shirt, I thought they would all stare at my scar, and I developed a body image complex. Now I have tackled this issue and it has become part of my story, thanks to this scar I am alive”* Development-Gross motor Ability to walk: *"I received lots of stimulation; otherwise, I would have ended up in a wheelchair."* Development-Speech and Social Language disorders: *"I also had a hard time learning to talk."* Healthcare utilisation Frequent appointments: *“I felt left out, I was always missing school because I had to go to the hospital for check-ups”*  *Mixed feelings are observed in those participants who have special health care needs at the hospital, from familiarity and thankfulness to rejection and discomfort: “It is a complicated subject because I am alive thanks to the hospital, but I am tired...Now I just go for scheduled check-ups, but when I am there, I just want to leave"* Healthcare workers-Communication Communication with ex-neonatal patients: *Some participants indicated having had a negative experience with health teams: “They said that I was not going to walk and that I was going to be blind, deaf and mute. They would give me no hope and say: ‘This baby will not make it to the sixth day…´” (FG 8). “My father was told that when birth is premature, either the baby or the mother dies... but here we are, both of us are alive” (FG 5). Likewise, they felt uncertain about what aspects of their present health status were actually related to their history of premature birth.*  *The hospital and the relationship with the medical team play a significant role, which is sometimes contradictory. On the one hand, many participants stated that they were thankful and appreciated the bonds established in the hospital setting. On the other, they often mentioned how medical diagnoses were uncertain or pessimistic regarding their survival and potential sequelae.*  Psychiatric Need for educational support: *Overlapping of special health care needs and school hours resulted, in some cases, in a poor school performance: “I felt left out, I was always missing school because I had to go to the hospital for check-ups” (FG 10). Repeating a grade was frequently associated with learning difficulties and an achievement gap in relation to classmates. Several interviewees had to attend a remedial education program. Most of them felt comfortable in such setting because it helped them overcome their learning difficulties and favored their socialization: “I attend a special education school because when I was in a bilingual school I could not advance to the next grade, the other kids mocked me or left me aside, they did not understand my problem. I am doing fine now” (FG 7).* Relationships with others Overprotective parent-child relationship: *They perceive their parents consider them to be more vulnerable and dependent on their custody than the rest of their children or their peers. This may lead to inter- and intragenerational conflicts: “I have always been treated differently than my sisters. Yes, they tend to be overprotective, that bothers me” (FG 2)... This is a traumatic experience for parents, and it is projected into their present relationship with their children...When overprotection is based on giving excessive care to preterm children, the relationship between parents and children becomes distorted. In this regard, a quadriplegic adolescent stated: “All moms left, but my mother would stay all morning at school with me [...]; I started caring for myself and I had to push her away a bit so that I could lead my own life” (FG 5).'* Suffering Ex-patients separation from their suffering: *For ex-preterm subjects, prematurity is more about their parents’ experience than about themselves: “When I tell my story, I use the third person; my mom went through this, not me. She considers my story her own” (FG 1). “We are based on what we were told, not on what we experienced” (FG 8). Participants also emphasize their parents’ suffering and uncertainty: “Parents suffer much more than us; they had to suffer waiting for us to get better and see us in that status” (FG 1).'* Normality Being treated normally; *“I have always been treated differently than my sisters. Yes, they tend to be overprotective, that bothers me”*  *“I wanted to have a piercing but my mother would not let me because they gave me lots of pricks when I was hospitalized and I had suffered, but I do not remember it”* Survival Survival: *“I am proud of having been born preterm. I almost passed away, and so did my mom. We both made it and it was a miracle”*  *Beyond life experiences, even though most participants recognize medical efforts, they attribute their survival to a miraculous and extraordinary event.*  *"Now I have tackled this issue and it has become part of my story, thanks to this scar I am alive”* Schooling: *“I attend a special education school because when I was in a bilingual school I could not advance to the next grade, the other kids mocked me or left me aside, they did not understand my problem. I am doing fine now”*  Other  Self-identifying as premature: *'The meaning that subjects assign to having been born preterm varies according to their health status, sequelae and life experience. Those who had sequelae and special health care needs 18 in their childhood associate their preterm birth to a life of efforts and difficulties* |
| Lee (2005) | Parental Support Culture differences: *3 families felt strongly that their stress derived from differences in the medical management approaches between the United States and their homeland.*  Parental involvement: *almost all of the parents expressed a desire to become more involved with the care of their infant*  Healthcare workers-Communication Language barrier: *the stress derived from communication with HCPs was mostly related to language barriers for the new immigrant parents.*  Keeping parents informed: *Other parents experienced stress from...not being thoroughly informed about their infant's condition or treatment plan.*  Communicating information effectively: *Other parents experienced stress from unknown medical terminology*  Healthcare workers-Knowledge and Competence Healthcare professionals behaviour: *HCP* [Healthcare professional] *behaviour as stressor for parents*  Identifying who is responsible for care: *2 families expressed frustration related to frequently changing bed spaces or hospital units and difficulty accessing doctors.'* Relationships with others Effects on family and friends: *Worry about the possibility of upsetting their own parents. Three families did not tell their own parents about their infant’s problem because they were worried that it would upset the grandparents.*  Family resources: *3 families felt overwhelmed by a lack of resources (especially in the area of family support)* Other Parental perception of uncertainty: *parent's perception of infant's severity of illness -parent's perception of infant's illness and future impact (i.e. non-specific developmental issues)*  Physical appearance: *Both mothers and fathers found their infant's appearance and behavior to be the stressors with the most impact* |
| Lou (2009) | Respiratory Mechanical ventilation: *"She had been put on a respirator. It was not so bad as I had imagined. I could see that she calmed down. She did not struggle so much and…But the doctors gave me a shock when I came to see her. They had taken an X-ray, and they could see that her lungs were white and stiff and I was told that she was not far from dying"*  Frequent respiratory illnesses: *"There were lots of masks and nebulisers during those years"* Gastrointestinal Oral feeding: *"It is small things.. I remember the very first time he ate porridge"*  Neurological Sleep disorders: *Subsequent to an account of the son's disturbed sleep at night for several months after discharge, which was an enormous challenge to the couple, a mother described the boy as follows: "He was such a giving child, full of joy!" … adding in a low voice… "Most of the time".* Skin Appearance of scars: *"The only reminder today is an ugly scar"* Development-Fine motor Ability to feed themselves: *"It is small things.. I remember the very first time they ate porridge. In just stod there in the kitchen and cried my heart out...it was the fact that I was able to experience my children eating with a spoon. It was great!"*  Development-Special senses Visual impairment: *At the time of the interviews, the only major sequel was in one child with seriously impaired vision.* Healthcare utilisation Need for frequent treatments: *"There were lots of masks and nebulisers during those years"* Psychiatric Dyslexia: *"We were told that when he started school he might have problems, dyslexia or learning dis-abilities."*  Need for educational support:*"We were told that when he started school he might have problems, dyslexia or learning disabilities."*  Relationships with others  Bonding with parents: *"He might also… When I was working in the kitchen, he might come running at full speed from the family room, hug me.. around my legs.. he was no bigger than that.. and say: "Oh, you are the very best mum".. and off he went again.. He just wanted me to know!"* Normality Normality: *Finally, a mother called it a developmental land-mark when an older sister dared show her irritation towards her little brother, "no longer treating him as if he were made of glass"*  *"They get up and try again until they succeed. That is the same way he keeps on trying."*  Survival Survival: *"They had taken an X-ray, and they could see that her lungs were white and stiff and I was told that she was not far from dying."*  *"Still, I consider the most important moment in my life.. the very second I pushed the pram over the doorstep of the neonatal unit. I remember it very clearly. I had been looking forward to that for two months."*  Survival without disability: *"And afterwards you are worried about how they are going to survive. If they would have impairments, and so on."* |
| Malhotra (2015) | Respiratory Mechanical ventilation: *Significant factors that make professionals categorise a baby as 'sick' High ventilator settings*  Oxygen dependence: *Significant factors that make professionals categorise a baby as 'sick'… Oxygen of over 30%* Cardiovascular Hypotension: *Significant factors that make professionals categorise a baby as 'sick' "hypotension"*  Presence of Patent Ductus Arteriosus: *Significant factors that make professionals categorise a baby as 'sick'…PDA* Gastrointestinal Necrotizing enterocolitis: *"If we are trying to stimulate the gut before it is ready making it more prone to having complications of either feeding intolerance or, more severely, necrotizing enterocolitis."*  Initiating enteral feeds: *"MEF* [minimal enteral feeds] *should be initiated in ﬁrst 2-3 days of life as long as the baby is stable."* Healthcare workers-Knowledge and Competence Consistency of decisions: *"it is difﬁcult to have faith in the decisions when they don't even agree with each other"* |
| Miljeteig (2006) | Parental Support  Maintaining hope: *The informants described how parents thought there was hope so long as the child was in hospital, and the doctor’s dilemma consisted in both keeping the parents’ hope alive and in giving them a realistic picture of the situation and of probable outcomes.*  Healthcare workers-Communication  Communicating with parents: *Most of the informants reported dilemmas in relation to parents. The most frequently mentioned problem was communication with parents of poor education and low socio-economic status. Many said that parents did not understand medical terms, and were thus unable to appreciate the seriousness of their child’s condition... "How do you explain 'brain-dead' to a person who does not understand what a 'brain' is?"*  Healthcare workers-Knowledge and Competence  Ethical decision making: *All of them underlined that it was, and had to be, the most senior doctor or registrar who took ethically difficult decisions. Many found that they had no influence on this matter, but all except two had in some way been involved in decisions concerning withdrawal. Some of them focused primarily on how to keep the baby alive, while others were more concerned about when to apply or withdraw treatment. In the latter cases, emphasis was laid on the available medical alternatives, on data concerning survival and treatment results for similar children at their unit, the resources available, the law against euthanasia, and the parents’ situation....All the informants, except one, claimed not to be influenced by their religious beliefs. Several referred to God as the ultimate decision maker, and regarded the relevant decisions as a practical rather than an ethical challenge... Many also said that it was easier for them to handle these situations than for their Western colleagues, because they were used to seeing children die.*  *Whether or not to involve the parents, or to seek their co-operation in decision making, was a frequently mentioned dilemma, although not all informants were convinced that parents ought to be involved in decisions concerning withdrawal of treatment. One problem experienced was that some parents perceived the doctors as gods. These parents tended to agree to almost everything that was recommend to them and refused to participate in decision making. Many of the doctors said they felt uncomfortable in such situations. They did not want to be responsible for life and death decisions. Three doctors explained how they left the final decision to the parents, so as to avoid the sense of guilt associated with deciding on someone else’s life.*  *Almost all the doctors at both hospitals mentioned the gender problem in India as a dilemma in their work. Several had experienced the conflict themselves. They had noted, for example, that parents found it easier to consent to the withdrawal of treatment, or that there was less interest in buying medicine, when the baby was a girl... Lack of understanding, lack of resources to take care of a daughter or other children, fear of high dowries, and the cultural stigma of having a girl were some of the factors mentioned.*  *Some reported that when they knew the baby would die anyway, they did not consider the withdrawal of treatment as an option...Babies in critical conditions provide learning opportunities, which doctors feel they can make use of so as to perform better next time or when the economy improves. This perspective was reported both by senior registrars and by postgraduate students.*  Relationships with others  Effects on family and friends: *In many cases of terminated treatment, the doctor had felt uneasy about continuing treatment for babies with uncertain outcomes, when they knew that the parents could not afford to provide lifelong care. Responsibility for a family’s future was experienced as the worst dilemma when deciding whether or not to withdraw treatment. Where the doctors managed to save a premature or critically sick child, they were aware that its survival was likely to create problems for the parents, requiring money for medicine, hospital visits and care. They also considered the risk of incurring lifelong expenses for the parents if the child failed to marry because of a handicap. This would also affect other children in the family...*  *Those doctors who were concerned about the future of children who survive talked about lack of opportunities with respect to the family rather than the child. Several doctors had experienced mothers at follow-up consultations complaining about the fact that the doctor had saved the child, as there was so much trouble with it. One doctor said that in such situations he could only comfort the mother, but he and others who reported similar experiences felt responsible, even though they had only been doing their jobs.*  *"Who am I, a third person, to decide? His brother and sister might not go to school because of this.”*  *Some underlined that these decisions are different in India compared to Western countries, where guidelines are based on the assumption that the parents or society will be able to cope with the longterm consequences. Many of them found that their own guidelines did not take their local social and economic realities sufficiently into account.*  Survival  Survival without disability; *"India needs productive people; we are creating the disabled."*  *Others said they were aware of the burdens that might result for society by treating high-risk babies. None had withdrawn treatment on these grounds, yet it was a perspective that concerned them when deciding to continue treatment.*  Other  Healthcare resources: *Limited resources were a common problem for our informants. Part of the dilemma was not being able to offer the best treatment to all, regardless of how many patients there were at the unit. Another aspect was the frustration of not being able to follow the national guidelines or the recommendations in Western textbooks and journal articles. Some had had the experience of having to withdraw, or of helping to withdraw, ventilation from a child in order to give the ventilator to another child with a better prognosis. In these cases it was known that the first child would most probably die anyway, or would only survive with a severe handicap even if given the best treatment available.*  *No one mentioned over-treatment as a dilemma in Indian NICUs, although many had a strong sense of wasting resources when critically ill babies were given costly, and highly intensive, care. They experienced this situation as frustrating, and some pointed out that 50% of the unit’s resources were used on the 1–2% who were most sick, and whom they hardly managed to save anyway.*  *Limited resources for treatment were also a consequence of the poor economy of parents. The doctors reported how they had to balance the need for expensive medication and equipment (which the parents must buy for their child) with their perception of the parents’ economy. They wanted to spare the parents the humiliation of having to refuse the best treatment or of not being able to act on recommendations to go to a private hospital, options which would probably result in economic ruin or the starvation of other children at home. Many of the doctors described personally trying to help out in such cases, by looking for funding or giving the parents money or medicine. Some also reported persuading the better off parents to buy things other babies could also benefit from. Another dilemma mentioned was that some children were transferred from private hospitals because the parents could no longer pay for treatment there* |
| Miracle (2004) | Gastrointestinal Breastfeeding: *"They told me how breast milk was good, helped the babies to grow and help fight infections. Maybe because I had a small baby, and I feel that it helps mature his digestive system. And he's so tiny and not supposed to be digesting food this way, so I feel it's probably that much easier on his system-what nature meant for him to eat."*  *"I'm glad I'm doing it. I feed good that I'm doing it. I feel good about myself. It makes me feel good about myself that I'm doing it for him."*  *"I didn't feel forced at all. They tell you all the facts about it, how great it is for the baby, how much healthier it is, and they let me know how much better it is for the baby than formula. So I'm happy they do that. No, I didn't feel forced. I needed that push in the right direction and just to see the outcome and to hear people say, "Wow, that is good,"and "Wow, look how good you did." To see all that and hear all that, it makes you feel good."*  *"Also, they've been telling me babies cannot digest the formula like the breastmilk."* Infection Prevention of infections: *"The nurse was talking to me about how breast milk fights off infections"*  *"She told me of the fact that they were earlier and didn't have the capabilities of fighting off infections, and they needed that from the milk, from me."*  *"There is a mother [peer counselor] that brings her baby with her to Milk Club, and she said her baby didn't have any colds or ear infections that 1st year. That story helped me. It's a success story."*  *"The neonatologist came in and said that it was to their benefit to grow quicker, they could tolerate it more, their immune system-that's what is best for them. That's all she needed to say, it was decided by sundown."*  Healthcare workers-Communication Developing a caring relationship: *"So it makes me feel comfortable, even with the doctors. I think it was Dr. Wallace, she said (while I was pumping), "How's it going?" And it makes me feel good that we talk to each other like family."* Normality Normality: *"And he's so tiny and not supposed to be digesting food this way, so I feel it's probably that much easier on his system-what nature meant for him to eat."* Growth Growth: *"The doctors were explaining to me that my breast milk would help her gain weight."*  *"The neonatologist came in and said that it was to their benefit to grow quicker,"* |
| Moyer (2010) | Healthcare utilisation Inappropriate treatments: *Community providers (including PCPs, home health nurses, pharmacists, and community emergency rooms) may lack the required knowledge and skills to manage complex infants, leading to suboptimal office-based care and perceived overutilisation of the emergency system.* Healthcare workers-Communication Communication about discharge: *Parents/care givers may be inadequately prepared for home care and management of fragile neonates due to a lack of consistent and early communication between parents and NICU staff and lack of coordinated educational and social services support programmes prior to discharge.* Healthcare workers-Knowledge and Competence Lack of consistency: *Healthcare providers (attending physicians, consulting physicians and other healthcare providers) in the NICU tend to act in isolation, which results in lack of a standardised, coordinated and comprehensive plan of care. Community providers (including PCPs, home health nurses, pharmacists, and community emergency rooms) may lack the required knowledge and skills to manage complex infants, leading to suboptimal office-based care and perceived overutilisation of the emergency system.*  Healthcare professional competence: *Community providers (including PCPs, home health nurses, pharmacists, and community emergency rooms) may lack the required knowledge and skills to manage complex infants,* |
| Orfali (2004) | Gastrointestinal  Oral feeding: *"I think his quality of life will be poor; he can’t even eat. He will be profoundly retarded”*  Neurological  Significant IVH: *‘‘Although she has a grade IV bleed, the resident says that she moves and looks around, and he thinks the odds are quite good.’’*  *“The neurological outcome is never known (when the baby has a bleed). Ethically, if the baby is severely compromised, what do you do?I’ve talked to people who have seen kids with grade IV bleeds who come out fine. It makes it hard to make the decision”*  *“She has an IVH grade IV, but she moves quite well and things might turn up better then expected”*  Development-Special senses  Hearing impairment: *"This kid is in really bad condition. I told the parents that he will never be able to see, to hear and I will get more data to show them how bad things are. Because in this particular case, there is no doubt!"*  Visual impairment: *"This kid is in really bad condition. I told the parents that he will never be able to see, to hear and I will get more data to show them how bad things are. Because in this particular case, there is no doubt!"*  Healthcare workers-Knowledge and Competence  Ethical decision making: *Medical authority was exercised in limiting available options. As an American pediatrician commented: ‘‘I think they [parents] have played a part in dealing with the decision as much as we [doctors] have played a part in fixing them.’’*  Survival  Survival with disability: *"I think his quality of life will be poor; he can’t even eat. He will be profoundly retarded”*  *“At 2 cm, we know that the outcome is very dismal, that the kid will be severely handicapped”*  Survival without disability: *"I have always thought we should limit treatment instead of giving (the parents) a handicapped child. Especially as we know very well what happens on the long run for a couple. Statistically, there are many divorces and things like that"* |
| Rasmussen (2016) | Neurological  Brain injury (not further specified):*‘‘Brain injury is laden with a lot more emotions and moral concerns for sure . . . neurologic or neurodevelopmental impairment in the long term is much more associated with a negative outcome compared to compromise of another organ that can be supported with medical therapies in the long term.’’*  Healthcare workers-Communication  Communicating in challenging settings:*“I see prognosis affecting families medical decisions . . . they do their best. I mean, it’s not easy but they do their best to factor in their values and their lives. And they can’t really speak for this individual who’s never spoken to them before. But I think they do their best. And it’s hard. It’s a hard situation to be in.”*  Other  Overall health state: *“By neurological prognosis I mean that we try to think of the whole life consequence. We talk about, you know, strength and cognitive capacity, but also life and communication and feeing yourself and getting around.”* |
| Reyna (2006) | Gastrointestinal Choking during feeding *"the only concern I have is, I don’t want them to choke. I’m fearful of choking."* Feeding difficulties: *The first theme was interpreting infant behaviors (Table 2). This theme included recognizing infant feeding readiness, hunger, and satiation cues. Feeding readiness included mothers’ consideration of their infants’ motor and behavior state organization before a feeding, particularly the infants’ level of alertness. Hunger cues were the behaviors mothers used to describe hunger in their infants. Similarly, satiation cues were cues recognized by mothers to indicate the infants had fed “enough” and was satisfied.*  Feeding practices: *The second theme was managing the evolving feeding process (Table 2). This involved the mother’s evaluation of her infant’s skill at bottle feeding, meeting the demands of the feeding schedule, and controlling her time and resources. This theme included the mother’s perception of how successful she was at balancing her infant’s frequent feedings with everyday demands such as grocery shopping, doing laundry, and caring for other children. In addition, a mother’s previous experience with bottle feeding and how she used the support of other family members to manage this process was part of this theme.*  *Realizing knowledge gaps was the third theme (Table 2). It involved understanding the nutritional needs of their infants, such as when to advance the feeding volume and when to omit a feeding. It included mothers’ feelings of how prepared they felt at discharge and the reality of caring for the infant at home. Another a part of this theme was what the mother had learned from her initial experiences and how she managed anxiety about caring for her infant after discharge.*  *Before discharge, all infants in this study were on scheduled feedings with a prescribed feeding volume. Routine discharge instructions for the mother included advancing the infant’s feeds as tolerated to an “ad libitum” schedule. Mothers had difficulty understanding these instructions and seemed hesitant to liberalize their infant’s intake after discharge. They worried both about giving too much formula at a feeding or about missing a feeding...Despite concerns regarding feeding volume, 26% (8) of the infants were noted to have “spitting through their nose.” This typically occurred as the feeding volume was advanced. None of the mothers recognized this as a problem or associated it with overfeeding or difficulty with suck-swallow-breathe coordination.'* Parental Support Parental competence: *The second theme was managing the evolving feeding process (Table 2). This involved the mother’s evaluation of her infant’s skill at bottle feeding, meeting the demands of the feeding schedule, and controlling her time and resources. This theme included the mother’s perception of how successful she was at balancing her infant’s frequent feedings with everyday demands such as grocery shopping, doing laundry, and caring for other children. In addition, a mother’s previous experience with bottle feeding and how she used the support of other family members to manage this process was part of this theme*  *All of the mothers identified having the support of a husband, a significant other, or a family member(s); however, the mothers were the infants’ primary care-givers. Mothers reported that family members were uncomfortable feeding their infants. Furthermore, mothers also reported that they were uncomfortable having other people feed their infants.* Healthcare workers-Communication Communicating information effectively: *The information provided in the NICU regarding feedings and well-baby care was considered appropriate and helpful. Mothers who were able to visit frequently while their infants were hospitalized had many opportunities to feed their infants before discharge and verbalized more comfort with the feeding process once home.* |
| Rohsiswatmo (2014) | Infection Sepsis: *Decrease blood stream infections Selected as key performance indicator* Healthcare workers-Knowledge and Competence Healthcare professional competence: *Processes thought to improve infection rates in NICU neonates: 'Education-socialization of employees; competence test; placement of employees according to competence; employee career path; renumeration'* Survival Survival: *Decrease mortality rate selected as key performance indicator*  Other  Duration of admission: *Decrease length of stay selected as key performance indicator* |
| Saigal (1999) | Development-Gross motor Ability to walk: *Needs assistance to walk (from equipment/person)* Development-Fine motor Difficulties with activities of daily living: *Needs assistance/to use special equipment to eat, dress/bathe or use the toilet* Development-Special senses Hearing impairment:  *deaf,*  Visual impairment: *Blind* Development-Speech and Social Language disorders: *Unable to talk* Psychiatric Mood disorders: *Occasionally fretful, angry, irritable, anxious, depressed, or experiencing 'night terrors'*  Need for educational support:  *Learns schoolwork very slowly and needs special help* Pain Pain: *Sometimes has pain* Normality Normality: *Happy and not worried most of the time* Other Overall health state: *It appears that HPs* [Healthcare Professional] *and parents view the mild to moderately disabled health states similarly, but parents were more accepting of the severely disabled health states than HPs.* |
| Saunders (2003) | Gastrointestinal Breastfeeding: *Benchmarking questions identified: how do you encourage breastfeeding on your unit?*  Parental Support Parental involvement: *Benchmarking questions identified: Does your unit welcome parents 24 hours/day? How are families encouraged and supported to participate in routine care of the infant? What provisions for privacy around the bedside do you offer? Are families encouraged to make their infants immediate environment as homelike as possible?*  Healthcare workers-Communication Communication about discharge: *Benchmarking questions identified: Does your hospital provide teaching support for outside personnel? How ready do you feel you are to care for your infant after discharge from the hospital?* Pain Pain management: *Benchmarking questions identified: What tools are used for pain assessment?* Suffering Suffering: *Benchmarking questions identified; Are ventilated infants routinely sedated?* Normality Normality: *Benchmarking questions identified: During your infant's hospital stay, how often did you feel like a parent? Are families encouraged to make their infants immediate environment as homelike as possible?* |
| Sawyer (2014) | Healthcare workers-Communication Communicating information effectively: *I was given all the information I needed Encouragement and reassurance,* Healthcare workers-Knowledge and Competence Healthcare professionals behaviour: *Staff professionalism and empathy The staff put me at ease The staff made me feel cared for as an individual*  *Staff being confident and in control Staff being calm in a crisis* |
| Schneiderman (1997) | Suffering Suffering: *Providing futile life-sustaining treatments "ridiculous," "dumb," "inappropriate," "insane," "outrageous," "frightening," "a teaching example of futility," "an example of what's wrong with medicine today,"* |
| Sharek (2003) | Respiratory Chronic Lung Disease: *Outcomes identified by CLD focus group: Chronic Lung Disease rate Oxygen requirement at discharge home Pneumothorax rate*  Oxygen dependence: *Outcomes identified by CLD focus group: Chronic Lung Disease rate Oxygen requirement at discharge home Pneumothorax rate*  Pneumothorax: *Outcomes identified by CLD focus group: Chronic Lung Disease rate Oxygen requirement at discharge home Pneumothorax rate* Development-Special senses Retinopathy of Prematurity: *Outcomes identified by CLD focus group: ROP rates* |
| Short (2003) | Development-Gross motor Need for physical therapy: *significantly more BPD children received...occupational, and physical therapy* Development-Speech and Social Language disorders: *significantly more BPD children received speech-language therapy* Parental Support Support from healthcare professionals: *Parents of children with a history of BPD reported that more of their children had occupational and physical therapy, including children who were without neurologic complications. The prolonged hospitalizations experienced by children with BPD and the frequent interactions of families with medical personnel may result in increased access and opportunities for services for parents of children with BPD.* Healthcare utilisation Frequent readmissions: *Parents of children with a history of BPD reported that more of their children had occupational and physical therapy, including children who were without neurologic complications. The prolonged hospitalizations experienced by children with BPD and the frequent interactions of families with medical personnel may result in increased access and opportunities for services for parents of children with BPD.* Psychiatric Need for educational support; *BPD children were more likely to be enrolled in lower grades than VLBW or term children. lacement in special education ser-vices, regardless of type, differentiated the groups, with 54% of the BPD, 37% of the VLBW, and 25% of the term children participating in special education.* |
| Sisk (2010) | Gastrointestinal Choice of milk for feeding: *Universally, mothers perceived the pumping schedule as a huge weight on their shoulders because of the time it required. Mothers often spoke of the conflict between taking care of themselves and their other responsibilities and the need to pump...Mothers often delayed pumping to be at their infant’s bedside for medical rounds, and it was unpredictable when those rounds would occur. Pumping at the bedside was an option; however, many mothers or their spouses were uncomfortable with this.*  *Attitudes toward milk expression influenced mothers’ willingness to pump.*  *Anticipation of breastfeeding provided support for milk expression. Mothers who planned to breastfeed as opposed to exclusively bottle feed their breast milk viewed pumping as something they had to do.* Parental Support Support from fathers: *Fathers ranged from being very supportive, “[we’re] in this together,” to being deterrents or completely absent.*  Parental ability to work: *Seven mothers returned to work within 3 weeks after delivery; none had paid maternity leave. They liked being back at work because it helped occupy their minds, but they reported being exhausted*  Coping with maternal illness: *Physical and mental challenges. The majority (59%) of mothers experienced a pregnancy-related medical complication, and almost half (44%) were treated with magnesium sulfate, a medication given to treat preterm labor and prevent seizures in severe preeclampsia. Mothers who received this medication stated that they had difficulty comprehending the pumping instructions or did not feel well enough to pump....*  *Mothers, especially those who were delivered by cesarean section, complained of extreme fatigue and desire for sleep in the first few days after delivery, and this interfered with pumping frequency and their ability to remember the pumping instructions*  *...Feeling anxious and stressed were common complaints during the immediate postpartum period. Lack of preparation for the preterm delivery and disruption of plans as well as concern for their infant’s health, job, and finances distracted mothers from pumping frequently. For some mothers, however, anxiety regarding their infants’ health motivated them to frequently pump.*  Barriers to parental involvement: *Lack of privacy was identified as a barrier to establishing a pumping schedule. Frequent interruptions and the flow of hospital staff, family, and friends interfered with their ability to learn to use the breast pump, relax during pumping, and pump frequently. Mothers disliked telling their visitors to leave, and they disliked pumping in front of them. Mothers stated that hospital staff members often were unable or unwilling to provide the amount of privacy they desired.*  *-availability of hospital grade breast pump helped transition from BF in hosp to BF at home*  *-pumping at home without stimulus of infant was more difficult*  *-transport to hospital to feed infants difficult*  Support from family and friends: *All mothers identified supportive people in their social networks. These included family members, friends, church members, and hospital staff. Practical assistance with chores (ie, child care for older children, meal preparation, and house cleaning) was critical to their success. The recognition and praise mothers received also motivated them to continue pumping.*  *-mothers relied on rides to hospital from family and friends, which sometimes worked, sometimes didn't in terms of feeding*  Balancing caring for themselves and their baby: *Universally, mothers perceived the pumping schedule as a huge weight on their shoulders because of the time it required. Mothers often spoke of the conflict between taking care of themselves and their other responsibilities and the need to pump...Mothers often delayed pumping to be at their infant’s bedside for medical rounds, and it was unpredictable when those rounds would occur. Pumping at the bedside was an option; however, many mothers or their spouses were uncomfortable with this.*  *Anticipation of breastfeeding provided support for milk expression. Mothers who planned to breastfeed as opposed to exclusively bottle feed their breast milk viewed pumping as something they had to do* |
| Smith (2006) | Gastrointestinal Breastfeeding: *"Because I think I understood the importance of what my wife was trying to do. It was extremely important for our babies, who are very fragile and sick, to receive breast milk."* Parental Support Support from fathers; *"I helped her as much as I could. I cleaned and carried the pump when necessary. I cleaned the pump kit when she was finished pumping, labelled the bottles, cleaned the bottles, transported the milk to the hospital, assisted with storage, and assisted her in whatever way I could while she was pumping."* |
| Snowdon (1997) | Parental Support Support from faith: *"We regard Philippa as a gift from God and so when she was ill, you know we prayed that--we obviously prayed that we wanted to keep her but we knew that God would do what was best for her and for us and so I think although it was very worrying and very traumatic, there was a sort of peace underneath that actually"* Healthcare workers-Communication Communicating in challenging settings: *"Nobody can play God. You're making a decision on somebody's life and they don't really know so the computer decides and that's the best way because you're not blaming anybody then are you? You don't really want the onus on yourself really but I suppose the medical profession can't.., keep the onus on themselves"*  Survival  Survival: *" I think any parent wants to try anything, you know, they don't sit down and think it's research, there could be side effects or there could be abnormalities that might come up. I think as parents...your first instinct is to save your baby"*  *"I suppose what they're saying is that er if at the end of the day conventional really really really isn't going to work for him then that would be absolutely ideal because.., the chances are maybe he would be picked"*  *"I suppose trials have to be a bit heartless, but you'd think that when the baby looks like they're dying, you'd think they'd just say... "Oh hell" you know "'let's try the ECMO, see if it saves this baby" but with that sort of a trial they can't do that can they? They have to say, "Well look, this baby looks like it's dying but l'm sorry it's getting conventional treatment and that"*  *"your first instinct is to save your baby"*  *"we obviously prayed that we wanted to keep her "*  Other  Inclusion in research: *"I feel desperately sorry for parents who, you know, were turned down particularly if their child doesn't live. I think it would be hard but I can see that it is necessary in case...the research shows that ECMO is actually detrimental to children"*  Iatrogenic harm: *"I suppose trials have to be a bit heartless, but you'd think that when the baby looks like they're dying, you'd think they'd just say... "Oh hell" you know "'let's try the ECMO, see if it saves this baby" but with that sort of a trial they can't do that can they? They have to say, "Well look, this baby looks like it's dying but l'm sorry it's getting conventional treatment and that"* |
| Snowdon (2014) | Parental Support Coping with maternal illness: *All women who had a CS, some of whom needed additional interventions, such as Laura, who underwent surgical removal of her placenta, and Caitlin, who had reparative bowel surgery after damage caused during delivery, had to deal with the aftermath of their birth and surgery and their initial experiences in the NICU at the same time...Where women were debilitated postnatally this could limit their contact with their babies. Although a number were taken to the NICU in a wheelchair or their bed, when babies were moved to another hospital it was not always possible for the women to accompany them.*  *The mode and pace of delivery also impacted upon the men. Some of the men did not arrive at the hospital in time for the birth, as events could be unexpected and fast moving. When women underwent an emergency CS, men could be excluded from the birth, a potentially isolating and anxiety-provoking experience.*  Parental involvement: *Once babies were delivered and taken to the NICU, there was usually a lag before parents were able to visit. Some of the parents were frustrated at not being allowed to go to the NICU with their baby and the wait for news or to be allowed to visit could seem interminable.* Healthcare workers-Communication Communicating in challenging settings: *When they arrived at hospital in preterm labour, or were admitted for observation and bed rest, some had difficult conversations with clinical staff about the implications of their gestational stage for decisions about care. NICUs commonly set boundaries around the care that they offer, as their facilities determine their lower thresholds for care...Parents who were around the 23-/24-week cusp not only had to contend with the worry of whether or not their babies would survive and in what condition, but also they were faced with the possibility that intensive care would not be mobilised in their case. At the borderline of viability they had to wait to see whether their baby attempted to breathe to find out whether or not care would be initiated. This sense of ambiguity could be heightened when women were given antenatal steroids to help to mature their babies’ lungs and improve their chances of survival, while also being faced with the possibility of no active care being offered postnatally.*  Keeping parents informed: *Once babies were delivered and taken to the NICU, there was usually a lag before parents were able to visit. Some of the parents were frustrated at not being allowed to go to the NICU with their baby and the wait for news or to be allowed to visit could seem interminable.'* Survival Survival: *this trial spoke directly to their most pressing concerns; the threat to survival and the possibility of disability*  *The overwhelming sense from the parents interviewed for BRACELET was that at the time they wanted their babies to be cared for and for everything possible to be done. A minority of the parents who were facing extremely preterm birth came to their own decisions about care, which pre-empted NICU policy on admission.*  Survival without disability: *this trial spoke directly to their most pressing concerns; the threat to survival and the possibility of disability*  Other  Inclusion in research: *Parents interest in a trial appeared to develop over time as their bereavement receded. Although most had experienced little contact with a trial over the years, parents were often interested in the research, and some would have liked more contact and information than they actually received. Trial communications were valued as a source of information, as an acknowledgement of loss and the contribution to research, as a connection back to their baby and a form of commemoration. Some parents had kept trial paperwork in their baby's memory box.*  *Danielle’s experiences of antenatal recruitment to BOOST-II UK were strikingly similar to Beverley’s but they described different positions on altruism and enrolment. For Danielle her sense of altruism and the potential benefits for others was sustained throughout her association with the trial, but for Beverley it was more transient and dissipated somewhat once she was able to engage with the reality of her daughter’s initial survival, condition and needs.*  *Discussion of the trial therefore required emotionally strained parents and physically debilitated mothers to engage not only with the information and choice offered to them in relation to cooling and the trial, but also confronted them at an early stage in their experience with the possibility of brain damage and disability for their newborn. Parents still reeling from delivery had to think about both the immediate and the long-term future.*  *Clearly parents come to neonatal intensive care via a range of different clinical pathways. Their reactions to the environment and to their babies are similarly varied and highly personal, bringing together emotions from recent and sometimes more distant events. It is against this background of threat, initial survival and initiation of care that they made their decisions about trial participation.*  *Ivan mentioned INIS right in the middle of this difficult time, as a direct response to the threat of serious infection for their baby. This places the trial into a therapeutic framework, and parents spoke of their hopes that INIS would make a difference, but by this point the gravity of their situation was sometimes clear and the trial could seem like a long shot rather than a magic bullet.*  *The offer of the trial, however, was disassociated from the crisis that the parents had experienced, partly because of the flexibility over timing and partly because the intervention related to a routine management of care.*  *Three main models of the potential effect of a trial could be identified in the data (Figure 16), which were characterised in the analysis as: -might help, won’t harm -might help, might harm -won’t harm, might help others'* |
| Squitieri (2013) | Development-Fine motor Difficulties with activities of daily living: *“A lot of times I have to put myself in certain positions to do things, like opening a jar- I have to squeeze it in between my arms instead of gripping it with my hand. And when I type, I have to position my fingers in a different way on the keyboard so I can reach the keys.”*  *Most adolescents were extremely functional and able to accomplish basic activities of daily living due to the high degree of learned compensation. Many females reported difficulty grooming their hair* Parental Support Support from family and friends: *“My mother in law and my mother both would watch my older daughter that first year quite a bit while I would take my daughter to therapy”* Healthcare utilisation Frequent appointments: *Many parents commented on the time commitment of formal therapy visits. Depending on the ease of access to a nearby therapist, many adolescents would miss school and their parent would need to take off from work.* Relationships with others Peer acceptance: *Peer acceptance and fear of being teased or excluded from social groups was a prominent theme throughout our interviews. In addition, many adolescents commented on the importance and positive influence of obtaining support from other adolescents with NBPP through various camps, therapy groups, or online.*  Psychological coping: *Despite their impairments, most adolescents had a positive view of themselves overall. Negative coping mechanisms were more common among younger adolescents and adolescents with greater levels of impairment. Older adolescents generally dealt with their impairments in a constructive manner either through learned compensation techniques, acceptance, or anticipation and alteration of activities if they believed their impairment would limit them in any way.*  Family resources: *All participants in our study had some form of insurance coverage. However, some parents reported frustrations regarding the cumbersome process of talking with insurers to confirm coverage or extended therapy visits.*  Effects on family and friends: *Almost all parents acknowledged the emotional adjustment of other family members in response to raising a child with physical impairment. Many relied on the help of extended family. Few parents reported negative effects on parental/sibling relationships.* Pain Chronic pain: *Occasionally some patients or their parents reported hypersensitivity or mild discomfort with therapy exercises or prolonged activity. No patients reported significant pain that impaired daily living. “She occasionally complains of pain in her shoulder area. Maybe 1-2 times per month."* Suffering Comfort: *Patient priorities: To sleep more comfortably* Normality Normality: *“My arm used to make me mad and sad because I couldn’t be like everybody else… ‘why can’t I raise my hand like this?’ and stuff like that."* Other Physical appearance: *“I got teased in the fourth grade. They’d call me ‘overblown bicep’ and ‘fat arm.’ Sometimes I would get really self-conscious and discouraged and think that my arm looks weird and that people are staring at me.”*  *“I don’t like wearing half sleeves or quarter length sleeves because it looks like a long sleeve on my right (palsy) arm but a quarter sleeve on my left (non-palsy) arm.”*  *Almost all adolescents and parents commented on aesthetics and body image regarding arm position, length/size discrepancies, and clothing restrictions.* |
| Stevens (2014) | Gastrointestinal Oral feeding: *"(The) very first time (feeding the baby) was just great, to tell you the truth. It was an experience, just like, ‘Wow’…it was just looking into him and just like, ‘wow, look what we made, ’know?’ It was just crazy…it was just wonderful.”*  Relationships with others Bonding with parents: *"It was pretty awesome [laughing] because (his daughter) had been in an incubator for a while and we couldn’t hold her and then we actually got to hold her. Holding a baby is something that I’ve always wanted so to me it was priceless"* Normality Normality: *"It was pretty awesome [laughing] because (his daughter) had been in an incubator for a while and we couldn’t hold her and then we actually got to hold her. Holding a baby is something that I’ve always wanted so to me it was priceless"* Growth Growth: *"you want to be sure that they take everything so that they’re gaining weight adequately"* |
| Thoren (2013) | Parental Support Online support: *"This group is for parents who have experienced the miracle of having a preemie. Let's share our stories to help each other through the difficult times of NICU and support each other through the even more challenging task of raising a preemie."* |
| Thoyre (2000) | Gastrointestinal Oral feeding: *"Just get the bottle in him. That’s all. "* |
| Turrill (2003) |  |
| Turrill (2003) | Neurological Significant IVH: *"By then.. em.. I think we're coming off the big risk of nasty big haemorrhages as well, unless something seriously goes wrong with them for some obscure reason."* Development-Special senses Visual impairment: *Children needing to wear hearing aids or glasses* Hearing impairment: *Children needing to wear hearing aids or glasses*  Parental Support Parental involvement: *Reduced parental contact was cited as a characteristic of increasing risk for alterations to development...Support of the family unit in hospital is essential to the future wellbeing of that family. However what that part of care has to play in the long term development outcomes of newborns who receive intensive care is unknown. Although parental support is a vital aspect of their role, relating this to developmental outcomes is misguided and again, highlights the lack of a common knowledge base supporting practice.* Healthcare workers-Communication Communication with parents: *"When you're talking to parents while you're doing cares and everything, you're not really talking to them,... you're having a vague conversation across the room"*  *Communication between professionals p.33 'Beyond the immediate admission, relationships betwen nurses and doctors and their differing roles and responsibilities appeared at times in conflict, with little team approach to care.'* Healthcare workers-Knowledge and Competence Healthcare professional competence: *nurses' lack of knowledge of short and long term LBW infant outcomes: lack of evidence-based guidelines to support nurses use; too lengthy; info not seen as important or relevant to their role; lack of time; no formal channels to disseminate information*  *nurses lack understanding that 80% of LBW infants develop normally, and what 'normally' is, means that they don't feel able to foster that normality in NICU care*  *'The physical stability of babies must remain the nurses first priority when considering optimal brain function in relation to future outcomes. However, the importance of controlling the neonatal environment to increase stability appeared to have been lost. The constant intensiveness of the working environment, while recognised as not normal, has been taken for granted.*  *The quality of physical care given to a VLBW baby in the first 24 hours is vital to his or her survival and future development. The responses from nurses in clinical and managerial positions showed they were acutely aware of this priority...four [higher grade] nurses had changed priorities [in the scenario], with parents gaining importance over time.*  *Nurses recognized that their limited knowledge, in-service education and research were seen to affect their decision-making abilities, confidence and consequent autonomy. The current strategy for continuing education is affected by the fact that nurses are rarely able to leave the cot-side to take part in meetings, seminars or reflective groups due to the pressure of clinical commitments. Also access to a clinical academic neonatal pathway is not currently available within the unit's education contracts'*  Consistency of decisions: *Beyond the immediate admission, relationships between nurses and doctors and their differing roles and responsibilities appeared at times in conflict, with little team approach to care.* Normality Normality: *nurses lack understanding that 80% of LBW infants develop normally, and what 'normally' is, means that they don't feel able to foster that normality in NICU care*  *With the emphasis on normal development being lacking in their knowledge base, the effects of interventions appear weighted towards a failure to achieve the ideal at the start. The level of responsibility the nurses felt they held towards the baby's future, coupled with this high risk of failure, posed an unrealistic challenge.* Growth Growth: *nutrition relates to growth and thriving*  Other  Healthcare resources: *The level of resources was thought to have the greatest impact on the nurses' ability to carry out their role, in particular the nursing resource.*  *'When considering what aspects of service impacted on their ability to carry out the care they ideally wanted to give, limited available time was important. With a workforce of only 62% of the regionally-funded QIS level, the constant pressure had a great impact on managing priorities for care.* |
| van Zuuren (2006) | Development-Gross motor Issues of development and motor skills: *"will it stay dependent on the care of oth-ers for the rest of his life, will it remain spastic, will it be wheelchair-bound?"* Development-Speech and Social Language disorders: *"Will the child be able to communicate in the future"* Healthcare workers-Communication Communicating in challenging settings: *Uncertainty, in particular unpredictability of the outcome in the child, is seen as a complicating factor in end-of-life decisions, and, to a lesser extent, in the problem of medical damage.... Long-term follow up is advocated in order to reduce this uncertainty.*  Suffering Suffering: *It was the nurses who most explicitly pointed to the direct suffering that treatment devices often impose on the child and who sometimes resented the parents’ or doctor’s wish to prolong treatment. For these nurses, there is often an imbalance between two important ethical principles: doing well and avoiding harm....So, clear disadvantages of doing well (treatment) are the immediate suffering of the newborn and the possible damage in the future.* Other Survival with disability: *"The greatest dilemma has to do with the handicaps we induce with our treatment." "A child with mild handicaps can have a good life. A child that hardly makes any developmental progress and that will be dependent on others all along .... (unfinished)."* |
| Vandenbussche (1999) | Neurological Neurological symptoms *-Mothers' and obstetricians' valuing of 4 types of infant outcome: -healthy infant -transient neurologic symptoms -permanent neurologic symptoms -neonatal death* Survival Survival:  *Pregnant women and mothers valued an infant with permanent handicap significantly higher than neonatal death, and this choice was irrespective of the type of birth (all p values < 0.01). Obstetricians, on the other hand, awarded lower, albeit not statistically different median values to permanent handicap than to death.*  Survival with disability: *Pregnant women and mothers valued an infant with permanent handicap significantly higher than neonatal death, and this choice was irrespective of the type of birth (all p values < 0.01). Obstetricians, on the other hand, awarded lower, albeit not statistically different median values to permanent handicap than to death.* |
| Votteler (2005) | Genitourinary Urological disorders: *Persistent urological problems concern twin A's mother as to whether he will be accepted or teased by his peers when he is older.*  Surgical Need for multiple operations: *Three twins required scoliosis correction in later child-hood. Neurosurgical problems have been significant in both 1988 pygopagus sets, requiring long-term evaluation.* Development-Gross motor Ability to undertake sport: *They report feeling self-conscious of their appearance and avoid athletic activity, which calls attention to their physique. She cannot participate in physical education because of orthopedic and gait problems.* Development-Special senses Hearing impairment: *Social communication is difficult because of his hearing and speech problems, and he is described as having a few friends and no experience in dating.* Development-Speech and Social Language disorders:  *Social communication is difficult because of his hearing and speech problems, and he is described as having a few friends and no experience in dating.*  Social difficulties: *Social communication is difficult because of his hearing and speech problems, and he is described as having a few friends and no experience in dating.* Healthcare utilisation Need for frequent treatments: *Three twins required scoliosis correction in later child-hood. Neurosurgical problems have been significant in both 1988 pygopagus sets, requiring long-term evaluation.* Psychiatric Need for educational support: *The patient is at an age-appropriate grade level but attends resource classes in math and achieves only average grades in other areas. The girls are at an age-appropriate grade level and earn mostly As with some Bs. Both aspire to pursue college and graduate school education.*  Psychiatric disorder: *The other boy has been diagnosed with autism and bipolar disorder and is receiving care in psychiatry, neurology, occupational therapy, and speech. The mother is very focused on the boys' physical and emotional symptoms*  Normality Normality: *Her parents consistently emphasized her normalcy and need to gain independence. The parents had a second child to 'normalize' life for their surviving son.*  Survival  Survival: *His mother reports a continuing sense of sadness over the neonatal death of the patient's twin. A recent first-time exposure to a photograph of this deceased infant was described as extremely meaningful and comforting. His parents report significant sorrow over the loss of twin B and the mother eventually went through counseling to work through these grief issues. Twin A's young parents are still grieving the death of twin B and are still questioning their decision making in terms of that twin's care.*  Other Physical appearance: *They report feeling self-conscious of their appearance and avoid athletic activity, which calls attention to their physique.* |
| Wielenga (2015) | Respiratory Mechanical ventilation: *Research priorities identified: ...(6) respiratory and ventilation;* Gastrointestinal Feeding practices: *Research priorities identified: Identifying best practices in enteral feeding Identifying best practices breast feeding Improving nutrition in preterm and sick term infants* Neurological Neurological care: *Research priorities identified: Identifying the best care practices for infants with neurological problems* Infection Sepsis: *Research priorities identified: Evaluating infection prevention strategies Identifying and evaluating interventions to monitor and reduce hospital-associated infections Evaluating sepsis management and care to improve outcomes* Skin Skin care: *Research priorities identified: Identifying and evaluating interventions to improve skin and wound care in neonates* Surgical Care for surgical babies: *Research priorities identified: Identifying the best care practices for surgical infants* Parental Support Parental involvement: *Research priorities identified: Identifying strategies to support the needs of parents and family members Identifying and evaluating strategies to support parental attachment Identifying best practices for the implementation of family centred care Evaluating the role and involvement of parents in the care of their infant Improving end-of-life care for neonates and their families Exploring the role of parents in ethical decision making* Healthcare workers-Knowledge and Competence Healthcare professionals behaviour: *Research priorities identified: (3) clinical nursing care practices; (4) quality and safety; (5) ethics; and (8) professional issues in neonatal intensive care nursing.* Pain Pain management: *Research priorities identified: Identifying effective interventions to prevent or reduce pain or stress Identifying best practices for pain assessment Identifying pain and/or stress guidelines* |

**eFigure 2: Results Of Permutation Test Analysis Regarding Gestational Age**


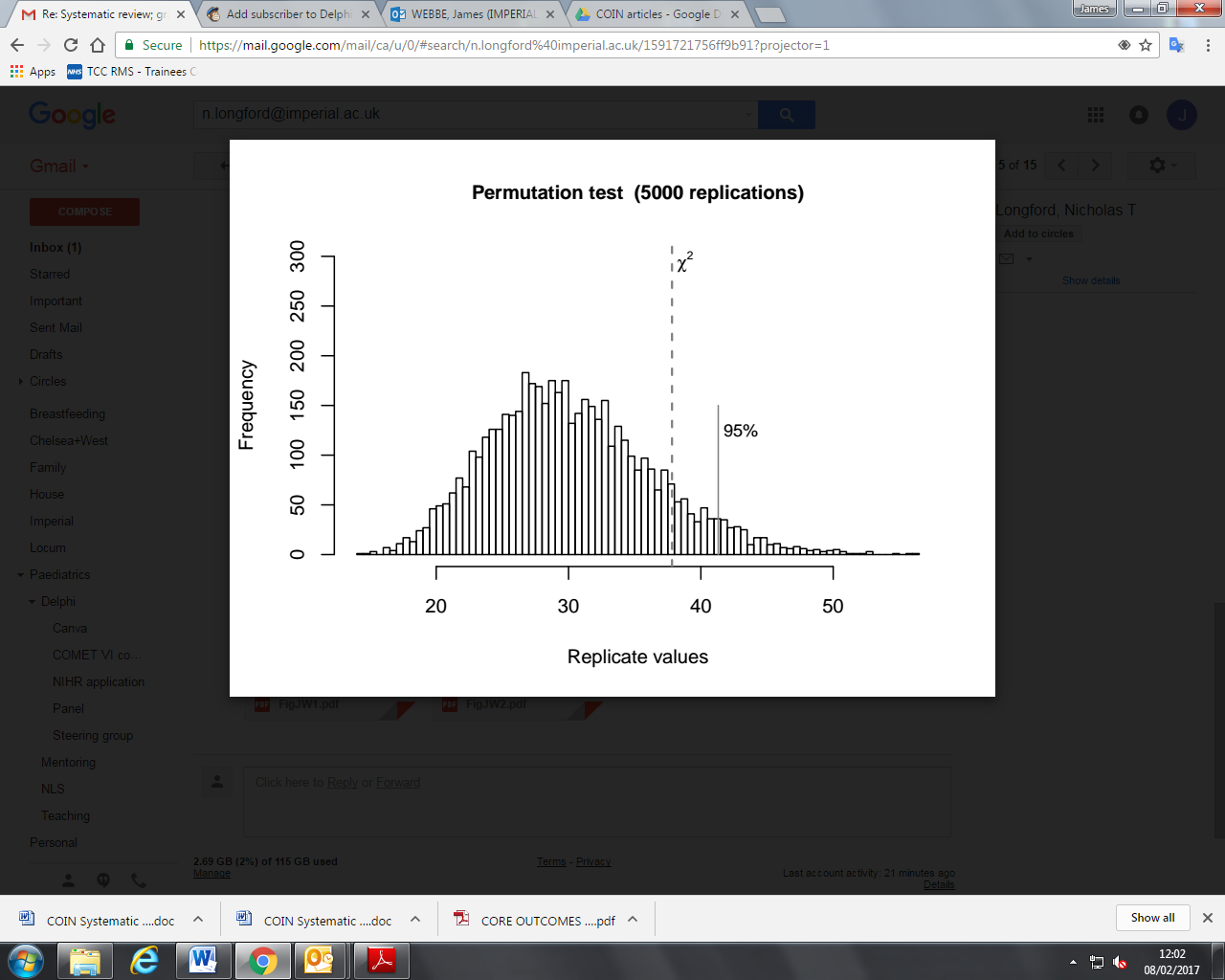


Permutation test analysis for association of gestational age of babies within a study and outcome categories identified within study. The diagram presents the null-distribution of the chi-square (goodness-of-fit) statistic obtained by simulations. The X axis describes the “replicate value” assigned to each replicate while the Y axis describes the frequency with which each replicate value arose during the 5000 replications.  The distribution does not have the chi-square shape because some articles/papers contribute with more than one entry in the contingency table of parties-by-topics.  The vertical dashes (χ^2^) mark the value of the statistic (evaluated on the observed dataset) and the vertical solid line (95%) is the critical value, where the distribution has its 95th percentile.  The value of the statistic is 37.82, the critical value is 41.31 (p-value=0.114).

**eFigure 3: Results Of Permutation Test Analysis Regarding Stakeholder Group**


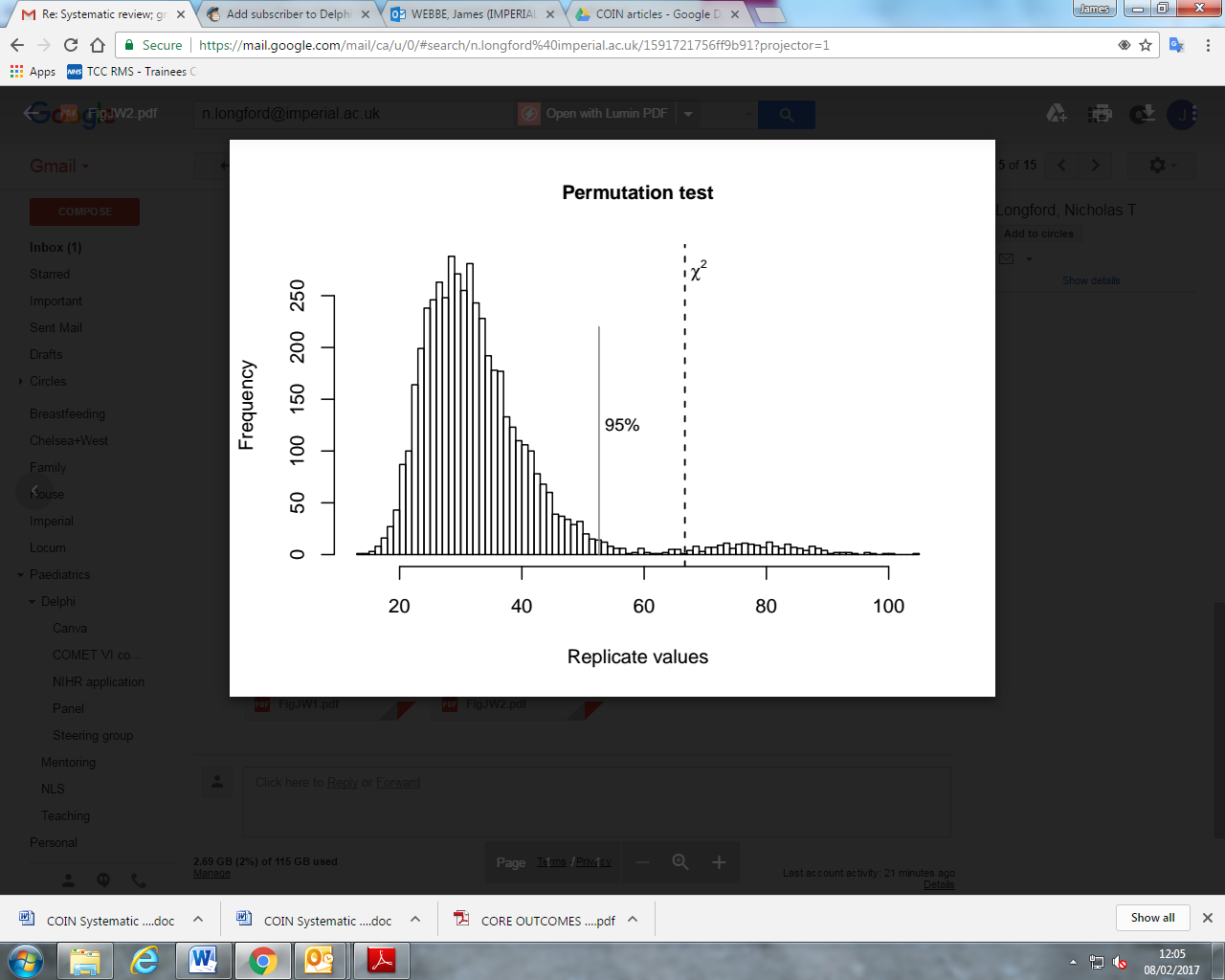


Permutation test analysis for association of stakeholder groups within a study and outcome categories identified within study. The diagram presents the null-distribution of the chi-square statistic obtained by simulations. The X axis describes the “replicate value” assigned to each replicate while the Y axis describes the frequency with which each replicate value arose during the 5000 replications.  The distribution does not have the chi-square shape because some articles/papers contribute with more than one entry in the contingency table of parties-by-topics.  The vertical dashes (χ^2^) mark the value of the statistic (evaluated on the observed dataset) and the vertical solid line (95%) is the critical value, where the distribution has its 95th percentile.  The value of the statistic is 66.67, the critical values is 52.62 (p-value=0.037).
