# Supplementary material for: Parent, patient and clinician perceptions of outcomes during and following neonatal care: a systematic review of qualitative research
Source: BMJ Paediatr Open. 2018 Oct 9;2(1):e000343. doi: 10.1136/bmjpo-2018-000343 (PMC6203019; doi:10.1136/bmjpo-2018-000343)
Supplement: Author's manuscript [file bmjpo-2018-000343.draft_revisions.pdf]

BMJ Paediatrics Open

BMJ Paediatrics Open is committed to open peer review. As part of this commitment we make the peer review history of every article we publish publicly available.

When an article is published we post the peer reviewers' comments and the authors' responses online. We also post the versions of the paper that were used during peer review. These are the versions that the peer review comments apply to.

The versions of the paper that follow are the versions that were submitted during the peer review process. They are not the versions of record or the final published versions. They should not be cited or distributed as the published version of this manuscript.

BMJ Paediatrics Open is an open access journal and the full, final, typeset and author-corrected version of record of the manuscript is available on our site with no access controls, subscription charges or pay-per-view fees (http://bmjpaedsopen.bmj.com).

If you have any questions on BMJ Paediatrics Open's open peer review process please email info.bmjpo@bmj.com

BMJ Paediatrics Open

Confidential: For Review Only

Parent, patient and clinician perceptions of outcomes during and following neonatal care: a systematic review of qualitative research

Journal:	*BMJ Paediatrics Open*
Manuscript ID	bmjpo-2018-000343
Article Type:	Original article
Date Submitted by the Author:	12-Jul-2018
Complete List of Authors:	Webbe, James; Imperial College London, Academic Neonatal Medicine<br>Brunton, Ginny; University College London Institute of Education<br>Ali, Shohaib; Imperial College London Faculty of Medicine<br>Longford, Nicholas ; Imperial College London, Department of Medicine<br>Modi, Neena; Imperial College London, Neonatal Medicine<br>Gale, Chris; Imperial College London, Academic Neonatal Medicine
Keywords:	Neonatology, Outcomes research, Patient perspective

SCHOLARONE™
Manuscripts

Parent, patient and clinician perceptions of outcomes during and following neonatal care: a systematic review of qualitative research

James Webbe[1], Ginny Brunton[2], Shohaib Ali[3], Nick Longford[4], Neena Modi[5], Chris Gale[6] on behalf of the Core Outcomes in Neonatology (COIN) project steering group*

* Collaborative group listed below

[1] James Webbe, MB BChir

Neonatal Medicine, Imperial College London

j.webbe@imperial.ac.uk

[2] Ginny Brunton, PhD

UCL Institute of Education, London

g.brunton@ucl.ac.uk

[3] Shohaib Ali

Imperial College, London

shohaib.ali14@imperial.ac.uk

[4] Nicholas Longford, PhD

Neonatal Medicine, Imperial College London

n.longford@imperial.ac.uk

[5] Neena Modi, MD

Confidential: For Review Only

Neonatal Medicine, Imperial College London

n.modi@imperial.ac.uk

[6] Chris Gale, PhD

Neonatal Medicine, Imperial College London

christopher.gale@imperial.ac.uk

Members of the COIN Steering Group

Elsa Afonso

Neonatal Staff Nurse. Cambridge University Hospitals NHS Foundation Trust, Cambridge.

Iyad Al-Muzaffar

Consultant Neonatologist and Parent of preterm baby. Cwm Taf University Health Board, Wales.

Ginny Brunton

Midwife and Research Methodologist. UCL Institute of Education, London.

James Duffy

Doctoral Research Fellow in Obstetrics and Gynaecology. Nuffield Department of Primary Health Sciences, Oxford.

Chris Gale

Clinical Senior Lecturer in Neonatal Medicine. Imperial College, London

Anne Greenough

Professor of Neonatology and Clinical Respiratory Physiology. King's College, London.

Nigel Hall

Associate Professor of Paediatric Surgery. University of Southampton, Southampton.

Marian Knight

Professor of Maternal and Child Population Health. National Perinatal Epidemiology Unit, Oxford

Jos Latour

Confidential: For Review Only

Professor of Clinical Nursing. Plymouth University, Plymouth.

Neil Marlow

Professor of Neonatal Medicine. University College, London.

Neena Modi

Professor of Neonatal Medicine. Imperial College, London.

Laura Noakes

Parent of neonatal patient.

Julie Nycyk

Consultant Neonatologist, Sandwell and West Birmingham Hospitals NHS Trust, West Midlands.

Mehali Patel

Research Engagement Officer. Bliss, London.

Angela Richard-Londt

Parent of neonatal patients.

James Webbe

Clinical Research Fellow. Imperial College, London.

Ben Wills-Eve

Ex-neonatal patient.

Corresponding Author

Chris Gale, Senior Clinical Lecturer in Neonatal Medicine

Section of Neonatal Medicine, Chelsea and Westminster Campus, Imperial College London

369 Fulham Road, London, SW10 9NX, United Kingdom

+ 44 (0)203 3153519

christopher.gale@imperial.ac.uk

Manuscript word count: 2491

Confidential: For Review Only

ABSTRACT

Objective

Multiple outcomes can be measured in infants that receive neonatal care. It is unknown whether outcomes of importance to parents and patients differ from those of health professionals. Our objective was to systematically map neonatal care outcomes discussed in qualitative research by patients, parents and healthcare professionals and test whether the frequency with which outcomes are discussed differs between groups.

Design

Systematic review of qualitative literature. The following databases were searched: Medline; CINAHL; EMBASE; PsycINFO and ASSIA from 1997 to 2017. Publications describing qualitative data relating to neonatal care outcomes, reported by former patients, parents or healthcare professionals, were included. Narrative text was analysed and outcomes grouped thematically by organ system. Permutation testing was applied to assess an association between the outcomes identified and stakeholder group.

Results

Sixty-two papers containing the views of over 4100 stakeholders were identified; 146 discrete outcomes were discussed; 58 outcomes related to organ systems and 88 to other more global domains. Permutation testing provides evidence that parents, former patients and health professionals reported outcomes with different frequencies (p=0.037).

Conclusions

Parents, patients and health professionals focus on different outcomes when discussing their experience of neonatal care. A wide range of neonatal care outcomes are reported in qualitative research; many are global outcomes relating to the overall status of the infant. The views of former patients and parents should be taken into consideration when designing research; the development of a core outcomes set for neonatal research will facilitate this.

Abstract word count: 244

Confidential: For Review Only

INTRODUCTION

In high resource settings approximately one in ten babies will require care in a neonatal unit (1). Conditions such as preterm birth affect patients' long-term outcomes: consequences include cardiovascular disease in adulthood (2), neurosensory impairment (3), respiratory disease (4), and lower rates of employment and marriage (5). Infants born more prematurely tend to have worse outcomes (6). As neonatal survival for babies of all gestational ages improves long-term outcomes become more important.

Parents and patients are rarely involved in outcome selection in paediatric research (7). Poor outcome selection causes research waste (8): research produced is not relevant to patients' lives. Neonatal care, and the underpinning research, should focus on outcomes important to those it affects most: former neonatal patients, parents and healthcare professionals (8, 9). Identifying these outcomes is crucial to ensure research is relevant and efficient (8, 10). Qualitative research provides a rich description of complex phenomena such as neonatal care (11). One commonly used approach to identify outcomes of importance to stakeholders is primary qualitative research. Considerable qualitative research exploring how parents and health professionals perceive neonatal care has been conducted previously (12, 13); therefore by systematically reviewing published qualitative research it is possible to map the outcomes discussed by different groups.

In this study we aimed to map the range of outcomes identified in qualitative literature by different stakeholder groups: parents, ex-neonatal patients and healthcare professionals. We also wanted to test the hypotheses that stakeholder groups prioritise outcomes differently, and that outcomes identified differ by infant gestational age category.

Confidential: For Review Only

This work is a component of a wider programme to compile a core outcomes set for neonatology (14). A core outcomes set is an agreed collection of important outcomes identified through robust consensus methods by all key stakeholder groups (15). The results of this study will be combined with the results of a systematic review of outcomes reported in clinical trials (16). These will be used as the starting point for the consensus process to determine a core outcomes set (14).

METHODS

We registered this systematic review prospectively on PROSPERO (prospective register of systematic reviews): CRD42016037874 (17). We conducted the review according to PRISMA guidelines (18). We searched the following databases: Medical Literature Analysis and Retrieval System Online (MEDLINE); Cumulative Index to Nursing and Allied Health Literature (CINAHL); Excerpta Medica Database (EMBASE); Psychological Information Database (PsycINFO) and Applied Social Sciences Index and Abstracts (ASSIA). Qualitative or mixed method studies were included if they contained outcomes identified by stakeholders in the context of babies admitted to a neonatal unit. Full inclusion and exclusion criteria are listed in eTable 1. We considered all studies published from 1st January 1997 to 1st January 2017 in a peer review journal in all languages. The databases were last searched on 14th February 2017. The search strategy utilised for MEDLINE is described in eFigure 1. The terms derived from this search strategy were translated to other databases.

All identified papers were screened by title and abstract and then by full text. After double-screening a sample of papers and agreeing criteria all screening was completed by one researcher (JW). For quality assurance, a second researcher screened a random 10%

Confidential: For Review Only

sample of abstracts and titles (CG). Agreement between reviewers was assessed by Cohen's kappa coefficient (19).

After screening all papers were coded independently by two researchers (JW and CG or GB) using Eppi-Reviewer 4 software (20). Any disagreement was resolved by a third researcher (CG or GB). Data on study design, stakeholder demographics, infant birth characteristics and verbatim text relating to neonatal care outcomes were extracted and stored. Quality assessment of individual studies was not undertaken as it is a controversial area of uncertain value in relation to qualitative research (21).

All outcomes were grouped according to a previously defined framework of organ systems (22) using the following domains: cardiovascular; respiratory; gastrointestinal; neurological; genitourinary; infection; skin and development. All three reviewers jointly refined this framework using methods incorporating thematic analysis (23-25). Where narrative data did not fit clearly into the domains, dialogue between all reviewers was used to develop new domains. Outcome domains were thematically analysed to develop higher order categories. A new hierarchy was developed to group outcomes because established hierarchies either did not relate well to neonatal care (26-28) or missed key concepts (15). This outcome hierarchy is described in text box 1.

Text box 1

An example of an outcome hierarchy:

Text extracts to identify or infer a result of clinical care, the 'outcome' such as "*Bonding with parents*".

Similar 'outcomes' were grouped into thematically linked 'domains' such as "*Relationships with others*".

Confidential: For Review Only

‘Outcome domains’ relating to similar concepts were grouped into ‘categories’ such as “*Social*”.

We did not address the ways in which an outcome was measured. For example the ‘outcome’ “*Parental bonding*” could be measured using parent reported scores or an external assessment.

We analysed whether outcomes identified differed by stakeholder groups and by infant gestational age category (using WHO definitions of prematurity) (29). We used permutation testing (30) to test for an association between the frequency that outcomes in different domains were identified and the stakeholder group involved. We performed five thousand replications to generate the distribution of the test statistic under the null hypothesis of no association, and compared our results to this distribution. We performed a similar analysis to test for an association between infant gestational age and frequency of outcome reporting. If a significant association was found we explored this further in a post-hoc analysis to identify where the observed results differed most from the frequencies expected under the hypothesis of no association established by the permutation analysis.

RESULTS

Database searches produced 1130 results which were screened and assessed for eligibility (Figure 1). After applying inclusion and exclusion criteria 62 studies containing the views of 4100 stakeholders were analysed. Agreement between reviewers was high (Cohen’s kappa coefficient=0.81) (19).

The 62 included studies reported data from 15 countries; 9 related to full-term infants, 31 to preterm infants (born <37 weeks gestational age) and 20 to extremely preterm infants

Confidential: For Review Only

(born <28 weeks gestational age). A range of methodologies was used including direct observation (13 studies) and individual (25 studies) or group interviews (13 studies). Questionnaires were used in 21 studies, two of which were Delphi processes. Included studies are described in eTable 2.

Included studies involved over 4100 participants. Parents were the most frequently involved stakeholder group (1969 parents in 40 studies; 65%); former neonatal patients were less commonly included (368 patients in 5 studies; 8%). Nurses and midwives were the professional group involved most often (1096 involved in 24 studies; 39%). Three hundred and sixteen doctors were involved in 18 studies (29%). We also identified 351 additional participants consisting of other family members, teachers, social workers and allied health professionals. In many studies, particularly those employing observation of clinical practice, the total number of research participants was not recorded.

One hundred and forty six distinct outcomes were extracted from the included studies. Fifty eight outcomes related to organ systems within the original framework; we were unable to categorise 88 outcomes within the original framework. The final framework is shown in Table 1. An example of the thematic analysis leading to the expanded framework is shown in Text Box 2.

Confidential: For Review Only

Table 1 Final Outcome Framework

**Outcome Domain Categories**	**Outcome Domains**
Organ system outcomes	Cardiovascular
	Respiratory
	Gastrointestinal
	Respiratory
	Neurological
	Genitourinary
	Infection
	Skin
	Developmental
Holistic outcomes	*Survival*
	Growth
	Pain
	Suffering
	Normality
	Other outcomes
Parent focussed outcomes	*Parental support*
	Other outcomes
Healthcare delivery outcomes	*Healthcare workers - Knowledge and Competence*
	Healthcare workers - Communication
	Other outcomes
Economic outcomes	*Healthcare utilisation*
	Other outcomes
Social outcomes	*Psychiatric outcomes*
	Relationships with others
	Other outcomes

Outcome domain categories and outcome domains added to the original framework marked in *italics*

Text box 2

Example of framework synthesis related to the outcome of 'Normality'. Thematic analysis of verbatim extracts identified a recurring theme:

"The mother also worried that...Lisa would not have a normal life" (31)

"Being reassured that he was on line for how old he was...Just reassurance he was doing well" (32)

"Finally, a mother called it a developmental land-mark when an older sister dared show her irritation towards her little brother, 'no longer treating him as if he were made of glass'" (33)

Confidential: For Review Only

From this and similar text the outcome of 'Normality' was derived by thematic analysis. It did not fit within the existing outcome hierarchy but was reported extensively, so a new domain was added to the framework again called "Normality". This outcome domain relating to the overall status of the infant was similar to outcome domains like "survival", "vitality" and "growth" so these domains were grouped together as an outcome domain category called: "Holistic outcomes".

The full inventory of outcomes is listed in eTable 3. A table of all outcomes in each study (with verbatim text extracts) is shown in eTable 4.

Outcomes were identified relating to all of the organ systems included in the original framework and assigned to an organ system outcome domain category (Table 2). The organ system outcome domains most frequently discussed at the study level were "development" (32 studies; 52%) and "gastrointestinal" (24 studies, 39%). The individual organ system outcomes most frequently discussed were "language disorders" (8 studies, 13%), "visual impairment" (7 studies, 11%) and "breastfeeding" (7 studies, 11%).

Confidential: For Review Only

Table 2 Organ System outcomes

Organ System Outcome Domains	Number of studies discussing outcome domain *(n=62)*	Outcome	Number of studies discussing outcome *(n=62)*	Verbatim text extract
Developmental	32	Ability to feed themselves	1	*"It was the fact that I was able to experience my children eating with a spoon. It was great!" (33)*
		Ability to undertake sport	1	*They report feeling self-conscious of their appearance and avoid athletic activity, which calls attention to their physique. (34)*
		Ability to walk	3	*"He walked four, my mother never forgot, she says it was a miracle of God" (35)*
		Difficulties with activities of daily living	4	*"A lot of times I have to put myself in certain positions to do things, like opening a jar- I have to squeeze it in between my arms instead of gripping it with my hand." (36)*
		Hearing impairment	5	*"I told the parents that he will never be able to see, to hear and I will get more data to show them how bad things are." (37)*
		Issues of development and motor skills	5	*"We both looked at my child, research, experience and what I could expect." (38)*
		Language disorders	8	*"I also had a hard time learning to talk." (39)*
		Need for physical therapy	1	*significantly more BPD children received...occupational, and physical therapy (40)*
		Normal hearing	1	*"She got to hear her dad sing" (41)*
		Retinopathy of prematurity	1	*Outcomes identified by CLD focus group: ROP rates (42)*
		Social difficulties	2	*Social communication is difficult because of his hearing and speech problems, and he is described as having a few friends and no experience in dating (34)*
		Visual impairment	7	*At the time of the interviews, the only major sequel was in one child with seriously impaired vision. (33)*
Gastrointestinal	24	Breastfeeding	7	*"I fully breastfed for four months-100%-and I am so proud of it." (38)*
		Choice of milk for feeding	2	*"it's like they [scn providers] didn't inform us when they were trying to feed my daughter [formula]" (43)*
		Choking during feeding	1	*"The only concern I have is, I don't want them to choke. I'm fearful of choking." (44)*
		Eating disorder	1	*Health Outcome reported by parents: Table 1: -eating disorder (45)*
		Feeding difficulties	5	*"We kept on saying to the staff on neonatal unit that it was only Gray's feeding that was stopping him from going home, everything else was fine" (46)*
		Feeding practices	2	*Mothers had difficulty understanding these instructions and seemed hesitant to liberalize their infant's intake after discharge. (44)*
		Fistulas	1	*One-third of all the children had a digestive issue, such as…fistulas (47)*
		Frequency of defaecation	1	*"A handbook with instructions about elimination, like if it's normal for the baby to go two days without defaecation" (48)*
		Initiating enteral feeds	2	*"MEF* [minimal enteral feeds] *should be initiated in first 2-3 days of life as long as the*

Confidential: For Review Only

				baby is stable." (49)
		Liver failure	1	*One-third of all the children had a digestive issue, such as…short gut (47)*
		Necrotising enterocolitis	1	*"If we are trying to stimulate the gut before it is ready making it more prone to having complications of either feeding intolerance or, more severely, necrotizing enterocolitis." (49)*
		Nutritional intake	1	*"Ever since he's been home, we've kept records of how much he was eating, on a daily basis." (50)*
		Oral feeding	3	*"(The) very first time (feeding the baby) was just great, to tell you the truth." (51)*
		Other gastrointestinal malformations	1	*One-third of all the children had a digestive issue, such as trachea-related malacias, fistulas, malformations, gut related perforations, absences (47)*
		Regurgitation	1	*"The other day I got really scared, the baby regurgitated, and milk came out of her nose, I went crazy" (48)*
		Short gut syndrome	1	*One-third of all the children had a digestive issue, such as…liver failures.(47)*
Respiratory	12	Asthma	1	*Health Outcome reported by parents: Table 1: -asthma (45)*
		Breathlessness	1	*Education priorities: Managing symptoms (e.g., secretions, breathlessness) in a dying baby (52)*
		Chronic Lung Disease	1	*Chronic Lung Disease Outcomes identified by CLD focus group: Chronic Lung Disease rate (42)*
		Excessive secretions	1	*Education priorities: Managing symptoms (e.g., secretions, breathlessness) in a dying baby (52)*
		Frequent respiratory illnesses	2	*"There were lots of masks and nebulisers during those years" (33)*
		Mechanical ventilation	5	*Over 30% of all infant descriptions were about babies who had tracheostomies and were unable to be weaned off a ventilator. (47)*
		Nasal congestion	1	*"If having nose congestion is something common for every premature baby" (48)*
		Oxygen dependence	5	*"My babies did not fit into the criteria for going home early due to one of the twins still being dependent on oxygen." (46)*
		Pneumothorax	1	*Chronic Lung Disease Outcomes identified by CLD focus group: … Pneumothorax rate (42)*
Neurological	11	Brain damage (not further specified)	2	*"Brain injury is laden with a lot more emotions and moral concerns for sure" (53)*
		Neurological care	1	*Research priorities identified: Identifying the best care practices for infants with neurological problems (54)*
		Neurological symptoms	2	*"can't feel some - my left - this is numb right here." (55)*
		Seizures	2	*"I explained this to the doctor. And he was the one that said it could possibly be seizures. So we're going to take him in and have him tested." (50)*
		Significant IVH	2	*"Although she has a grade IV bleed, the resident says that she moves and looks around, and he thinks the odds are quite good." (37)*
		Sleep disorders	4	*Subsequent to an account of the son's disturbed sleep at night for several months after discharge, which was an enormous challenge to the couple (33)*
Surgical	5	Appearance of scars	2	*"I do not like the scar on my belly […]; I was at the beach and everyone kept staring at*

Confidential: For Review Only

				me like `That is a big scar!´" (39)
		Care for surgical babies	1	*Research priorities identified: Identifying the best care practices for surgical infants (54)*
		Need for ileostomy	1	*"One mother, left with one surviving twin who has an ileostomy, told me that she is glad the sibling died and does not have to endure what the living twin is enduring." (56)*
		Need for multiple operations	2	*the mother also worried that there would be more surgeries (31)*
Infection	5	Prevention of infection	1	*"She told me of the fact that they were earlier and didn't have the capabilities of fighting off infections, and they needed that from the milk, from me." (57)*
		Sepsis	3	*Decrease blood stream infections Selected as key performance indicator (58)*
		Susceptibility to infection	1	*"The biggest worry right now is when he is going to get sick." (50)*
Skin	4	Appearance of scars	2	*In addition, hospitalization and different interventions in their first days of life have left marks on their bodies. (39)*
		Burns	1	*"I didn't believe that it could really burn the skin until I had experienced it." (59)*
		Extravasation injuries	1	*"The i.v. site was covered with cloth.... leaked for a long time, skin area was inflamed and oedematous like Popeye's arm." (59)*
		Pressure sores	1	*"I had to skip changing a [hydrocephalus] baby's position for that shift … Her head developed pressure sores." (59)*
		Skin care	1	*Research priorities identified: Identifying and evaluating interventions to improve skin and wound care in neonates (54)*
Cardiovascular	1	Hypotension	1	*Significant factors that make professionals categorise a baby as 'sick' "hypotension" (49)*
		Presence of Patent Ductus Arteriosus	1	*Significant factors that make professionals categorise a baby as 'sick' PDA (49)*
Genitourinary	1	Urological disorders	1	*Persistent urological problems concern twin A's mother as to whether he will be accepted or teased by his peers when he is older.(34)*

Confidential: For Review Only

Confidential: For Review Only

The majority of outcomes identified did not relate to individual organ systems. Some related to the overall status of the infant and were assigned to a holistic outcome domain category (Table 3). Other domains related to the effects experiencing neonatal care has on parents; these were assigned to a “Parent focussed” outcome domain category (Table 4). Another group of domains related to the neonatal care delivered; these were assigned to a “Healthcare delivery” outcome domain category (Table 5). A group of domains was identified relating to the cost of neonatal care; these were assigned to an “Economic” outcome category (Table 6). Finally a group of outcome domains was identified relating to the relationships neonatal patients develop with others; these were assigned to a “Social” outcome domain category (Table 7).

Confidential: For Review Only

Table 3 Holistic outcomes

Holistic Outcome Domains	Number of studies discussing outcome domain (n=62)	Outcome	Number of studies discussing outcome (n=62)	Verbatim text extract
Normality	22	Ability to lead a normal life	2	*“the mother also worried that...Lisa would not have a normal life” (31)*
		Being treated normally	1	*“I have always been treated differently than my sisters. Yes, they tend to be overprotective, that bothers me” (39)*
		Inability to create a normal life	1	*"We are so busy in our ICU doing our tasks that the chronic kids are left for periods of time alone in their cribs with little interaction except mobiles." (47)*
		Normal health	1	*Table 1: -parents perception of child's general health was measured (45)*
		Normality	16	*A major focus for parents was seeking information that told them that what was happening was ‘normal’ and that everything was going to be ‘fine’. (60)*
		Thriving	1	*“Seeing babies younger than him thrive. . . and then seeing the babies approximately his age survive, thriving, and doing well." (61)*
Suffering	15	Comfort	4	*"You can almost feel what it’s like in the incubator, lying on the lambskin, that it's how I would want to have laid and . . . Well, it looks very comfortable" (62)*
		Ex-patients separation from their suffering	1	*“Parents suffer much more than us; they had to suffer waiting for us to get better and see us in that status”(39)*
		Suffering	9	*"This infant's short life was never comfortable...I frequently felt we were torturing the child just doing daily care." (47)*
		Symptom control	1	*Educational priorities identified included: Managing symptoms (e.g., secretions, breathlessness) in a dying baby (52)*
Survival	14	Survival	11	*"It hurts. I didn't know, I didn't know if they were going to make it or not." (50)*
		Survival with disability	3	*"It isn't up to us to say what is quality of life, because parents might think that even if the child was disabled, that it was better than not having a child at all." (63)*
		Survival without disability	4	*"And afterwards you are worried about how they are going to survive. If they would have impairments, and so on." (33)*
Growth	8	Growth	8	*"She was born so early, it's nice to see that she's finally catching up with how she's growing" (32)*
Pain	7	Chronic pain	1	*“She occasionally complains of pain in her shoulder area. Maybe 1-2 times per month." (36)*
		Pain	4	*“It like hurts when you wake up in the morning.” (55)*
		Pain management	2	*Research priorities identified: Identifying effective interventions to prevent or reduce pain or stress (54)*
Other outcomes		Overall health state	2	*“We try to think of the whole life consequence. We talk about, you know, strength and cognitive capacity, but also life and communication and feeding yourself and getting*

Confidential: For Review Only

				around." (53)
		Vitality	2	*"The doctor said that, whatever we do, however good we are, it is mostly up to the infant himself. No matter how small they are, they can have something within them-selves." (64)*
		Physiological stability	1	*The qualitative data show that nurses are aware that increased noise levels in a NICU have detrimental physiological effects for the newborn (65)*
		Physical appearance	7	*Both mothers and fathers found their infant's appearance and behaviour to be the stressors with the most impact (66)*

Confidential: For Review Only

Table 4 Parent focussed outcomes

Parent Focussed Outcome Domains	Number of studies discussing outcome domain *(n=62)*	Outcome	Number of studies discussing outcome *(n=62)*	Verbatim text extract
Parental support	30	Balancing caring for themselves and their baby	1	*Mothers often spoke of the conflict between taking care of themselves and their other responsibilities and the need to pump (67)*
		Barriers to parental involvement	1	*"I felt at times that I had to ask permission to change my baby's nappy or check that it required changing although I understood that it was to protect Gray from too much 'messing about' ." (46)*
		Care provided close to home	1	*"[i was glad our infant was transferred to the com-munity hospital] because of lack of transportation. Because then i [was] able to see him more." (43)*
		Coping with maternal illness	5	*One nurse spoke of her belief that mothers could be diagnosed with depressive conditions, particularly when they took home a baby that had ongoing health problems or a disability, and the mother came to understand the reality of her situation. (63)*
		Culture differences	2	*3 families felt strongly that their stress derived from differences in the medical management approaches between the United States and their homeland. (66)*
		Maintaining hope	1	*The informants described how parents thought there was hope so long as the child was in hospital, and the doctor's dilemma consisted in both keeping the parents' hope alive and in giving them a realistic picture of the situation (68)*
		Online support	1	*"This group is for parents who have experienced the miracle of having a preemie. Let's share our stories to help each other through the difficult times of NICU and support each other through the even more challenging task of raising a preemie." (69)*
		Parental ability to work	2	*They liked being back at work because it helped occupy their minds, but they reported being exhausted (67)*
		Parental competence	4	*"We learned everything we needed and knew what we had to do, I was quite comfortable when we went home." (38)*
		Parental involvement	10	*"During our baby's stay in the neonatal unit both myself and Peter were fully involved in our son's care and did most of the caring such as nappy changing and NGT feeds" (46)*
		Preparation for NICU admission	1	*'For several mothers, the tour inspired hope for their newborn's prognosis, especially when the mothers saw very premature infants who were said to be progressing well.' (61)*
		Support from faith	1	*"We regard Philippa as a gift from God and so when she was ill, you know we prayed" (70)*
		Support from family and friends	5	*"My mother in law and my mother both would watch my older daughter that first year quite a bit while I would take my daughter to therapy" (36)*
		Support from fathers	2	*Fathers ranged from being very supportive, "[we're] in this together," to being deterrents*

Confidential: For Review Only

				or completely absent. (67)
		Support from healthcare professionals	6	*"The nursing staff, the doctors...they really know what they're doing...not only medically, but dealing with us personally...that helped a lot." (41)*
Other outcomes		Long term effects on parents	2	*We should be looking at Postnatal Depression after the baby goes home. . .Once they actually get a baby home, that's when the reality sets in. All the triggers are there. (63)*
		Support beyond NICU	1	*"the medical professionals who have become your support system and your family while your child was alive...all of a sudden they're gone...I think it's important to have some kind of support system or information...otherwise it's like stepping into this huge abyss." (41)*
		Parental perception of uncertainty	1	*parent's perception of infant's illness and future impact (66)*

Confidential: For Review Only

Table 5 Healthcare delivery outcomes

Healthcare Delivery Outcome Domains	Number of studies discussing outcome domain *(n=62)*	Outcome	Number of studies discussing outcome *(n=62)*	Verbatim text extract
Healthcare workers - Communication	30	Allowing time for conversation	1	*"There was plenty of time to consider it really thoroughly and ask questions." (38)*
		Awareness of parental views	1	*The data show that in 9 cases the clinical team had no knowledge of the parents' wishes. (71)*
		Candour	1	*"It's about trust, you know. I will trust someone who was honest and said he didn't know (more) than someone who lied." (72)*
		Communicating in challenging settings	10	*When they arrived at hospital in preterm labour, or were admitted for observation and bed rest, some had difficult conversations with clinical staff about the implications of their gestational stage for decisions about care. NICUs commonly set boundaries around the care that they offer (73)*
		Communicating information effectively	7	*Other parents experienced stress from unknown medical terminology(66)*
		Communication about discharge	3	*Parents/care givers may be inadequately prepared for home care and management of fragile neonates due to a lack of consistent and early communication (74)*
		Communication with ex-neonatal patients	1	*On the one hand, many participants stated that they were thankful and appreciated the bonds established in the hospital setting. (39)*
		Communication with parents	2	*"When you're talking to parents while you're doing cares and everything, you're not really talking to them,... you're having a vague conversation across the room" (75)*
		Developing a caring relationship	5	*As the providers gave support to families, families also were described as supporting the staff (47)*
		Keeping parents informed	7	*"I asked so many questions and read all the charts every day, and i probably angered them. Squeaky wheel gets the oil, as they say." (43)*
		Language barrier	1	*The stress derived from communication with HCPs [healthcare professionals] was mostly related to language barriers for the new immigrant parents.(66)*
		Treating parents with respect	3	*"I got yelled at by a nurse at [the scn] for rubbing my son's foot [even though that was okay at the nicu]." (43)*
		Using aids to communication	1	*Most of the mothers in both groups were positive about having their conversations with the neonatologist taped. (76)*
Healthcare workers – Knowledge and Competence	23	Consistency of decisions	6	*"Everybody had a different point of view but they were opinions, not facts. So that was huge, don't even get me started on that, that was just a nightmare." (60)*
		Ethical decision making	5	*". but when you actually mention this, say, "Well, in fact you know, we don't really know what's the best treatment," it is a delicate moment." (72)*
		Expertise in palliative care	1	*Nurses and doctors talked about being able to clearly recognize that a child was going*

Confidential: For Review Only

				to eventually die, and the used words such as 'torturing' or 'flogging the child to death' with interventions rather than providing comfort care at the end of life. (47)
		Healthcare professionals behaviour	5	*"And then she was talking and it took her 5 minutes to finish her conversation with the other nurse, and it wasn't a nurse related conversation it was just a casual conversation … Like I felt a bit (sic) she wasn't their priority." (60)*
		Healthcare professional competence	7	*Most of the parents recalled specific incidents that they perceived as poor medical care; typically, these incidents involved technical procedures or medical knowledge (77)*
		Identifying who is responsible for care	3	*"Sometimes we're not real clear who to follow up with." (43)*
		Medical errors	1	*"I set the infusion rate at 12 mL/min instead of 1.2 mL/min. I couldn't see the faint decimal point." (59)*
		Staff insecurity	1	*They experienced estimated breastfeeding as a more difficult method because they felt insecure about the infants' intake of breast milk (78)*
		Staffing levels	2	*It was especially helpful for the parents to see so many nurses and physicians in the NICU.(61)*
Other outcomes		Iatrogenic harm	3	*"there are potential toxicities, very real toxicities associated with it, so there is this balance of...benefit and harm..." (72)*
		Inclusion in research	2	*Although most had experienced little contact with a trial over the years, parents were often interested in the research, and some would have liked more contact and information than they actually received.(73)*

Confidential: For Review Only

Table 6 Economic outcomes

Economic Outcome Domains	Number of studies discussing outcome domain *(n=62)*	Outcome	Number of studies discussing outcome *(n=62)*	Verbatim text extract
Healthcare utilisation	15	Frequent appointments	2	*"I felt left out, I was always missing school because I had to go to the hospital for check-ups" (39)*
		Frequent readmissions	4	*The prolonged hospitalizations experienced by children with BPD and the frequent interactions of families with medical personnel may result in increased access and opportunities for services for parents of children with BPD. (40)*
		Inappropriate treatments	2	*Community providers…may lack the required knowledge and skills to manage complex infants, leading to suboptimal office-based care and perceived overutilisation of the emergency system.(74)*
		Need for frequent treatments	3	*"There were lots of masks and nebulisers during those years" (33)*
		Need for lifelong care	3	*"MDs are making such choices for parents and when the outcome is disastrous they just expect parents to take home severely handicapped babies and deal with life-long problems." (56)*
		Recurrent sickness	1	*"We've only put him with other children for the past month. The biggest worry right now is when he is going to get sick." (50)*
Other outcomes		Duration of admission	2	*Decrease length of stay selected as key performance indicator (58)*
		Healthcare resources	3	*Although respondents frequently discussed the emotional toll to all concerned, the monetary cost of long-term stays was very rarely (<1%) mentioned.(47)*

Confidential: For Review Only

Table 7 Social outcomes

Social Outcome Domains	Number of studies discussing outcome domain *(n=62)*	Outcome	Number of studies discussing outcome *(n=62)*	Verbatim text extract
Relationships with others	19	Bonding with family and friends	3	*"The only thing we might have done...some of our closest friends...it would have been nice to have them there as well" (41)*
		Bonding with parents	8	*"I find it a great joy when the mums do hold the baby against their chest" (79)*
		Childhood happiness	1	*"But to me I look at her and I don't see it all. I see this little happy little thing!" (50)*
		Effects on family and friends	7	*Almost all parents acknowledged the emotional adjustment of other family members in response to raising a child with physical impairment. (36)*
		Family resources	2	*3 families felt overwhelmed by a lack of resources (especially in the area of family support) (66)*
		Overprotective parent-child relationship	1	*"I started caring for myself and I had to push her away a bit so that I could lead my own life" (39)*
		Peer acceptance	2	*I've had four year olds tell me the other kids don't want to play with them cause they have a dumb arm.(55)*
		Psychological coping	1	*I've had four year olds tell me the other kids don't want to play with them cause they have a dumb arm.(36)*
Psychiatric	7	Autism	1	*Mainstream class appropriate for age without any special support; Psychiatric disorder; Autism (45)*
		Behavioural disturbances	1	*"Because she did not act like a child of her age, because beyond being premature, I did not deal with it in the crying, agitation." (35)*
		Dyslexia	1	*"We were told that when he started school he might have problems, dyslexia or learning disabilities." (33)*
		Mood disorders	1	*Occasionally fretful, angry, irritable, anxious, depressed, or experiencing 'night terrors' (80)*
		Need for educational support	7	*The patient is at an age-appropriate grade level but attends resource classes in math and achieves only average grades in other areas. (34)*
		Psychiatric disorder	3	*The mother is very focused on the boys' physical and emotional symptoms (34)*
Other outcomes		Schooling	1	*"I attend a special education school because when I was in a bilingual school I could not advance to the next grade, the other kids mocked me or left me aside, they did not understand my problem. I am doing fine now" (39)*
		Self-identifying as premature	1	*The meaning that subjects assign to having been born preterm varies according to their health status, sequelae and life experience. (39)*

Confidential: For Review Only

From these outcome domains the most frequently discussed at study level were “parental support” (30 studies, 48%) and “healthcare workers - communication” (30 studies, 48%). The individual outcomes most frequently discussed were “normality” (16 studies, 26%) and “survival” (11 studies, 18%).

Permutation test analysis showed a statistically significant association (p=0.037) between different stakeholder groups and outcome categories identified (eFigure 3). The frequency with which patients discussed the outcomes was most divergent from the other groups. In particular, patients discussed outcomes relating to the genitourinary, surgical, developmental and pain outcome domains more than would be expected by chance. We found no statistically significant association (p=0.114) between gestational age and outcome categories (eFigure 2).

DISCUSSION

We have systematically reviewed and synthesised the outcomes reported in qualitative research by those with lived experience of neonatal care: patients, parents and healthcare professionals. We show that the patterns of outcomes discussed by former neonatal patients, parents and healthcare professionals are different. This is in keeping with previous single centre research (81) and case reports (82). This indicates that healthcare professionals should consider whether the outcomes they discuss align with patients’ and parents’ concerns (83). Acceptance of the differences shown should form part of the process of shared decision making in clinical practice (84). Poor outcome selection is also a known problem in paediatric research (7, 85), involving patients and parents will help to reduce research waste (86, 87).

Confidential: For Review Only

The outcomes identified extend beyond the organ system specific outcomes commonly reported in clinical trials and include global concepts such as “normality” of the child in later life, the impact on an infant’s family and the healthcare team, financial and time costs, and how patients interact with wider society. Our findings are in keeping with observational studies illustrating the wide reaching consequences of neonatal illness (88-90). Another feature of the outcomes identified is that rather than relating to a specific diagnosis or disease many reflect the global status of the child. Diagnoses like necrotising enterocolitis or retinopathy of prematurity were mentioned less frequently than their consequences, such as feeding difficulties or visual impairment. In general, the outcomes identified indicate that pathological processes and diagnoses are less relevant to patients and parents than the effects they have on day to day life. Priority should be given to identifying efficient ways of measuring more global outcomes of neonatal conditions throughout childhood and later life, for example through robust linkage of neonatal data with education databases.

This more holistic approach should extend to how babies are categorised. Our work included an undoubtedly heterogeneous population, but this was driven by discussions with former neonatal patients and parents at the planning stages of this project. They strongly stated that “a sick baby is a sick baby” regardless of birthweight or gestational age: a statement that is supported by our finding that there was no significant difference in how frequently outcomes were discussed in relation to babies of differing gestational ages. Splitting research populations by arbitrary landmarks not recognised by parents or former patients (82) may be a source of research heterogeneity.

The strengths of our study included identification and synthesis of outcomes from an international and methodologically diverse range of studies, relating to babies of all

Confidential: For Review Only

gestational ages, and a wide range of stakeholders. We included outcomes that stakeholders spontaneously identified. As a result, we were able to include data from a wider range and diversity of stakeholders than a primary research study could. We followed a pre-registered protocol with reporting in line with PRISMA guidelines (18). It has been argued that quality assessment is needed in ‘mapping’ reviews to aid in interpretation and uptake of findings (24), but the value of this approach is uncertain (21). The consultation phase of our core outcome set development work will provide the opportunity to critically reflect on the contribution of these findings to our understanding of what constitutes an ‘important’ outcome in neonatal research.

A limitation of our study is that, in line with many systematic reviews, we are synthesising data from studies that did not explicitly address the research question we are asking. This meant that we combined data about which outcomes parents, patients or healthcare professionals mentioned during research. As a result we described how frequently outcomes were discussed, rather than the importance assigned by groups to each outcome. Another limitation is that the researchers who undertook the primary qualitative research in the included studies will have influenced our review through their analysis; we reviewed data that was a step removed from the opinions of the stakeholders themselves. However, by following rigorous methodology and employing a comprehensive search strategy we have combined all available data to produce this mapping review.

This work supports the importance of identifying a core outcomes set, and highlights the importance of input from all stakeholder groups. In other fields core outcomes sets have successfully aligned patient and healthcare professional research priorities (86).

CONCLUSION

Confidential: For Review Only

Parents, patients and clinicians report a wide range of neonatal care outcomes. Parents and patients focus on different outcomes than health professionals. Outcomes reported do not map to organ systems commonly addressed in clinical trials, many are global outcomes. We suggest that the views of former patients and parents should be taken into consideration by researchers and funding bodies.

Confidential: For Review Only

DECLARATIONS

CONFLICT OF INTEREST DISCLOSURE

The authors declare that they have no competing interests.

ACKNOWLEDGEMENTS

The authors are grateful to Louise Wann (West Middlesex University Hospital) for her contributions running the database searches.

AUTHOR CONTRIBUTIONS

JW and CG conceived this systematic review. This protocol was created by JW, GB and CG. Searches were performed by LW. All search results were reviewed by JW and assessed by the eligibility criteria above. Quality assurance was completed by CG. Coding and result synthesis was completed by JW, GB and CG. Statistical analysis was completed by NL. The first draft of the manuscript was written by JW, CG, GB, and NL; NM edited and reviewed the manuscript. It was approved by JW, CG, GB, SA, LW, NL, NM and the COIN Steering Group.

FUNDING AND SPONSORSHIP

This research is sponsored by Imperial College London and supported by an MRC Clinician Scientist Fellowship award to CG (MR/N008405/1) and salary support for JW from the Portland Hospital. Imperial College London, the MRC and the Portland Hospital had no involvement in the research, or this publication.

Confidential: For Review Only

WHAT IS ALREADY KNOWN ON THIS TOPIC

- Multiple outcomes can be measured in infants that receive neonatal care.
- It is not known which outcomes are considered important by former neonatal patients, parents and healthcare professionals, or whether these differ between groups.

WHAT THIS STUDY ADDS

- The predominant outcomes identified by parents, former patients and health professionals related to holistic concepts (such as “normality).
- Significant differences were identified in outcomes discussed by parents, patients and health professionals.
- Differences in neonatal outcomes prioritised by parents, patients and health professionals should be recognised when planning research.

Confidential: For Review Only

REFERENCES

1. Harrison W, Goodman D. Epidemiologic Trends in Neonatal Intensive Care, 2007-2012. JAMA pediatrics. 2015;169(9):855-62. Epub 2015/07/28.
2. Gluckman PD, Hanson MA, Cooper C, Thornburg KL. Effect of in utero and early-life conditions on adult health and disease. The New England journal of medicine. 2008;359(1):61-73. Epub 2008/07/04.
3. Doyle LW, Anderson PJ. Adult outcome of extremely preterm infants. Pediatrics. 2010;126(2):342-51. Epub 2010/08/04.
4. Roberts G, Cheong JL. Long-term growth and general health for the tiniest or most immature infants. Seminars in fetal & neonatal medicine. 2014;19(2):118-24. Epub 2013/12/03.
5. Saigal S, Day KL, Van Lieshout RJ, Schmidt LA, Morrison KM, Boyle MH. Health, Wealth, Social Integration, and Sexuality of Extremely Low-Birth-Weight Prematurely Born Adults in the Fourth Decade of Life. JAMA pediatrics. 2016;170(7):678-86. Epub 2016/05/24.
6. Boyle EM, Poulsen G, Field DJ, Kurinczuk JJ, Wolke D, Alfirevic Z, et al. Effects of gestational age at birth on health outcomes at 3 and 5 years of age: population based cohort study. British Medical Journal. 2012;344.
7. Sinha IP, Williamson PR, Smyth RL. Outcomes in clinical trials of inhaled corticosteroids for children with asthma are narrowly focussed on short term disease activity. PloS one. 2009;4(7):e6276. Epub 2009/07/18.
8. Heneghan C, Goldacre B, Mahtani KR. Why clinical trial outcomes fail to translate into benefits for patients. Trials. 2017;18(1):122. Epub 2017/03/16.
9. Goodare H, Smith R. The rights of patients in research. BMJ. 1995;310(6990):1277-8. Epub 1995/05/20.
10. Staley K. Exploring Impact: Public involvement in NHS, public health and social care research. Eastleigh: INVOLVE; 2009.
11. Sofaer S. Qualitative methods: what are they and why use them? Health services research. 1999;34(5 Pt 2):1101-18. Epub 1999/12/11.
12. POPPY Steering Group. Family-centred care in neonatal units. A summary of research results and recommendations from the POPPY project. London: NCT, 2009.
13. Kain VJ. An exploration of the grief experiences of neonatal nurses: A focus group study. Journal of Neonatal Nursing. 2013;19(2):80-8.
14. Webbe J, Brunton G, Ali S, Duffy JM, Modi N, Gale C. Developing, implementing and disseminating a core outcome set for neonatal medicine. BMJ Paediatrics Open. 2017;1(1).
15. Williamson PR, Altman DG, Bagley H, Barnes KL, Blazeby JM, Brookes ST, et al. The COMET Handbook: version 1.0. Trials. 2017;18.
16. Ali S, Webbe J, Duffy J, Modi N, Gale C, editors. Outcome reporting in trials involving preterm infants: a systematic review. COMET VI; 2016; Amsterdam.
17. PROSPERO database. [13/02/2017]; Available from: http://www.crd.york.ac.uk/prospero/display_record.asp?ID=CRD42016037874. .
18. Moher D, Liberati A, Tetzlaff J, Altman DG. Preferred reporting items for systematic reviews and meta-analyses: the PRISMA statement. Annals of internal medicine. 2009;151(4):264-9, W64. Epub 2009/07/23.
19. Landis JR, Koch GG. The measurement of observer agreement for categorical data. Biometrics. 1977;33(1):159-74. Epub 1977/03/01.
20. Thomas J, Brunton J, Graziosi S. EPPI-Reviewer 4: software for research synthesis. EPPI-Centre Software. London: Social Science Research Unit, Institute of Education, University of London; 2010.
21. Collier D, Mahoney J. Insights and pitfalls - Selection bias in qualitative research. World Politics. 1996;49(1):56-+.

Confidential: For Review Only

22. NHS Digital. National Neonatal Data Set. 2017 [12/07/2017]; Available from: http://www.datadictionary.nhs.uk/data_dictionary/messages/clinical_data_sets/data_sets/national_neonatal_data_set/national_neonatal_data_set_-_episodic_and_daily_care_fr.asp?shownav=1.
23. Arksey H, O'Malley L. Scoping studies: towards a methodological framework. International Journal of Social Research Methodology. 2007;8(1):19-32.
24. Levac D, Colquhoun H, O'Brien KK. Scoping studies: advancing the methodology. Implement Sci. 2010;5(1):69.
25. Thomas J, Harden A, Newman M. Synthesis: combining results systematically and appropriately. In: Gough D, Oliver S, Thomas J, editors. An introduction to systematic reviews 2nd ed. London: Sage; 2017. p. 178-226.
26. Bakas T, McLennon SM, Carpenter JS, Buelow JM, Otte JL, Hanna KM, et al. Systematic review of health-related quality of life models. Health Qual Life Out. 2012;10.
27. Porter ME. What Is Value in Health Care? New England Journal of Medicine. 2010;363(26):2477-81.
28. Carpenter WR, Meyer AM, Abernethy AP, Sturmer T, Kosorok MR. A framework for understanding cancer comparative effectiveness research data needs. J Clin Epidemiol. 2012;65(11):1150-8.
29. Dimes Mo, PMNCH, Children St, WHO. Born Too Soon: The Global Action Report on Preterm Birth. Geneva: World Health Organization, 2012.
30. Ludbrook J. ADVANTAGES OF PERMUTATION (RANDOMIZATION) TESTS IN CLINICAL AND EXPERIMENTAL PHARMACOLOGY AND PHYSIOLOGY. Clinical and Experimental Pharmacology and Physiology. 1994;21(9):673-86.
31. Docherty SL, Lowry C, Miles MS. Poverty as context for the parenting experience of low-income Lumbee Indian mothers with a medically fragile infant. Neonatal network : NN. 2007;26(6):361-9. Epub 2007/12/12.
32. Hussey-Gardner BT, Wachtel RC, Viscardi RM. Parent perceptions of an NICU follow-up clinic. Neonatal network : NN. 1998;17(1):33-9. Epub 1998/04/04.
33. Lou H, Pedersen BD, Hedegaard M. Questions never asked. Positive family outcomes of extremely premature childbirth. Quality of life research : an international journal of quality of life aspects of treatment, care and rehabilitation. 2009;18(5):567-73. Epub 2009/05/01.
34. Votteler TP, Lipsky K. Long-term results of 10 conjoined twin separations. Journal of pediatric surgery. 2005;40(4):618-29. Epub 2005/04/27.
35. Arruda DC, Marcon SS. [Family experience of dealing with sequels of the prematurity of a child]. Revista brasileira de enfermagem. 2010;63(4):595-602. Epub 2010/09/14. Experiencia da familia ao conviver com sequelas decorrentes da prematuridade do filho.
36. Squitieri L, Larson BP, Chang KW, Yang LJ, Chung KC. Understanding quality of life and patient expectations among adolescents with neonatal brachial plexus palsy: a qualitative and quantitative pilot study. The Journal of hand surgery. 2013;38(12):2387-97 e2. Epub 2014/01/15.
37. Orfali K. Parental role in medical decision-making: fact or fiction? A comparative study of ethical dilemmas in French and American neonatal intensive care units. Soc Sci Med. 2004;58(10):2009-22. Epub 2004/03/17.
38. Brodsgaard A, Zimmermann R, Petersen M. A preterm lifeline: Early discharge programme based on family-centred care. Journal for specialists in pediatric nursing : JSPN. 2015;20(4):232-43. Epub 2015/07/22.
39. Leavy P, Violeta Prina M, Martinez Caceres MJ, Bauer G. Social representations of premature birth from the perspective of individuals born preterm in the 1990s. Archivos argentinos de pediatria. 2015;113(1):6-11. Epub 2015/01/27.
40. Short EJ, Klein NK, Lewis BA, Fulton S, Eisengart S, Kercsmar C, et al. Cognitive and academic consequences of bronchopulmonary dysplasia and very low birth weight: 8-year-old outcomes. Pediatrics. 2003;112(5):e359. Epub 2003/11/05.
41. Armentrout D. Living with grief following removal of infant life support: parents' perspectives. Critical care nursing clinics of North America. 2009;21(2):253-65. Epub 2009/05/23.

Confidential: For Review Only

42. Sharek PJ, Baker R, Litman F, Kaempf J, Burch K, Schwarz E, et al. Evaluation and development of potentially better practices to prevent chronic lung disease and reduce lung injury in neonates. Pediatrics. 2003;111(4 Pt 2):e426-31. Epub 2003/04/03.
43. Hanrahan K, Gates M, Attar MA, Lang SW, Frohna A, Clark SJ. Neonatal back transport: perspectives from parents of Medicaid-insured infants and providers. Neonatal network : NN. 2007;26(5):301-11. Epub 2007/10/12.
44. Reyna BA, Pickler RH, Thompson A. A descriptive study of mothers' experiences feeding their preterm infants after discharge. Advances in neonatal care : official journal of the National Association of Neonatal Nurses. 2006;6(6):333-40. Epub 2007/01/09.
45. Chollat C, Enser M, Houivet E, Provost D, Benichou J, Marpeau L, et al. School-age outcomes following a randomized controlled trial of magnesium sulfate for neuroprotection of preterm infants. The Journal of pediatrics. 2014;165(2):398-400 e3. Epub 2014/05/20.
46. Cescutti-Butler L. Eliciting parental views regarding early discharge to home care for premature infants. Infant. 2009;5(1):23-8.
47. Catlin A. Extremely long hospitalizations of newborns in the United States: data, descriptions, dilemmas. Advances in neonatal care : official journal of the National Association of Neonatal Nurses. 2008;8(2):125-32. Epub 2008/04/18.
48. Gaíva AM, Ferriani GC. Prematurity: children and family experience. Acta Paulista de Enfermagem. 2001;14(1):17-28.
49. Malhotra Y, Nzegwu N, Harrington J, Ehrenkranz RA, Hafler JP. Identifying Barriers to Initiating Minimal Enteral Feedings in Very Low-Birth-Weight Infants: A Mixed Methods Approach. Am J Perinat. 2016;33(1):47-56.
50. Holditch-Davis D, Bartlett TR, Blickman AL, Miles MS. Posttraumatic stress symptoms in mothers of premature infants. Journal of obstetric, gynecologic, and neonatal nursing : JOGNN. 2003;32(2):161-71. Epub 2003/04/11.
51. Stevens EE, Gazza E, Pickler R. Parental Experience Learning to Feed Their Preterm Infants. Adv Neonat Care. 2014;14(5):354-61.
52. Ahern K. What neonatal intensive care nurses need to know about neonatal palliative care. Advances in neonatal care : official journal of the National Association of Neonatal Nurses. 2013;13(2):108-14. Epub 2013/03/28.
53. Rasmussen LA, Bell E, Racine E. A Qualitative Study of Physician Perspectives on Prognostication in Neonatal Hypoxic Ischemic Encephalopathy. Journal of child neurology. 2016;31(11):1312-9. Epub 2016/07/06.
54. Wielenga JM, Tume LN, Latour JM, van den Hoogen A. European neonatal intensive care nursing research priorities: an e-Delphi study. Archives of disease in childhood Fetal and neonatal edition. 2015;100(1):F66-71. Epub 2014/09/28.
55. Chang KWC, Austin A, Yeaman J, Phillips L, Kratz A, Yang LJS, et al. Health-Related Quality of Life Components in Children With Neonatal Brachial Plexus Palsy: A Qualitative Study. Pm&R. 2017;9(4):383-91.
56. Hefferman P, Heilig S. Giving "moral distress" a voice: ethical concerns among neonatal intensive care unit personnel. Cambridge quarterly of healthcare ethics : CQ : the international journal of healthcare ethics committees. 1999;8(2):173-8. Epub 1999/03/06.
57. Miracle DJ, Meier PP, Bennett PA. Mothers' decisions to change from formula to mothers' milk for very-low-birth-weight infants. Journal of obstetric, gynecologic, and neonatal nursing : JOGNN. 2004;33(6):692-703. Epub 2004/11/25.
58. Rohsiswatmo R, Rafika S, Marsubrin PM. Prevention and control of blood stream infection using the balanced scorecard approach. Acta medica Indonesiana. 2014;46(3):209-16. Epub 2014/10/29.
59. Jirapaet V, Jirapaet K, Sopajaree C. The nurses' experience of barriers to safe practice in the neonatal intensive care unit in Thailand. Journal of obstetric, gynecologic, and neonatal nursing : JOGNN. 2006;35(6):746-54. Epub 2006/11/16.

Confidential: For Review Only

60. Jones L, Taylor T, Watson B, Fenwick J, Dordic T. Negotiating Care in the Special Care Nursery: Parents' and Nurses' Perceptions of Nurse-Parent Communication. Journal of pediatric nursing. 2015;30(6):e71-80. Epub 2015/05/03.
61. Griffin T, Kavanaugh K, Soto CF, White M. Parental evaluation of a tour of the neonatal intensive care unit during a high-risk pregnancy. Journal of obstetric, gynecologic, and neonatal nursing : JOGNN. 1997;26(1):59-65. Epub 1997/01/01.
62. Brinchmann BS, Nortvedt P. Ethical decision making in neonatal units--the normative significance of vitality. Medicine, health care, and philosophy. 2001;4(2):193-200. Epub 2001/09/08.
63. Green J, Darbyshire P, Adams A, Jackson D. Desperately seeking parenthood: neonatal nurses reflect on parental anguish. Journal of clinical nursing. 2015;24(13-14):1885-94. Epub 2015/05/06.
64. Brinchmann BS. 'They have to show that they can make it': vitality as a criterion for the prognosis of premature infants. Nursing ethics. 2000;7(2):141-7. Epub 2000/09/15.
65. Darcy AE, Hancock LE, Ware EJ. A descriptive study of noise in the neonatal intensive care unit. Ambient levels and perceptions of contributing factors. Advances in neonatal care : official journal of the National Association of Neonatal Nurses. 2008;8(3):165-75. Epub 2008/06/07.
66. Lee SY, Lee KA, Rankin SH, Alkon A, Weiss SJ. Acculturation and stress in Chinese-American parents of infants cared for in the intensive care unit. Advances in neonatal care : official journal of the National Association of Neonatal Nurses. 2005;5(6):315-28. Epub 2005/12/13.
67. Sisk P, Quandt S, Parson N, Tucker J. Breast Milk Expression and Maintenance in Mothers of Very Low Birth Weight Infants: Supports and Barriers. J Hum Lact. 2010;26(4):368-75.
68. Miljeteig I, Norheim OF. My job is to keep him alive, but what about his brother and sister? How Indian doctors experience ethical dilemmas in neonatal medicine. Developing world bioethics. 2006;6(1):23-32. Epub 2006/01/27.
69. Thoren EM, Metze B, Buhrer C, Garten L. Online support for parents of preterm infants: a qualitative and content analysis of Facebook 'preemie' groups. Archives of disease in childhood Fetal and neonatal edition. 2013;98(6):F534-8. Epub 2013/06/14.
70. Snowdon C, Garcia J, Elbourne D. Making sense of randomization; Responses of parents of critically ill babies to random allocation of treatment in a clinical trial. Social Science & Medicine. 1997;45(9):1337-55.
71. Hentschel R, Lindner K, Krueger M, Reiter-Theil S. Restriction of ongoing intensive care in neonates: A prospective study. Pediatrics. 2006;118(2):563-9.
72. Garcia J, Elbourne D, Snowdon C. Equipoise: a case study of the views of clinicians involved in two neonatal trials. Clinical trials (London, England). 2004;1(2):170.
73. Snowdon C, Brocklehurst P, Tasker R, Platt MW, Harvey S, Elbourne D. Death, Bereavement and randomised controlled trials (BRACELET): a methodological study of policy and practice in neonatal and paediatric intensive care trials. Health Technol Asses. 2014;18(42):1-+.
74. Moyer VA, Singh H, Finkel KL, Giardino AP. Transitions from neonatal intensive care unit to ambulatory care: description and evaluation of the proactive risk assessment process. Qual Saf Health Care. 2010;19:I26-I30.
75. Turrill S. A focus of care for neonatal nursing: the relationship between neonatal nursing practice and outcomes. Part 2. Paediatric Nursing. 2003;15(5):30.
76. Koh TH, Butow PN, Coory M, Budge D, Collie LA, Whitehall J, et al. Provision of taped conversations with neonatologists to mothers of babies in intensive care: randomised controlled trial. BMJ. 2007;334(7583):28. Epub 2006/12/05.
77. Kavanaugh K. Parents' experience surrounding the death of a newborn whose birth is at the margin of viability. Journal of obstetric, gynecologic, and neonatal nursing : JOGNN. 1997;26(1):43-51. Epub 1997/01/01.

Confidential: For Review Only

78. Ericson J, Flacking R. Estimated breastfeeding to support breastfeeding in the neonatal intensive care unit. Journal of obstetric, gynecologic, and neonatal nursing : JOGNN. 2013;42(1):29-37. Epub 2012/11/28.
79. Chia P, Sellick K, Gan S. The attitudes and practices of neonatal nurses in the use of kangaroo care. Aust J Adv Nurs. 2006;23(4):20-7.
80. Saigal S, Stoskopf BL, Feeny D, Furlong W, Burrows E, Rosenbaum PL, et al. Differences in preferences for neonatal outcomes among health care professionals, parents, and adolescents. Jama-J Am Med Assoc. 1999;281(21):1991-7.
81. Janvier A, Farlow B, Baardsnes J, Pearce R, Barrington KJ. Measuring and communicating meaningful outcomes in neonatology: A family perspective. Seminars in perinatology. 2016;40(8):571-7. Epub 2016/10/30.
82. Staub K, Baardsnes J, Hébert N, Hébert M, Newell S, Pearce R. Our child is not just a gestational age. A first-hand account of what parents want and need to know before premature birth. Acta Paediatrica. 2014;103:1035-8.
83. Pearce R, Baardsnes J. Term MRI for small preterm babies: do parents really want to know and why has nobody asked them? Acta Paediatrica. 2012;101(10):1013-5.
84. Charles C, Gafni A, Whelan T. Shared decision-making in the medical encounter: What does it mean? (Or it takes at least two to tango). Social Science & Medicine. 1997;44(5):681-92.
85. Gardner W, Kelleher KJ. Core Quality and Outcome Measures for Pediatric Health. JAMA pediatrics. 2017;171(9):827-8.
86. de Wit M, Abma T, Koelewijn-van Loon M, Collins S, Kirwan J. Involving patient research partners has a significant impact on outcomes research: a responsive evaluation of the international OMERACT conferences. BMJ open. 2013;3(5).
87. INVOLVE. Public involvement in research: values and principles framework. Eastleigh: INVOLVE, 2015.
88. Saigal S, Doyle LW. An overview of mortality and sequelae of preterm birth from infancy to adulthood. Lancet. 2008;371(9608):261-9. Epub 2008/01/22.
89. Muller-Nix C, Forcada-Guex M, Pierrehumbert B, Jaunin L, Borghini A, Ansermet F. Prematurity, maternal stress and mother-child interactions. Early Hum Dev. 2004;79(2):145-58.
90. Hall EO. A double concern: grandmothers' experiences when a small grandchild is critically ill. Journal of pediatric nursing. 2004;19(1):61-9. Epub 2004/02/14.

Confidential: For Review Only

Confidential: For Review Only

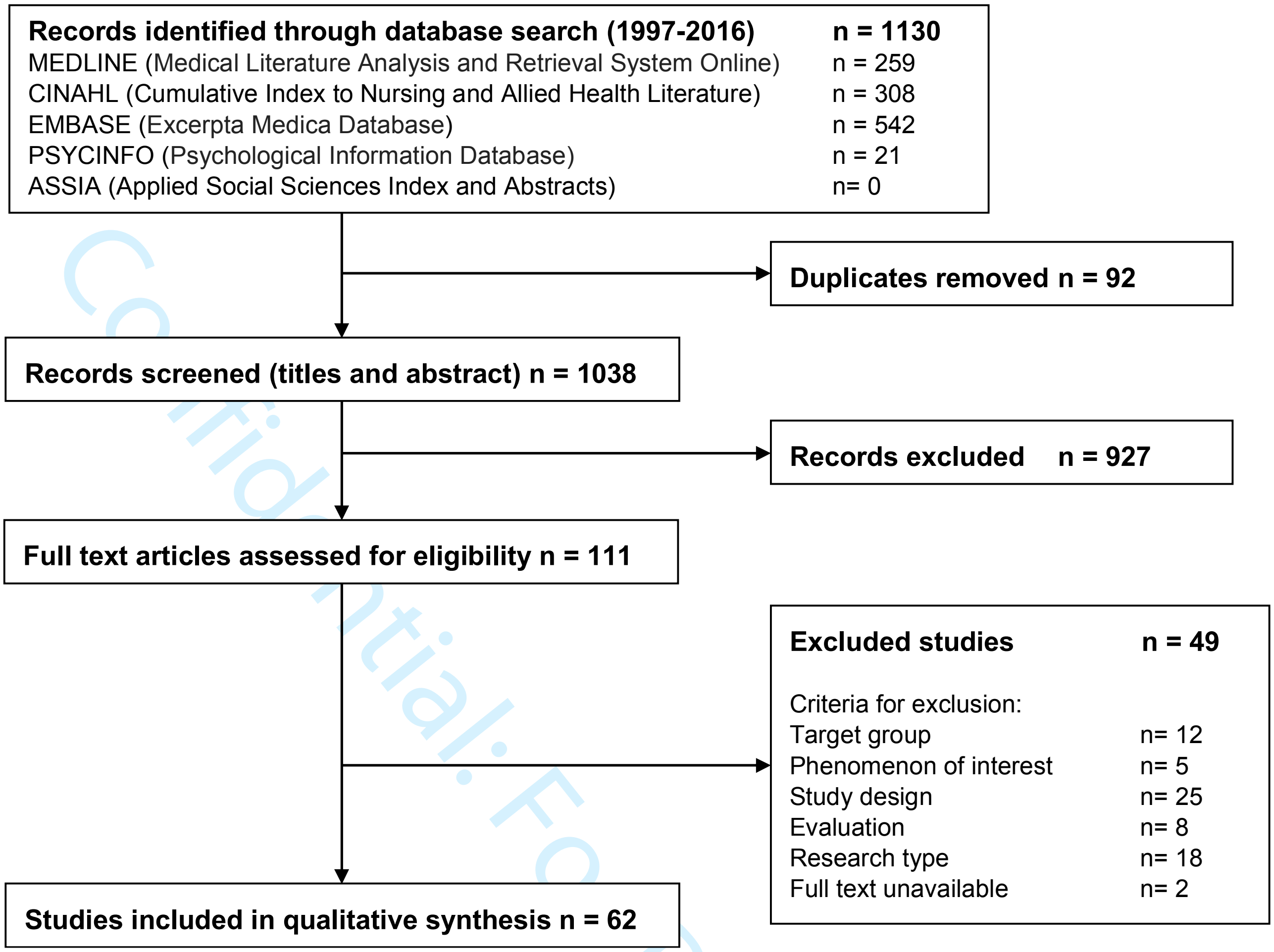

Confidential: For Review Only

Online only supplemental material

eTable 1: Eligiblity criteria
eFigure 1: Search strategy
eTable 2: Summary of included studies
eTable 3: List of all outcomes
eTable 4: Outcomes within each study
eFigure 2: Results of permutation test analysis regarding gestational age
eFigure 3: Results of permutation test analysis regarding stakeholder group

Confidential: For Review Only

eTable 1: Eligibility Criteria

Sample: Babies of any gestational age at birth receiving care in a neonatal unit.

Phenomenon of Interest: Care on a Neonatal Intensive Care Unit or Special Care Baby Unit. Care provided exclusively on labour or postnatal wards or in an outpatient/community environment was not included.

Design: Qualitative research (including phenomenology, ethnography, case studies and grounded theory) was included. Quantitative research was included if qualitative data had been gathered (e.g. surveys developed with parent or caregiver input).

Evaluation: Data relating to outcomes that former patients, parents or healthcare professionals feel are important.

Research type: Qualitative research or mixed methods research was included. Research that was exclusively quantitative was excluded.

Confidential: For Review Only

eFigure 1: Search Strategy

The following search strategy was utilised to search the Medline database:

1. Medline; neonat*.ti,ab;
2. Medline; exp INFANT, NEWBORN/;
3. Medline; ((preterm OR "pre term" OR prematur*) AND (infan* OR neonat* OR baby OR babies)).ti,ab;
4. Medline; (infant* adj2 premature).af;
5. Medline; exp NEONATOLOGY/;
6. Medline; exp INTENSIVE CARE UNITS, NEONATAL/;
7. Medline; exp INTENSIVE CARE, NEONATAL/;
8. Medline; ("intensive care" adj2 neonat*).ti,ab;
9. Medline; NICU.ti,ab;
10. Medline; SCBU.ti,ab;
11. Medline; "special care baby unit*".ti,ab;
12. Medline; INFANT, PREMATURE, DISEASES/;
13. Medline; 1 OR 2 OR 3 OR 4 OR 5 OR 6 OR 7 OR 8 OR 9 OR 10 OR 11 OR 12;
14. Medline; (treatment adj2 outcome*).ti,ab;
15. Medline; exp TREATMENT OUTCOME/;
16. Medline; "key outcome*".ti,ab;
17. Medline; (important adj2 outcome*).ti,ab;
18. Medline; exp PATIENT OUTCOME ASSESSMENT/;
19. Medline; (outcome* adj2 set).ti,ab;
20. Medline; exp "OUTCOME ASSESSMENT (HEALTH CARE)"/;
21. Medline; "outcome assessment*".ti,ab;
22. Medline; (core adj2 outcome*).ti,ab;
23. Medline; "outcome measure*".ti,ab;
24. Medline; 14 OR 15 OR 16 OR 17 OR 18 OR 19 OR 20 OR 21 OR 22 OR 23;
25. Medline; ("semi-structured" OR semistructured OR unstructured OR informal OR "indepth" OR indepth OR "face-to-face").ti,ab;
26. Medline; ((structured OR guide) adj3 (interview* OR discussion*)).ti,ab;
27. Medline; ("focus group*" OR qualitative OR ethnograph* OR fieldwork OR "field work" OR "key informant*").ti,ab;
28. Medline; exp INTERVIEWS AS TOPIC/;
29. Medline; exp FOCUS GROUPS/;
30. Medline; exp NARRATION/;
31. Medline; exp QUALITATIVE RESEARCH/;
32. Medline; 25 O 26 OR 27 OR 28 OR 29 OR 30 OR 31 OR 32;
33. Medline; 13 AND 24 AND 32;

Confidential: For Review Only

eTable 2: Summary Of Included Studies

Source	Country	Gestational age of neonates involved	Birthweight of neonates involved	Methodology	Stakeholders (numbers and further details given in brackets where available)
Ahern (2013)	Australia	Unspecified	Unspecified	Questionnaire (Delphi); Interviews (individual)	Doctors; Nurses; Others (social worker, clinical educator)
Armentrout (2009)	USA	Term; Preterm; Extreme preterm	Not specified	Interviews (individual); Interviews (group)	Parents (11 mothers, 4 fathers)
Arruda et al. (2010)	Brazil	Preterm; Extreme preterm	Very low	Interviews (individual); Interviews (group	Parents (8 mothers); Other family members (1 maternal grandmother)
Brinchmann (2000)	Norway	Preterm; Extreme preterm	Unspecified	Interviews (unspecified size); Direct observation	Doctors (8); Nurses (10);
Brinchmann et al. (2001)	Norway	Unspecified	Unspecified	Interviews (unspecified size); Direct observation	Doctors (8); Nurses (10); Parents (20)
Brødsgaard (2015)	Denmark	Preterm; Extreme preterm	Normal; Low; Very low; Extremely low	Questionnaire; Interviews (group)	Parents (15)
Callen et al. (2005)	Canada	Preterm; Extreme preterm	Very low; Extremely low	Direct observation	Parents (64 mothers)
Catlin (2008)	USA	Term; Extreme preterm	Normal; Low; Very low; Extremely low	Questionnaire	Doctors (42); Nurses (291); Others (4 social workers and 76 unspecified)
Cescutti-Butler (2009)	UK	Unspecified	Unspecified	Interviews (unspecified size)	Parents (4 mothers, 1 father)
Chang (2014)	USA	Unspecified	Unspecified	Interviews (group)	Doctors; Nurses; Other allied healthcare professionals (occupational therapist); Former neonatal patients (13); Parents (23 mothers, 5 fathers); Other family members (1 grandparent, 2 unspecified); Others (social worker, clinical co-ordinator)
Chia (2006)	Australia	Unspecified	Unspecified	Questionnaire; Interviews (unspecified size)	Nurses (34)
Chollat (2014)	France	Preterm; Extreme preterm	Low; Very low	Questionnaire	Parents (431)
Darcy (2008)	USA	Unspecified	Unspecified	Interviews (unspecified size)	Nurses (6)
Docherty (2007)	USA	Term; Preterm	Unspecified	Interviews (individual)	Parents (5 mothers)
Ericson (2013)	Sweden	Preterm; Extreme preterm	Low	Questionnaire	Nurses (45)
Gaíva (2001)	Brazil	Preterm	Unspecified	Questionnaire	Parents (11 mothers)
Garcia (2004)	UK	Term; Preterm	Unspecified	Interview (individual)	Doctors (30)
Gontijo (2008)	Brazil	Preterm	Low	Questionnaire; Direct observation	Other allied healthcare professionals (19 unspecified); Parents (13 mothers);
Green (2015)	Australia	Extreme preterm	Unspecified	Questionnaire; Interviews (individual); Interviews (group)	Nurses (414)
Gregory (2012)	USA and Canada	Preterm	Unspecified	Questionnaire	Nurses (70)
Griffin (1997)	USA	Preterm; Extreme preterm	Unspecified	Interviews (unspecified size)	Parents (6 mothers, 2 fathers); Others (5 parents of infants not admitted to NICU)
Hanrahan (2007)	USA	Unspecified	Unspecified	Interviews (group)	Doctors (12); Nurses (11); Parents (10 mothers, 2 fathers)
Hefferman (1999)	USA	Extreme Preterm	Unspecified	Questionnaire	Doctors (3); Nurses (27); Other allied healthcare professionals (2 nurse managers, 1 respiratory care supervisor)
Hentschel (2006)	Germany	Term; Preterm	Unspecified	Direct observation	Doctors; Nurses; Parents (31)
Holditch-Davis (2003)	USA	Preterm	Normal; Low; Very low	Interviews (unspecified size)	Parents (30 mothers)
Hussey-Gardner (1998)	USA	Preterm	Very low	Interviews (unspecified size); Direct observation; Other (artefact collection)	Parents (7 mothers, 4 fathers); Other family members (3 grandmothers)
Jirapaet (2006)	Thailand	Unspecified	Unspecified	Interviews (individual)	Nurses (27)
Johnson (2007)	USA	Preterm	Very low	Interviews (unspecified size)	Parents (18 mothers)
Jones (2015)	Australia	Preterm; Extreme preterm	Normal; Low; Very low; Extremely low	Interviews (individual); Interviews (group)	Nurses (12); Parents (27 mothers, 5 fathers)
Kavanaugh (1997)	USA	Unspecified	Extremely low	Interviews (individual)	Parents (5 mothers, 3 fathers)
Koh (2007)	Australia	Preterm	Low	Questionnaire; Interviews (individual)	Parents (200 mothers)
Leavy (2015)	Argentina	Preterm	Very low	Interviews (group)	Former neonatal patients (59)
Lee (2005)	USA	Preterm	Unspecified	Questionnaire; Interviews (unspecified)	Parents (30 mothers, 25 fathers)
Lou (2009)	Denmark	Extreme preterm	Very low	Interviews (individual)	Parents (11 mothers, 9 fathers)
Malhotra (2015)	USA	Unspecified	Very low	Interviews (unspecified size); Direct observation	Doctors (10); Nurses (16)
Miljeteig (2006)	India	Term; Preterm	Unspecified	Interviews (individual); Direct observation	Doctors (14); Nurses (8)
Miracle (2004)	USA	Preterm; Extreme preterm	Very low; Extremely low	Interviews (individual)	Parents (21 mothers)
Moyer (2010)	USA	Unspecified	Unspecified	Interviews (unspecified size); Direct observation; Other (Health care failure mode and effect analysis)	Doctors; Nurses; Parent (1); Others (discharge planners, social worker)
Orfali (2004)	USA	Preterm; Extreme preterm	Low; Very low	Interviews; Direct observation	Doctors (60); Parents (71)
Rasmussen (2016)	Canada	Unspecified	Unspecified	Interviews (individual)	Doctors (12)
Reyna (2006)	USA	Preterm	Unspecified	Interview (size unspecified)	Parents (27 mothers)

Confidential: For Review Only

Rohsiswatmo (2014)	Indonesia	Unspecified	Low; Very low	Questionnaire; Interviews (group); Observation; Other (operational research)	Other allied healthcare professionals (unspecif
Saigal (1999)	Canada	Unspecified	Normal; Extremely low	Questionnaire; Interviews (individual)	Doctors (100); Nurses (103); Former neonatal (264); Parents (275)
Saunders (2003)	USA	Unspecified	Very low	Interviews (group); Other (Internal process analysis, Collaborative learning)	Doctors; Nurses; Other allied healthcare profes (respiratory therapists, pharmacists, physical a occupational therapists) Parents (76); Others (secretaries, social workers)
Sawyer (2014)	UK	Preterm	Unspecified	Questionnaire; Interviews (individual); Interviews (group)	Parents (145 mothers, 85 fathers)
Schneiderman (1997)	USA	Unspecified	Unspecified	Interviews (individual)	Other allied healthcare professionals (22 Emer department directors, 43 NICU directors, 43 Et committee chairpeople)
Sharek (2003)	USA	Unspecified	Low; Very low; Extremely low	Interviews (group)	Other allied healthcare professionals (unspecif
Short (2003)	USA	Term; Preterm; Extreme preterm	Normal; Very low	Questionnaire; Interviews (unspecified size)	Parents; Other (teachers)
Sisk (2010)	USA	Preterm; Extreme preterm	Low; Very low; Extremely low	Interviews (individual)	Parents (32 mothers)
Smith (2006)	USA	Preterm; Extreme preterm	Unspecified	Interviews (individual)	Parents (16 fathers)
Snowdon (1997)	UK	Term	Unspecified	Interviews (unspecified size)	Parents (21 mothers, 16 fathers)
Snowdon (2014)	UK	Term; Preterm; Extreme preterm	Normal; Low; Very low; Extremely low	Interviews (individual)	Other allied healthcare professionals (51 trial te members); Parents (51)
Squitieri (2013)	USA	Unspecified	Unspecified	Questionnaire; Interviews (individual)	Former neonatal patients (18); Parents (18)
Stevens (2014)	USA	Preterm; Extreme preterm	Low; Very low; Extremely low	Interviews (individual)	Parents (12 mothers, 8 fathers)
Thoren (2013)	Germany	Preterm	Unspecified	Other (Evaluation of Facebook groups and discussion content)	Parents; Other (representatives of online group
Thoyre (2000)	USA	Unspecified	Very low	Interviews (individual)	Parents (22 mothers)
Turrill (2003)	UK	Unspecified	Unspecified	Interviews (unspecified size)	Nurses (6); Other allied healthcare professiona clinical services manager)
Turrill (2003)	UK	Unspecified	Unspecified	Interviews (unspecified size); Direct observation	Nurses
van Zuuren (2006)	Netherlands	Unspecified	Unspecified	Interviews (individual); Direct observation	Doctors (5); Nurses (6); Others (1 social worke pastoral care worker)
Vandenbussche (1999)	Netherlands	Unspecified	Unspecified	Questionnaire	Doctors (12); Parents (30 mothers)
Votteler (2005)	USA	Unspecified	Unspecified	Questionnaire; Interviews (individual)	Former neonatal patients (14); Parents; Other f members
Wielenga (2015)	Netherlands	Unspecified	Unspecified	Questionnaire (Delphi)	Nurses; Other allied healthcare professionals (

Confidential: For Review Only

eTable 3: Outcomes Identified

Outcome Domain Category	Outcome Domain	Outcome
Organ system	Respiratory	Asthma
		Breathlessness
		Chronic Lung Disease
		Excessive secretions
		Frequent respiratory illnesses
		Mechanical ventilation
		Nasal congestion
		Oxygen dependence
		Pneumothorax
	Cardiovascular	Hypotension
		Presence of Patent Ductus Arteriosus
	Gastrointestinal	Breastfeeding
		Choice of milk for feeding
		Choking during feeding
		Eating disorder
		Feeding difficulties
		Feeding practices
		Fistulas
		Frequency of defaecation
		Initiating enteral feeds
		Liver failure
		Necrotising enterocolitis
		Nutritional intake
		Oral feeding
		Other gastrointestinal malformations
		Regurgitation
		Short gut syndrome
	Neurological	Brain damage (not further specified)
		Neurological care
		Neurological symptoms
		Seizures
		Significant IVH
		Sleep disorders
	Genitourinary	Urological disorders
	Infection	Prevention of infection
		Sepsis
		Susceptibility to infection
	Skin	Appearance of scars
		Burns
		Extravasation injuries

Confidential: For Review Only

		Pressure sores
		Skin care
	Surgical	Appearance of scars
		Care for surgical babies
		Need for ileostomy
		Need for multiple operations
	Developmental	Ability to feed themselves
		Ability to undertake sport
		Ability to walk
		Difficulties with activities of daily living
		Hearing impairment
		Issues of development and motor skills
		Language disorders
		Need for physical therapy
		Normal hearing
		Retinopathy of prematurity
		Social difficulties
		Visual impairment
Holistic	Normality	Ability to lead a normal life
		Being treated normally
		Inability to create a normal life
		Normal health
		Normality
		Thriving
	Suffering	Comfort
		Ex-patients separation from their suffering
		Symptom control
		Suffering
	Survival	Survival
		Survival with disability
		Survival without disability
	Pain	Chronic pain
		Pain
		Pain management
	Growth	Growth
	Other holistic outcomes	Overall Health State
		Physical appearance
		Physiological stability
		Vitality
Parent focussed	Parental support	Balancing caring for themselves and their baby
		Barriers to parental involvement
		Care provided close to home
		Coping with maternal illness
		Culture differences

Confidential: For Review Only

		Maintaining hope
		Online support
		Parental ability to work
		Parental competence
		Parental involvement
		Preparation for NICU admission
		Support from faith
		Support from family and friends
		Support from fathers
		Support from healthcare professionals
	Other parent focussed outcomes	Long term effects on parents
		Parental perception of uncertainty
		Support beyond NICU
Healthcare delivery	Healthcare workers- Communication	Allowing time for conversation
		Awareness of parental views
		Candour
		Communicating in challenging settings
		Communicating information effectively
		Communication about discharge
		Communication with ex-neonatal patients
		Communication with parents
		Developing a caring relationship
		Keeping parents informed
		Language barrier
		Treating parents with respect
		Using aids to communication
	Healthcare workers- Knowledge and Competence	Consistency of decisions
		Ethical decision making
		Expertise in palliative care
		Healthcare professional competence
		Healthcare professionals behaviour
		Identifying who is responsible for care
		Medical errors
		Staff insecurity
		Staffing levels
		Iatrogenic harm
	Other healthcare delivery outcomes	Inclusion in research
Economic	Healthcare utilisation	Frequent appointments
		Frequent readmissions
		Inappropriate treatments
		Need for frequent treatments
		Need for lifelong care
		Recurrent sickness
	Other economic outcomes	Duration of admission

Confidential: For Review Only

		Healthcare resources
Social	Psychiatric	Autism
		Behavioural disturbances
		Dyslexia
		Mood disorders
		Need for educational support
		Psychiatric disorder
	Relationships with others	Bonding with family and friends
		Bonding with parents
		Childhood happiness
		Effects on family and friends
		Family resources
		Overprotective parent-child relationship
		Peer acceptance
		Psychological coping
	Other social outcomes	Schooling
		Self-identifying as premature

Confidential: For Review Only

eTable 4: Outcomes Within Each Study

Source	Outcome Domains and outcomes
Ahern (2013)	Respiratory<br>Breathlessness: *Education priorities: Managing symptoms (eg, secretions, breathlessness) in a dying baby*<br>Excessive secretions: *Education priorities: Managing symptoms (eg, secretions, breathlessness) in a dying baby*<br>Gastrointestinal<br>Feeding Difficulties: *Education priorities: Managing feeding in a dying baby*<br>Parental Support<br>Support from Healthcare Professionals: *Educational priorities identified included: How to provide emotional support to grieving parents Helping parents say "goodbye" Supporting parents when they are at different stages of grief Identifying and supporting families at high risk of complicated grief*<br>Healthcare workers-Communication<br>Communicating in challenging settings: *Education priorities: Communicating with parents of palliative neonates Educational priorities identified included: Knowing what to say; using the right words Communicating with parents about the palliative care plan Initiating difficult conversations*<br>Pain<br>Pain: *Educational priorities identified included: Assessing and managing pain in a dying baby*<br>Suffering<br>Symptom control: *Managing symptoms Educational priorities identified included: Managing symptoms (eg, secretions, breathlessness) in a dying baby*
Armentrout<br><br>(2009)	Development-Special Senses<br>Normal hearing:*"She got to hear her dad sing,"*<br>Parental Support<br>Support from healthcare professionals:*"the nursing staff, the doctors...they really know what they're doing...not only medically, but dealing with us personally...that helped a lot."*<br>Healthcare workers-Communication<br>Developing a caring relationship: *"such a great blessing...that there was no hindrance in the terms of any kind of an attitude or anything with the nurse or the attending doctor that made us feel uncomfort-able or gave us any additional grief."*<br>*"the nursing staff, the doctors they really know what they're doing not only medically, but dealing with us personally that helped a lot."*<br>Healthcare workers-Knowledge and Competence<br>Healthcare professional competence: *"the nursing staff, the doctors they really know what they're doing not only medically, but dealing with us personally that helped a lot."*<br>Relationships with others<br>Bonding with parents: *"She got to hear her dad sing, she got to...she knew we were holding her"*<br>*"my feeling is, if your child has to die, what better place than in mom's arms"*<br>Bonding with family and friends: *"The only thing we might have done...some of our closest friends...it would have been nice to have them there as well...none of our friends got to see or meet him while he was alive."*

Confidential: For Review Only

	"I wanted them to spend time with her, hold her, and bathe her and do whatever" being able to have their infant with them as part of a family was something that all parents proclaimed they "would never replace for anything in the world."<br>Effects on family and friends: *"I guess I feel like I lost about a year of my son's time. He turned 3 shortly after my daughter died, and it wasn't until he was a little after 4 that I turned around in the kitchen and saw this little boy and said "Who are you?" I wasn't all there for about a year and this little boy, even though he was the one who got me through it I really didn't know who he was."*<br>Suffering<br>Suffering: *"He was just suffering, and we didn't want him to suffer." "not withdrawing support would be... "some-thing to satisfy my empty arms."*<br>Normality<br>Normality: *"Personally my only regret was that I didn't bathe him" "And the baptism...why didn't we do it sooner...why did we wait for somebody on call to show up with a bottle of sterile water and a cotton ball...why didn't we do it the right way?"*<br>*"I think it's important to allow yourself to be a mother before you let go...of the baby...to be a parent to some degree...to change their diaper...feed 'em if you can...play with their toes and fingers and nose and...just be a mother to 'em...be a parent to 'em...before you turn it off, because that...is something that you will cherish for the rest of your life."*<br>*For some it was their only opportunity to hold and talk to their infant like "a normal baby without all the tubes and wires."*<br>Other<br>Support beyond NICU: *"the medical professionals who have become your support system and your family while your child was alive...all of a sudden they're gone...I think it's important to have some kind of support system or information...otherwise it's like stepping into this huge abyss."*<br>Long term effects on parents: *"would get up in the morning I would be normal after about 2 weeks, 3 weeks act normally take the little one to school, stay in the car for a while come back home and get into bed for the next 6 hours and cry,"*<br>*"The milestone of a year was really bad. And it is still bad. I mean I'm functioning. I put on make-up. I get dressed. I go out. I look normal but it lasts for a long time the bad thing."*
Arruda et al.<br><br>(2010)	Respiratory<br>Frequent respiratory illnesses: *all concerned and involved with numerous hospitalizations resulting from respiratory failure "He had a lot of pneumonia up to two years, was direct in hospital"* Need for supplemental oxygen: *"Even the three years he was in direct oxygen"*<br>*Need for ongoing respiratory therapy "She has long respiratory therapy"*<br>Neurological<br>Seizures: *"I did not know he could have seizures. He started to drool, the twitch of the hand fingers, vomited, turned the eye and everything"*<br>Brain damage (not further specified): *"In tomography and magnetic resonance given spot in the brain. According to the doctor, these spots were the cardiac arrest he had in the NICU, the lack of oxygen." "When I took the pediatrician, she suspected hydrocephalus because the head circumference is growing very fast. Hence, the neuro said no, that his brain had suffered a lot, but would recover."*<br>Sleep disorder: *They had a lot of irritability, change in posture, sleep, among others*<br>Development-Gross motor<br>Ability to walk *"He did not walk as a baby, just dragged." "He walked four, my mother never forgot, she says it was a miracle of God"*<br>Development-Special senses<br>Visual impairment *"The first thing I noticed at two years was he, all he would see closer eye toy [...] was all close, there came the glasses"*

Confidential: For Review Only

	"I took the ophthalmologist with less than a year, he wears glasses since baby"<br>*"We discovered his problem because one day was passing a plane and we show and he did not see"*<br>Development-Speech and Social<br>Social difficulties: *"It does not seem that he is seven years old. So I think so, the teacher says he wants to play under the table, his actions are different, in front of people. People treat different, it feels different"*<br>Language disorders: *"how she learned to give kiss? He began to speak phonemes? Everything was the stimulus that we gave"*<br>Parental Support<br>Parental competence: *"In the first months after he left the ICU, everything was already appearing: it was too much crying, irritation, they were not normal, as I had no knowledge ... I did not know and then went back to seek this knowledge"*<br>Healthcare utilisation<br>Frequent readmissions: *all concerned and involved with numerous hospitalizations resulting from respiratory failure*<br>Psychiatric<br>Behavioural disturbances: *They had a lot of irritability, change in posture, sleep, among others "because she did not behave like another child of her age, and I had three children, knew there was nothing right ... The difference was crying, irritation were not normal."*<br>*"Because she did not act like a child of her age, because beyond being premature, I did not deal with it in the crying, agitation."*<br>Need for educational support: *"Then I went behind the neuro pediatrician in Maringa, then came the diagnosis of hydrocephalus, motor problem ... and you'll have to put your child in a special school ."*<br>Relationships with others<br>Effects on family and friends: *" One day he fell ill super the other day and did not improve, went to the doctor and she said it was convulsing, I had never seen him so ... but one thing is that my mother did not let her take medicine because still believed in God, then I do not know if that's why "*<br>Normality<br>Normality: *"Then, after two years, he took more health ... no one says that was born premature."*<br>Other<br>Physical appearance: *"all World wanted to see, because he was too small" "My husband's aunt came to say that she was like lizard, I was very sad"*
Brinchmann<br><br>(2000)	Other<br>Vitality: *"All the pictures, X-rays, ultrasounds and test results indicated that this was an infant we were just keeping alive. But he himself showed signs that he was doing all right, and so things did not hang together."*<br>*"Putting a hand over his eyes was a sign of surplus resources for him" "The doctor said that, whatever we do, however good we are, it is mostly up to the infant himself. No matter how small they are, they can have something within them-selves."*
Brinchmann et<br><br>al. (2001)	Healthcare workers-Knowledge and Competence<br>Healthcare professional competence: *The health professionals said that the ability to sense whether an infant showed vitality came with experience.*<br>Suffering<br>Comfort: *"You can almost feel what it's like in the incubator, lying on the lambskin, that it's how I would want to have laid and . . . Well, it looks very comfortable"*<br>Other

Confidential: For Review Only

	Vitality: *"They had understood that the baby was going to be small, but they had no idea how tiny he was, could not say in advance, 350g. Then the nurse who first took him in her hands after he came out said that he moved his arms, his feet, his body, and cried out, and showed a lot of strength, the moment he was born. She said she believed he could make it because he showed such vitality"*<br><br>*"There was more of a spark in her, they meant that she had more spark and seemed stronger. She had more go"*
Brødsgaard<br><br>(2015)	Gastrointestinal<br>Breastfeeding: *"I fully breastfed for four months-100%-and I am so proud of it."*<br>Development-Gross motor<br>Issues of development and motor skills: *"Issues of development and motor skills, we both looked at my child, research, experience and what I could expect."*<br>Development-Fine motor<br>Issues of development and motor skills: *"Issues of development and motor skills, we both looked at my child, research, experience and what I could expect."*<br>Parental Support<br>Parental competence: *"We learned everything we needed and knew what we had to do, I was quite comfortable when we went home." "Although initially it was scary, I developed the skills, it was a natural progression; we learned how to take care of him."*<br>Healthcare workers-Communication<br>Treating parents with respect: *"In NICU we are on the sidelines, at home we have the main role and you come and advise, [we can] clearly feel the difference."*<br>Allowing time for conversation: *"There was so much information, to take in before we could . . . know whether it [EDP] suited us. There was plenty of time to consider it really thoroughly and ask questions." "Felt very much that there was space and time,"*<br>Relationships with others<br>Bonding with family and friends: *Only when at home on EDP did they feel like a proper family. "I felt divided, torn into two pieces, which were only assembled once I got home."*<br>*In particular, families with older siblings were very enthusiastic about EDP as it had a significant positive impact on siblings' involvement and participation.*
Callen et al.<br><br>(2005)	Gastrointestinal<br>Breastfeeding: *Mother experiencing difficulty assisting infant to latch and position correctly Mother reported having a low milk volume*<br>Parental Support<br>Coping with maternal illness: *Maternally Reported Barriers to Establishing and Maintaining Breastfeeding: Mother is on medications, has poor nutrition, infected incision, high blood pressure, cold, influenza, spinal headache Mother is exhausted, tired, fatigued Mother lacks confidence, is worried, anxious, stressed, depressed, frustrated, guilty*
Catlin (2008)	Respiratory<br>Mechanical ventilation: *'This theme regarding 'Pathophysiology' described the inability of the children to breathe or eat independently. Over 30% of all infant descriptions were about babies who had tracheostomies and were unable to be weaned off a ventilator.'*<br>Gastrointestinal<br>Feeding difficulties: *This theme regarding 'Pathophysiology' described the inability of the children to breathe or eat independently....Thirty two percent of the anecdotal reports related to inability of the infants to eat, suck, swallow or absorb nutrition. Respondents to the survey*

Confidential: For Review Only

	reported nutrition categories as the most frequent reason for infants to remain hospitalized.<br>Fistulas: *Respondents to the survey reported nutrition categories as the most frequent reason for infants to remain hospitalized. One-third of all the children had a digestive issue, such as trachea-related malacias, fistulas, malformations, gut related perforations, absences, short gut and liver failures.'*<br>Liver failure: *Respondents to the survey reported nutrition categories as the most frequent reason for infants to remain hospitalized. One-third of all the children had a digestive issue, such as trachea-related malacias, fistulas, malformations, gut related perforations, absences, short gut and liver failures.'*<br>Other gastrointestinal malformations: *Respondents to the survey reported nutrition categories as the most frequent reason for infants to remain hospitalized. One-third of all the children had a digestive issue, such as trachea-related malacias, fistulas, malformations, gut related perforations, absences, short gut and liver failures.'*<br>Short gut syndrome: *Respondents to the survey reported nutrition categories as the most frequent reason for infants to remain hospitalized. One-third of all the children had a digestive issue, such as trachea-related malacias, fistulas, malformations, gut related perforations, absences, short gut and liver failures.'*<br>Development-Gross motor<br>Issues of development and motor skills: *Respondents were concerned that children were not being taught to sit, crawl or talk as would occur outside of the intensive care unit. When child life personnel were available, they were described as very helpful. They would bring in developmentally appropriate toys and teach the staff how to care for 'growing, 30 pound, NICU babies.'*<br>Development-Speech and Social<br>Language disorders: *Respondents were concerned that children were not being taught to sit, crawl or talk as would occur outside of the intensive care unit.*<br>Parental Support<br>Parental involvement: *"The little boy never left the hospital because his mother was young and single. She had no one to help with the care of this very sick boy. He ended up being in the unit 5 months longer than necessary because we had to get medical foster care to take care of him."*<br>Healthcare workers-Communication<br>Developing a caring relationship: *As the providers gave support to families, families also were described as supporting the staff....It was also difficult for the nurses and physicians to separate from the infants and families after a long period.*<br>Healthcare workers-Knowledge and Competence<br>Identifying who is responsible for care: *Respondents described an absence of review or oversight of infants with extremely long hospitalizations. Just who was supposed to make decisions about children who were extremely and chronically ill? Was there ever an end point? What was the end point? Can interventions once started ever to be stopped? Who was to determine that the burden on the child was now greater than the benefit? .*<br>Expertise in palliative care: *Nurses and doctors talked about being able to clearly recognize that a child was going to eventually die, and the used words such as 'torturing' or 'flogging the child to death' with interventions rather than providing comfort care at the end of life.*<br>Ethical decision making: *Physicians and nurses were upset when other staff members were not willing to stop technological support, even when the interventions were doing nothing to change the prognosis.*<br>Relationships with others<br>Bonding with parents: *Participants reported 78% of parents remaining involved with the child throughout the entire hospitalization, with 23% not staying involved.*

Confidential: For Review Only

	Another problem described were parents who were not able to care for such sick infants. Twenty three percent of the respondents reported parents who were either never involved or who gave up on the children.'<br>Suffering<br>Suffering: *"This infant's short life was never comfortable. He struggled just to breath, day after day, month after month without relief unless he was paralyzed and heavily sedated. I frequently felt we were torturing the child just doing daily care."*<br>*"Finally I guided his parents to let him go. I hated myself and my colleagues for putting me in this situation, a task that should have been done so many months ago. There is nothing to be proud of, just grief and tragedy all around. We did a great disservice to this child."*<br>*"Parents never accepted the diagnosis of a lethal condition. Despite several neonatologists, cardiologists and an ethics committee referral, no one had the courage to discontinue respiratory support. In the last two months has had several cardiopulmonary arrests and was resuscitated because "everyone is scared of the parents"."*<br>Normality<br>Inability to create a normal life: *"We are so busy in our ICU doing our tasks that the chronic kids are left for periods of time alone in their cribs with little interaction except mobiles."*<br>Other<br>healthcare resources: *Of note, the theme 'Cost of Care' was rarely mentioned.*<br>*Although respondents frequently discussed the emotional toll to all concerned, the monetary cost of long-term stays was very rarely (<1%) mentioned.*
Cescutti-Butler<br><br>(2009)	Respiratory<br>Oxygen dependence: *"My babies did not fit into the criteria for going home early due to one of the twins still being dependent on oxygen. However if I had had an opportunity to take them home earlier I would have jumped at it"*<br>Gastrointestinal<br>Feeding difficulties: *"We kept on saying to the staff on neonatal unit that it was only Gray's feeding that was stopping him from going home, everything else was fine" Val explained how her twins were hospitalised for three weeks and for two of those weeks there were no major problems, as during this time her babies just needed to establish feeding. "I felt particularly sensitive around the issue of "topping up" and it upset me when my babies were topped up with formula milk through tube feeds. It made me feel inadequate and a failure for not being able to provide enough milk for my babies."*<br>Parental Support<br>Barriers to parental involvement: *"I felt at times that I had to ask permission to change my baby's nappy or check that it required changing although I understood that it was to protect Gray from too much "messing about" . I did not feel that myself or my husband could open the incubator and take Gray out. We had to wait for a nurse to say "oh, you going to have a cuddle?"*<br>*"You go in every day but you still feel guilty that someone else is taking care of your babies for you even though it's not your fault"*<br>Parental involvement: *"My husband and I were fully involved in the care of our babies and felt able to question and challenge decisions"*<br>*"During our baby's stay in the neonatal unit both myself and Peter were fully involved in our son's care and did most of the caring such as nappy changing and NGT feeds"*<br>*"give the parents the opportunity to care for their babies sooner than expected with help, which to me sounds like a good plan"*<br>*"In terms of being in control-I think it was more that mostly we weren't"*<br>Support from healthcare professionals: *"The care and support from the neonatal staff was fantastic, and without this support I would not have known what I was doing or how to care for my babies"*<br>Healthcare utilisation

Confidential: For Review Only

	Duration of admission: *"When I was told I couldn't bring them home until term I was mortified because that was two months down the road and no way did I want to wait that long"*<br>Healthcare workers-Knowledge and Competence<br>Consistency of decisions: *"One member of staff indicated Christmas and another New Year for the discharge of Gray. We knew that it was only the feeding that was delaying his discharge and so we kept on asking if we could take him home tube feeding. We did not always feel involved in decisions about Gray's discharge despite us asking repeatedly if we could take him home. Just as we had resigned ourselves to being in hospital over Christmas we were suddenly told on the Monday (a week before Christmas) that I could room in that night with a view to going home either on Tuesday or Wednesday at the latest. This was very frustrating as now all of a sudden he's now allowed home!"*<br>Relationships with others<br>Effects on family and friends: *"During the eight weeks of our baby's stay in hospital my husband and I did find it difficult travelling back and forth, because we were mindful of our other child who was only six years of age at the time and we tried to maintain a sense of normality for his sake"*<br>Other<br>Physical appearance: *"My first impression of her was as "a tiny red thing"*
Chang (2014)	Neurological<br>Neurological symptoms: *I can't feel some - my left - this is numb right here.*<br>Development-Gross motor<br>Issues of development and motor skills: *I fall down easily.*<br>Development-Fine motor<br>Difficulty with activities of daily living: *It's almost to the point where she can't pick up a spoon with this hand.*<br>*You have to put the key in the ignition. So he would always have to reach around and it's very hard.*<br>Healthcare utilisation<br>Need for frequent treatments: *Like I have to miss my Friday lunches to go and sit with a teacher for the whole lunch and tell her what's happened. And like I have to I'm always getting called down to the office for another physical therapist to come and talk to me, and I'm always like it's like it's not it's like both that I have to miss school work for it, number one.*<br>Need for lifelong care: *She's been in therapy since she was 2 weeks old.*<br>Relationships with others<br>Peer acceptance: *Like this young girl telling me Friday, the other girls are getting asked out and boys won't talk to her*<br>*I've had four year olds tell me the other kids don't want to play with them cause they have a dumb arm.*<br>*It's hard for fitting in. Like kids will make remarks. Like what's that thing? Why is it like that?*<br>Bonding with family and friends: *My family is really helpful.*<br>Pain<br>Pain: *It like hurts when you wake up in the morning.*<br>Normality<br>Ability to lead a normal life: *I don't like people doing stuff for me at all.*<br>*She would spend all day trying to do one thing so she could do it herself, you know.*<br>Other<br>Physical appearance: *Cause this arm's a little bit shorter than the other one, like lengthwise.*

Confidential: For Review Only

Chia (2006)	Parental Support<br>Parental competence: *"it's a parent education thing too-explaining to them what it actually means and how they can go about doing it and how often and how long it does actually take-you need to sort of explain to parents what to wear."*<br>Healthcare workers-Knowledge and Competence<br>Healthcare professional competence: *"I think they [nurses] need to have a knowledge of it, I think that's fairly important and I am not sure that everybody does have"*<br>Relationships with others<br>Bonding with parents: *"I find it a great joy when the mums do hold the baby against their chest... irrespective of whether it's a primigravida or a multigravida. You get the same buzz out of it and so do the dads."*
Chollat (2014)	Respiratory<br>Asthma: *Table 1: -asthma*<br>Gastrointestinal<br>Eating disorders: *Table 1: -eating disorder*<br>Neurological<br>Sleep disorders: *'sleep disorder' measured*<br>Development-Gross motor<br>Issues of development and motor skills: *Neuromotor deficits CP, unable to walk or walks only with aid CP, but walks without aid No CP but dyspraxia or motor coordination disorder*<br>Development-Fine motor<br>Difficulties with activities of daily living: *(Other motor disorders were defined as coordination disorders, difficulties in cycling, drawing, cutting with scissors, playing with construction toys or puzzles, washing him/herself, dressing or tying shoes, receiving psychomotor or ergotherapy sessions.)*<br>Development-Special senses<br>Hearing impairment: *Table 1: -hearing and visual disorders were measured*<br>Visual impairment: *Table 1: -hearing and visual disorders were measured*<br>Development-Speech and Social<br>Language disorders: *Language disorders were defined by treatment by a speech-language pathologist*<br>Healthcare utilisation<br>Frequent readmissions: *Table 1: -hospital admissions were measured*<br>Psychiatric<br>Autism: *Autism, pervasive development disorders, treated with risperidone Hyperactivity or attention deficit disorder or methylphenidate medication or overall SDQ >17 or SDQ hyperactivity subscale >7 or conduct disorder as reason for a visit to a psychiatrist or a psychologist at age 11 y*<br>Need for educational support: *Has repeated a grade and/or receives/needs special support at school, has schooling difficulties, language disorder*<br>Psychiatric disorder: *Autism, pervasive development disorders, treated with risperidone Hyperactivity or attention deficit disorder or methylphenidate medication or overall SDQ >17 or SDQ hyperactivity subscale >7 or conduct disorder as reason for a visit to a psychiatrist or a psychologist at age 11 y*

Confidential: For Review Only

	Normality<br>Normal health: *Table 1: -parents perception of child's general health was measured*
Darcy (2008)	Neurological<br>Sleep disorders: *The qualitative data show that nurses are aware that increased noise levels in a NICU have detrimental physiological effects for the newborn, including alterations in sleep/wake cycles*<br>Other<br>Growth: *The qualitative data show that nurses are aware that increased noise levels in a NICU have detrimental physiological effects for the newborn...problems with appropriate growth and development.* Physiological stability: *The qualitative data show that nurses are aware that increased noise levels in a NICU have detrimental physiological effects for the newborn, including… immediate changes in vital signs,*
Docherty (2007)	Surgical<br>Need for multiple operations: *the mother also worried that there would be more surgeries*<br>Appearance of scars: *Of particular concern were the scars on Nick's body from medical procedures, surgery,*<br>Parental Support<br>Support from fathers: *Her support system was very limited; her relationship with Allison's father was sporadic and filled with conflict. Because he thought the mother had had an abortion, the father was in shock at the baby's birth and erratically provided support.*<br>Support from family and friends*: In addition, there were issues with her parents, who had custody of two of her other children. Her support network included her mother and the paternal aunt. Others in the family were not accepting of her pregnancy or the baby. The mother felt that the community blamed her for her son's illness, and this eroded her support. As one mother noted: "It would scare me at times because they, I wasn't understanding some of what they was telling me. And I'd stay upset a lot. But then my mom she would call back and she would get understanding."*<br>Barriers to parental involvement: *As with most mothers of critically ill infants, they also struggled with seeing their sick child surrounded by and dependent on technology and worried about the immediate and long-term outcomes. This made visits overwhelming and stressful.*<br>Parental involvement: *Lisa's mother felt special because of her competence in caring for her child, her feelings of responsibility for the child's well-being*<br>*Being a single parent meant that these mothers were often alone in making complex decisions for their infant during hospitalization. One mother deferred all decisions to the staff. Others struggled to learn more about the infant's health problem and became competent in making decisions.*<br>Parental ability to work: *"She's put my life on hold completely. Um, because, I could've started work at a plant that I've been tryin' to get on for the past, longest. And uh, goin' back to school is out, too."*<br>Healthcare utilisation<br>Need for frequent treatments: *the mother also worried that there would be more surgeries*<br>Healthcare workers-Communication<br>Communicating information effectively : *she became upset because she could not understand what the staff told her about the child's illness and treatments "It would scare me at times because they, I wasn't understanding some of what they was telling me. And I'd stay upset a lot. But then my mom she would call back and she would get understanding."*<br>*'Most of the mothers had limited exposure to complicated tertiary care hospitals and were challenged in communicating with highly*

Confidential: For Review Only

	educated health care providers, in understanding their infant's complex condition, and in making complex decisions related to care.'<br>Relationships with others<br>Bonding with parents: *she felt very guilty and reported difficulties attaching to her child during her hospitalizations*<br>Suffering<br>Suffering: *she reported feeling...distress at seeing him suffer*<br>Normality<br>Ability to lead a normal life: *the mother also worried that...Lisa would not have a normal life*<br>Survival<br>Survival: *the mother began worrying about whether her son would live*<br>Other<br>Physical appearance: *Of particular concern were the scars on Nick's body from medical procedures, surgery, and the total parenteral nutrition line.*
Ericson (2013)	Gastrointestinal<br>Breastfeeding: *They experienced estimated breastfeeding as a more difficult method because they felt insecure about the infants' intake of breast milk*<br>Parental Support<br>Parental involvement in care: *"The mothers are strengthened in their maternal role and dare to trust their own abil-ity. The mothers can read the infants signals at an earlier stage and in a better way."*<br>Healthcare workers-Knowledge and Competence<br>Staff insecurity: *..Some staff experienced a loss of control after estimated breastfeeding was implemented as they did not know the exact intake of breast milk after a breastfeeding session... ...Some staff did not trust what they saw and what the mother was telling them. The staff considered that sometimes the reduction was too big and the infant did not get sufficient milk and lost weight. Other times, the reduction was too small and the infant got too much milk and vomited. ...Some staff experienced the decision making in estimated breastfeeding as difficult as they had to predict and evaluate decisions made from a more complex viewpoint. ...They experienced estimated breastfeeding as a more difficult method because they felt insecure about the infants' intake of breast milk and struggled to use amore person-centered care approach where the mothers' and infants' states and needs were acknowledged.'*<br>Relationships with others<br>Bonding with parents: *'Staff experienced estimated breastfeeding as a more supportive method for the development of the mother/infant relationship because of the uninterrupted closeness.' 'The staff stated that the mothers stopped comparing how well or how poorly other mothers' infants fed after estimated breastfeeding was implemented'.*<br>Suffering<br>Comfort: *"The infant can lie securely in the mother's arms directly after breastfeeding and not on a hard scale."*
Gaíva (2001)	Respiratory<br>Nasal congestion: *"If having nose congestion is something common for every premature baby"*<br>Gastrointestinal<br>Regurgitation: *"The other day I got really scared, the baby regurgitated, and milk came out of her nose, I went crazy"*<br>Frequency of defaecation: *"A handbook with instructions about elimination, like if it's normal for the baby to go two days without defaecation"*

Confidential: For Review Only

	Feeding difficulties: *"From the mothers' reports, it is observed that activities like...breastfeeding, become complex when taking care of a baby."*<br>Healthcare utilisation<br>Frequent readmissions: *"I'm afraid of the baby having to return, I think they could talk more about the premature baby, the risks"*<br>Healthcare workers-Communication<br>Keeping parents informed: *"They could provide a manual about what the baby might have" "Preparation for discharge refers to instructions about care with hygiene, feeding – focused on breastfeeding, administrating medications, and returning to the outpatient clinic for follow-up visits. The mothers reinforce their need to receive instructions at the moment of their child's hospital discharge."*<br>*"In view of the statements, it is observed there is a need for educational material covering the care that should be offered to newborns in risk situations."*
Garcia (2004)	Healthcare workers-Communication<br>Communicating uncertainty: *"It's difficult, you know. I think as medical practitioners it's, I think in medical practice we find it difficult to ... We have to accept that we don't have all the answers in terms of treatment ... and of course I'm sure that comes as a disappointment to the families."* Candour: *"It's about trust, you know. I will trust someone who was honest and said he didn't know (more) than someone who lied."*<br>Healthcare workers-Knowledge and Competence<br>Ethical decision making: *"It's difficult, you know. I think as medical prac-titioners it's, I think in medical practice we find it difficult to ... We have to accept that we don't have all the answers in terms of treatment ... and of course I'm sure that comes as a disappointment to the families."*<br>*"but when you actually mention this, say, "Well, in fact you know, we don't really know what's the best treatment," it is a delicate moment. I'm not really bothered from how they perceive me, as a person, because you know, there's lots of things I don't know in life, but their confidence of how their baby's going to be managed, that's the issue that comes up."*<br>Other<br>Iatrogenic harm: *"... I don't think we know...there are potential toxicities, very real toxicities associated with it, so there is this balance of...benefit and harm..."*
Gontijo (2008)	Healthcare workers-Knowledge and Competence<br>Consistency of decisions: *'In interviews with mothers, it becomes more evident, because they claim that only become effectively adopt the kangaroo position when they are sent to Kangaroo infirmary. Even so, kangaroo position, It is not adopted continuously in the second stage.'*<br>Growth<br>Growth: *evident satisfaction with the method, especially about the best development of your child, highlighting the weight gain which is the main factor*
Green (2015)	Parental Support<br>Coping with maternal illness: *Understandably, the nurses were very concerned about the mental health and well-being of all mothers in the NICU, however, their level of concern was heightened for mothers of extremely premature IVF babies. They emphasised that it was only natural that mothers who had suffered multiple losses, and potentially another loss if the baby did not survive, to experience severe psychological distress. Another loss could be the loss of a 'normal baby'. One nurse spoke of her belief that mothers could be diagnosed with depressive conditions, particularly when they took home a baby that had ongoing health problems or a disability, and the mother*

Confidential: For Review Only

	came to understand the reality of her situation.'<br>Healthcare utilisation<br>Inappropriate treatments; *For the nurses, all babies are precious, and while they recognised that pregnancies conceived through assisted reproductive techniques were precious to their parents, they rejected the idea that these babies were more precious than babies who were conceived naturally. The nurses were concerned that the metaphor of 'precious' used to described IVF babies, could create a view that mode of conception will affect the treatment options offered to parents of extremely premature babies.*<br>Healthcare workers-Communication<br>Communicating in challenging settings; *The nurses believed that when parents requested 'everything' (Nurse 13) to save the life of their baby, they would not know what 'everything' meant.*<br>Healthcare workers-Knowledge and Competence<br>Ethical decision making; *...all of the nurses who were interviewed had seen and worked professionally with many families with a history of infertility, and hence had first-hand experience witnessing the complex dilemmas and realities faced by infertile couples*<br>*The nurses understood the parents' desperation to have a baby and experience parenthood. They believed on the other hand that such desperation should not be the sole determiner of whether the baby survived.*<br>Suffering<br>Suffering: *"It's hard looking after a baby where it's being kept alive just to meet the needs of the parents, because they want a baby."*<br>*Participants knew the baby would experience pain and suffer from intensive care therapies. All the nurses believed that they were affected by the baby's suffering, because it was "agonising for the staff who have to look after them"*<br>Survival<br>Survival: *"It's their one and only chance of having a child. They can only see that immediate sense of having a child. . .they want to take home a baby."*<br>Survival with disability: *"As hard as it is for us to understand, they are happy to have a child of their own regardless of what it is. They're happy to suffer the consequences, so to speak, just to say that it's their own; that it's something that they can love."*<br>*"It isn't up to us to say what is quality of life, because parents might think that even if the child was disabled, that it was better than not having a child at all."*<br>*"They want to take a baby home and often voice that they don't care what the infant is like."*<br>*"To have something that's alive, I can understand that. I can understand somebody saying, 'I want that infant in preference to nothing at all'."*<br>Other<br>Long term effects on parents: *"We should be looking at Postnatal Depression after the baby goes home. . .Once they actually get a baby home, that's when the reality sets in. All the triggers are there."*
Gregory (2012)	Respiratory<br>Oxygen dependence: *Oxygen status*<br>Gastrointestinal<br>Feeding practices : *Participants were asked about the clinical criteria that are used to determine initiation, delay, and advancement of enteral feeding and about the specifics of enteral feeding administration (ie, bolus vs continuous, frequency of feeding, type of feeding, and nutritional fortification added to feeding). Finally, participants were asked to report the volume at which patients were considered to be at "full feeds" and how PN was discontinued.'*<br>Infection

Confidential: For Review Only

	Sepsis: *Identified outcome: Sepsis*<br>Healthcare workers-Knowledge and Competence<br>Consistency of decisions: *Compliance with guidelines Practice patterns of the physician Practice patterns of the nurses*<br>Growth<br>Growth: *Time to return to birth weight*
Griffin (1997)	Parental Support<br>Preparation for NICU admission: *Parents described benefits of the tour, including that it (a) decreased their fears, (b) inspired hope for their newborn's prognosis, (c) provided reassurance about care in the NICU, and (d) prepared them for their newborn's NICU hospitalization. All parents described at least one of these benefits, including five mothers who said the tour was overwhelming or difficult because of the appearance of the newborns.*<br>*'For several mothers, the tour inspired hope for their newborn's prognosis, especially when the mothers saw very premature infants who were said to be progressing well.*<br>*Parents evaluated and provided suggestions on the way the tour was arranged and conducted and offered advice to other parents. In general, all parents recommended that parents in similar circumstances should be offered a prenatal tour of the NICU.*<br>*Parents advised that more health care providers suggest tours to parents diagnosed with a high-risk pregnancy. Two mothers also recommended that other perinatal health care providers should tour the NICU so that they can be supportive to parents. One mother perceived that her need to tour the NICU was not supported by the staff on the antepartum unit.*<br>*Parents who toured with their partners commented that having each other as a support person was helpful. They recommended that the tour be scheduled so that the partner or other support person could accompany the parent.*<br>Healthcare workers-Communication<br>Communicating information effectively: *Parents re-ported that it was important to receive detailed information on the following: (a) newborns who had a di-agnosis or gestational age similar to what was antici-pated for their newborn; (b)a description of equipment for the newborn; (c) roles of staff members; and (d) a description of the parental role in the NICU, including the visitation polic It was important to parents to hear about the parental role....However, all parents did not perceive that they received adequate information on the parental role.*<br>*The need for more specific information became apparent to parents after their infants were cared for in the NICU. These parents indicated that they wanted more information on expectations for their role in the NICU, breastfeeding, sibling visitation, and the potential for the newborn to be transferred from the NICU to another unit before discharge. Two parents suggested that handouts could supplement or reinforce information that was given during the tour and assist parents to inform family and friends about the NICU.*<br>*Parents reported that the tour should be individualized to meet the specific needs of parents. Parents perceived the tour as individualized when they went as a couple or an individual rather than in a group, had an opportunity to ask questions, and saw newborns who had a diagnosis or gestational age similar to that expected for their newborn. Therefore, it was critical for the nurse conducting the tour to know the parents' maternal-fetal diagnosis. Several parents made additional suggestions, such as having an opportunity to go on a second tour or changing the order in which the NICU patient care areas are shown; these demonstrate the parents' individual needs.*<br>Healthcare workers-Knowledge and Competence<br>Healthcare professionals behaviour: *"Knowing they do care about them and they do realize they are human and not machines . . .you could feel that they really cared and worried"*<br>*Parents reported that the tour was comforting and reassuring because it gave them an opportunity to observe the type and quality of care that the infants received.*

Confidential: For Review Only

	Hearing specific information about primary nursing also helped some mothers to feel more comfortable. These mothers explained that it was reassuring to know that their questions could be answered because the primary nurse would know their infant.<br>*Most parents reported that the nurses who conducted the tours were knowledgeable and comforting. These nurses were described as compassionate, concerned, helpful, and considerate of the time parents needed to understand the information and ask questions.*<br>Staffing levels: *It was especially helpful for the parents to see so many nurses and physicians in the NICU.*<br>Relationships with others<br>Effects on family and friends: *The tour gave mothers information about the NICU they needed to share with other family members. One mother indicated that she had gained an understanding of the unit and was better prepared to talk to her child about the NICU. Three of the four mothers who were not accompanied on the tour by the fathers reported that they shared information about the NICU with the fathers, which was comforting to them. One of these mothers described her husband's reaction to their infant's admission to the NICU, "My husband was calm because I had already told him what to expect."*<br>Normality<br>Thriving: *"The tour gave me hope that he was going to be fine. Seeing babies younger than him thrive. . . and then seeing the babies approximately his age survive, thriving, and doing well."*<br>Other<br>Physical appearance: *"it's still frightening to see babies that small."*
Hanrahan<br><br>(2007)	Gastrointestinal<br>Choice of milk for feeding: *"it's like they [scn providers] didn't inform us when they were trying to feed my daughter [formula]"*<br>Parental Support<br>Care provided close to home: *"and nurses' opinions would come into it, too. if they're aware…that parents aren't showing up, including in the evenings…then that may weigh into the decision-making process…make us want to get the baby close to home because we know the parents can't get here."*<br>*"[i was glad our infant was transferred to the com-munity hospital] because of lack of transportation.Because then i [was] able to see him more."*<br>Healthcare workers-Communication<br>Communicating information effectively: *"i don't think that [it] is communicated as well that your baby's well enough to go back to a unit that isn't as intensive as the one that it's coming from."*<br>*"they were originally using all these technical terms [about back transport], and he [the infant's father] was like "What is that? slow down, we still have no idea what it is.""*<br>Keeping parents informed: *"i mean, i found at [the nicu] they explain more to you what was happening with my daughter, what they were doing, how well she was doing. You know, at [the scn] you would go there and they're just doing something. they're not—they don't have time. You know you have to approach them…and say, okay, what's going on now? how well is [my infant] doing today? You know, stuff like that. i want them [scn providers] to, you know, talk to me, let me know, don't sugar coat what's, you know, going on."*<br>*"it's like they [scn providers] didn't inform us when they were trying to feed my daughter [formula] and she was on breast milk, so she threw it all up.…they didn't tell us until we got up there."*<br>*"i asked so many questions and read all the charts every day, and i probably angered them. squeaky wheel gets the oil, as they say."*<br>Treating parents with respect *"i got yelled at by a nurse at [the scn] for rubbing my son's foot [even though that was okay at the nicu]. and rubbing his hand while he had a hold of my finger. i was just rubbing his little palm, and then she told me not to do it because it would stimulate him." "i would've liked them to greet me as a parent—you know, acknowledge me."*

Confidential: For Review Only

	"[My experience at the scn] could have been better; they could have made me feel comfortable there."<br>Healthcare workers-Knowledge and Competence<br>Staffing levels: *"[at the scn] you're not gonna have somebody there all the time, right next to your baby."*<br>*"one time we went [to the scn] and it was like eight-thirty at night. and there was only like three or four nurses in there, and it was a full room. and there were babies in every bed. and they were having a lot of problems with this one little boy, and they left all the other babies and went to him. so i ended up staying there real late because i wasn't gonna leave my daughter by herself and have something happen to her while they're just with this one child. You know, i think staffing is a really big issue [at the scn]."*<br>Identifying who is responsible for care: *"sometimes we're not real clear who to follow up with."*
Hefferman<br><br>(1999)	Gastrointestinal<br>Other gastrointestinal malformations: *"One mother, left with one surviving twin who has an ileostomy, told me that she is glad the sibling died and does not have to endure what the living twin is enduring."*<br>Surgical<br>Requirement for ileostomy: *"One mother, left with one surviving twin who has an ileostomy, told me that she is glad the sibling died and does not have to endure what the living twin is enduring."*<br>Healthcare utilisation<br>Need for lifelong care: *"MDs are making such choices for parents and when the outcome is disastrous they just expect parents to take home severely handicapped babies and deal with life-long problems."*<br>Suffering<br>Suffering: *"Three weeks of high pressure only proved to prolong her death rather than improve viability...Being one of the nurses at that bedside every day was frustrating and often agonizing. I definitely feel that even with all our technical expertise, we failed to give good and sensitive care to both this particular infant and her parents."*<br>*"The problem [with] our high tech machines is not only do they prolong life, they often prolong death. I am not saying we should never attempt to salvage VLBW babies. We have some miraculous success stories. But we should have some guidelines to follow and the decency to stop before we begin to do harm."*<br>*"It often seems that when we resuscitate these edge of viability infants it is to please the parents, especially in the IVF environment that we have today. The concern is not for the comfort of the infant; the concern is to give the parents their child no matter what."*<br>*"One mother, left with one surviving twin who has an ileostomy, told me that she is glad the sibling died and does not have to endure what the living twin is enduring."*<br>*"We poke, prod, and torture them for weeks, give a lot of false hope to the parents, stress the staff, and then they are taken off and allowed to die . . . for us it is ethically the worst part of our job."*<br>Normality<br>Normality: *"It is hard to care for very small sick infants whose potential as a normal child and adult is poor."*
Hentschel<br><br>(2006)	Healthcare workers-Communication<br>Awareness of parental views: *The data show that in 9 cases the clinical team had no knowledge of the parents' wishes.*<br>Pain<br>Pain *: Table 3 -summary of medical staff's perceptions of parents' wishes concerning RIC* [Restriction of ongoing Intensive Care] *Agree to RIC: "Avoidance of pain/suffering"*<br>Suffering

Confidential: For Review Only

	Suffering: *Wishes of parents as percieved by medical staff "Avoidance of pain/suffering" "To let the infant die in peace"*<br>Normality<br>Normality: *Wishes of parents: "A healthy child" "To take the child home"*<br>Survival<br>Survival: *-summary of medical staff's perceptions of parents' wishes concerning RIC: "Survival of the infant" "Maximum therapy" "Therapy even in case of low chance"*
Holditch-Davis<br><br>(2003)	Gastrointestinal<br>Nutritional intake: *"Ever since he's been home, we've kept records of how much he was eating, on a daily basis."*<br>Neurological<br>Seizures: *"I thought I'll time it and see how long these things last-which was anywhere from 5 to 7 minutes. And his color would change, and the way he did his eyes. They rolled. And his eyes would water, and he would tense up. I explained this to the doctor. And he was the one that said it could possibly be seizures. So we're going to take him in and have him tested. But this is something that we were told could be a possibility, and I don't see it as anything major."*<br>Infection<br>Susceptibility to infection: *"particular about her germ thing. I don't want her chewing on every-body else's toys or what have you."*<br>*"The biggest worry right now is when he is going to get sick. I'm sure he's able to handle it, but at the same time, we're overly protective and don't let people in his face"*<br>Healthcare utilisation<br>Recurrent sickness: *"We've only put him with other children for the past month. The biggest worry right now is when he is going to get sick. I'm sure he's able to handle it, but at the same time, we're overly protective and don't let people in his face."*<br>Relationships with others<br>Childhood happiness: *"But to me I look at her and I don't see it all. I see this little happy little thing!"*<br>Normality<br>Normality: *"And how helpless he was and that I couldn't hold him. That was really the hardest thing."*<br>*"But I don't look at her as a premature....But to me I look at her and I don't see it all. I see this little happy little thing!"*<br>*"Is he going to be his normal size? . . . If he was supposed to be small then that's fine. He can be small. But I guess I'll never know."*<br>Survival<br>Survival *"It hurts. I didn't know, I didn't know if they were going to make it or not."*<br>*"I have a fear of them dying. I'm still scared because they were premature that that could happen to us. And grant-ed that could happen even if they weren't premature!"*<br>Growth<br>Growth *"The most difficult thing for me is the weight thing. Is he big enough? Is he not big enough? Should he have been bigger? Would he have been bigger? How big would he have been when he would, if he was born when he was supposed to be born?"*<br>*"just look at her as a little baby. . . . The doctor says she's 3 lb under weight. So that's like 20% bodyweight."*
Hussey-<br><br>Gardner (1998)	Healthcare workers-Communication<br>Developing a caring relationship: *"I couldn't get over how gentle she was. It seemed like she was truly concerned"*<br>Normality<br>Normality: *"Being reassured that he was on line for how old he was...Just reasurance he was doing well"*

Confidential: For Review Only

	Growth<br>Growth: *"She was born so early, it's nice to see that she's finally catching up with how she's growing"*<br>*"I asked more questions about where he was supposed to be..."*
Jirapaet (2006)	Respiratory<br>Mechanical ventilation: *"I couldn't find time to change the adhesive tape, that was wet by saliva, of the endotracheal tube of another baby … it caused tube dislodgment …"*<br>*"I was filling [free flow of] water into the humidifier chamber and was distracted by a call to assist a physician in an emergency case. I for-got to close the line clamp … water flowed into the baby's lungs unnoticed."*<br>Skin<br>Extravasation injuries: *"The i.v. site was covered with cloth.... leaked for a long time, skin area was inflamed and edematous like popeye's arm."*<br>*"I was unsure if the baby had an IV leakage and asked a senior nurse to confirm it. But she was busy …. I saw the baby again when there was tremendous edema."*<br>Burns; *"wasn't lazy in changing the pulse-oximeter sensor site [recommended to relocate q3-4 h] … But I didn't believe that it could really burn the skin until I had experienced it."*<br>Pressure sores: *"I had to skip changing a [hydrocephalus] baby's posi-tion for that shift … Her head developed pressure sores."*<br>Healthcare workers-Communication<br>Communicating information effectively: *"Doctor wrote a new order on the patient chart and left it on the desk, but didn't say a word …. The order should have been taken but no one noticed it."*<br>Healthcare workers-Knowledge and Competence<br>Medical errors: *"I had to draw an antibiotic from a vial that was diluted by another … without knowing that it con-tained the wrong preparation …"*<br>*"Doctor wrote 3 like 5."*<br>*"I set the infusion rate at 12 mL/min instead of 1.2 mL/min. I couldn't see the faint decimal point."*<br>Other<br>Iatrogenic harm: *"I performed a venepuncture without using a radiant warmer properly … baby developed hypothermia."*
Johnson (2007)	Parental Support<br>Parental involvement: *Being involved in caring for the infant was described as being needed by every mother in the study.*<br>Relationships with others<br>Bonding with parents: *"made me realize that I know my little girl"*<br>*Many of the mothers spoke how holding helped them "understand" their baby's needs better Mothers related feelings of "being needed."*<br>Suffering<br>Comfort: *"I will make you feel so much better in my arms,"*<br>Normality<br>Normality: *"mommy will sing to you our favorite song for as long as you want"*<br>*"loud noises the rest of the time, we can escape to be alone for an hour."*<br>*as one mother stated, "you can't learn how to be a mother from a book. You have to learn with your baby."*

Confidential: For Review Only

Jones (2015)	Gastrointestinal<br>Breastfeeding: *"it was her first time and just explaining to her how, the right way to breastfeed and attachment and things like that (Nurse 9)"*<br>*"We went through from start to finish any questions she had. We ran through a breastfeed, we had all the props out, and just with the use of the props and things it was a bit easier …It actually worked out really, really well. After that, we got the baby to breastfeed beautifully and it was nice"*<br>Parental support<br>Culture differences: *But I think sometimes some of the Aboriginal Community can also be hard to talk to. I think that's just their environment that they've been bought up in*<br>Support from healthcare workers: *They'd say "Well at the end of the day go with your feelings of what, if you feel she's feeding really well when you do a certain thing, then keep doing it and if you do something else it and doesn't work, then stop doing it,"… "You will know best because you just know how it works with your new baby"*<br>*'So it's just about giving them lots of options and supporting them to make the choice that's right for them'*<br>Support from family and friends: *For example, one woman requested that her friend be able to come into the nursery and hold her baby. The woman had no family members in Australia, and her friend was the only person providing support. The nurse caring for her baby refused her request on the grounds that the unit had a 'policy' that only parents and grandparents could hold the baby. This created distress and conflict.*<br>Healthcare workers-Communication<br>Communicating information effectively: *One of the most common and important issues for both parents and nurses was the concept of sharing 'information'. Parents 'wanted' nurses to 'provide' information and nurses 'wanted to provide' parents with information. Interestingly, fathers had a tendency to mention information first compared to mothers. From the parents' perspective they wanted to know 'everything' from what they considered to be 'general information' about for example what 'creams' they should use on their baby to how equipment worked and, of course, the condition and progress of their baby.*<br>*I feel like I'm receiving all the information that I need, so I'm really extremely happy about that even though sometimes we're not asking questions, they will give us additional information*<br>Communicating in challenging settings:*I think your very young mothers, very hard to talk to because they're babies and they don't understand, they see us more as a authority figure as in someone that could be their friend, and be helpful ……*<br>Developing a caring relationship: *Nurses who 'listened' and were 'empathic' were highly valued. Parents also used words such as 'friendly' 'approachable' and 'caring' to describe the communication style of supportive nurses; I just felt she had a way with words and made me feel really safe (Regional Mother 4).*<br>*I have been nursing for a while now, the most important thing you would do with any patient; whether it a baby, whether it an adult, you build up a rapport*<br>Keeping parents informed: *She rang the previous room, and they told her, "Well, your baby's not here anymore," so they put her through to my room, I got the phone call, to me and she was like, well, just a bit cranky at the fact her baby had been moved without any prior knowledge to it, and I coped a brunt of it (Nurse 13).*<br>Treating parents with respect: *Yeah, I think the nurse was worried about the whole touching and whatnot … Like she's very full on with protocol but she was very abrupt and didn't explain things. So she upset mum and this upset me a bit too (Metropolitan father 26).*<br>Healthcare workers-Knowledge and Competence<br>Consistency of decisions:*'Everybody had a different point of view but they were opinions, not facts. So that was huge, don't even get me*

Confidential: For Review Only

	started on that, that was just a nightmare. (Regional mother 6)'<br>Healthcare professionals behaviour: "*I asked "Can you come?" 'Cause they had to check that it's down the right tube, …. And she didn't come and do it and I had to go and get her and say, "Can you help me?" 'Cause I'm not allowed to do it by myself. And then she was talking and it took her 5 minutes to finish her conversation with the other nurse, and it wasn't a nurse related conversation it was just a casual conversation … Like I felt a bit (sic) she wasn't their priority."*<br>Normality<br>Normality: *A major focus for parents was seeking information that told them that what was happening was 'normal' and that everything was going to be 'fine'.*
Kavanaugh<br><br>(1997)	Parental Support<br>Support from family and friends: *Certain behaviors of family, friends, and health care providers were perceived by parents as supportive. Some behaviors that were described as helpful by the parents were not unique to wny specific relationship, whereas others were confined to relationships with health care providers. Three behaviors that were not specific to any particular relationship were accepting the parents' feelings and behaviors, being there, and sharing the experience.*<br>*Parents reported that they did not attend a support group because they could not share their experience in a large group or because their family provided adequate support.*<br>Support from healthcare professionals: *The other behaviors described only with respect to health care providers were giving information, providing competent care, and giving special attention.*<br>*To the parents, being there meant being available. Nurses were available to the parents by being physically present or by being close by if the parent needed the nurse, especially if the mother was alone during labor....Parents used the term "share" when they described individuals who genuinely understood their experience.*<br>*Only one mother recalled sharing the loss with someone who was not a bereaved parent. This mother described her experience with a nurse during the delivery.*<br>*Parents perceived that health care providers gave them special attention by breaking hospital rules, giving additional time, and making the parent a priority among other patients. Breaking hospital rules included expanding visitation to allow for unrestricted visits from family and friends.*<br>*Most parents could not identify the reason for inadequate pain relief. One mother stated that she was denied an epidural by an anesthesiologist, who told her that "women have been doing this for thousands of years without an epidural.*<br>Healthcare workers-Communication<br>Communicating in challenging settings: *"And I don't want some fluke off the wall, you know. You don't have to tell me. But what I'm saying is the things that have a reasonable chance of happening" the parents reported that when delivery was imminent, the obstetricians explained that the newborn would have had a better chance of survival had the pregnancy been maintained for an additional few weeks. In the other instance, the mother reported that the obstetrician told her about the likelihood of her twins' survival when she was treated for preterm labor.*<br>*Only one couple perceived that they were given an opportunity to discuss how much aggressive care they wanted for their twins. This couple was the only one to report that they felt prepared for their twins' deaths even before they were born.*<br>*Certain decisions, such as choosing the type of burial, were especially difficult because of the parents' emotional state or lack of experience. Most parents had no experience making funeral arrangements. All parents reported that it was helpful to have information, guidance, and additional time before making final decisions.*<br>Keeping parents informed: *All parents reported that they wanted health care providers to keep them informed of the mother's and*

Confidential: For Review Only

	newborn's condition and treatment plans. When health care workers' provided honest, straightforward information in understandable language, parents gained a sense of control over the situation and their fears decreased.<br>Healthcare workers-Knowledge and Competence<br>Health professional competence: *Most of the parents recalled specific incidents that they perceived as poor medical care; typically, these incidents involved technical procedures or medical knowledge...Parents expected expert knowledge, pain relief, and consistency in care: "Team" was the word used to describe staff who were organized and had communicated well among themselves, especially when transfer of care occurred between shifts or units.*<br>Relationships with others<br>Effects on family and friends: *Family and friends were perceived as available to parents when they visited or called. Parents stressed the importance of others' acknowledging their loss and being willing and unafraid to talk to them. Comments that parents perceived as inappropriate were those that minimized the loss, provided unsolicited advice, or criticized the parents on their health care.*<br>*Breaking hospital rules included expanding visitation to allow for unrestricted visits from family and friends.*
Koh (2007)	Healthcare workers-Communication<br>Using aids to communication: *Most of the mothers in both groups were positive about having their conversations with the neonatologist taped.*<br>*six of the mothers in the control group could not recall their conversations with the neonatologist*
Leavy (2015)	Skin<br>Appearance of scars: *In addition, hospitalization and different interventions in their first days of life have left marks on their bodies. Such marks may become a stigma if their peers give them a negative meaning, leading to prejudice and discrimination*<br>*"I do not like the scar on my belly [...]; I was at the beach and everyone kept staring at me like `That is a big scar!´"*<br>*Being afraid of becoming the laughingstock may make them feel insecure, thus affecting the construction of their own identity: "I think about these things (scars) when it comes to girls; it makes things more difficult because girls think you have washboard abs but your stomach is actually all flabby because of a poorly healed scar resulting from a surgery" (FG 6), said one participant in relation to a keloid resulting from a surgery. However, some take ownership of these marks and give them a new, positive meaning: "When I was young, I felt embarrassed when I had to take off my t-shirt, I thought they would all stare at my scar, and I developed a body image complex. Now I have tackled this issue and it has become part of my story, thanks to this scar I am alive" (FG 9). In this case, the scar becomes the footprint of a surgery that allowed the participant to survive, and this is more significant than the stigmatizing vision of others.*<br>Surgical<br>Appearance of scars: *"I do not like the scar on my belly [...]; I was at the beach and everyone kept staring at me like `That is a big scar!´"*<br>*"I think about these things (scars) when it comes to girls; it makes things more difficult because girls think you have washboard abs but your stomach is actually all flabby because of a poorly healed scar resulting from a surgery"*<br>*"When I was young, I felt embarrassed when I had to take off my t-shirt, I thought they would all stare at my scar, and I developed a body image complex. Now I have tackled this issue and it has become part of my story, thanks to this scar I am alive"*<br>Development-Gross motor<br>Ability to walk: *"I received lots of stimulation; otherwise, I would have ended up in a wheelchair."*<br>Development-Speech and Social<br>Language disorders: *"I also had a hard time learning to talk."*<br>Healthcare utilisation

Confidential: For Review Only

	Frequent appointments: *"I felt left out, I was always missing school because I had to go to the hospital for check-ups"*
	Mixed feelings are observed in those participants who have special health care needs at the hospital, from familiarity and thankfulness to rejection and discomfort: "It is a complicated subject because I am alive thanks to the hospital, but I am tired...Now I just go for scheduled check-ups, but when I am there, I just want to leave"
	Healthcare workers-Communication
	Communication with ex-neonatal patients: *Some participants indicated having had a negative experience with health teams: "They said that I was not going to walk and that I was going to be blind, deaf and mute. They would give me no hope and say: 'This baby will not make it to the sixth day…´" (FG 8). "My father was told that when birth is premature, either the baby or the mother dies... but here we are, both of us are alive" (FG 5). Likewise, they felt uncertain about what aspects of their present health status were actually related to their history of premature birth.*
	The hospital and the relationship with the medical team play a significant role, which is sometimes contradictory. On the one hand, many participants stated that they were thankful and appreciated the bonds established in the hospital setting. On the other, they often mentioned how medical diagnoses were uncertain or pessimistic regarding their survival and potential sequelae.
	Psychiatric
	Need for educational support: *Overlapping of special health care needs and school hours resulted, in some cases, in a poor school performance: "I felt left out, I was always missing school because I had to go to the hospital for check-ups" (FG 10). Repeating a grade was frequently associated with learning difficulties and an achievement gap in relation to classmates. Several interviewees had to attend a remedial education program. Most of them felt comfortable in such setting because it helped them overcome their learning difficulties and favored their socialization: "I attend a special education school because when I was in a bilingual school I could not advance to the next grade, the other kids mocked me or left me aside, they did not understand my problem. I am doing fine now" (FG 7).*
	Relationships with others
	Overprotective parent-child relationship: *They perceive their parents consider them to be more vulnerable and dependent on their custody than the rest of their children or their peers. This may lead to inter- and intragenerational conflicts: "I have always been treated differently than my sisters. Yes, they tend to be overprotective, that bothers me" (FG 2)... This is a traumatic experience for parents, and it is projected into their present relationship with their children...When overprotection is based on giving excessive care to preterm children, the relationship between parents and children becomes distorted. In this regard, a quadriplegic adolescent stated: "All moms left, but my mother would stay all morning at school with me [...]; I started caring for myself and I had to push her away a bit so that I could lead my own life" (FG 5).'*
	Suffering
	Ex-patients separation from their suffering: *For ex-preterm subjects, prematurity is more about their parents' experience than about themselves: "When I tell my story, I use the third person; my mom went through this, not me. She considers my story her own" (FG 1). "We are based on what we were told, not on what we experienced" (FG 8). Participants also emphasize their parents' suffering and uncertainty: "Parents suffer much more than us; they had to suffer waiting for us to get better and see us in that status" (FG 1).'*
	Normality
	Being treated normally; *"I have always been treated differently than my sisters. Yes, they tend to be overprotective, that bothers me" "I wanted to have a piercing but my mother would not let me because they gave me lots of pricks when I was hospitalized and I had suffered, but I do not remember it"*
	Survival
	Survival: *"I am proud of having been born preterm. I almost passed away, and so did my mom. We both made it and it was a miracle"*

Confidential: For Review Only

	Beyond life experiences, even though most participants recognize medical efforts, they attribute their survival to a miraculous and extraordinary event.<br>*"Now I have tackled this issue and it has become part of my story, thanks to this scar I am alive"* Schooling: *"I attend a special education school because when I was in a bilingual school I could not advance to the next grade, the other kids mocked me or left me aside, they did not understand my problem. I am doing fine now"*<br>Other<br>Self-identifying as premature: *'The meaning that subjects assign to having been born preterm varies according to their health status, sequelae and life experience. Those who had sequelae and special health care needs 18 in their childhood associate their preterm birth to a life of efforts and difficulties*
Lee (2005)	Parental Support<br>Culture differences: *3 families felt strongly that their stress derived from differences in the medical management approaches between the United States and their homeland.*<br>Parental involvement: *almost all of the parents expressed a desire to become more involved with the care of their infant*<br>Healthcare workers-Communication<br>Language barrier: *the stress derived from communication with HCPs was mostly related to language barriers for the new immigrant parents.*<br>Keeping parents informed: *Other parents experienced stress from...not being thoroughly informed about their infant's condition or treatment plan.*<br>Communicating information effectively: *Other parents experienced stress from unknown medical terminology*<br>Healthcare workers-Knowledge and Competence<br>Healthcare professionals behaviour: *HCP* [Healthcare professional] *behaviour as stressor for parents*<br>Identifying who is responsible for care: *2 families expressed frustration related to frequently changing bed spaces or hospital units and difficulty accessing doctors.'*<br>Relationships with others<br>Effects on family and friends: *Worry about the possibility of upsetting their own parents. Three families did not tell their own parents about their infant's problem because they were worried that it would upset the grandparents.*<br>Family resources: *3 families felt overwhelmed by a lack of resources (especially in the area of family support)*<br>Other<br>Parental perception of uncertainty: *parent's perception of infant's severity of illness -parent's perception of infant's illness and future impact (i.e. non-specific developmental issues)*<br>Physical appearance: *Both mothers and fathers found their infant's appearance and behavior to be the stressors with the most impact*
Lou (2009)	Respiratory<br>Mechanical ventilation: *"She had been put on a respirator. It was not so bad as I had imagined. I could see that she calmed down. She did not struggle so much and…But the doctors gave me a shock when I came to see her. They had taken an X-ray, and they could see that her lungs were white and stiff and I was told that she was not far from dying"*<br>Frequent respiratory illnesses: *"There were lots of masks and nebulisers during those years"*<br>Gastrointestinal<br>Oral feeding: *"It is small things.. I remember the very first time he ate porridge"*

Confidential: For Review Only

Confidential: For Review Only

	Neurological<br>Sleep disorders: *Subsequent to an account of the son's disturbed sleep at night for several months after discharge, which was an enormous challenge to the couple, a mother described the boy as follows: "He was such a giving child, full of joy!" … adding in a low voice… "Most of the time".*<br>Skin<br>Appearance of scars: *"The only reminder today is an ugly scar"*<br>Development-Fine motor<br>Ability to feed themselves: *"It is small things.. I remember the very first time they ate porridge. In just stod there in the kitchen and cried my heart out...it was the fact that I was able to experience my children eating with a spoon. It was great!"*<br>Development-Special senses<br>Visual impairment: *At the time of the interviews, the only major sequel was in one child with seriously impaired vision.*<br>Healthcare utilisation<br>Need for frequent treatments: *"There were lots of masks and nebulisers during those years"*<br>Psychiatric<br>Dyslexia: *"We were told that when he started school he might have problems, dyslexia or learning dis-abilities."*<br>Need for educational support:*"We were told that when he started school he might have problems, dyslexia or learning disabilities."*<br>Relationships with others<br>Bonding with parents: *"He might also… When I was working in the kitchen, he might come running at full speed from the family room, hug me.. around my legs.. he was no bigger than that.. and say: "Oh, you are the very best mum".. and off he went again.. He just wanted me to know!"*<br>Normality<br>Normality: *Finally, a mother called it a developmental land-mark when an older sister dared show her irritation towards her little brother, "no longer treating him as if he were made of glass"*<br>*"They get up and try again until they succeed. That is the same way he keeps on trying."*<br>Survival<br>Survival: *"They had taken an X-ray, and they could see that her lungs were white and stiff and I was told that she was not far from dying."*<br>*"Still, I consider the most important moment in my life.. the very second I pushed the pram over the doorstep of the neonatal unit. I remember it very clearly. I had been looking forward to that for two months."*<br>Survival without disability: *"And afterwards you are worried about how they are going to survive. If they would have impairments, and so on."*
Malhotra (2015)	Respiratory<br>Mechanical ventilation: *Significant factors that make professionals categorise a baby as 'sick' High ventilator settings*<br>Oxygen dependence: *Significant factors that make professionals categorise a baby as 'sick'… Oxygen of over 30%*<br>Cardiovascular<br>Hypotension: *Significant factors that make professionals categorise a baby as 'sick' "hypotension"*<br>Presence of Patent Ductus Arteriosus: *Significant factors that make professionals categorise a baby as 'sick'…PDA*<br>Gastrointestinal<br>Necrotizing enterocolitis: *"If we are trying to stimulate the gut before it is ready making it more prone to having complications of either*

	feeding intolerance or, more severely, necrotizing enterocolitis."<br>Initiating enteral feeds: *"MEF* [minimal enteral feeds] *should be initiated in first 2-3 days of life as long as the baby is stable."*<br>Healthcare workers-Knowledge and Competence<br>Consistency of decisions: *"it is difficult to have faith in the decisions when they don't even agree with each other"*
Miljeteig (2006)	Parental Support<br>Maintaining hope: *The informants described how parents thought there was hope so long as the child was in hospital, and the doctor's dilemma consisted in both keeping the parents' hope alive and in giving them a realistic picture of the situation and of probable outcomes.*<br>Healthcare workers-Communication<br>Communicating with parents: *Most of the informants reported dilemmas in relation to parents. The most frequently mentioned problem was communication with parents of poor education and low socio-economic status. Many said that parents did not understand medical terms, and were thus unable to appreciate the seriousness of their child's condition... "How do you explain 'brain-dead' to a person who does not understand what a 'brain' is?"*<br>Healthcare workers-Knowledge and Competence<br>Ethical decision making: *All of them underlined that it was, and had to be, the most senior doctor or registrar who took ethically difficult decisions. Many found that they had no influence on this matter, but all except two had in some way been involved in decisions concerning withdrawal. Some of them focused primarily on how to keep the baby alive, while others were more concerned about when to apply or withdraw treatment. In the latter cases, emphasis was laid on the available medical alternatives, on data concerning survival and treatment results for similar children at their unit, the resources available, the law against euthanasia, and the parents' situation....All the informants, except one, claimed not to be influenced by their religious beliefs. Several referred to God as the ultimate decision maker, and regarded the relevant decisions as a practical rather than an ethical challenge... Many also said that it was easier for them to handle these situations than for their Western colleagues, because they were used to seeing children die.*<br>*Whether or not to involve the parents, or to seek their co-operation in decision making, was a frequently mentioned dilemma, although not all informants were convinced that parents ought to be involved in decisions concerning withdrawal of treatment. One problem experienced was that some parents perceived the doctors as gods. These parents tended to agree to almost everything that was recommend to them and refused to participate in decision making. Many of the doctors said they felt uncomfortable in such situations. They did not want to be responsible for life and death decisions. Three doctors explained how they left the final decision to the parents, so as to avoid the sense of guilt associated with deciding on someone else's life.*<br>*Almost all the doctors at both hospitals mentioned the gender problem in India as a dilemma in their work. Several had experienced the conflict themselves. They had noted, for example, that parents found it easier to consent to the withdrawal of treatment, or that there was less interest in buying medicine, when the baby was a girl... Lack of understanding, lack of resources to take care of a daughter or other children, fear of high dowries, and the cultural stigma of having a girl were some of the factors mentioned.*<br>*Some reported that when they knew the baby would die anyway, they did not consider the withdrawal of treatment as an option...Babies in critical conditions provide learning opportunities, which doctors feel they can make use of so as to perform better next time or when the economy improves. This perspective was reported both by senior registrars and by postgraduate students.*<br>Relationships with others<br>Effects on family and friends: *In many cases of terminated treatment, the doctor had felt uneasy about continuing treatment for babies with uncertain outcomes, when they knew that the parents could not afford to provide lifelong care. Responsibility for a family's future was experienced as the worst dilemma when deciding whether or not to withdraw treatment. Where the doctors managed to save a*

Confidential: For Review Only

	premature or critically sick child, they were aware that its survival was likely to create problems for the parents, requiring money for medicine, hospital visits and care. They also considered the risk of incurring lifelong expenses for the parents if the child failed to marry because of a handicap. This would also affect other children in the family...<br>*Those doctors who were concerned about the future of children who survive talked about lack of opportunities with respect to the family rather than the child. Several doctors had experienced mothers at follow-up consultations complaining about the fact that the doctor had saved the child, as there was so much trouble with it. One doctor said that in such situations he could only comfort the mother, but he and others who reported similar experiences felt responsible, even though they had only been doing their jobs.*<br>*"Who am I, a third person, to decide? His brother and sister might not go to school because of this."*<br>*Some underlined that these decisions are different in India compared to Western countries, where guidelines are based on the assumption that the parents or society will be able to cope with the longterm consequences. Many of them found that their own guidelines did not take their local social and economic realities sufficiently into account.*<br>Survival<br>Survival without disability; *"India needs productive people; we are creating the disabled."*<br>*Others said they were aware of the burdens that might result for society by treating high-risk babies. None had withdrawn treatment on these grounds, yet it was a perspective that concerned them when deciding to continue treatment.*<br>Other<br>Healthcare resources: *Limited resources were a common problem for our informants. Part of the dilemma was not being able to offer the best treatment to all, regardless of how many patients there were at the unit. Another aspect was the frustration of not being able to follow the national guidelines or the recommendations in Western textbooks and journal articles. Some had had the experience of having to withdraw, or of helping to withdraw, ventilation from a child in order to give the ventilator to another child with a better prognosis. In these cases it was known that the first child would most probably die anyway, or would only survive with a severe handicap even if given the best treatment available.*<br>*No one mentioned over-treatment as a dilemma in Indian NICUs, although many had a strong sense of wasting resources when critically ill babies were given costly, and highly intensive, care. They experienced this situation as frustrating, and some pointed out that 50% of the unit's resources were used on the 1–2% who were most sick, and whom they hardly managed to save anyway.*<br>*Limited resources for treatment were also a consequence of the poor economy of parents. The doctors reported how they had to balance the need for expensive medication and equipment (which the parents must buy for their child) with their perception of the parents' economy. They wanted to spare the parents the humiliation of having to refuse the best treatment or of not being able to act on recommendations to go to a private hospital, options which would probably result in economic ruin or the starvation of other children at home. Many of the doctors described personally trying to help out in such cases, by looking for funding or giving the parents money or medicine. Some also reported persuading the better off parents to buy things other babies could also benefit from. Another dilemma mentioned was that some children were transferred from private hospitals because the parents could no longer pay for treatment there*
Miracle (2004)	Gastrointestinal<br>Breastfeeding: *"They told me how breast milk was good, helped the babies to grow and help fight infections. Maybe because I had a small baby, and I feel that it helps mature his digestive system. And he's so tiny and not supposed to be digesting food this way, so I feel it's probably that much easier on his system-what nature meant for him to eat."*<br>*"I'm glad I'm doing it. I feed good that I'm doing it. I feel good about myself. It makes me feel good about myself that I'm doing it for him."*<br>*"I didn't feel forced at all. They tell you all the facts about it, how great it is for the baby, how much healthier it is, and they let me know how much better it is for the baby than formula. So I'm happy they do that. No, I didn't feel forced. I needed that push in the right direction*

Confidential: For Review Only

	and just to see the outcome and to hear people say, "Wow, that is good,"and "Wow, look how good you did." To see all that and hear all that, it makes you feel good."<br>*"Also, they've been telling me babies cannot digest the formula like the breastmilk."*<br>Infection<br>Prevention of infections: *"The nurse was talking to me about how breast milk fights off infections"*<br>*"She told me of the fact that they were earlier and didn't have the capabilities of fighting off infections, and they needed that from the milk, from me."*<br>*"There is a mother [peer counselor] that brings her baby with her to Milk Club, and she said her baby didn't have any colds or ear infections that 1st year. That story helped me. It's a success story."*<br>*"The neonatologist came in and said that it was to their benefit to grow quicker, they could tolerate it more, their immune system-that's what is best for them. That's all she needed to say, it was decided by sundown."*<br>Healthcare workers-Communication<br>Developing a caring relationship: *"So it makes me feel comfortable, even with the doctors. I think it was Dr. Wallace, she said (while I was pumping), "How's it going?" And it makes me feel good that we talk to each other like family."*<br>Normality<br>Normality: *"And he's so tiny and not supposed to be digesting food this way, so I feel it's probably that much easier on his system-what nature meant for him to eat."*<br>Growth<br>Growth: *"The doctors were explaining to me that my breast milk would help her gain weight."*<br>*"The neonatologist came in and said that it was to their benefit to grow quicker,"*
Moyer (2010)	Healthcare utilisation<br>Inappropriate treatments: *Community providers (including PCPs, home health nurses, pharmacists, and community emergency rooms) may lack the required knowledge and skills to manage complex infants, leading to suboptimal office-based care and perceived overutilisation of the emergency system.*<br>Healthcare workers-Communication<br>Communication about discharge: *Parents/care givers may be inadequately prepared for home care and management of fragile neonates due to a lack of consistent and early communication between parents and NICU staff and lack of coordinated educational and social services support programmes prior to discharge.*<br>Healthcare workers-Knowledge and Competence<br>Lack of consistency: *Healthcare providers (attending physicians, consulting physicians and other healthcare providers) in the NICU tend to act in isolation, which results in lack of a standardised, coordinated and comprehensive plan of care. Community providers (including PCPs, home health nurses, pharmacists, and community emergency rooms) may lack the required knowledge and skills to manage complex infants, leading to suboptimal office-based care and perceived overutilisation of the emergency system.*<br>Healthcare professional competence: *Community providers (including PCPs, home health nurses, pharmacists, and community emergency rooms) may lack the required knowledge and skills to manage complex infants,*
Orfali (2004)	Gastrointestinal<br>Oral feeding: *"I think his quality of life will be poor; he can't even eat. He will be profoundly retarded"*<br>Neurological

Confidential: For Review Only

	Significant IVH: *''Although she has a grade IV bleed, the resident says that she moves and looks around, and he thinks the odds are quite good.''*<br>*"The neurological outcome is never known (when the baby has a bleed). Ethically, if the baby is severely compromised, what do you do?I've talked to people who have seen kids with grade IV bleeds who come out fine. It makes it hard to make the decision"*<br>*"She has an IVH grade IV, but she moves quite well and things might turn up better then expected"*<br>Development-Special senses<br>Hearing impairment: *"This kid is in really bad condition. I told the parents that he will never be able to see, to hear and I will get more data to show them how bad things are. Because in this particular case, there is no doubt!"*<br>Visual impairment: *"This kid is in really bad condition. I told the parents that he will never be able to see, to hear and I will get more data to show them how bad things are. Because in this particular case, there is no doubt!"*<br>Healthcare workers-Knowledge and Competence<br>Ethical decision making: *Medical authority was exercised in limiting available options. As an American pediatrician commented: "I think they [parents] have played a part in dealing with the decision as much as we [doctors] have played a part in fixing them.''*<br>Survival<br>Survival with disability: *"I think his quality of life will be poor; he can't even eat. He will be profoundly retarded"*<br>*"At 2 cm, we know that the outcome is very dismal, that the kid will be severely handicapped"*<br>Survival without disability: *"I have always thought we should limit treatment instead of giving (the parents) a handicapped child. Especially as we know very well what happens on the long run for a couple. Statistically, there are many divorces and things like that"*
Rasmussen<br><br>(2016)	Neurological<br>Brain injury (not further specified):*''Brain injury is laden with a lot more emotions and moral concerns for sure . . . neurologic or neurodevelopmental impairment in the long term is much more associated with a negative outcome compared to compromise of another organ that can be supported with medical therapies in the long term.''*<br>Healthcare workers-Communication<br>Communicating in challenging settings:*"I see prognosis affecting families medical decisions . . . they do their best. I mean, it's not easy but they do their best to factor in their values and their lives. And they can't really speak for this individual who's never spoken to them before. But I think they do their best. And it's hard. It's a hard situation to be in."*<br>Other<br>Overall health state: *"By neurological prognosis I mean that we try to think of the whole life consequence. We talk about, you know, strength and cognitive capacity, but also life and communication and feeing yourself and getting around."*
Reyna (2006)	Gastrointestinal<br>Choking during feeding *"the only concern I have is, I don't want them to choke. I'm fearful of choking."* Feeding difficulties: *The first theme was interpreting infant behaviors (Table 2). This theme included recognizing infant feeding readiness, hunger, and satiation cues. Feeding readiness included mothers' consideration of their infants' motor and behavior state organization before a feeding, particularly the infants' level of alertness. Hunger cues were the behaviors mothers used to describe hunger in their infants. Similarly, satiation cues were cues recognized by mothers to indicate the infants had fed "enough" and was satisfied.*<br>Feeding practices: *The second theme was managing the evolving feeding process (Table 2). This involved the mother's evaluation of her infant's skill at bottle feeding, meeting the demands of the feeding schedule, and controlling her time and resources. This theme included the mother's perception of how successful she was at balancing her infant's frequent feedings with everyday demands such as*

Confidential: For Review Only

	grocery shopping, doing laundry, and caring for other children. In addition, a mother's previous experience with bottle feeding and how she used the support of other family members to manage this process was part of this theme.<br>*Realizing knowledge gaps was the third theme (Table 2). It involved understanding the nutritional needs of their infants, such as when to advance the feeding volume and when to omit a feeding. It included mothers' feelings of how prepared they felt at discharge and the reality of caring for the infant at home. Another a part of this theme was what the mother had learned from her initial experiences and how she managed anxiety about caring for her infant after discharge.*<br>*Before discharge, all infants in this study were on scheduled feedings with a prescribed feeding volume. Routine discharge instructions for the mother included advancing the infant's feeds as tolerated to an "ad libitum" schedule. Mothers had difficulty understanding these instructions and seemed hesitant to liberalize their infant's intake after discharge. They worried both about giving too much formula at a feeding or about missing a feeding...Despite concerns regarding feeding volume, 26% (8) of the infants were noted to have "spitting through their nose." This typically occurred as the feeding volume was advanced. None of the mothers recognized this as a problem or associated it with overfeeding or difficulty with suck-swallow-breathe coordination.'*<br>Parental Support<br>Parental competence: *The second theme was managing the evolving feeding process (Table 2). This involved the mother's evaluation of her infant's skill at bottle feeding, meeting the demands of the feeding schedule, and controlling her time and resources. This theme included the mother's perception of how successful she was at balancing her infant's frequent feedings with everyday demands such as grocery shopping, doing laundry, and caring for other children. In addition, a mother's previous experience with bottle feeding and how she used the support of other family members to manage this process was part of this theme*<br>*All of the mothers identified having the support of a husband, a significant other, or a family member(s); however, the mothers were the infants' primary care-givers. Mothers reported that family members were uncomfortable feeding their infants. Furthermore, mothers also reported that they were uncomfortable having other people feed their infants.*<br>Healthcare workers-Communication<br>Communicating information effectively: *The information provided in the NICU regarding feedings and well-baby care was considered appropriate and helpful. Mothers who were able to visit frequently while their infants were hospitalized had many opportunities to feed their infants before discharge and verbalized more comfort with the feeding process once home.*
Rohsiswatmo<br><br>(2014)	Infection<br>Sepsis: *Decrease blood stream infections Selected as key performance indicator*<br>Healthcare workers-Knowledge and Competence<br>Healthcare professional competence: *Processes thought to improve infection rates in NICU neonates: 'Education-socialization of employees; competence test; placement of employees according to competence; employee career path; renumeration'*<br>Survival<br>Survival: *Decrease mortality rate selected as key performance indicator*<br>Other<br>Duration of admission: *Decrease length of stay selected as key performance indicator*
Saigal (1999)	Development-Gross motor<br>Ability to walk: *Needs assistance to walk (from equipment/person)*<br>Development-Fine motor<br>Difficulties with activities of daily living: *Needs assistance/to use special equipment to eat, dress/bathe or use the toilet*

Confidential: For Review Only

	Development-Special senses<br>Hearing impairment: *deaf,*<br>Visual impairment: *Blind*<br>Development-Speech and Social<br>Language disorders: *Unable to talk*<br>Psychiatric<br>Mood disorders: *Occasionally fretful, angry, irritable, anxious, depressed, or experiencing 'night terrors'*<br>Need for educational support: *Learns schoolwork very slowly and needs special help*<br>Pain<br>Pain: *Sometimes has pain*<br>Normality<br>Normality: *Happy and not worried most of the time*<br>Other<br>Overall health state: *It appears that HPs* [Healthcare Professional] *and parents view the mild to moderately disabled health states similarly, but parents were more accepting of the severely disabled health states than HPs.*
Saunders<br><br>(2003)	Gastrointestinal<br>Breastfeeding: *Benchmarking questions identified: how do you encourage breastfeeding on your unit?*<br>Parental Support<br>Parental involvement: *Benchmarking questions identified: Does your unit welcome parents 24 hours/day? How are families encouraged and supported to participate in routine care of the infant? What provisions for privacy around the bedside do you offer? Are families encouraged to make their infants immediate environment as homelike as possible?*<br>Healthcare workers-Communication<br>Communication about discharge: *Benchmarking questions identified: Does your hospital provide teaching support for outside personnel? How ready do you feel you are to care for your infant after discharge from the hospital?*<br>Pain<br>Pain management: *Benchmarking questions identified: What tools are used for pain assessment?*<br>Suffering<br>Suffering: *Benchmarking questions identified; Are ventilated infants routinely sedated?*<br>Normality<br>Normality: *Benchmarking questions identified: During your infant's hospital stay, how often did you feel like a parent? Are families encouraged to make their infants immediate environment as homelike as possible?*
Sawyer (2014)	Healthcare workers-Communication<br>Communicating information effectively: *I was given all the information I needed Encouragement and reassurance,*<br>Healthcare workers-Knowledge and Competence<br>Healthcare professionals behaviour: *Staff professionalism and empathy The staff put me at ease The staff made me feel cared for as an individual*<br>*Staff being confident and in control Staff being calm in a crisis*

Schneiderman<br><br>(1997)	Suffering<br>Suffering: *Providing futile life-sustaining treatments "ridiculous," "dumb," "inappropriate," "insane," "outrageous," "frightening," "a teaching example of futility," "an example of what's wrong with medicine today,"*
Sharek (2003)	Respiratory<br>Chronic Lung Disease: *Outcomes identified by CLD focus group: Chronic Lung Disease rate Oxygen requirement at discharge home Pneumothorax rate*<br>Oxygen dependence: *Outcomes identified by CLD focus group: Chronic Lung Disease rate Oxygen requirement at discharge home Pneumothorax rate*<br>Pneumothorax: *Outcomes identified by CLD focus group: Chronic Lung Disease rate Oxygen requirement at discharge home Pneumothorax rate*<br>Development-Special senses<br>Retinopathy of Prematurity: *Outcomes identified by CLD focus group: ROP rates*
Short (2003)	Development-Gross motor<br>Need for physical therapy: *significantly more BPD children received...occupational, and physical therapy*<br>Development-Speech and Social<br>Language disorders: *significantly more BPD children received speech-language therapy*<br>Parental Support<br>Support from healthcare professionals: *Parents of children with a history of BPD reported that more of their children had occupational and physical therapy, including children who were without neurologic complications. The prolonged hospitalizations experienced by children with BPD and the frequent interactions of families with medical personnel may result in increased access and opportunities for services for parents of children with BPD.*<br>Healthcare utilisation<br>Frequent readmissions: *Parents of children with a history of BPD reported that more of their children had occupational and physical therapy, including children who were without neurologic complications. The prolonged hospitalizations experienced by children with BPD and the frequent interactions of families with medical personnel may result in increased access and opportunities for services for parents of children with BPD.*<br>Psychiatric<br>Need for educational support; *BPD children were more likely to be enrolled in lower grades than VLBW or term children. lacement in special education ser-vices, regardless of type, differentiated the groups, with 54% of the BPD, 37% of the VLBW, and 25% of the term children participating in special education.*
Sisk (2010)	Gastrointestinal<br>Choice of milk for feeding: *Universally, mothers perceived the pumping schedule as a huge weight on their shoulders because of the time it required. Mothers often spoke of the conflict between taking care of themselves and their other responsibilities and the need to pump...Mothers often delayed pumping to be at their infant's bedside for medical rounds, and it was unpredictable when those rounds would occur. Pumping at the bedside was an option; however, many mothers or their spouses were uncomfortable with this.*<br>*Attitudes toward milk expression influenced mothers' willingness to pump.*<br>*Anticipation of breastfeeding provided support for milk expression. Mothers who planned to breastfeed as opposed to exclusively bottle*

Confidential: For Review Only

	feed their breast milk viewed pumping as something they had to do.<br>Parental Support<br>Support from fathers: *Fathers ranged from being very supportive, "[we're] in this together," to being deterrents or completely absent.*<br>Parental ability to work: *Seven mothers returned to work within 3 weeks after delivery; none had paid maternity leave. They liked being back at work because it helped occupy their minds, but they reported being exhausted*<br>Coping with maternal illness: *Physical and mental challenges. The majority (59%) of mothers experienced a pregnancy-related medical complication, and almost half (44%) were treated with magnesium sulfate, a medication given to treat preterm labor and prevent seizures in severe preeclampsia. Mothers who received this medication stated that they had difficulty comprehending the pumping instructions or did not feel well enough to pump....*<br>*Mothers, especially those who were delivered by cesarean section, complained of extreme fatigue and desire for sleep in the first few days after delivery, and this interfered with pumping frequency and their ability to remember the pumping instructions*<br>*...Feeling anxious and stressed were common complaints during the immediate postpartum period. Lack of preparation for the preterm delivery and disruption of plans as well as concern for their infant's health, job, and finances distracted mothers from pumping frequently. For some mothers, however, anxiety regarding their infants' health motivated them to frequently pump.*<br>Barriers to parental involvement: *Lack of privacy was identified as a barrier to establishing a pumping schedule. Frequent interruptions and the flow of hospital staff, family, and friends interfered with their ability to learn to use the breast pump, relax during pumping, and pump frequently. Mothers disliked telling their visitors to leave, and they disliked pumping in front of them. Mothers stated that hospital staff members often were unable or unwilling to provide the amount of privacy they desired.*<br>*-availability of hospital grade breast pump helped transition from BF in hosp to BF at home*<br>*-pumping at home without stimulus of infant was more difficult*<br>*-transport to hospital to feed infants difficult*<br>Support from family and friends: *All mothers identified supportive people in their social networks. These included family members, friends, church members, and hospital staff. Practical assistance with chores (ie, child care for older children, meal preparation, and house cleaning) was critical to their success. The recognition and praise mothers received also motivated them to continue pumping.*<br>*-mothers relied on rides to hospital from family and friends, which sometimes worked, sometimes didn't in terms of feeding*<br>Balancing caring for themselves and their baby: *Universally, mothers perceived the pumping schedule as a huge weight on their shoulders because of the time it required. Mothers often spoke of the conflict between taking care of themselves and their other responsibilities and the need to pump...Mothers often delayed pumping to be at their infant's bedside for medical rounds, and it was unpredictable when those rounds would occur. Pumping at the bedside was an option; however, many mothers or their spouses were uncomfortable with this.*<br>*Anticipation of breastfeeding provided support for milk expression. Mothers who planned to breastfeed as opposed to exclusively bottle feed their breast milk viewed pumping as something they had to do*
Smith (2006)	Gastrointestinal<br>Breastfeeding: *"Because I think I understood the importance of what my wife was trying to do. It was extremely important for our babies, who are very fragile and sick, to receive breast milk."*<br>Parental Support<br>Support from fathers; *"I helped her as much as I could. I cleaned and carried the pump when necessary. I cleaned the pump kit when she was finished pumping, labelled the bottles, cleaned the bottles, transported the milk to the hospital, assisted with storage, and assisted her in whatever way I could while she was pumping."*

Confidential: For Review Only

Snowdon<br><br>(1997)	Parental Support<br>Support from faith: *"We regard Philippa as a gift from God and so when she was ill, you know we prayed that--we obviously prayed that we wanted to keep her but we knew that God would do what was best for her and for us and so I think although it was very worrying and very traumatic, there was a sort of peace underneath that actually"*<br>Healthcare workers-Communication<br>Communicating in challenging settings: *"Nobody can play God. You're making a decision on somebody's life and they don't really know so the computer decides and that's the best way because you're not blaming anybody then are you? You don't really want the onus on yourself really but I suppose the medical profession can't.., keep the onus on themselves"*<br>Survival<br>Survival: *" I think any parent wants to try anything, you know, they don't sit down and think it's research, there could be side effects or there could be abnormalities that might come up. I think as parents...your first instinct is to save your baby"*<br>*"I suppose what they're saying is that er if at the end of the day conventional really really really isn't going to work for him then that would be absolutely ideal because.., the chances are maybe he would be picked"*<br>*"I suppose trials have to be a bit heartless, but you'd think that when the baby looks like they're dying, you'd think they'd just say... "Oh hell" you know "'let's try the ECMO, see if it saves this baby" but with that sort of a trial they can't do that can they? They have to say, "Well look, this baby looks like it's dying but I'm sorry it's getting conventional treatment and that"*<br>*"your first instinct is to save your baby"*<br>*"we obviously prayed that we wanted to keep her "*<br>Other<br>Inclusion in research: *"I feel desperately sorry for parents who, you know, were turned down particularly if their child doesn't live. I think it would be hard but I can see that it is necessary in case...the research shows that ECMO is actually detrimental to children"*<br>Iatrogenic harm: *"I suppose trials have to be a bit heartless, but you'd think that when the baby looks like they're dying, you'd think they'd just say... "Oh hell" you know "'let's try the ECMO, see if it saves this baby" but with that sort of a trial they can't do that can they? They have to say, "Well look, this baby looks like it's dying but I'm sorry it's getting conventional treatment and that"*
Snowdon<br><br>(2014)	Parental Support<br>Coping with maternal illness: *All women who had a CS, some of whom needed additional interventions, such as Laura, who underwent surgical removal of her placenta, and Caitlin, who had reparative bowel surgery after damage caused during delivery, had to deal with the aftermath of their birth and surgery and their initial experiences in the NICU at the same time...Where women were debilitated postnatally this could limit their contact with their babies. Although a number were taken to the NICU in a wheelchair or their bed, when babies were moved to another hospital it was not always possible for the women to accompany them.*<br>*The mode and pace of delivery also impacted upon the men. Some of the men did not arrive at the hospital in time for the birth, as events could be unexpected and fast moving. When women underwent an emergency CS, men could be excluded from the birth, a potentially isolating and anxiety-provoking experience.*<br>Parental involvement: *Once babies were delivered and taken to the NICU, there was usually a lag before parents were able to visit. Some of the parents were frustrated at not being allowed to go to the NICU with their baby and the wait for news or to be allowed to visit could seem interminable.*<br>Healthcare workers-Communication<br>Communicating in challenging settings: *When they arrived at hospital in preterm labour, or were admitted for observation and bed rest, some had difficult conversations with clinical staff about the implications of their gestational stage for decisions about care. NICUs*

Confidential: For Review Only

	commonly set boundaries around the care that they offer, as their facilities determine their lower thresholds for care...Parents who were around the 23-/24-week cusp not only had to contend with the worry of whether or not their babies would survive and in what condition, but also they were faced with the possibility that intensive care would not be mobilised in their case. At the borderline of viability they had to wait to see whether their baby attempted to breathe to find out whether or not care would be initiated. This sense of ambiguity could be heightened when women were given antenatal steroids to help to mature their babies' lungs and improve their chances of survival, while also being faced with the possibility of no active care being offered postnatally.<br>Keeping parents informed: *Once babies were delivered and taken to the NICU, there was usually a lag before parents were able to visit. Some of the parents were frustrated at not being allowed to go to the NICU with their baby and the wait for news or to be allowed to visit could seem interminable.'*<br>Survival<br>Survival: *this trial spoke directly to their most pressing concerns; the threat to survival and the possibility of disability*<br>*The overwhelming sense from the parents interviewed for BRACELET was that at the time they wanted their babies to be cared for and for everything possible to be done. A minority of the parents who were facing extremely preterm birth came to their own decisions about care, which pre-empted NICU policy on admission.*<br>Survival without disability: *this trial spoke directly to their most pressing concerns; the threat to survival and the possibility of disability*<br>Other<br>Inclusion in research: *Parents interest in a trial appeared to develop over time as their bereavement receded. Although most had experienced little contact with a trial over the years, parents were often interested in the research, and some would have liked more contact and information than they actually received. Trial communications were valued as a source of information, as an acknowledgement of loss and the contribution to research, as a connection back to their baby and a form of commemoration. Some parents had kept trial paperwork in their baby's memory box.*<br>*Danielle's experiences of antenatal recruitment to BOOST-II UK were strikingly similar to Beverley's but they described different positions on altruism and enrolment. For Danielle her sense of altruism and the potential benefits for others was sustained throughout her association with the trial, but for Beverley it was more transient and dissipated somewhat once she was able to engage with the reality of her daughter's initial survival, condition and needs.*<br>*Discussion of the trial therefore required emotionally strained parents and physically debilitated mothers to engage not only with the information and choice offered to them in relation to cooling and the trial, but also confronted them at an early stage in their experience with the possibility of brain damage and disability for their newborn. Parents still reeling from delivery had to think about both the immediate and the long-term future.*<br>*Clearly parents come to neonatal intensive care via a range of different clinical pathways. Their reactions to the environment and to their babies are similarly varied and highly personal, bringing together emotions from recent and sometimes more distant events. It is against this background of threat, initial survival and initiation of care that they made their decisions about trial participation.*<br>*Ivan mentioned INIS right in the middle of this difficult time, as a direct response to the threat of serious infection for their baby. This places the trial into a therapeutic framework, and parents spoke of their hopes that INIS would make a difference, but by this point the gravity of their situation was sometimes clear and the trial could seem like a long shot rather than a magic bullet.*<br>*The offer of the trial, however, was disassociated from the crisis that the parents had experienced, partly because of the flexibility over timing and partly because the intervention related to a routine management of care.*<br>*Three main models of the potential effect of a trial could be identified in the data (Figure 16), which were characterised in the analysis as: -might help, won't harm -might help, might harm -won't harm, might help others'*

Confidential: For Review Only

Squitieri (2013)	Development-Fine motor<br>Difficulties with activities of daily living: *"A lot of times I have to put myself in certain positions to do things, like opening a jar- I have to squeeze it in between my arms instead of gripping it with my hand. And when I type, I have to position my fingers in a different way on the keyboard so I can reach the keys."*<br>*Most adolescents were extremely functional and able to accomplish basic activities of daily living due to the high degree of learned compensation. Many females reported difficulty grooming their hair*<br>Parental Support<br>Support from family and friends: *"My mother in law and my mother both would watch my older daughter that first year quite a bit while I would take my daughter to therapy"*<br>Healthcare utilisation<br>Frequent appointments: *Many parents commented on the time commitment of formal therapy visits. Depending on the ease of access to a nearby therapist, many adolescents would miss school and their parent would need to take off from work.*<br>Relationships with others<br>Peer acceptance: *Peer acceptance and fear of being teased or excluded from social groups was a prominent theme throughout our interviews. In addition, many adolescents commented on the importance and positive influence of obtaining support from other adolescents with NBPP through various camps, therapy groups, or online.*<br>Psychological coping: *Despite their impairments, most adolescents had a positive view of themselves overall. Negative coping mechanisms were more common among younger adolescents and adolescents with greater levels of impairment. Older adolescents generally dealt with their impairments in a constructive manner either through learned compensation techniques, acceptance, or anticipation and alteration of activities if they believed their impairment would limit them in any way.*<br>Family resources: *All participants in our study had some form of insurance coverage. However, some parents reported frustrations regarding the cumbersome process of talking with insurers to confirm coverage or extended therapy visits.*<br>Effects on family and friends: *Almost all parents acknowledged the emotional adjustment of other family members in response to raising a child with physical impairment. Many relied on the help of extended family. Few parents reported negative effects on parental/sibling relationships.*<br>Pain<br>Chronic pain: *Occasionally some patients or their parents reported hypersensitivity or mild discomfort with therapy exercises or prolonged activity. No patients reported significant pain that impaired daily living. "She occasionally complains of pain in her shoulder area. Maybe 1-2 times per month."*<br>Suffering<br>Comfort: *Patient priorities: To sleep more comfortably*<br>Normality<br>Normality: *"My arm used to make me mad and sad because I couldn't be like everybody else… 'why can't I raise my hand like this?' and stuff like that."*<br>Other<br>Physical appearance: *"I got teased in the fourth grade. They'd call me 'overblown bicep' and 'fat arm.' Sometimes I would get really self-conscious and discouraged and think that my arm looks weird and that people are staring at me."*<br>*"I don't like wearing half sleeves or quarter length sleeves because it looks like a long sleeve on my right (palsy) arm but a quarter sleeve on my left (non-palsy) arm."*

Confidential: For Review Only

	Almost all adolescents and parents commented on aesthetics and body image regarding arm position, length/size discrepancies, and clothing restrictions.
Stevens (2014)	Gastrointestinal<br>Oral feeding: *"(The) very first time (feeding the baby) was just great, to tell you the truth. It was an experience, just like, 'Wow'…it was just looking into him and just like, 'wow, look what we made, 'know?' It was just crazy…it was just wonderful."*<br>Relationships with others<br>Bonding with parents: *"It was pretty awesome [laughing] because (his daughter) had been in an incubator for a while and we couldn't hold her and then we actually got to hold her. Holding a baby is something that I've always wanted so to me it was priceless"*<br>Normality<br>Normality: *"It was pretty awesome [laughing] because (his daughter) had been in an incubator for a while and we couldn't hold her and then we actually got to hold her. Holding a baby is something that I've always wanted so to me it was priceless"*<br>Growth<br>Growth: *"you want to be sure that they take everything so that they're gaining weight adequately"*
Thoren (2013)	Parental Support<br>Online support: *"This group is for parents who have experienced the miracle of having a preemie. Let's share our stories to help each other through the difficult times of NICU and support each other through the even more challenging task of raising a preemie."*
Thoyre (2000)	Gastrointestinal<br>Oral feeding: *"Just get the bottle in him. That's all. "*
Turrill (2003)	
Turrill (2003)	Neurological<br>Significant IVH: *"By then.. em.. I think we're coming off the big risk of nasty big haemorrhages as well, unless something seriously goes wrong with them for some obscure reason."*<br>Development-Special senses<br>Visual impairment: *Children needing to wear hearing aids or glasses*<br>Hearing impairment: *Children needing to wear hearing aids or glasses*<br>Parental Support<br>Parental involvement: *Reduced parental contact was cited as a characteristic of increasing risk for alterations to development...Support of the family unit in hospital is essential to the future wellbeing of that family. However what that part of care has to play in the long term development outcomes of newborns who receive intensive care is unknown. Although parental support is a vital aspect of their role, relating this to developmental outcomes is misguided and again, highlights the lack of a common knowledge base supporting practice.*<br>Healthcare workers-Communication<br>Communication with parents: *"When you're talking to parents while you're doing cares and everything, you're not really talking to them,... you're having a vague conversation across the room"*<br>*Communication between professionals p.33 'Beyond the immediate admission, relationships betwen nurses and doctors and their differing roles and responsibilities appeared at times in conflict, with little team approach to care.'*<br>Healthcare workers-Knowledge and Competence<br>Healthcare professional competence: *nurses' lack of knowledge of short and long term LBW infant outcomes: lack of evidence-based*

Confidential: For Review Only

	guidelines to support nurses use; too lengthy; info not seen as important or relevant to their role; lack of time; no formal channels to disseminate information<br>*nurses lack understanding that 80% of LBW infants develop normally, and what 'normally' is, means that they don't feel able to foster that normality in NICU care*<br>*'The physical stability of babies must remain the nurses first priority when considering optimal brain function in relation to future outcomes. However, the importance of controlling the neonatal environment to increase stability appeared to have been lost. The constant intensiveness of the working environment, while recognised as not normal, has been taken for granted.*<br>*The quality of physical care given to a VLBW baby in the first 24 hours is vital to his or her survival and future development. The responses from nurses in clinical and managerial positions showed they were acutely aware of this priority...four [higher grade] nurses had changed priorities [in the scenario], with parents gaining importance over time.*<br>*Nurses recognized that their limited knowledge, in-service education and research were seen to affect their decision-making abilities, confidence and consequent autonomy. The current strategy for continuing education is affected by the fact that nurses are rarely able to leave the cot-side to take part in meetings, seminars or reflective groups due to the pressure of clinical commitments. Also access to a clinical academic neonatal pathway is not currently available within the unit's education contracts'*<br>Consistency of decisions: *Beyond the immediate admission, relationships between nurses and doctors and their differing roles and responsibilities appeared at times in conflict, with little team approach to care.*<br>Normality<br>Normality: *nurses lack understanding that 80% of LBW infants develop normally, and what 'normally' is, means that they don't feel able to foster that normality in NICU care*<br>*With the emphasis on normal development being lacking in their knowledge base, the effects of interventions appear weighted towards a failure to achieve the ideal at the start. The level of responsibility the nurses felt they held towards the baby's future, coupled with this high risk of failure, posed an unrealistic challenge.*<br>Growth<br>Growth: *nutrition relates to growth and thriving*<br>Other<br>Healthcare resources: *The level of resources was thought to have the greatest impact on the nurses' ability to carry out their role, in particular the nursing resource.*<br>*'When considering what aspects of service impacted on their ability to carry out the care they ideally wanted to give, limited available time was important. With a workforce of only 62% of the regionally-funded QIS level, the constant pressure had a great impact on managing priorities for care.*
van Zuuren<br><br>(2006)	Development-Gross motor<br>Issues of development and motor skills: *"will it stay dependent on the care of oth-ers for the rest of his life, will it remain spastic, will it be wheelchair-bound?"*<br>Development-Speech and Social<br>Language disorders: *"Will the child be able to communicate in the future"*<br>Healthcare workers-Communication<br>Communicating in challenging settings: *Uncertainty, in particular unpredictability of the outcome in the child, is seen as a complicating factor in end-of-life decisions, and, to a lesser extent, in the problem of medical damage.... Long-term follow up is advocated in order to reduce this uncertainty.*

Confidential: For Review Only

	Suffering<br>Suffering: *It was the nurses who most explicitly pointed to the direct suffering that treatment devices often impose on the child and who sometimes resented the parents' or doctor's wish to prolong treatment. For these nurses, there is often an imbalance between two important ethical principles: doing well and avoiding harm....So, clear disadvantages of doing well (treatment) are the immediate suffering of the newborn and the possible damage in the future.*<br>Other<br>Survival with disability: *"The greatest dilemma has to do with the handicaps we induce with our treatment." "A child with mild handicaps can have a good life. A child that hardly makes any developmental progress and that will be dependent on others all along .... (unfinished)."*
Vandenbussche<br><br>(1999)	Neurological<br>Neurological symptoms *-Mothers' and obstetricians' valuing of 4 types of infant outcome: -healthy infant -transient neurologic symptoms -permanent neurologic symptoms -neonatal death*<br>Survival<br>Survival: *Pregnant women and mothers valued an infant with permanent handicap significantly higher than neonatal death, and this choice was irrespective of the type of birth (all p values < 0.01). Obstetricians, on the other hand, awarded lower, albeit not statistically different median values to permanent handicap than to death.*<br>Survival with disability: *Pregnant women and mothers valued an infant with permanent handicap significantly higher than neonatal death, and this choice was irrespective of the type of birth (all p values < 0.01). Obstetricians, on the other hand, awarded lower, albeit not statistically different median values to permanent handicap than to death.*
Votteler (2005)	Genitourinary<br>Urological disorders: *Persistent urological problems concern twin A's mother as to whether he will be accepted or teased by his peers when he is older.*<br>Surgical<br>Need for multiple operations: *Three twins required scoliosis correction in later child-hood. Neurosurgical problems have been significant in both 1988 pygopagus sets, requiring long-term evaluation.*<br>Development-Gross motor<br>Ability to undertake sport: *They report feeling self-conscious of their appearance and avoid athletic activity, which calls attention to their physique. She cannot participate in physical education because of orthopedic and gait problems.*<br>Development-Special senses<br>Hearing impairment: *Social communication is difficult because of his hearing and speech problems, and he is described as having a few friends and no experience in dating.*<br>Development-Speech and Social<br>Language disorders: *Social communication is difficult because of his hearing and speech problems, and he is described as having a few friends and no experience in dating.*<br>Social difficulties: *Social communication is difficult because of his hearing and speech problems, and he is described as having a few friends and no experience in dating.*<br>Healthcare utilisation<br>Need for frequent treatments: *Three twins required scoliosis correction in later child-hood. Neurosurgical problems have been significant*

Confidential: For Review Only

	in both 1988 pygopagus sets, requiring long-term evaluation.<br>Psychiatric<br>Need for educational support: *The patient is at an age-appropriate grade level but attends resource classes in math and achieves only average grades in other areas. The girls are at an age-appropriate grade level and earn mostly As with some Bs. Both aspire to pursue college and graduate school education.*<br>Psychiatric disorder: *The other boy has been diagnosed with autism and bipolar disorder and is receiving care in psychiatry, neurology, occupational therapy, and speech. The mother is very focused on the boys' physical and emotional symptoms*<br>Normality<br>Normality: *Her parents consistently emphasized her normalcy and need to gain independence. The parents had a second child to 'normalize' life for their surviving son.*<br>Survival<br>Survival: *His mother reports a continuing sense of sadness over the neonatal death of the patient's twin. A recent first-time exposure to a photograph of this deceased infant was described as extremely meaningful and comforting. His parents report significant sorrow over the loss of twin B and the mother eventually went through counseling to work through these grief issues. Twin A's young parents are still grieving the death of twin B and are still questioning their decision making in terms of that twin's care.*<br>Other<br>Physical appearance: *They report feeling self-conscious of their appearance and avoid athletic activity, which calls attention to their physique.*
Wielenga<br><br>(2015)	Respiratory<br>Mechanical ventilation: *Research priorities identified: ...(6) respiratory and ventilation;*<br>Gastrointestinal<br>Feeding practices: *Research priorities identified: Identifying best practices in enteral feeding Identifying best practices breast feeding Improving nutrition in preterm and sick term infants*<br>Neurological<br>Neurological care: *Research priorities identified: Identifying the best care practices for infants with neurological problems*<br>Infection<br>Sepsis: *Research priorities identified: Evaluating infection prevention strategies Identifying and evaluating interventions to monitor and reduce hospital-associated infections Evaluating sepsis management and care to improve outcomes*<br>Skin<br>Skin care: *Research priorities identified: Identifying and evaluating interventions to improve skin and wound care in neonates*<br>Surgical<br>Care for surgical babies: *Research priorities identified: Identifying the best care practices for surgical infants*<br>Parental Support<br>Parental involvement: *Research priorities identified: Identifying strategies to support the needs of parents and family members Identifying and evaluating strategies to support parental attachment Identifying best practices for the implementation of family centred care Evaluating the role and involvement of parents in the care of their infant Improving end-of-life care for neonates and their families Exploring the role of parents in ethical decision making*<br>Healthcare workers-Knowledge and Competence<br>Healthcare professionals behaviour: *Research priorities identified: (3) clinical nursing care practices; (4) quality and safety; (5) ethics; and*

Confidential: For Review Only

	(8) professional issues in neonatal intensive care nursing.<br>Pain<br>Pain management: *Research priorities identified: Identifying effective interventions to prevent or reduce pain or stress Identifying best practices for pain assessment Identifying pain and/or stress guidelines*

Confidential: For Review Only

eFigure 2: Results Of Permutation Test Analysis Regarding Gestational Age

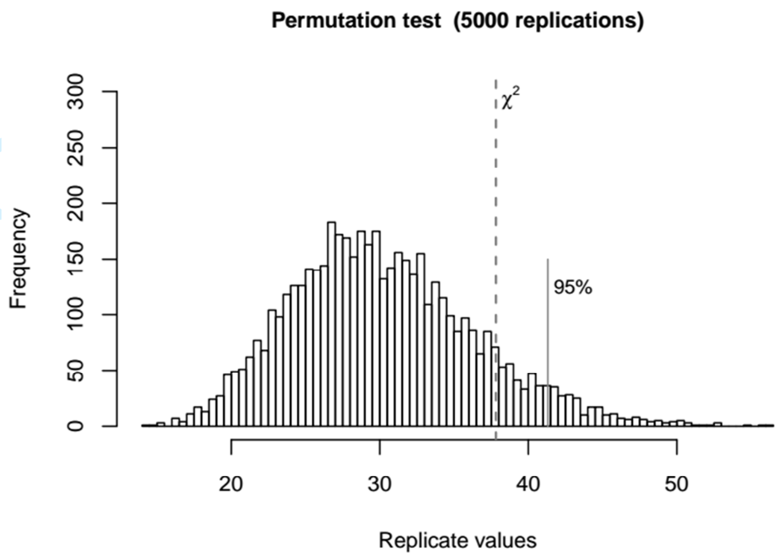

Permutation test analysis for association of gestational age of babies within a study and outcome categories identified within study. The diagram presents the null-distribution of the chi-square (goodness-of-fit) statistic obtained by simulations. The X axis describes the "replicate value" assigned to each replicate while the Y axis describes the frequency with which each replicate value arose during the 5000 replications. The distribution does not have the chi-square shape because some articles/papers contribute with more than one entry in the contingency table of parties-by-topics. The vertical dashes (χ^2) mark the value of the statistic (evaluated on the observed dataset) and the vertical solid line (95%) is the critical value, where the distribution has its 95th percentile. The value of the statistic is 37.82, the critical value is 41.31 (p-value=0.114).

For peer review only

eFigure 3: Results Of Permutation Test Analysis Regarding Stakeholder Group

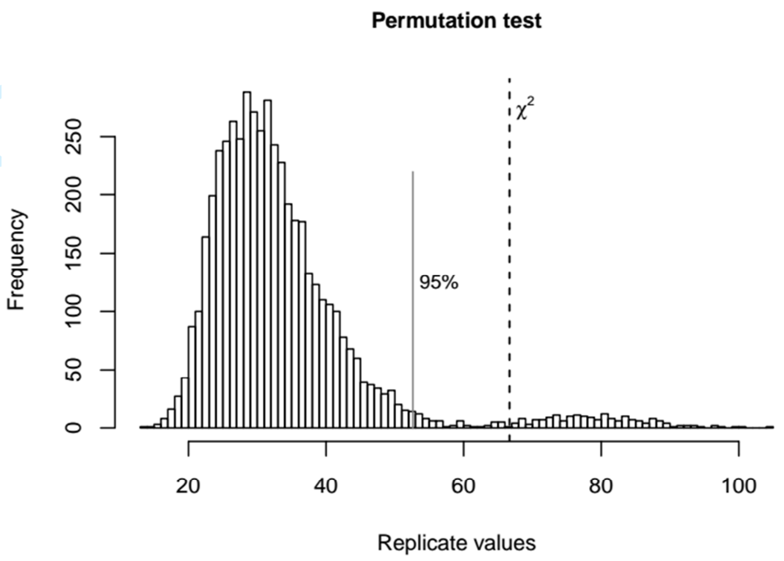

Permutation test analysis for association of stakeholder groups within a study and outcome categories identified within study. The diagram presents the null-distribution of the chi-square statistic obtained by simulations. The X axis describes the "replicate value" assigned to each replicate while the Y axis describes the frequency with which each replicate value arose during the 5000 replications. The distribution does not have the chi-square shape because some articles/papers contribute with more than one entry in the contingency table of parties-by-topics. The vertical dashes (χ^2) mark the value of the statistic (evaluated on the observed dataset) and the vertical solid line (95%) is the critical value, where the distribution has its 95th percentile. The value of the statistic is 66.67, the critical values is 52.62 (p-value=0.037).

For peer review only

BMJ Paediatrics Open

Parent, patient and clinician perceptions of outcomes during and following neonatal care: a systematic review of qualitative research

Journal:	*BMJ Paediatrics Open*
Manuscript ID	bmjpo-2018-000343.R1
Article Type:	Original article
Date Submitted by the Author:	09-Aug-2018

Complete List of Authors:	Webbe, James; Imperial College London, Academic Neonatal Medicine<br>Brunton, Ginny; University College London Institute of Education<br>Ali, Shohaib; Imperial College London Faculty of Medicine<br>Longford, Nicholas ; Imperial College London, Department of Medicine<br>Modi, Neena; Imperial College London, Neonatal Medicine<br>Gale, Chris; Imperial College London, Academic Neonatal Medicine

Keywords:	Neonatology, Outcomes research, Patient perspective

SCHOLARONE™
Manuscripts

Confidential: For Review Only

Parent, patient and clinician perceptions of outcomes during and following neonatal care: a systematic review of qualitative research

James Webbe[1], Ginny Brunton[2], Shohaib Ali[3], Nick Longford[4], Neena Modi[5], Chris Gale[6] on behalf of the Core Outcomes in Neonatology (COIN) project steering group*

* Collaborative group listed below

[1] James Webbe, MB BChir

Neonatal Medicine, Imperial College London

j.webbe@imperial.ac.uk

[2] Ginny Brunton, PhD

UCL Institute of Education, London

g.brunton@ucl.ac.uk

[3] Shohaib Ali

Imperial College, London

shohaib.ali14@imperial.ac.uk

[4] Nicholas Longford, PhD

Neonatal Medicine, Imperial College London

n.longford@imperial.ac.uk

[5] Neena Modi, MD

Confidential: For Review Only

Neonatal Medicine, Imperial College London

n.modi@imperial.ac.uk

[6] Chris Gale, PhD

Neonatal Medicine, Imperial College London

christopher.gale@imperial.ac.uk

Confidential: For Review Only

Members of the COIN Steering Group

Elsa Afonso

Neonatal Staff Nurse. Cambridge University Hospitals NHS Foundation Trust, Cambridge.

Iyad Al-Muzaffar

Consultant Neonatologist and Parent of preterm baby. Cwm Taf University Health Board, Wales.

Ginny Brunton

Midwife and Research Methodologist. UCL Institute of Education, London.

James Duffy

Doctoral Research Fellow in Obstetrics and Gynaecology. Nuffield Department of Primary Health Sciences, Oxford.

Chris Gale

Clinical Senior Lecturer in Neonatal Medicine. Imperial College, London

Anne Greenough

Professor of Neonatology and Clinical Respiratory Physiology. King's College, London.

Nigel Hall

Associate Professor of Paediatric Surgery. University of Southampton, Southampton.

Marian Knight

Professor of Maternal and Child Population Health. National Perinatal Epidemiology Unit, Oxford

Jos Latour

Professor of Clinical Nursing. Plymouth University, Plymouth.

Neil Marlow

Professor of Neonatal Medicine. University College, London.

Neena Modi

Professor of Neonatal Medicine. Imperial College, London.

Laura Noakes

Parent of neonatal patient.

Julie Nycyk

Consultant Neonatologist, Sandwell and West Birmingham Hospitals NHS Trust, West Midlands.

Mehali Patel

Research Engagement Officer. Bliss, London.

Angela Richard-Londt

Parent of neonatal patients.

James Webbe

Clinical Research Fellow. Imperial College, London.

Ben Wills-Eve

Ex-neonatal patient.

Corresponding Author

Chris Gale, Senior Clinical Lecturer in Neonatal Medicine

Section of Neonatal Medicine, Chelsea and Westminster Campus, Imperial College London

369 Fulham Road, London, SW10 9NX, United Kingdom

+ 44 (0)203 3153519

christopher.gale@imperial.ac.uk

Confidential: For Review Only

Manuscript word count: 2600

ABSTRACT

Objective

Multiple outcomes can be measured in infants that receive neonatal care. It is unknown whether outcomes of importance to parents and patients differ from those of health professionals. Our objective was to systematically map neonatal care outcomes discussed in qualitative research by patients, parents and healthcare professionals and test whether the frequency with which outcomes are discussed differs between groups.

Design

Systematic review of qualitative literature. The following databases were searched: Medline; CINAHL; EMBASE; PsycINFO and ASSIA from 1997 to 2017. Publications describing qualitative data relating to neonatal care outcomes, reported by former patients, parents or healthcare professionals, were included. Narrative text was analysed and outcomes grouped thematically by organ system. Permutation testing was applied to assess an association between the outcomes identified and stakeholder group.

Results

Sixty-two papers containing the views of over 4100 stakeholders were identified; 146 discrete outcomes were discussed; 58 outcomes related to organ systems and 88 to other more global domains. Permutation testing provides evidence that parents, former patients and health professionals reported outcomes with different frequencies (p=0.037).

Conclusions

Parents, patients and health professionals focus on different outcomes when discussing their experience of neonatal care. A wide range of neonatal care outcomes are reported in qualitative research; many are global outcomes relating to the overall status of the infant. The views of former patients and parents should be taken into consideration when designing research; the development of a core outcomes set for neonatal research will facilitate this.

Abstract word count: 244

Confidential: For Review Only

INTRODUCTION

In high resource settings approximately one in ten babies will require care in a neonatal unit (1). Conditions such as preterm birth affect patients' long-term outcomes: consequences include cardiovascular disease in adulthood (2), neurosensory impairment (3), respiratory disease (4), and lower rates of employment and marriage (5). Infants born more prematurely tend to have worse outcomes (6). As neonatal survival for babies of all gestational ages improves long-term outcomes become more important.

An outcome is the measured effect that illness or treatment has on an individual (7). Parents and patients are rarely involved in outcome selection in paediatric research (8). Poor outcome selection causes research waste (9): research produced is not relevant to patients' lives. Neonatal care, and the underpinning research, should focus on outcomes important to those it affects most: former neonatal patients, parents and healthcare professionals (9, 10). Identifying these outcomes is crucial to ensure research is relevant and efficient (9, 11). Qualitative research provides a rich description of complex phenomena such as neonatal care (12). One commonly used approach to identify outcomes of importance to stakeholders is primary qualitative research. Considerable qualitative research exploring how parents and health professionals perceive neonatal care has been conducted previously (13, 14); therefore by systematically reviewing published qualitative research it is possible to map the outcomes discussed by different groups. This review does not include all research on how stakeholders perceive neonatal care: it is focused on how former patients, parents and health professionals perceive the outcomes of this care.

Confidential: For Review Only

In this study we aimed to map the range of outcomes identified in qualitative literature by different stakeholder groups: parents, ex-neonatal patients and healthcare professionals. We also wanted to test the hypotheses that stakeholder groups prioritise outcomes differently, and that outcomes identified differ by infant gestational age category.

This work is a component of a wider programme to compile a core outcomes set for neonatology (15). A core outcomes set is an agreed collection of important outcomes identified through robust consensus methods by all key stakeholder groups (7). The results of this study will be combined with the results of a systematic review of outcomes reported in clinical trials (16). These will be used as the starting point for the consensus process to determine a core outcomes set (15).

METHODS

We registered this systematic review prospectively on PROSPERO (prospective register of systematic reviews): CRD42016037874 (17). We conducted the review according to PRISMA guidelines (18). We searched the following databases: Medical Literature Analysis and Retrieval System Online (MEDLINE); Cumulative Index to Nursing and Allied Health Literature (CINAHL); Excerpta Medica Database (EMBASE); Psychological Information Database (PsycINFO) and Applied Social Sciences Index and Abstracts (ASSIA). Qualitative or mixed method studies were included if they contained outcomes identified by stakeholders in the context of babies admitted to a neonatal unit. Full inclusion and exclusion criteria are listed in eTable 1. We considered all studies published from 1st January 1997 to 1st January 2017 in a peer review journal in all languages (where necessary a translation was obtained). The databases were last searched on 14th February 2017. The search strategy utilised for MEDLINE is described in eFigure 1. The terms derived from this search strategy were translated to other databases.

Confidential: For Review Only

All identified papers were screened by title and abstract and then by full text. After double-screening a sample of papers and agreeing criteria all screening was completed by one researcher (JW). For quality assurance, a second researcher screened a random 10% sample of abstracts and titles (CG). Agreement between reviewers was assessed by Cohen's kappa coefficient (19).

After screening all papers were coded independently by two researchers (JW and CG or GB) using Eppi-Reviewer 4 software (20). Any disagreement was resolved by a third researcher (CG or GB). Data on study design, stakeholder demographics, infant birth characteristics and verbatim text relating to neonatal care outcomes were extracted and stored. Quality assessment of individual studies was not undertaken as it is a controversial area of uncertain value in relation to qualitative research (21).

All outcomes were grouped according to a previously defined framework of organ systems (22) using the following domains: cardiovascular; respiratory; gastrointestinal; neurological; genitourinary; infection; skin and development. All three reviewers jointly refined this framework using methods incorporating thematic analysis (23-25). Where narrative data did not fit clearly into the domains, dialogue between all reviewers was used to develop new domains. Outcome domains were thematically analysed to develop higher order categories. A new hierarchy was developed to group outcomes because established hierarchies either did not relate well to neonatal care (26-28) or missed key concepts (7). This outcome hierarchy is described in text box 1.

Confidential: For Review Only

Text box 1

An example of an outcome hierarchy:<br>Text extracts to identify or infer a result of clinical care, the ‘outcome’ such as “*Bonding with parents*”.<br>Similar ‘outcomes’ were grouped into thematically linked ‘domains’ such as “*Relationships with others*”.<br>‘Outcome domains’ relating to similar concepts were grouped into ‘categories’ such as “*Social*”.<br><br>We did not address the ways in which an outcome was measured. For example the ‘outcome’ “*Parental bonding*” could be measured using parent reported scores or an external assessment.

We analysed whether outcomes identified differed by stakeholder groups and by infant gestational age category (using WHO definitions of prematurity) (29). We used permutation testing (30) to test for an association between the frequency that outcomes in different domains were identified and the stakeholder group involved. We performed five thousand replications to generate the distribution of the test statistic under the null hypothesis of no association, and compared our results to this distribution. We performed a similar analysis to test for an association between infant gestational age and frequency of outcome reporting. If a significant association was found we explored this further in a post-hoc analysis to identify where the observed results differed most from the frequencies expected under the hypothesis of no association established by the permutation analysis.

Confidential: For Review Only

RESULTS

Database searches produced 1130 results which were screened and assessed for eligibility (Figure 1). After applying inclusion and exclusion criteria 62 studies containing the views of 4100 stakeholders were analysed. Agreement between reviewers was high (Cohen's kappa coefficient=0.81) (19).

The 62 included studies reported data from 15 countries; 9 related to full-term infants, 31 to preterm infants (born <37 weeks gestational age) and 20 to extremely preterm infants (born <28 weeks gestational age). A range of methodologies was used including direct observation (13 studies) and individual (25 studies) or group interviews (13 studies). Questionnaires were used in 21 studies, two of which were Delphi processes. Included studies are described in eTable 2.

Included studies involved over 4100 participants. Parents were the most frequently involved stakeholder group (1969 parents in 40 studies; 65%); former neonatal patients were less commonly included (368 patients in 5 studies; 8%). Nurses and midwives were the professional group involved most often (1096 involved in 24 studies; 39%). Three hundred and sixteen doctors were involved in 18 studies (29%). We also identified 351 additional participants consisting of other family members, teachers, social workers and allied health professionals. In many studies, particularly those employing observation of clinical practice, the total number of research participants was not recorded.

One hundred and forty six distinct outcomes were extracted from the included studies. Fifty eight outcomes related to organ systems within the original framework; we were unable to categorise 88 outcomes within the original framework. The final framework is shown in

Confidential: For Review Only

Table 1. An example of the thematic analysis leading to the expanded framework is shown in Text Box 2.

Table 1 Final Outcome Framework

**Outcome Domain Categories**	**Outcome Domains**
Organ system outcomes	Cardiovascular
	Respiratory
	Gastrointestinal
	Respiratory
	Neurological
	Genitourinary
	Infection
	Skin
	Developmental
Holistic outcomes	*Survival*
	Growth
	Pain
	Suffering
	Normality
	Other outcomes
Parent focussed outcomes	*Parental support*
	Other outcomes
Healthcare delivery outcomes	*Healthcare workers - Knowledge and Competence*
	Healthcare workers - Communication
	Other outcomes
Economic outcomes	*Healthcare utilisation*
	Other outcomes
Social outcomes	*Psychiatric outcomes*
	Relationships with others
	Other outcomes

Outcome domain categories and outcome domains added to the original framework marked in *italics*

Text box 2

Example of framework synthesis related to the outcome of 'Normality'. Thematic analysis of verbatim extracts identified a recurring theme:

"The mother also worried that...Lisa would not have a normal life" (31)

"Being reassured that he was on line for how old he was...Just reassurance he was doing well" (32)

Confidential: For Review Only

"Finally, a mother called it a developmental land-mark when an older sister dared show her irritation towards her little brother, 'no longer treating him as if he were made of glass'" (33)

From this and similar text the outcome of 'Normality' was derived by thematic analysis. It did not fit within the existing outcome hierarchy but was reported extensively, so a new domain was added to the framework again called "Normality". This outcome domain relating to the overall status of the infant was similar to outcome domains like "survival", "vitality" and "growth" so these domains were grouped together as an outcome domain category called: "Holistic outcomes".

The full inventory of outcomes is listed in eTable 3. A table of all outcomes in each study (with verbatim text extracts) is shown in eTable 4.

Outcomes were identified relating to all of the organ systems included in the original framework and assigned to an organ system outcome domain category (Table 2). The organ system outcome domains most frequently discussed at the study level were "development" (32 studies; 52%) and "gastrointestinal" (24 studies, 39%). The individual organ system outcomes most frequently discussed were "language disorders" (8 studies, 13%), "visual impairment" (7 studies, 11%) and "breastfeeding" (7 studies, 11%).

Confidential: For Review Only

Table 2 Organ System outcomes

Organ System Outcome Domains	Number of studies discussing outcome domain *(n=62)*	Outcome	Number of studies discussing outcome *(n=62)*	Verbatim text extract
Developmental	32	Ability to walk	3	*"He walked four, my mother never forgot, she says it was a miracle of God" (34)*
		Difficulties with activities of daily living	4	*"A lot of times I have to put myself in certain positions to do things, like opening a jar- I have to squeeze it in between my arms instead of gripping it with my hand." (35)*
		Hearing impairment	5	*"I told the parents that he will never be able to see, to hear and I will get more data to show them how bad things are." (36)*
		Issues of development and motor skills	5	*"We both looked at my child, research, experience and what I could expect." (37)*
		Language disorders	8	*"I also had a hard time learning to talk." (38)*
		Social difficulties	2	*Social communication is difficult because of his hearing and speech problems, and he is described as having a few friends and no experience in dating (39)*
		Visual impairment	7	*At the time of the interviews, the only major sequel was in one child with seriously impaired vision. (33)*
		Other outcomes only in 1 paper		Ability to feed themselves: Ability to undertake sport: Need for physical therapy: Normal hearing: Retinopathy of prematurity
Gastrointestinal	24	Breastfeeding	7	*"I fully breastfed for four months-100%-and I am so proud of it." (37)*
		Choice of milk for feeding	2	*"it's like they [scn providers] didn't inform us when they were trying to feed my daughter [formula]" (40)*
		Feeding difficulties	5	*"We kept on saying to the staff on neonatal unit that it was only Gray's feeding that was stopping him from going home, everything else was fine" (41)*
		Feeding practices	2	*Mothers had difficulty understanding these instructions and seemed hesitant to liberalize their infant's intake after discharge. (42)*
		Initiating enteral feeds	2	*"MEF* [minimal enteral feeds] *should be initiated in first 2-3 days of life as long as the baby is stable." (43)*
		Oral feeding	3	*"(The) very first time (feeding the baby) was just great, to tell you the truth." (44)*
		Other outcomes only in 1 paper		Choking during feeding: Eating disorder: Fistulas: Frequency of defaecation: Liver failure: Necrotising enterocolitis: Nutritional intake: Other gastrointestinal malformations: Regurgitation: Short gut syndrome
Respiratory	12	Frequent respiratory illnesses	2	*"There were lots of masks and nebulisers during those years" (33)*
		Mechanical ventilation	5	*Over 30% of all infant descriptions were about babies who had tracheostomies and were unable to be weaned off a ventilator. (45)*
		Oxygen dependence	5	*"My babies did not fit into the criteria for going home early due to one of the twins still being dependent on oxygen." (41)*

Confidential: For Review Only

		Other outcomes only in 1 paper		Asthma: Breathlessness: Chronic Lung Disease: Excessive secretions: Nasal congestion: Pneumothorax
Neurological	11	Brain damage (not further specified)	2	*“Brain injury is laden with a lot more emotions and moral concerns for sure” (46)*
		Neurological symptoms	2	*“can’t feel some - my left - this is numb right here.” (47)*
		Seizures	2	*“I explained this to the doctor. And he was the one that said it could possibly be seizures. So we're going to take him in and have him tested.” (48)*
		Significant IVH	2	*“Although she has a grade IV bleed, the resident says that she moves and looks around, and he thinks the odds are quite good.’’ (36)*
		Sleep disorders	4	*Subsequent to an account of the son's disturbed sleep at night for several months after discharge, which was an enormous challenge to the couple (33)*
		Other outcomes only in 1 paper		Neurological care
Surgical	5	Appearance of scars	2	*“I do not like the scar on my belly [...]; I was at the beach and everyone kept staring at me like `That is a big scar!´” (38)*
		Need for multiple operations	2	*the mother also worried that there would be more surgeries (31)*
		Other outcomes only in 1 paper		Care for surgical babies: Need for ileostomy
Infection	5	Sepsis	3	*Decrease blood stream infections Selected as key performance indicator (49)*
		Other outcomes only in 1 paper		Prevention of infection: Susceptibility to infection
Skin	4	Appearance of scars	2	*In addition, hospitalization and different interventions in their first days of life have left marks on their bodies. (38)*
		Other outcomes only in 1 paper		Burns: Extravasation injuries: Pressure sores: Skin care
Cardiovascular	1	Other outcomes only in 1 paper		Hypotension: Presence of patent ductus arteriosus
Genitourinary	1	Other outcomes only in 1 paper		Urological disorders

Confidential: For Review Only

The majority of outcomes identified did not relate to individual organ systems. Some related to the overall status of the infant and were assigned to a holistic outcome domain category (Table 3). Other domains related to the effects experiencing neonatal care has on parents; these were assigned to a "Parent focussed" outcome domain category (Table 4). Another group of domains related to the neonatal care delivered; these were assigned to a "Healthcare delivery" outcome domain category (Table 5). A group of domains was identified relating to the cost of neonatal care; these were assigned to an "Economic" outcome category (Table 6). Finally a group of outcome domains was identified relating to the relationships neonatal patients develop with others; these were assigned to a "Social" outcome domain category (Table 7).

Confidential: For Review Only

Table 3 Holistic outcomes

Holistic Outcome Domains	Number of studies discussing outcome domain *(n=62)*	Outcome	Number of studies discussing outcome *(n=62)*	Verbatim text extract
Normality	22	Ability to lead a normal life	2	*“the mother also worried that...Lisa would not have a normal life” (31)*
		Normality	16	*A major focus for parents was seeking information that told them that what was happening was ‘normal’ and that everything was going to be ‘fine’. (50)*
		Other outcomes only in 1 paper		Being treated normally: Inability to create a normal life: Normal health: Thriving
Suffering	15	Comfort	4	*"You can almost feel what it’s like in the incubator, lying on the lambskin, that it's how I would want to have laid and . . . Well, it looks very comfortable" (51)*
		Suffering	9	*"This infant's short life was never comfortable...I frequently felt we were torturing the child just doing daily care." (45)*
		Other outcomes only in 1 paper		Ex-patients separation from their suffering: Symptom control
Survival	14	Survival	11	*"It hurts. I didn't know, I didn't know if they were going to make it or not." (48)*
		Survival with disability	3	*"It isn't up to us to say what is quality of life, because parents might think that even if the child was disabled, that it was better than not having a child at all." (52)*
		Survival without disability	4	*"And afterwards you are worried about how they are going to survive. If they would have impairments, and so on." (33)*
Growth	8	Growth	8	*"She was born so early, it's nice to see that she's finally catching up with how she's growing" (32)*
Pain	7	Pain	4	*“It like hurts when you wake up in the morning.” (47)*
		Pain management	2	*Research priorities identified: Identifying effective interventions to prevent or reduce pain or stress (53)*
		Other outcomes only in 1 paper		Chronic pain
Other outcomes		Overall health state	2	*“We try to think of the whole life consequence. We talk about, you know, strength and cognitive capacity, but also life and communication and feeding yourself and getting around.’’ (46)*
		Vitality	2	*"The doctor said that, whatever we do, however good we are, it is mostly up to the infant himself. No matter how small they are, they can have something within them-selves." (54)*
		Physical appearance	7	*Both mothers and fathers found their infant's appearance and behaviour to be the stressors with the most impact (55)*
		Other outcomes only in 1 paper		Physiological stability

Confidential: For Review Only

Table 4 Parent focussed outcomes

Parent Focussed Outcome Domains	Number of studies discussing outcome domain *(n=62)*	Outcome	Number of studies discussing outcome *(n=62)*	Verbatim text extract
Parental support	30	Coping with maternal illness	5	*One nurse spoke of her belief that mothers could be diagnosed with depressive conditions (52)*
		Culture differences	2	*3 families felt strongly that their stress derived from differences in the medical management approaches between the United States and their homeland. (55)*
		Parental ability to work	2	*They liked being back at work because it helped occupy their minds, but they reported being exhausted (56)*
		Parental competence	4	*"We learned everything we needed and knew what we had to do, I was quite comfortable when we went home." (37)*
		Parental involvement	10	*"During our baby's stay in the neonatal unit both myself and Peter were fully involved in our son's care and did most of the caring such as nappy changing and NGT feeds" (41)*
		Support from family and friends	5	*"My mother in law and my mother both would watch my older daughter that first year quite a bit while I would take my daughter to therapy" (35)*
		Support from fathers	2	*Fathers ranged from being very supportive, "[we're] in this together," to being deterrents or completely absent. (56)*
		Support from healthcare professionals	6	*"The nursing staff, the doctors...they really know what they're doing...not only medically, but dealing with us personally...that helped a lot." (57)*
		Other outcomes only in 1 paper		Balancing caring for themselves and their baby: Barriers to parental involvement: Care provided close to home: Maintaining hope: Online support: Preparation for NICU admission: Support from faith
Other outcomes		Long term effects on parents	2	*We should be looking at Postnatal Depression after the baby goes home. . .Once they actually get a baby home, that's when the reality sets in. All the triggers are there. (52)*
		Other outcomes only in 1 paper		Support beyond NICU: Parental perception of uncertainty

Confidential: For Review Only

Table 5 Healthcare delivery outcomes

Healthcare Delivery Outcome Domains	Number of studies discussing outcome domain *(n=62)*	Outcome	Number of studies discussing outcome *(n=62)*	Verbatim text extract
Healthcare workers - Communication	30	Communicating in challenging settings	10	*When they arrived at hospital … some had difficult conversations with clinical staff … NICUs commonly set boundaries around the care that they offer (58)*
		Communicating information effectively	7	*Other parents experienced stress from unknown medical terminology(55)*
		Communication about discharge	3	*Parents/care givers may be inadequately prepared for home care and management of fragile neonates due to a lack of consistent and early communication (59)*
		Communication with parents	2	*"When you're talking to parents while you're doing cares and everything, you're not really talking to them,... you're having a vague conversation across the room" (60)*
		Developing a caring relationship	5	*As the providers gave support to families, families also were described as supporting the staff (45)*
		Keeping parents informed	7	*"I asked so many questions and read all the charts every day, and i probably angered them. Squeaky wheel gets the oil, as they say." (40)*
		Treating parents with respect	3	*"I got yelled at by a nurse at [the scn] for rubbing my son's foot [even though that was okay at the nicu]." (40)*
		Other outcomes only in 1 paper		Allowing time for conversation: Awareness of parental views: Candour: Communication with ex-neonatal patients: Language barrier: Using aids to communication
Healthcare workers – Knowledge and Competence	23	Consistency of decisions	6	*"Everybody had a different point of view but they were opinions, not facts. So that was huge, don't even get me started on that, that was just a nightmare." (50)*
		Ethical decision making	5	*". but when you actually mention this, say, "Well, in fact you know, we don't really know what's the best treatment," it is a delicate moment." (61)*
		Healthcare professionals behaviour	5	*"it wasn't a nurse related conversation it was just a casual conversation … Like I felt a bit (sic) she wasn't their priority." (50)*
		Healthcare professional competence	7	*Most of the parents recalled specific incidents that they perceived as poor medical care; typically, these incidents involved technical procedures or medical knowledge (62)*
		Identifying who is responsible for care	3	*"Sometimes we're not real clear who to follow up with." (40)*
		Staffing levels	2	*It was especially helpful for the parents to see so many nurses and physicians in the NICU.(63)*
		Other outcomes only in 1 paper		Expertise in palliative care: Medical errors: Staff insecurity
Other outcomes		Iatrogenic harm	3	*"there are potential toxicities, very real toxicities associated with it" (61)*
		Inclusion in research	2	*parents were often interested in the research, and some would have liked more contact and information than they actually received.(58)*

Confidential: For Review Only

Table 6 Economic outcomes

Economic Outcome Domains	Number of studies discussing outcome domain *(n=62)*	Outcome	Number of studies discussing outcome *(n=62)*	Verbatim text extract
Healthcare utilisation	15	Frequent appointments	2	*"I felt left out, I was always missing school because I had to go to the hospital for check-ups" (38)*
		Frequent readmissions	4	*The prolonged hospitalizations experienced by children with BPD and the frequent interactions of families with medical personnel may result in increased access and opportunities for services for parents of children with BPD. (64)*
		Inappropriate treatments	2	*Community providers…may lack the required knowledge and skills to manage complex infants, leading to suboptimal office-based care and perceived overutilisation of the emergency system.(59)*
		Need for frequent treatments	3	*"There were lots of masks and nebulisers during those years" (33)*
		Need for lifelong care	3	*"when the outcome is disastrous they just expect parents to take home severely handicapped babies and deal with life-long problems." (65)*
		Recurrent sickness	1	*"We've only put him with other children for the past month. The biggest worry right now is when he is going to get sick." (48)*
Other outcomes		Duration of admission	2	*Decrease length of stay selected as key performance indicator (49)*
		Healthcare resources	3	*Although respondents frequently discussed the emotional toll to all concerned, the monetary cost of long-term stays was very rarely (<1%) mentioned.(45)*

Confidential: For Review Only

Table 7 Social outcomes

Social Outcome Domains	Number of studies discussing outcome domain *(n=62)*	Outcome	Number of studies discussing outcome *(n=62)*	Verbatim text extract
Relationships with others	19	Bonding with family and friends	3	*"The only thing we might have done...some of our closest friends...it would have been nice to have them there as well" (57)*
		Bonding with parents	8	*"I find it a great joy when the mums do hold the baby against their chest" (66)*
		Effects on family and friends	7	*Almost all parents acknowledged the emotional adjustment of other family members in response to raising a child with physical impairment. (35)*
		Family resources	2	*3 families felt overwhelmed by a lack of resources (especially in the area of family support) (55)*
		Peer acceptance	2	*I've had four year olds tell me the other kids don't want to play with them cause they have a dumb arm.(47)*
		Other outcomes only in 1 paper		Childhood happiness: Overprotective parent-child relationship: Psychological coping
Psychiatric	7	Need for educational support	7	*The patient is at an age-appropriate grade level but attends resource classes in math and achieves only average grades in other areas. (39)*
		Psychiatric disorder	3	*The mother is very focused on the boys' physical and emotional symptoms (39)*
		Other outcomes only in 1 paper		Autism: Behavioural disturbances: Dyslexia: Mood disorders
Other outcomes		Other outcomes only in 1 paper		Schooling: Self-identifying as premature

Confidential: For Review Only

From these outcome domains the most frequently discussed at study level were "parental support" (30 studies, 48%) and "healthcare workers - communication" (30 studies, 48%). The individual outcomes most frequently discussed were "normality" (16 studies, 26%) and "survival" (11 studies, 18%).

Permutation test analysis showed a statistically significant association (p=0.037) between different stakeholder groups and outcome categories identified (eFigure 3). The frequency with which patients discussed the outcomes was most divergent from the other groups. In particular, patients discussed outcomes relating to the genitourinary, surgical, developmental and pain outcome domains more than would be expected by chance.
We found no statistically significant association (p=0.114) between gestational age and outcome categories (eFigure 2).

DISCUSSION

We have systematically reviewed and synthesised the outcomes reported in qualitative research by those with lived experience of neonatal care: patients, parents and healthcare professionals. We show that the patterns of outcomes discussed by former neonatal patients, parents and healthcare professionals are different. This is in keeping with previous single centre research (67) and case reports (68). This indicates that healthcare professionals should consider whether the outcomes they discuss align with patients' and parents' concerns (69). Acceptance of the differences shown should form part of the process of shared decision making in clinical practice (70). Poor outcome selection is also a known problem in paediatric research (8, 71), involving patients and parents will help to reduce research waste (72, 73).

Confidential: For Review Only

The outcomes identified extend beyond the organ system specific outcomes commonly reported in clinical trials and include global concepts such as “normality” of the child in later life, the impact on an infant’s family and the healthcare team, financial and time costs, and how patients interact with wider society. Our findings are in keeping with observational studies illustrating the wide reaching consequences of neonatal illness (74-76). Another feature of the outcomes identified is that rather than relating to a specific diagnosis or disease many reflect the global status of the child. Diagnoses like necrotising enterocolitis or retinopathy of prematurity were mentioned less frequently than their consequences, such as feeding difficulties or visual impairment. In general, the outcomes identified indicate that pathological processes and diagnoses are less relevant to patients and parents than the effects they have on day to day life. Priority should be given to identifying efficient ways of measuring more global outcomes of neonatal conditions throughout childhood and later life, for example through robust linkage of neonatal data with education databases.

This more holistic approach should extend to how babies are categorised. Our work included an undoubtedly heterogeneous population, but this was driven by discussions with former neonatal patients and parents at the planning stages of this project. They strongly stated that “a sick baby is a sick baby” regardless of birthweight or gestational age: a statement that is supported by our finding that there was no significant difference in how frequently outcomes were discussed in relation to babies of differing gestational ages. Splitting research populations by arbitrary landmarks not recognised by parents or former patients (68) may be a source of research heterogeneity.

The strengths of our study included identification and synthesis of outcomes from an international and methodologically diverse range of studies, relating to babies of all

Confidential: For Review Only

gestational ages, and a wide range of stakeholders. We included outcomes that stakeholders spontaneously identified. As a result, we were able to include data from a wider range and diversity of stakeholders than a primary research study could. We followed a pre-registered protocol with reporting in line with PRISMA guidelines (18). It has been argued that quality assessment is needed in 'mapping' reviews to aid in interpretation and uptake of findings (24), but the value of this approach is uncertain (21). The consultation phase of our core outcome set development work will provide the opportunity to critically reflect on the contribution of these findings to our understanding of what constitutes an 'important' outcome in neonatal research.

A limitation of our study is that, in line with many systematic reviews, we are synthesising data from studies that did not explicitly address the research question we are asking. This meant that we combined data about which outcomes parents, patients or healthcare professionals mentioned during research. As a result we described how frequently outcomes were discussed, rather than the importance assigned by groups to each outcome. Many outcomes were only discussed in a single study. We present them here to show the range and breadth of outcomes discussed, but cannot comment on whether they are more or less important than more frequently mentioned outcomes. Another limitation is that the researchers who undertook the primary qualitative research in the included studies will have influenced our review through their analysis; we reviewed data that was a step removed from the opinions of the stakeholders themselves. However, by following rigorous methodology and employing a comprehensive search strategy we have combined all available data to produce this mapping review.

Trying to measure all of the varied outcomes identified in this work in research would be impractical, if not impossible. This work supports the importance of identifying a core

Confidential: For Review Only

outcomes set, and highlights the importance of input from all stakeholder groups. In other fields core outcomes sets have successfully aligned patient and healthcare professional research priorities (72).

CONCLUSION

Parents, patients and clinicians report a wide range of neonatal care outcomes. Parents and patients focus on different outcomes than health professionals. Outcomes reported do not map to organ systems commonly addressed in clinical trials, many are global outcomes. We suggest that the views of former patients and parents should be taken into consideration by researchers and funding bodies.

Confidential: For Review Only

DECLARATIONS

CONFLICT OF INTEREST DISCLOSURE

The authors declare that they have no competing interests.

ACKNOWLEDGEMENTS

The authors are grateful to Louise Wann (West Middlesex University Hospital) for her contributions running the database searches.

AUTHOR CONTRIBUTIONS

JW and CG conceived this systematic review. This protocol was created by JW, GB and CG. Searches were performed by LW. All search results were reviewed by JW and assessed by the eligibility criteria above. Quality assurance was completed by CG. Coding and result synthesis was completed by JW, GB and CG. Statistical analysis was completed by NL. The first draft of the manuscript was written by JW, CG, GB, and NL; NM edited and reviewed the manuscript. It was approved by JW, CG, GB, SA, LW, NL, NM and the COIN Steering Group.

FUNDING AND SPONSORSHIP

This research is sponsored by Imperial College London and supported by an MRC Clinician Scientist Fellowship award to CG (MR/N008405/1) and salary support for JW from the Portland Hospital. Imperial College London, the MRC and the Portland Hospital had no involvement in the research, or this publication.

Confidential: For Review Only

WHAT IS ALREADY KNOWN ON THIS TOPIC

- Multiple outcomes can be measured in infants that receive neonatal care.
- It is not known which outcomes are considered important by former neonatal patients, parents and healthcare professionals, or whether these differ between groups.

WHAT THIS STUDY ADDS

- The predominant outcomes identified by parents, former patients and health professionals related to holistic concepts (such as “normality).
- Significant differences were identified in outcomes discussed by parents, patients and health professionals.
- Differences in neonatal outcomes prioritised by parents, patients and health professionals should be recognised when planning research.

Confidential: For Review Only

REFERENCES

1. Harrison W, Goodman D. Epidemiologic Trends in Neonatal Intensive Care, 2007-2012. JAMA pediatrics. 2015;169(9):855-62. Epub 2015/07/28.
2. Gluckman PD, Hanson MA, Cooper C, Thornburg KL. Effect of in utero and early-life conditions on adult health and disease. The New England journal of medicine. 2008;359(1):61-73. Epub 2008/07/04.
3. Doyle LW, Anderson PJ. Adult outcome of extremely preterm infants. Pediatrics. 2010;126(2):342-51. Epub 2010/08/04.
4. Roberts G, Cheong JL. Long-term growth and general health for the tiniest or most immature infants. Seminars in fetal & neonatal medicine. 2014;19(2):118-24. Epub 2013/12/03.
5. Saigal S, Day KL, Van Lieshout RJ, Schmidt LA, Morrison KM, Boyle MH. Health, Wealth, Social Integration, and Sexuality of Extremely Low-Birth-Weight Prematurely Born Adults in the Fourth Decade of Life. JAMA pediatrics. 2016;170(7):678-86. Epub 2016/05/24.
6. Boyle EM, Poulsen G, Field DJ, Kurinczuk JJ, Wolke D, Alfirevic Z, et al. Effects of gestational age at birth on health outcomes at 3 and 5 years of age: population based cohort study. British Medical Journal. 2012;344.
7. Williamson PR, Altman DG, Bagley H, Barnes KL, Blazeby JM, Brookes ST, et al. The COMET Handbook: version 1.0. Trials. 2017;18.
8. Sinha IP, Williamson PR, Smyth RL. Outcomes in clinical trials of inhaled corticosteroids for children with asthma are narrowly focussed on short term disease activity. PloS one. 2009;4(7):e6276. Epub 2009/07/18.
9. Heneghan C, Goldacre B, Mahtani KR. Why clinical trial outcomes fail to translate into benefits for patients. Trials. 2017;18(1):122. Epub 2017/03/16.
10. Goodare H, Smith R. The rights of patients in research. BMJ. 1995;310(6990):1277-8. Epub 1995/05/20.
11. Staley K. Exploring Impact: Public involvement in NHS, public health and social care research. Eastleigh: INVOLVE; 2009.
12. Sofaer S. Qualitative methods: what are they and why use them? Health services research. 1999;34(5 Pt 2):1101-18. Epub 1999/12/11.
13. POPPY Steering Group. Family-centred care in neonatal units. A summary of research results and recommendations from the POPPY project. London: NCT, 2009.
14. Kain VJ. An exploration of the grief experiences of neonatal nurses: A focus group study. Journal of Neonatal Nursing. 2013;19(2):80-8.
15. Webbe J, Brunton G, Ali S, Duffy JM, Modi N, Gale C. Developing, implementing and disseminating a core outcome set for neonatal medicine. BMJ Paediatrics Open. 2017;1(1).
16. Ali S, Webbe J, Duffy J, Modi N, Gale C, editors. Outcome reporting in trials involving preterm infants: a systematic review. COMET VI; 2016; Amsterdam.
17. PROSPERO database. [13/02/2017]; Available from: http://www.crd.york.ac.uk/prospero/display_record.asp?ID=CRD42016037874. .
18. Moher D, Liberati A, Tetzlaff J, Altman DG. Preferred reporting items for systematic reviews and meta-analyses: the PRISMA statement. Annals of internal medicine. 2009;151(4):264-9, W64. Epub 2009/07/23.
19. Landis JR, Koch GG. The measurement of observer agreement for categorical data. Biometrics. 1977;33(1):159-74. Epub 1977/03/01.
20. Thomas J, Brunton J, Graziosi S. EPPI-Reviewer 4: software for research synthesis. EPPI-Centre Software. London: Social Science Research Unit, Institute of Education, University of London; 2010.
21. Collier D, Mahoney J. Insights and pitfalls - Selection bias in qualitative research. World Politics. 1996;49(1):56-+.

Confidential: For Review Only

22. NHS Digital. National Neonatal Data Set. 2017 [12/07/2017]; Available from: http://www.datadictionary.nhs.uk/data_dictionary/messages/clinical_data_sets/data_sets/national_neonatal_data_set/national_neonatal_data_set_-_episodic_and_daily_care_fr.asp?shownav=1.
23. Arksey H, O'Malley L. Scoping studies: towards a methodological framework. International Journal of Social Research Methodology. 2007;8(1):19-32.
24. Levac D, Colquhoun H, O'Brien KK. Scoping studies: advancing the methodology. Implement Sci. 2010;5(1):69.
25. Thomas J, Harden A, Newman M. Synthesis: combining results systematically and appropriately. In: Gough D, Oliver S, Thomas J, editors. An introduction to systematic reviews 2nd ed. London: Sage; 2017. p. 178-226.
26. Bakas T, McLennon SM, Carpenter JS, Buelow JM, Otte JL, Hanna KM, et al. Systematic review of health-related quality of life models. Health Qual Life Out. 2012;10.
27. Porter ME. What Is Value in Health Care? New England Journal of Medicine. 2010;363(26):2477-81.
28. Carpenter WR, Meyer AM, Abernethy AP, Sturmer T, Kosorok MR. A framework for understanding cancer comparative effectiveness research data needs. J Clin Epidemiol. 2012;65(11):1150-8.
29. Dimes Mo, PMNCH, Children St, WHO. Born Too Soon: The Global Action Report on Preterm Birth. Geneva: World Health Organization, 2012.
30. Ludbrook J. ADVANTAGES OF PERMUTATION (RANDOMIZATION) TESTS IN CLINICAL AND EXPERIMENTAL PHARMACOLOGY AND PHYSIOLOGY. Clinical and Experimental Pharmacology and Physiology. 1994;21(9):673-86.
31. Docherty SL, Lowry C, Miles MS. Poverty as context for the parenting experience of low-income Lumbee Indian mothers with a medically fragile infant. Neonatal network : NN. 2007;26(6):361-9. Epub 2007/12/12.
32. Hussey-Gardner BT, Wachtel RC, Viscardi RM. Parent perceptions of an NICU follow-up clinic. Neonatal network : NN. 1998;17(1):33-9. Epub 1998/04/04.
33. Lou H, Pedersen BD, Hedegaard M. Questions never asked. Positive family outcomes of extremely premature childbirth. Quality of life research : an international journal of quality of life aspects of treatment, care and rehabilitation. 2009;18(5):567-73. Epub 2009/05/01.
34. Arruda DC, Marcon SS. [Family experience of dealing with sequels of the prematurity of a child]. Revista brasileira de enfermagem. 2010;63(4):595-602. Epub 2010/09/14. Experiencia da familia ao conviver com sequelas decorrentes da prematuridade do filho.
35. Squitieri L, Larson BP, Chang KW, Yang LJ, Chung KC. Understanding quality of life and patient expectations among adolescents with neonatal brachial plexus palsy: a qualitative and quantitative pilot study. The Journal of hand surgery. 2013;38(12):2387-97 e2. Epub 2014/01/15.
36. Orfali K. Parental role in medical decision-making: fact or fiction? A comparative study of ethical dilemmas in French and American neonatal intensive care units. Soc Sci Med. 2004;58(10):2009-22. Epub 2004/03/17.
37. Brodsgaard A, Zimmermann R, Petersen M. A preterm lifeline: Early discharge programme based on family-centred care. Journal for specialists in pediatric nursing : JSPN. 2015;20(4):232-43. Epub 2015/07/22.
38. Leavy P, Violeta Prina M, Martinez Caceres MJ, Bauer G. Social representations of premature birth from the perspective of individuals born preterm in the 1990s. Archivos argentinos de pediatria. 2015;113(1):6-11. Epub 2015/01/27.
39. Votteler TP, Lipsky K. Long-term results of 10 conjoined twin separations. Journal of pediatric surgery. 2005;40(4):618-29. Epub 2005/04/27.
40. Hanrahan K, Gates M, Attar MA, Lang SW, Frohna A, Clark SJ. Neonatal back transport: perspectives from parents of Medicaid-insured infants and providers. Neonatal network : NN. 2007;26(5):301-11. Epub 2007/10/12.
41. Cescutti-Butler L. Eliciting parental views regarding early discharge to home care for premature infants. Infant. 2009;5(1):23-8.

Confidential: For Review Only

42. Reyna BA, Pickler RH, Thompson A. A descriptive study of mothers' experiences feeding their preterm infants after discharge. Advances in neonatal care : official journal of the National Association of Neonatal Nurses. 2006;6(6):333-40. Epub 2007/01/09.
43. Malhotra Y, Nzegwu N, Harrington J, Ehrenkranz RA, Hafler JP. Identifying Barriers to Initiating Minimal Enteral Feedings in Very Low-Birth-Weight Infants: A Mixed Methods Approach. Am J Perinat. 2016;33(1):47-56.
44. Stevens EE, Gazza E, Pickler R. Parental Experience Learning to Feed Their Preterm Infants. Adv Neonat Care. 2014;14(5):354-61.
45. Catlin A. Extremely long hospitalizations of newborns in the United States: data, descriptions, dilemmas. Advances in neonatal care : official journal of the National Association of Neonatal Nurses. 2008;8(2):125-32. Epub 2008/04/18.
46. Rasmussen LA, Bell E, Racine E. A Qualitative Study of Physician Perspectives on Prognostication in Neonatal Hypoxic Ischemic Encephalopathy. Journal of child neurology. 2016;31(11):1312-9. Epub 2016/07/06.
47. Chang KWC, Austin A, Yeaman J, Phillips L, Kratz A, Yang LJS, et al. Health-Related Quality of Life Components in Children With Neonatal Brachial Plexus Palsy: A Qualitative Study. Pm&R. 2017;9(4):383-91.
48. Holditch-Davis D, Bartlett TR, Blickman AL, Miles MS. Posttraumatic stress symptoms in mothers of premature infants. Journal of obstetric, gynecologic, and neonatal nursing : JOGNN. 2003;32(2):161-71. Epub 2003/04/11.
49. Rohsiswatmo R, Rafika S, Marsubrin PM. Prevention and control of blood stream infection using the balanced scorecard approach. Acta medica Indonesiana. 2014;46(3):209-16. Epub 2014/10/29.
50. Jones L, Taylor T, Watson B, Fenwick J, Dordic T. Negotiating Care in the Special Care Nursery: Parents' and Nurses' Perceptions of Nurse-Parent Communication. Journal of pediatric nursing. 2015;30(6):e71-80. Epub 2015/05/03.
51. Brinchmann BS, Nortvedt P. Ethical decision making in neonatal units--the normative significance of vitality. Medicine, health care, and philosophy. 2001;4(2):193-200. Epub 2001/09/08.
52. Green J, Darbyshire P, Adams A, Jackson D. Desperately seeking parenthood: neonatal nurses reflect on parental anguish. Journal of clinical nursing. 2015;24(13-14):1885-94. Epub 2015/05/06.
53. Wielenga JM, Tume LN, Latour JM, van den Hoogen A. European neonatal intensive care nursing research priorities: an e-Delphi study. Archives of disease in childhood Fetal and neonatal edition. 2015;100(1):F66-71. Epub 2014/09/28.
54. Brinchmann BS. 'They have to show that they can make it': vitality as a criterion for the prognosis of premature infants. Nursing ethics. 2000;7(2):141-7. Epub 2000/09/15.
55. Lee SY, Lee KA, Rankin SH, Alkon A, Weiss SJ. Acculturation and stress in Chinese-American parents of infants cared for in the intensive care unit. Advances in neonatal care : official journal of the National Association of Neonatal Nurses. 2005;5(6):315-28. Epub 2005/12/13.
56. Sisk P, Quandt S, Parson N, Tucker J. Breast Milk Expression and Maintenance in Mothers of Very Low Birth Weight Infants: Supports and Barriers. J Hum Lact. 2010;26(4):368-75.
57. Armentrout D. Living with grief following removal of infant life support: parents' perspectives. Critical care nursing clinics of North America. 2009;21(2):253-65. Epub 2009/05/23.
58. Snowdon C, Brocklehurst P, Tasker R, Platt MW, Harvey S, Elbourne D. Death, Bereavement and randomised controlled trials (BRACELET): a methodological study of policy and practice in neonatal and paediatric intensive care trials. Health Technol Asses. 2014;18(42):1-+.
59. Moyer VA, Singh H, Finkel KL, Giardino AP. Transitions from neonatal intensive care unit to ambulatory care: description and evaluation of the proactive risk assessment process. Qual Saf Health Care. 2010;19:I26-I30.
60. Turrill S. A focus of care for neonatal nursing: the relationship between neonatal nursing practice and outcomes. Part 2. Paediatric Nursing. 2003;15(5):30.

Confidential: For Review Only

61. Garcia J, Elbourne D, Snowdon C. Equipoise: a case study of the views of clinicians involved in two neonatal trials. Clinical trials (London, England). 2004;1(2):170.
62. Kavanaugh K. Parents' experience surrounding the death of a newborn whose birth is at the margin of viability. Journal of obstetric, gynecologic, and neonatal nursing : JOGNN. 1997;26(1):43-51. Epub 1997/01/01.
63. Griffin T, Kavanaugh K, Soto CF, White M. Parental evaluation of a tour of the neonatal intensive care unit during a high-risk pregnancy. Journal of obstetric, gynecologic, and neonatal nursing : JOGNN. 1997;26(1):59-65. Epub 1997/01/01.
64. Short EJ, Klein NK, Lewis BA, Fulton S, Eisengart S, Kercsmar C, et al. Cognitive and academic consequences of bronchopulmonary dysplasia and very low birth weight: 8-year-old outcomes. Pediatrics. 2003;112(5):e359. Epub 2003/11/05.
65. Hefferman P, Heilig S. Giving "moral distress" a voice: ethical concerns among neonatal intensive care unit personnel. Cambridge quarterly of healthcare ethics : CQ : the international journal of healthcare ethics committees. 1999;8(2):173-8. Epub 1999/03/06.
66. Chia P, Sellick K, Gan S. The attitudes and practices of neonatal nurses in the use of kangaroo care. Aust J Adv Nurs. 2006;23(4):20-7.
67. Janvier A, Farlow B, Baardsnes J, Pearce R, Barrington KJ. Measuring and communicating meaningful outcomes in neonatology: A family perspective. Seminars in perinatology. 2016;40(8):571-7. Epub 2016/10/30.
68. Staub K, Baardsnes J, Hébert N, Hébert M, Newell S, Pearce R. Our child is not just a gestational age. A first-hand account of what parents want and need to know before premature birth. Acta Paediatrica. 2014;103:1035-8.
69. Pearce R, Baardsnes J. Term MRI for small preterm babies: do parents really want to know and why has nobody asked them? Acta Paediatrica. 2012;101(10):1013-5.
70. Charles C, Gafni A, Whelan T. Shared decision-making in the medical encounter: What does it mean? (Or it takes at least two to tango). Social Science & Medicine. 1997;44(5):681-92.
71. Gardner W, Kelleher KJ. Core Quality and Outcome Measures for Pediatric Health. JAMA pediatrics. 2017;171(9):827-8.
72. de Wit M, Abma T, Koelewijn-van Loon M, Collins S, Kirwan J. Involving patient research partners has a significant impact on outcomes research: a responsive evaluation of the international OMERACT conferences. BMJ open. 2013;3(5).
73. INVOLVE. Public involvement in research: values and principles framework. Eastleigh: INVOLVE, 2015.
74. Saigal S, Doyle LW. An overview of mortality and sequelae of preterm birth from infancy to adulthood. Lancet. 2008;371(9608):261-9. Epub 2008/01/22.
75. Muller-Nix C, Forcada-Guex M, Pierrehumbert B, Jaunin L, Borghini A, Ansermet F. Prematurity, maternal stress and mother-child interactions. Early Hum Dev. 2004;79(2):145-58.
76. Hall EO. A double concern: grandmothers' experiences when a small grandchild is critically ill. Journal of pediatric nursing. 2004;19(1):61-9. Epub 2004/02/14.

Confidential: For Review Only

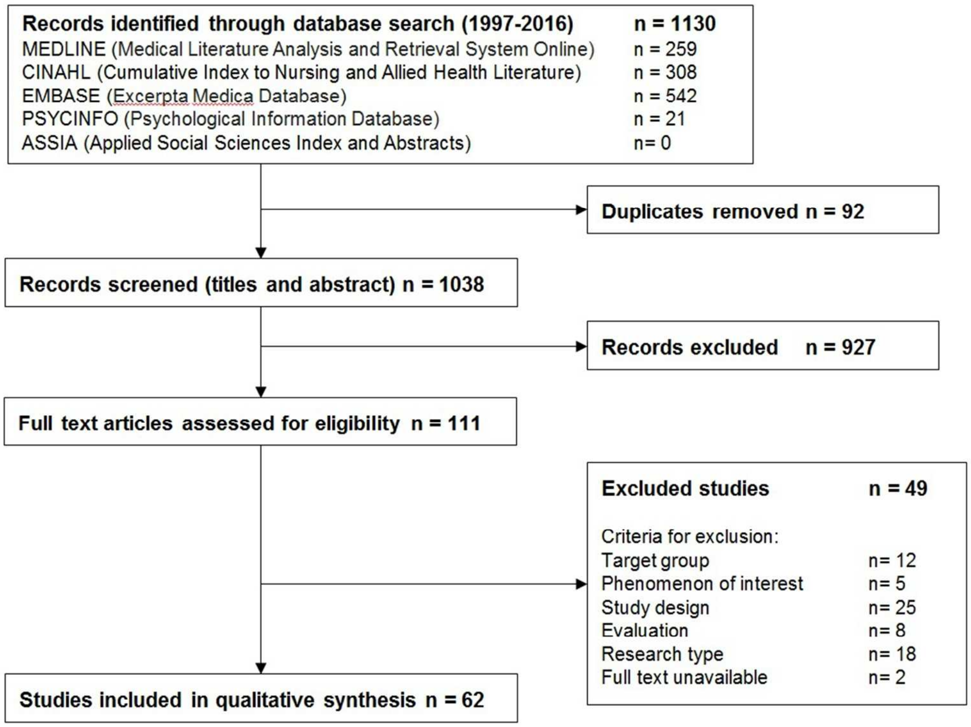

276x206mm (96 x 96 DPI)

review Only

Online only supplemental material

eTable 1: Eligiblity criteria
eFigure 1: Search strategy
eTable 2: Summary of included studies
eTable 3: List of all outcomes
eTable 4: Outcomes within each study
eFigure 2: Results of permutation test analysis regarding gestational age
eFigure 3: Results of permutation test analysis regarding stakeholder group

Confidential: For Review Only

eTable 1: Eligibility Criteria

Sample: Babies of any gestational age at birth receiving care in a neonatal unit.

Phenomenon of Interest: Care on a Neonatal Intensive Care Unit or Special Care Baby Unit. Care provided exclusively on labour or postnatal wards or in an outpatient/community environment was not included.

Design: Qualitative research (including phenomenology, ethnography, case studies and grounded theory) was included. Quantitative research was included if qualitative data had been gathered (e.g. surveys developed with parent or caregiver input).

Evaluation: Data relating to outcomes that former patients, parents or healthcare professionals feel are important.

Research type: Qualitative research or mixed methods research was included. Research that was exclusively quantitative was excluded.

Confidential: For Review Only

eFigure 1: Search Strategy

The following search strategy was utilised to search the Medline database:

1. Medline; neonat*.ti,ab;
2. Medline; exp INFANT, NEWBORN/;
3. Medline; ((preterm OR "pre term" OR prematur*) AND (infan* OR neonat* OR baby OR babies)).ti,ab;
4. Medline; (infant* adj2 premature).af;
5. Medline; exp NEONATOLOGY/;
6. Medline; exp INTENSIVE CARE UNITS, NEONATAL/;
7. Medline; exp INTENSIVE CARE, NEONATAL/;
8. Medline; ("intensive care" adj2 neonat*).ti,ab;
9. Medline; NICU.ti,ab;
10. Medline; SCBU.ti,ab;
11. Medline; "special care baby unit*".ti,ab;
12. Medline; INFANT, PREMATURE, DISEASES/;
13. Medline; 1 OR 2 OR 3 OR 4 OR 5 OR 6 OR 7 OR 8 OR 9 OR 10 OR 11 OR 12;
14. Medline; (treatment adj2 outcome*).ti,ab;
15. Medline; exp TREATMENT OUTCOME/;
16. Medline; "key outcome*".ti,ab;
17. Medline; (important adj2 outcome*).ti,ab;
18. Medline; exp PATIENT OUTCOME ASSESSMENT/;
19. Medline; (outcome* adj2 set).ti,ab;
20. Medline; exp "OUTCOME ASSESSMENT (HEALTH CARE)"/;
21. Medline; "outcome assessment*".ti,ab;
22. Medline; (core adj2 outcome*).ti,ab;
23. Medline; "outcome measure*".ti,ab;
24. Medline; 14 OR 15 OR 16 OR 17 OR 18 OR 19 OR 20 OR 21 OR 22 OR 23;
25. Medline; ("semi-structured" OR semistructured OR unstructured OR informal OR "indepth" OR indepth OR "face-to-face").ti,ab;
26. Medline; ((structured OR guide) adj3 (interview* OR discussion*)).ti,ab;
27. Medline; ("focus group*" OR qualitative OR ethnograph* OR fieldwork OR "field work" OR "key informant*").ti,ab;
28. Medline; exp INTERVIEWS AS TOPIC/;
29. Medline; exp FOCUS GROUPS/;
30. Medline; exp NARRATION/;
31. Medline; exp QUALITATIVE RESEARCH/;
32. Medline; 25 O 26 OR 27 OR 28 OR 29 OR 30 OR 31 OR 32;
33. Medline; 13 AND 24 AND 32;

Confidential: For Review Only

eTable 2: Summary Of Included Studies

Source	Country	Gestational age of neonates involved	Birthweight of neonates involved	Methodology	Stakeholders (numbers and further details given in brackets where available)
Ahern (2013)	Australia	Unspecified	Unspecified	Questionnaire (Delphi); Interviews (individual)	Doctors; Nurses; Others (social worker, clinical educator)
Armentrout (2009)	USA	Term; Preterm; Extreme preterm	Not specified	Interviews (individual); Interviews (group)	Parents (11 mothers, 4 fathers)
Arruda et al. (2010)	Brazil	Preterm; Extreme preterm	Very low	Interviews (individual); Interviews (group	Parents (8 mothers); Other family members (1 maternal grandmother)
Brinchmann (2000)	Norway	Preterm; Extreme preterm	Unspecified	Interviews (unspecified size); Direct observation	Doctors (8); Nurses (10);
Brinchmann et al. (2001)	Norway	Unspecified	Unspecified	Interviews (unspecified size); Direct observation	Doctors (8); Nurses (10); Parents (20)
Brødsgaard (2015)	Denmark	Preterm; Extreme preterm	Normal; Low; Very low; Extremely low	Questionnaire; Interviews (group)	Parents (15)
Callen et al. (2005)	Canada	Preterm; Extreme preterm	Very low; Extremely low	Direct observation	Parents (64 mothers)
Catlin (2008)	USA	Term; Extreme preterm	Normal; Low; Very low; Extremely low	Questionnaire	Doctors (42); Nurses (291); Others (4 social workers and 76 unspecified)
Cescutti-Butler (2009)	UK	Unspecified	Unspecified	Interviews (unspecified size)	Parents (4 mothers, 1 father)
Chang (2014)	USA	Unspecified	Unspecified	Interviews (group)	Doctors; Nurses; Other allied healthcare professionals (occupational therapist); Former neonatal patients (13); Parents (23 mothers, 5 fathers); Other family members (1 grandparent, 2 unspecified); Others (social worker, clinical co-ordinator)
Chia (2006)	Australia	Unspecified	Unspecified	Questionnaire; Interviews (unspecified size)	Nurses (34)
Chollat (2014)	France	Preterm; Extreme preterm	Low; Very low	Questionnaire	Parents (431)
Darcy (2008)	USA	Unspecified	Unspecified	Interviews (unspecified size)	Nurses (6)
Docherty (2007)	USA	Term; Preterm	Unspecified	Interviews (individual)	Parents (5 mothers)
Ericson (2013)	Sweden	Preterm; Extreme preterm	Low	Questionnaire	Nurses (45)
Gaíva (2001)	Brazil	Preterm	Unspecified	Questionnaire	Parents (11 mothers)
Garcia (2004)	UK	Term; Preterm	Unspecified	Interview (individual)	Doctors (30)
Gontijo (2008)	Brazil	Preterm	Low	Questionnaire; Direct observation	Other allied healthcare professionals (19 unspecified); Parents (13 mothers);
Green (2015)	Australia	Extreme preterm	Unspecified	Questionnaire; Interviews (individual); Interviews (group)	Nurses (414)
Gregory (2012)	USA and Canada	Preterm	Unspecified	Questionnaire	Nurses (70)
Griffin (1997)	USA	Preterm; Extreme preterm	Unspecified	Interviews (unspecified size)	Parents (6 mothers, 2 fathers); Others (5 parents of infants not admitted to NICU)
Hanrahan (2007)	USA	Unspecified	Unspecified	Interviews (group)	Doctors (12); Nurses (11); Parents (10 mothers, 2 fathers)
Hefferman (1999)	USA	Extreme Preterm	Unspecified	Questionnaire	Doctors (3); Nurses (27); Other allied healthcare professionals (2 nurse managers, 1 respiratory care supervisor)
Hentschel (2006)	Germany	Term; Preterm	Unspecified	Direct observation	Doctors; Nurses; Parents (31)
Holditch-Davis (2003)	USA	Preterm	Normal; Low; Very low	Interviews (unspecified size)	Parents (30 mothers)
Hussey-Gardner (1998)	USA	Preterm	Very low	Interviews (unspecified size); Direct observation; Other (artefact collection)	Parents (7 mothers, 4 fathers); Other family members (3 grandmothers)
Jirapaet (2006)	Thailand	Unspecified	Unspecified	Interviews (individual)	Nurses (27)
Johnson (2007)	USA	Preterm	Very low	Interviews (unspecified size)	Parents (18 mothers)
Jones (2015)	Australia	Preterm; Extreme preterm	Normal; Low; Very low; Extremely low	Interviews (individual); Interviews (group)	Nurses (12); Parents (27 mothers, 5 fathers)
Kavanaugh (1997)	USA	Unspecified	Extremely low	Interviews (individual)	Parents (5 mothers, 3 fathers)
Koh (2007)	Australia	Preterm	Low	Questionnaire; Interviews (individual)	Parents (200 mothers)
Leavy (2015)	Argentina	Preterm	Very low	Interviews (group)	Former neonatal patients (59)
Lee (2005)	USA	Preterm	Unspecified	Questionnaire; Interviews (unspecified)	Parents (30 mothers, 25 fathers)
Lou (2009)	Denmark	Extreme preterm	Very low	Interviews (individual)	Parents (11 mothers, 9 fathers)
Malhotra (2015)	USA	Unspecified	Very low	Interviews (unspecified size); Direct observation	Doctors (10); Nurses (16)
Miljeteig (2006)	India	Term; Preterm	Unspecified	Interviews (individual); Direct observation	Doctors (14); Nurses (8)
Miracle (2004)	USA	Preterm; Extreme preterm	Very low; Extremely low	Interviews (individual)	Parents (21 mothers)
Moyer (2010)	USA	Unspecified	Unspecified	Interviews (unspecified size); Direct observation; Other (Health care failure mode and effect analysis)	Doctors; Nurses; Parent (1); Others (discharge planners, social worker)
Orfali (2004)	USA	Preterm; Extreme preterm	Low; Very low	Interviews; Direct observation	Doctors (60); Parents (71)
Rasmussen (2016)	Canada	Unspecified	Unspecified	Interviews (individual)	Doctors (12)
Reyna (2006)	USA	Preterm	Unspecified	Interview (size unspecified)	Parents (27 mothers)

Confidential: For Review Only

Rohsiswatmo (2014)	Indonesia	Unspecified	Low; Very low	Questionnaire; Interviews (group); Observation; Other (operational research)	Other allied healthcare professionals (unspecif
Saigal (1999)	Canada	Unspecified	Normal; Extremely low	Questionnaire; Interviews (individual)	Doctors (100); Nurses (103); Former neonatal (264); Parents (275)
Saunders (2003)	USA	Unspecified	Very low	Interviews (group); Other (Internal process analysis, Collaborative learning)	Doctors; Nurses; Other allied healthcare profes (respiratory therapists, pharmacists, physical a occupational therapists) Parents (76); Others (secretaries, social workers)
Sawyer (2014)	UK	Preterm	Unspecified	Questionnaire; Interviews (individual); Interviews (group)	Parents (145 mothers, 85 fathers)
Schneiderman (1997)	USA	Unspecified	Unspecified	Interviews (individual)	Other allied healthcare professionals (22 Emer department directors, 43 NICU directors, 43 Et committee chairpeople)
Sharek (2003)	USA	Unspecified	Low; Very low; Extremely low	Interviews (group)	Other allied healthcare professionals (unspecif
Short (2003)	USA	Term; Preterm; Extreme preterm	Normal; Very low	Questionnaire; Interviews (unspecified size)	Parents; Other (teachers)
Sisk (2010)	USA	Preterm; Extreme preterm	Low; Very low; Extremely low	Interviews (individual)	Parents (32 mothers)
Smith (2006)	USA	Preterm; Extreme preterm	Unspecified	Interviews (individual)	Parents (16 fathers)
Snowdon (1997)	UK	Term	Unspecified	Interviews (unspecified size)	Parents (21 mothers, 16 fathers)
Snowdon (2014)	UK	Term; Preterm; Extreme preterm	Normal; Low; Very low; Extremely low	Interviews (individual)	Other allied healthcare professionals (51 trial te members); Parents (51)
Squitieri (2013)	USA	Unspecified	Unspecified	Questionnaire; Interviews (individual)	Former neonatal patients (18); Parents (18)
Stevens (2014)	USA	Preterm; Extreme preterm	Low; Very low; Extremely low	Interviews (individual)	Parents (12 mothers, 8 fathers)
Thoren (2013)	Germany	Preterm	Unspecified	Other (Evaluation of Facebook groups and discussion content)	Parents; Other (representatives of online group
Thoyre (2000)	USA	Unspecified	Very low	Interviews (individual)	Parents (22 mothers)
Turrill (2003)	UK	Unspecified	Unspecified	Interviews (unspecified size)	Nurses (6); Other allied healthcare professiona clinical services manager)
Turrill (2003)	UK	Unspecified	Unspecified	Interviews (unspecified size); Direct observation	Nurses
van Zuuren (2006)	Netherlands	Unspecified	Unspecified	Interviews (individual); Direct observation	Doctors (5); Nurses (6); Others (1 social worke pastoral care worker)
Vandenbussche (1999)	Netherlands	Unspecified	Unspecified	Questionnaire	Doctors (12); Parents (30 mothers)
Votteler (2005)	USA	Unspecified	Unspecified	Questionnaire; Interviews (individual)	Former neonatal patients (14); Parents; Other f members
Wielenga (2015)	Netherlands	Unspecified	Unspecified	Questionnaire (Delphi)	Nurses; Other allied healthcare professionals (

Confidential: For Review Only

eTable 3: Outcomes Identified

Outcome Domain Category	Outcome Domain	Outcome
Organ system	Respiratory	Asthma
		Breathlessness
		Chronic Lung Disease
		Excessive secretions
		Frequent respiratory illnesses
		Mechanical ventilation
		Nasal congestion
		Oxygen dependence
		Pneumothorax
	Cardiovascular	Hypotension
		Presence of Patent Ductus Arteriosus
	Gastrointestinal	Breastfeeding
		Choice of milk for feeding
		Choking during feeding
		Eating disorder
		Feeding difficulties
		Feeding practices
		Fistulas
		Frequency of defaecation
		Initiating enteral feeds
		Liver failure
		Necrotising enterocolitis
		Nutritional intake
		Oral feeding
		Other gastrointestinal malformations
		Regurgitation
		Short gut syndrome
	Neurological	Brain damage (not further specified)
		Neurological care
		Neurological symptoms
		Seizures
		Significant IVH
		Sleep disorders
	Genitourinary	Urological disorders
	Infection	Prevention of infection
		Sepsis
		Susceptibility to infection
	Skin	Appearance of scars
		Burns
		Extravasation injuries

Confidential: For Review Only

		Pressure sores
		Skin care
	Surgical	Appearance of scars
		Care for surgical babies
		Need for ileostomy
		Need for multiple operations
	Developmental	Ability to feed themselves
		Ability to undertake sport
		Ability to walk
		Difficulties with activities of daily living
		Hearing impairment
		Issues of development and motor skills
		Language disorders
		Need for physical therapy
		Normal hearing
		Retinopathy of prematurity
		Social difficulties
		Visual impairment
Holistic	Normality	Ability to lead a normal life
		Being treated normally
		Inability to create a normal life
		Normal health
		Normality
		Thriving
	Suffering	Comfort
		Ex-patients separation from their suffering
		Symptom control
		Suffering
	Survival	Survival
		Survival with disability
		Survival without disability
	Pain	Chronic pain
		Pain
		Pain management
	Growth	Growth
	Other holistic outcomes	Overall Health State
		Physical appearance
		Physiological stability
		Vitality
Parent focussed	Parental support	Balancing caring for themselves and their baby
		Barriers to parental involvement
		Care provided close to home
		Coping with maternal illness
		Culture differences

Confidential: For Review Only

		Maintaining hope
		Online support
		Parental ability to work
		Parental competence
		Parental involvement
		Preparation for NICU admission
		Support from faith
		Support from family and friends
		Support from fathers
		Support from healthcare professionals
	Other parent focussed outcomes	Long term effects on parents
		Parental perception of uncertainty
		Support beyond NICU
Healthcare delivery	Healthcare workers-Communication	Allowing time for conversation
		Awareness of parental views
		Candour
		Communicating in challenging settings
		Communicating information effectively
		Communication about discharge
		Communication with ex-neonatal patients
		Communication with parents
		Developing a caring relationship
		Keeping parents informed
		Language barrier
		Treating parents with respect
		Using aids to communication
	Healthcare workers-Knowledge and Competence	Consistency of decisions
		Ethical decision making
		Expertise in palliative care
		Healthcare professional competence
		Healthcare professionals behaviour
		Identifying who is responsible for care
		Medical errors
		Staff insecurity
		Staffing levels
		Iatrogenic harm
	Other healthcare delivery outcomes	Inclusion in research
Economic	Healthcare utilisation	Frequent appointments
		Frequent readmissions
		Inappropriate treatments
		Need for frequent treatments
		Need for lifelong care
		Recurrent sickness
	Other economic outcomes	Duration of admission

Confidential: For Review Only

		Healthcare resources
Social	Psychiatric	Autism
		Behavioural disturbances
		Dyslexia
		Mood disorders
		Need for educational support
		Psychiatric disorder
	Relationships with others	Bonding with family and friends
		Bonding with parents
		Childhood happiness
		Effects on family and friends
		Family resources
		Overprotective parent-child relationship
		Peer acceptance
		Psychological coping
	Other social outcomes	Schooling
		Self-identifying as premature

Confidential: For Review Only

eTable 4: Outcomes Within Each Study

Source	Outcome Domains and outcomes
Ahern (2013)	Respiratory<br>Breathlessness: *Education priorities: Managing symptoms (eg, secretions, breathlessness) in a dying baby*<br>Excessive secretions: *Education priorities: Managing symptoms (eg, secretions, breathlessness) in a dying baby*<br>Gastrointestinal<br>Feeding Difficulties: *Education priorities: Managing feeding in a dying baby*<br>Parental Support<br>Support from Healthcare Professionals: *Educational priorities identified included: How to provide emotional support to grieving parents Helping parents say "goodbye" Supporting parents when they are at different stages of grief Identifying and supporting families at high risk of complicated grief*<br>Healthcare workers-Communication<br>Communicating in challenging settings: *Education priorities: Communicating with parents of palliative neonates Educational priorities identified included: Knowing what to say; using the right words Communicating with parents about the palliative care plan Initiating difficult conversations*<br>Pain<br>Pain: *Educational priorities identified included: Assessing and managing pain in a dying baby*<br>Suffering<br>Symptom control: *Managing symptoms Educational priorities identified included: Managing symptoms (eg, secretions, breathlessness) in a dying baby*
Armentrout<br><br>(2009)	Development-Special Senses<br>Normal hearing:*"She got to hear her dad sing,"*<br>Parental Support<br>Support from healthcare professionals:*"the nursing staff, the doctors...they really know what they're doing...not only medically, but dealing with us personally...that helped a lot."*<br>Healthcare workers-Communication<br>Developing a caring relationship: *"such a great blessing...that there was no hindrance in the terms of any kind of an attitude or anything with the nurse or the attending doctor that made us feel uncomfort-able or gave us any additional grief."*<br>*"the nursing staff, the doctors they really know what they're doing not only medically, but dealing with us personally that helped a lot."*<br>Healthcare workers-Knowledge and Competence<br>Healthcare professional competence: *"the nursing staff, the doctors they really know what they're doing not only medically, but dealing with us personally that helped a lot."*<br>Relationships with others<br>Bonding with parents: *"She got to hear her dad sing, she got to...she knew we were holding her"*<br>*"my feeling is, if your child has to die, what better place than in mom's arms"*<br>Bonding with family and friends: *"The only thing we might have done...some of our closest friends...it would have been nice to have them there as well...none of our friends got to see or meet him while he was alive."*

Confidential: For Review Only

	"I wanted them to spend time with her, hold her, and bathe her and do whatever" being able to have their infant with them as part of a family was something that all parents proclaimed they "would never replace for anything in the world."<br>Effects on family and friends: *"I guess I feel like I lost about a year of my son's time. He turned 3 shortly after my daughter died, and it wasn't until he was a little after 4 that I turned around in the kitchen and saw this little boy and said "Who are you?" I wasn't all there for about a year and this little boy, even though he was the one who got me through it I really didn't know who he was."*<br>Suffering<br>Suffering: *"He was just suffering, and we didn't want him to suffer." "not withdrawing support would be... "some-thing to satisfy my empty arms."*<br>Normality<br>Normality: *"Personally my only regret was that I didn't bathe him" "And the baptism...why didn't we do it sooner...why did we wait for somebody on call to show up with a bottle of sterile water and a cotton ball...why didn't we do it the right way?"*<br>*"I think it's important to allow yourself to be a mother before you let go...of the baby...to be a parent to some degree...to change their diaper...feed 'em if you can...play with their toes and fingers and nose and...just be a mother to 'em...be a parent to 'em...before you turn it off, because that...is something that you will cherish for the rest of your life."*<br>*For some it was their only opportunity to hold and talk to their infant like "a normal baby without all the tubes and wires."*<br>Other<br>Support beyond NICU: *"the medical professionals who have become your support system and your family while your child was alive...all of a sudden they're gone...I think it's important to have some kind of support system or information...otherwise it's like stepping into this huge abyss."*<br>Long term effects on parents: *"would get up in the morning I would be normal after about 2 weeks, 3 weeks act normally take the little one to school, stay in the car for a while come back home and get into bed for the next 6 hours and cry,"*<br>*"The milestone of a year was really bad. And it is still bad. I mean I'm functioning. I put on make-up. I get dressed. I go out. I look normal but it lasts for a long time the bad thing."*
Arruda et al.<br><br>(2010)	Respiratory<br>Frequent respiratory illnesses: *all concerned and involved with numerous hospitalizations resulting from respiratory failure "He had a lot of pneumonia up to two years, was direct in hospital"* Need for supplemental oxygen: *"Even the three years he was in direct oxygen" Need for ongoing respiratory therapy "She has long respiratory therapy"*<br>Neurological<br>Seizures: *"I did not know he could have seizures. He started to drool, the twitch of the hand fingers, vomited, turned the eye and everything"*<br>Brain damage (not further specified): *"In tomography and magnetic resonance given spot in the brain. According to the doctor, these spots were the cardiac arrest he had in the NICU, the lack of oxygen." "When I took the pediatrician, she suspected hydrocephalus because the head circumference is growing very fast. Hence, the neuro said no, that his brain had suffered a lot, but would recover."*<br>Sleep disorder: *They had a lot of irritability, change in posture, sleep, among others*<br>Development-Gross motor<br>Ability to walk *"He did not walk as a baby, just dragged." "He walked four, my mother never forgot, she says it was a miracle of God"*<br>Development-Special senses<br>Visual impairment *"The first thing I noticed at two years was he, all he would see closer eye toy [...] was all close, there came the glasses"*

Confidential: For Review Only

	"I took the ophthalmologist with less than a year, he wears glasses since baby"<br>*"We discovered his problem because one day was passing a plane and we show and he did not see"*<br>Development-Speech and Social<br>Social difficulties: *"It does not seem that he is seven years old. So I think so, the teacher says he wants to play under the table, his actions are different, in front of people. People treat different, it feels different"*<br>Language disorders: *"how she learned to give kiss? He began to speak phonemes? Everything was the stimulus that we gave"*<br>Parental Support<br>Parental competence: *"In the first months after he left the ICU, everything was already appearing: it was too much crying, irritation, they were not normal, as I had no knowledge ... I did not know and then went back to seek this knowledge"*<br>Healthcare utilisation<br>Frequent readmissions: *all concerned and involved with numerous hospitalizations resulting from respiratory failure*<br>Psychiatric<br>Behavioural disturbances: *They had a lot of irritability, change in posture, sleep, among others "because she did not behave like another child of her age, and I had three children, knew there was nothing right ... The difference was crying, irritation were not normal."*<br>*"Because she did not act like a child of her age, because beyond being premature, I did not deal with it in the crying, agitation."*<br>Need for educational support: *"Then I went behind the neuro pediatrician in Maringa, then came the diagnosis of hydrocephalus, motor problem ... and you'll have to put your child in a special school ."*<br>Relationships with others<br>Effects on family and friends: *" One day he fell ill super the other day and did not improve, went to the doctor and she said it was convulsing, I had never seen him so ... but one thing is that my mother did not let her take medicine because still believed in God, then I do not know if that's why "*<br>Normality<br>Normality: *"Then, after two years, he took more health ... no one says that was born premature."*<br>Other<br>Physical appearance: *"all World wanted to see, because he was too small" "My husband's aunt came to say that she was like lizard, I was very sad"*
Brinchmann<br><br>(2000)	Other<br>Vitality: *"All the pictures, X-rays, ultrasounds and test results indicated that this was an infant we were just keeping alive. But he himself showed signs that he was doing all right, and so things did not hang together."*<br>*"Putting a hand over his eyes was a sign of surplus resources for him" "The doctor said that, whatever we do, however good we are, it is mostly up to the infant himself. No matter how small they are, they can have something within them-selves."*
Brinchmann et<br><br>al. (2001)	Healthcare workers-Knowledge and Competence<br>Healthcare professional competence: *The health professionals said that the ability to sense whether an infant showed vitality came with experience.*<br>Suffering<br>Comfort: *"You can almost feel what it's like in the incubator, lying on the lambskin, that it's how I would want to have laid and . . . Well, it looks very comfortable"*<br>Other

Confidential: For Review Only

	Vitality: *"They had understood that the baby was going to be small, but they had no idea how tiny he was, could not say in advance, 350g. Then the nurse who first took him in her hands after he came out said that he moved his arms, his feet, his body, and cried out, and showed a lot of strength, the moment he was born. She said she believed he could make it because he showed such vitality"*<br><br>*"There was more of a spark in her, they meant that she had more spark and seemed stronger. She had more go"*
Brødsgaard<br><br>(2015)	Gastrointestinal<br>Breastfeeding: *"I fully breastfed for four months-100%-and I am so proud of it."*<br>Development-Gross motor<br>Issues of development and motor skills: *"Issues of development and motor skills, we both looked at my child, research, experience and what I could expect."*<br>Development-Fine motor<br>Issues of development and motor skills: *"Issues of development and motor skills, we both looked at my child, research, experience and what I could expect."*<br>Parental Support<br>Parental competence: *"We learned everything we needed and knew what we had to do, I was quite comfortable when we went home." "Although initially it was scary, I developed the skills, it was a natural progression; we learned how to take care of him."*<br>Healthcare workers-Communication<br>Treating parents with respect: *"In NICU we are on the sidelines, at home we have the main role and you come and advise, [we can] clearly feel the difference."*<br>Allowing time for conversation: *"There was so much information, to take in before we could . . . know whether it [EDP] suited us. There was plenty of time to consider it really thoroughly and ask questions." "Felt very much that there was space and time,"*<br>Relationships with others<br>Bonding with family and friends: *Only when at home on EDP did they feel like a proper family. "I felt divided, torn into two pieces, which were only assembled once I got home."*<br>*In particular, families with older siblings were very enthusiastic about EDP as it had a significant positive impact on siblings' involvement and participation.*
Callen et al.<br><br>(2005)	Gastrointestinal<br>Breastfeeding: *Mother experiencing difficulty assisting infant to latch and position correctly Mother reported having a low milk volume*<br>Parental Support<br>Coping with maternal illness: *Maternally Reported Barriers to Establishing and Maintaining Breastfeeding: Mother is on medications, has poor nutrition, infected incision, high blood pressure, cold, influenza, spinal headache Mother is exhausted, tired, fatigued Mother lacks confidence, is worried, anxious, stressed, depressed, frustrated, guilty*
Catlin (2008)	Respiratory<br>Mechanical ventilation: *'This theme regarding 'Pathophysiology' described the inability of the children to breathe or eat independently. Over 30% of all infant descriptions were about babies who had tracheostomies and were unable to be weaned off a ventilator.'*<br>Gastrointestinal<br>Feeding difficulties: *This theme regarding 'Pathophysiology' described the inability of the children to breathe or eat independently....Thirty two percent of the anecdotal reports related to inability of the infants to eat, suck, swallow or absorb nutrition. Respondents to the survey*

Confidential: For Review Only

	reported nutrition categories as the most frequent reason for infants to remain hospitalized.<br>Fistulas: *Respondents to the survey reported nutrition categories as the most frequent reason for infants to remain hospitalized. One-third of all the children had a digestive issue, such as trachea-related malacias, fistulas, malformations, gut related perforations, absences, short gut and liver failures.'*<br>Liver failure: *Respondents to the survey reported nutrition categories as the most frequent reason for infants to remain hospitalized. One-third of all the children had a digestive issue, such as trachea-related malacias, fistulas, malformations, gut related perforations, absences, short gut and liver failures.'*<br>Other gastrointestinal malformations: *Respondents to the survey reported nutrition categories as the most frequent reason for infants to remain hospitalized. One-third of all the children had a digestive issue, such as trachea-related malacias, fistulas, malformations, gut related perforations, absences, short gut and liver failures.'*<br>Short gut syndrome: *Respondents to the survey reported nutrition categories as the most frequent reason for infants to remain hospitalized. One-third of all the children had a digestive issue, such as trachea-related malacias, fistulas, malformations, gut related perforations, absences, short gut and liver failures.'*<br>Development-Gross motor<br>Issues of development and motor skills: *Respondents were concerned that children were not being taught to sit, crawl or talk as would occur outside of the intensive care unit. When child life personnel were available, they were described as very helpful. They would bring in developmentally appropriate toys and teach the staff how to care for 'growing, 30 pound, NICU babies.'*<br>Development-Speech and Social<br>Language disorders: *Respondents were concerned that children were not being taught to sit, crawl or talk as would occur outside of the intensive care unit.*<br>Parental Support<br>Parental involvement: *"The little boy never left the hospital because his mother was young and single. She had no one to help with the care of this very sick boy. He ended up being in the unit 5 months longer than necessary because we had to get medical foster care to take care of him."*<br>Healthcare workers-Communication<br>Developing a caring relationship: *As the providers gave support to families, families also were described as supporting the staff....It was also difficult for the nurses and physicians to separate from the infants and families after a long period.*<br>Healthcare workers-Knowledge and Competence<br>Identifying who is responsible for care: *Respondents described an absence of review or oversight of infants with extremely long hospitalizations. Just who was supposed to make decisions about children who were extremely and chronically ill? Was there ever an end point? What was the end point? Can interventions once started ever to be stopped? Who was to determine that the burden on the child was now greater than the benefit? .*<br>Expertise in palliative care: *Nurses and doctors talked about being able to clearly recognize that a child was going to eventually die, and the used words such as 'torturing' or 'flogging the child to death' with interventions rather than providing comfort care at the end of life.*<br>Ethical decision making: *Physicians and nurses were upset when other staff members were not willing to stop technological support, even when the interventions were doing nothing to change the prognosis.*<br>Relationships with others<br>Bonding with parents: *Participants reported 78% of parents remaining involved with the child throughout the entire hospitalization, with 23% not staying involved.*

Confidential: For Review Only

	Another problem described were parents who were not able to care for such sick infants. Twenty three percent of the respondents reported parents who were either never involved or who gave up on the children.'<br>Suffering<br>Suffering: *"This infant's short life was never comfortable. He struggled just to breath, day after day, month after month without relief unless he was paralyzed and heavily sedated. I frequently felt we were torturing the child just doing daily care."*<br>*"Finally I guided his parents to let him go. I hated myself and my colleagues for putting me in this situation, a task that should have been done so many months ago. There is nothing to be proud of, just grief and tragedy all around. We did a great disservice to this child."*<br>*"Parents never accepted the diagnosis of a lethal condition. Despite several neonatologists, cardiologists and an ethics committee referral, no one had the courage to discontinue respiratory support. In the last two months has had several cardiopulmonary arrests and was resuscitated because "everyone is scared of the parents"."*<br>Normality<br>Inability to create a normal life: *"We are so busy in our ICU doing our tasks that the chronic kids are left for periods of time alone in their cribs with little interaction except mobiles."*<br>Other<br>healthcare resources: *Of note, the theme 'Cost of Care' was rarely mentioned.*<br>*Although respondents frequently discussed the emotional toll to all concerned, the monetary cost of long-term stays was very rarely (<1%) mentioned.*
Cescutti-Butler<br><br>(2009)	Respiratory<br>Oxygen dependence: *"My babies did not fit into the criteria for going home early due to one of the twins still being dependent on oxygen. However if I had had an opportunity to take them home earlier I would have jumped at it"*<br>Gastrointestinal<br>Feeding difficulties: *"We kept on saying to the staff on neonatal unit that it was only Gray's feeding that was stopping him from going home, everything else was fine" Val explained how her twins were hospitalised for three weeks and for two of those weeks there were no major problems, as during this time her babies just needed to establish feeding. "I felt particularly sensitive around the issue of "topping up" and it upset me when my babies were topped up with formula milk through tube feeds. It made me feel inadequate and a failure for not being able to provide enough milk for my babies."*<br>Parental Support<br>Barriers to parental involvement: *"I felt at times that I had to ask permission to change my baby's nappy or check that it required changing although I understood that it was to protect Gray from too much "messing about" . I did not feel that myself or my husband could open the incubator and take Gray out. We had to wait for a nurse to say "oh, you going to have a cuddle?"*<br>*"You go in every day but you still feel guilty that someone else is taking care of your babies for you even though it's not your fault"*<br>Parental involvement: *"My husband and I were fully involved in the care of our babies and felt able to question and challenge decisions"*<br>*"During our baby's stay in the neonatal unit both myself and Peter were fully involved in our son's care and did most of the caring such as nappy changing and NGT feeds"*<br>*"give the parents the opportunity to care for their babies sooner than expected with help, which to me sounds like a good plan"*<br>*"In terms of being in control-I think it was more that mostly we weren't"*<br>Support from healthcare professionals: *"The care and support from the neonatal staff was fantastic, and without this support I would not have known what I was doing or how to care for my babies"*<br>Healthcare utilisation

Confidential: For Review Only

	Duration of admission: *"When I was told I couldn't bring them home until term I was mortified because that was two months down the road and no way did I want to wait that long"*<br>Healthcare workers-Knowledge and Competence<br>Consistency of decisions: *"One member of staff indicated Christmas and another New Year for the discharge of Gray. We knew that it was only the feeding that was delaying his discharge and so we kept on asking if we could take him home tube feeding. We did not always feel involved in decisions about Gray's discharge despite us asking repeatedly if we could take him home. Just as we had resigned ourselves to being in hospital over Christmas we were suddenly told on the Monday (a week before Christmas) that I could room in that night with a view to going home either on Tuesday or Wednesday at the latest. This was very frustrating as now all of a sudden he's now allowed home!"*<br>Relationships with others<br>Effects on family and friends: *"During the eight weeks of our baby's stay in hospital my husband and I did find it difficult travelling back and forth, because we were mindful of our other child who was only six years of age at the time and we tried to maintain a sense of normality for his sake"*<br>Other<br>Physical appearance: *"My first impression of her was as "a tiny red thing"*
Chang (2014)	Neurological<br>Neurological symptoms: *I can't feel some - my left - this is numb right here.*<br>Development-Gross motor<br>Issues of development and motor skills: *I fall down easily.*<br>Development-Fine motor<br>Difficulty with activities of daily living: *It's almost to the point where she can't pick up a spoon with this hand.*<br>*You have to put the key in the ignition. So he would always have to reach around and it's very hard.*<br>Healthcare utilisation<br>Need for frequent treatments: *Like I have to miss my Friday lunches to go and sit with a teacher for the whole lunch and tell her what's happened. And like I have to I'm always getting called down to the office for another physical therapist to come and talk to me, and I'm always like it's like it's not it's like both that I have to miss school work for it, number one.*<br>Need for lifelong care: *She's been in therapy since she was 2 weeks old.*<br>Relationships with others<br>Peer acceptance: *Like this young girl telling me Friday, the other girls are getting asked out and boys won't talk to her*<br>*I've had four year olds tell me the other kids don't want to play with them cause they have a dumb arm.*<br>*It's hard for fitting in. Like kids will make remarks. Like what's that thing? Why is it like that?*<br>Bonding with family and friends: *My family is really helpful.*<br>Pain<br>Pain: *It like hurts when you wake up in the morning.*<br>Normality<br>Ability to lead a normal life: *I don't like people doing stuff for me at all.*<br>*She would spend all day trying to do one thing so she could do it herself, you know.*<br>Other<br>Physical appearance: *Cause this arm's a little bit shorter than the other one, like lengthwise.*

Confidential: For Review Only

Chia (2006)	Parental Support<br>Parental competence: *"it's a parent education thing too-explaining to them what it actually means and how they can go about doing it and how often and how long it does actually take-you need to sort of explain to parents what to wear."*<br>Healthcare workers-Knowledge and Competence<br>Healthcare professional competence: *"I think they [nurses] need to have a knowledge of it, I think that's fairly important and I am not sure that everybody does have"*<br>Relationships with others<br>Bonding with parents: *"I find it a great joy when the mums do hold the baby against their chest... irrespective of whether it's a primigravida or a multigravida. You get the same buzz out of it and so do the dads."*
Chollat (2014)	Respiratory<br>Asthma: *Table 1: -asthma*<br>Gastrointestinal<br>Eating disorders: *Table 1: -eating disorder*<br>Neurological<br>Sleep disorders: *'sleep disorder' measured*<br>Development-Gross motor<br>Issues of development and motor skills: *Neuromotor deficits CP, unable to walk or walks only with aid CP, but walks without aid No CP but dyspraxia or motor coordination disorder*<br>Development-Fine motor<br>Difficulties with activities of daily living: *(Other motor disorders were defined as coordination disorders, difficulties in cycling, drawing, cutting with scissors, playing with construction toys or puzzles, washing him/herself, dressing or tying shoes, receiving psychomotor or ergotherapy sessions.)*<br>Development-Special senses<br>Hearing impairment: *Table 1: -hearing and visual disorders were measured*<br>Visual impairment: *Table 1: -hearing and visual disorders were measured*<br>Development-Speech and Social<br>Language disorders: *Language disorders were defined by treatment by a speech-language pathologist*<br>Healthcare utilisation<br>Frequent readmissions: *Table 1: -hospital admissions were measured*<br>Psychiatric<br>Autism: *Autism, pervasive development disorders, treated with risperidone Hyperactivity or attention deficit disorder or methylphenidate medication or overall SDQ >17 or SDQ hyperactivity subscale >7 or conduct disorder as reason for a visit to a psychiatrist or a psychologist at age 11 y*<br>Need for educational support: *Has repeated a grade and/or receives/needs special support at school, has schooling difficulties, language disorder*<br>Psychiatric disorder: *Autism, pervasive development disorders, treated with risperidone Hyperactivity or attention deficit disorder or methylphenidate medication or overall SDQ >17 or SDQ hyperactivity subscale >7 or conduct disorder as reason for a visit to a psychiatrist or a psychologist at age 11 y*

Confidential: For Review Only

	Normality<br>Normal health: *Table 1: -parents perception of child's general health was measured*
Darcy (2008)	Neurological<br>Sleep disorders: *The qualitative data show that nurses are aware that increased noise levels in a NICU have detrimental physiological effects for the newborn, including alterations in sleep/wake cycles*<br>Other<br>Growth: *The qualitative data show that nurses are aware that increased noise levels in a NICU have detrimental physiological effects for the newborn...problems with appropriate growth and development.* Physiological stability: *The qualitative data show that nurses are aware that increased noise levels in a NICU have detrimental physiological effects for the newborn, including… immediate changes in vital signs,*
Docherty (2007)	Surgical<br>Need for multiple operations: *the mother also worried that there would be more surgeries*<br>Appearance of scars: *Of particular concern were the scars on Nick's body from medical procedures, surgery,*<br>Parental Support<br>Support from fathers: *Her support system was very limited; her relationship with Allison's father was sporadic and filled with conflict. Because he thought the mother had had an abortion, the father was in shock at the baby's birth and erratically provided support.*<br>Support from family and friends*: In addition, there were issues with her parents, who had custody of two of her other children. Her support network included her mother and the paternal aunt. Others in the family were not accepting of her pregnancy or the baby. The mother felt that the community blamed her for her son's illness, and this eroded her support. As one mother noted: "It would scare me at times because they, I wasn't understanding some of what they was telling me. And I'd stay upset a lot. But then my mom she would call back and she would get understanding."*<br>Barriers to parental involvement: *As with most mothers of critically ill infants, they also struggled with seeing their sick child surrounded by and dependent on technology and worried about the immediate and long-term outcomes. This made visits overwhelming and stressful.*<br>Parental involvement: *Lisa's mother felt special because of her competence in caring for her child, her feelings of responsibility for the child's well-being*<br>*Being a single parent meant that these mothers were often alone in making complex decisions for their infant during hospitalization. One mother deferred all decisions to the staff. Others struggled to learn more about the infant's health problem and became competent in making decisions.*<br>Parental ability to work: *"She's put my life on hold completely. Um, because, I could've started work at a plant that I've been tryin' to get on for the past, longest. And uh, goin' back to school is out, too."*<br>Healthcare utilisation<br>Need for frequent treatments: *the mother also worried that there would be more surgeries*<br>Healthcare workers-Communication<br>Communicating information effectively : *she became upset because she could not understand what the staff told her about the child's illness and treatments "It would scare me at times because they, I wasn't understanding some of what they was telling me. And I'd stay upset a lot. But then my mom she would call back and she would get understanding."*<br>*'Most of the mothers had limited exposure to complicated tertiary care hospitals and were challenged in communicating with highly*

Confidential: For Review Only

Confidential: For Review Only

	educated health care providers, in understanding their infant's complex condition, and in making complex decisions related to care.'<br>Relationships with others<br>Bonding with parents: *she felt very guilty and reported difficulties attaching to her child during her hospitalizations*<br>Suffering<br>Suffering: *she reported feeling...distress at seeing him suffer*<br>Normality<br>Ability to lead a normal life: *the mother also worried that...Lisa would not have a normal life*<br>Survival<br>Survival: *the mother began worrying about whether her son would live*<br>Other<br>Physical appearance: *Of particular concern were the scars on Nick's body from medical procedures, surgery, and the total parenteral nutrition line.*
Ericson (2013)	Gastrointestinal<br>Breastfeeding: *They experienced estimated breastfeeding as a more difficult method because they felt insecure about the infants' intake of breast milk*<br>Parental Support<br>Parental involvement in care: *"The mothers are strengthened in their maternal role and dare to trust their own abil-ity. The mothers can read the infants signals at an earlier stage and in a better way."*<br>Healthcare workers-Knowledge and Competence<br>Staff insecurity: *..Some staff experienced a loss of control after estimated breastfeeding was implemented as they did not know the exact intake of breast milk after a breastfeeding session... ...Some staff did not trust what they saw and what the mother was telling them. The staff considered that sometimes the reduction was too big and the infant did not get sufficient milk and lost weight. Other times, the reduction was too small and the infant got too much milk and vomited. ...Some staff experienced the decision making in estimated breastfeeding as difficult as they had to predict and evaluate decisions made from a more complex viewpoint. ...They experienced estimated breastfeeding as a more difficult method because they felt insecure about the infants' intake of breast milk and struggled to use amore person-centered care approach where the mothers' and infants' states and needs were acknowledged.'*<br>Relationships with others<br>Bonding with parents: *'Staff experienced estimated breastfeeding as a more supportive method for the development of the mother/infant relationship because of the uninterrupted closeness.' 'The staff stated that the mothers stopped comparing how well or how poorly other mothers' infants fed after estimated breastfeeding was implemented'.*<br>Suffering<br>Comfort: *"The infant can lie securely in the mother's arms directly after breastfeeding and not on a hard scale."*
Gaíva (2001)	Respiratory<br>Nasal congestion: *"If having nose congestion is something common for every premature baby"*<br>Gastrointestinal<br>Regurgitation: *"The other day I got really scared, the baby regurgitated, and milk came out of her nose, I went crazy"*<br>Frequency of defaecation: *"A handbook with instructions about elimination, like if it's normal for the baby to go two days without defaecation"*

	Feeding difficulties: *"From the mothers' reports, it is observed that activities like...breastfeeding, become complex when taking care of a baby."*<br>Healthcare utilisation<br>Frequent readmissions: *"I'm afraid of the baby having to return, I think they could talk more about the premature baby, the risks"*<br>Healthcare workers-Communication<br>Keeping parents informed: *"They could provide a manual about what the baby might have" "Preparation for discharge refers to instructions about care with hygiene, feeding – focused on breastfeeding, administrating medications, and returning to the outpatient clinic for follow-up visits. The mothers reinforce their need to receive instructions at the moment of their child's hospital discharge."*<br>*"In view of the statements, it is observed there is a need for educational material covering the care that should be offered to newborns in risk situations."*
Garcia (2004)	Healthcare workers-Communication<br>Communicating uncertainty: *"It's difficult, you know. I think as medical practitioners it's, I think in medical practice we find it difficult to ... We have to accept that we don't have all the answers in terms of treatment ... and of course I'm sure that comes as a disappointment to the families."* Candour: *"It's about trust, you know. I will trust someone who was honest and said he didn't know (more) than someone who lied."*<br>Healthcare workers-Knowledge and Competence<br>Ethical decision making: *"It's difficult, you know. I think as medical prac-titioners it's, I think in medical practice we find it difficult to ... We have to accept that we don't have all the answers in terms of treatment ... and of course I'm sure that comes as a disappointment to the families."*<br>*"but when you actually mention this, say, "Well, in fact you know, we don't really know what's the best treatment," it is a delicate moment. I'm not really bothered from how they perceive me, as a person, because you know, there's lots of things I don't know in life, but their confidence of how their baby's going to be managed, that's the issue that comes up."*<br>Other<br>Iatrogenic harm: *"... I don't think we know...there are potential toxicities, very real toxicities associated with it, so there is this balance of...benefit and harm..."*
Gontijo (2008)	Healthcare workers-Knowledge and Competence<br>Consistency of decisions: *'In interviews with mothers, it becomes more evident, because they claim that only become effectively adopt the kangaroo position when they are sent to Kangaroo infirmary. Even so, kangaroo position, It is not adopted continuously in the second stage.'*<br>Growth<br>Growth: *evident satisfaction with the method, especially about the best development of your child, highlighting the weight gain which is the main factor*
Green (2015)	Parental Support<br>Coping with maternal illness: *Understandably, the nurses were very concerned about the mental health and well-being of all mothers in the NICU, however, their level of concern was heightened for mothers of extremely premature IVF babies. They emphasised that it was only natural that mothers who had suffered multiple losses, and potentially another loss if the baby did not survive, to experience severe psychological distress. Another loss could be the loss of a 'normal baby'. One nurse spoke of her belief that mothers could be diagnosed with depressive conditions, particularly when they took home a baby that had ongoing health problems or a disability, and the mother*

Confidential: For Review Only

	came to understand the reality of her situation.'<br>Healthcare utilisation<br>Inappropriate treatments; *For the nurses, all babies are precious, and while they recognised that pregnancies conceived through assisted reproductive techniques were precious to their parents, they rejected the idea that these babies were more precious than babies who were conceived naturally. The nurses were concerned that the metaphor of 'precious' used to described IVF babies, could create a view that mode of conception will affect the treatment options offered to parents of extremely premature babies.*<br>Healthcare workers-Communication<br>Communicating in challenging settings; *The nurses believed that when parents requested 'everything' (Nurse 13) to save the life of their baby, they would not know what 'everything' meant.*<br>Healthcare workers-Knowledge and Competence<br>Ethical decision making; *...all of the nurses who were interviewed had seen and worked professionally with many families with a history of infertility, and hence had first-hand experience witnessing the complex dilemmas and realities faced by infertile couples*<br>*The nurses understood the parents' desperation to have a baby and experience parenthood. They believed on the other hand that such desperation should not be the sole determiner of whether the baby survived.*<br>Suffering<br>Suffering: *"It's hard looking after a baby where it's being kept alive just to meet the needs of the parents, because they want a baby." Participants knew the baby would experience pain and suffer from intensive care therapies. All the nurses believed that they were affected by the baby's suffering, because it was "agonising for the staff who have to look after them"*<br>Survival<br>Survival: *"It's their one and only chance of having a child. They can only see that immediate sense of having a child. . .they want to take home a baby."*<br>Survival with disability: *"As hard as it is for us to understand, they are happy to have a child of their own regardless of what it is. They're happy to suffer the consequences, so to speak, just to say that it's their own; that it's something that they can love."*<br>*"It isn't up to us to say what is quality of life, because parents might think that even if the child was disabled, that it was better than not having a child at all."*<br>*"They want to take a baby home and often voice that they don't care what the infant is like."*<br>*"To have something that's alive, I can understand that. I can understand somebody saying, 'I want that infant in preference to nothing at all'."*<br>Other<br>Long term effects on parents: *"We should be looking at Postnatal Depression after the baby goes home. . .Once they actually get a baby home, that's when the reality sets in. All the triggers are there."*
Gregory (2012)	Respiratory<br>Oxygen dependence: *Oxygen status*<br>Gastrointestinal<br>Feeding practices : *Participants were asked about the clinical criteria that are used to determine initiation, delay, and advancement of enteral feeding and about the specifics of enteral feeding administration (ie, bolus vs continuous, frequency of feeding, type of feeding, and nutritional fortification added to feeding). Finally, participants were asked to report the volume at which patients were considered to be at "full feeds" and how PN was discontinued.'*<br>Infection

Confidential: For Review Only

	Sepsis: *Identified outcome: Sepsis*<br>Healthcare workers-Knowledge and Competence<br>Consistency of decisions: *Compliance with guidelines Practice patterns of the physician Practice patterns of the nurses*<br>Growth<br>Growth: *Time to return to birth weight*
Griffin (1997)	Parental Support<br>Preparation for NICU admission: *Parents described benefits of the tour, including that it (a) decreased their fears, (b) inspired hope for their newborn’s prognosis, (c) provided reassurance about care in the NICU, and (d) prepared them for their newborn’s NICU hospitalization. All parents described at least one of these benefits, including five mothers who said the tour was overwhelming or difficult because of the appearance of the newborns.*<br>*'For several mothers, the tour inspired hope for their newborn’s prognosis, especially when the mothers saw very premature infants who were said to be progressing well.*<br>*Parents evaluated and provided suggestions on the way the tour was arranged and conducted and offered advice to other parents. In general, all parents recommended that parents in similar circumstances should be offered a prenatal tour of the NICU.*<br>*Parents advised that more health care providers suggest tours to parents diagnosed with a high-risk pregnancy. Two mothers also recommended that other perinatal health care providers should tour the NICU so that they can be supportive to parents. One mother perceived that her need to tour the NICU was not supported by the staff on the antepartum unit.*<br>*Parents who toured with their partners commented that having each other as a support person was helpful. They recommended that the tour be scheduled so that the partner or other support person could accompany the parent.*<br>Healthcare workers-Communication<br>Communicating information effectively: *Parents re-ported that it was important to receive detailed information on the following: (a) newborns who had a di-agnosis or gestational age similar to what was antici-pated for their newborn; (b)a description of equipment for the newborn; (c) roles of staff members; and (d) a description of the parental role in the NICU, including the visitation polic It was important to parents to hear about the parental role....However, all parents did not perceive that they received adequate information on the parental role.*<br>*The need for more specific information became apparent to parents after their infants were cared for in the NICU. These parents indicated that they wanted more information on expectations for their role in the NICU, breastfeeding, sibling visitation, and the potential for the newborn to be transferred from the NICU to another unit before discharge. Two parents suggested that handouts could supplement or reinforce information that was given during the tour and assist parents to inform family and friends about the NICU.*<br>*Parents reported that the tour should be individualized to meet the specific needs of parents. Parents perceived the tour as individualized when they went as a couple or an individual rather than in a group, had an opportunity to ask questions, and saw newborns who had a diagnosis or gestational age similar to that expected for their newborn. Therefore, it was critical for the nurse conducting the tour to know the parents’ maternal-fetal diagnosis. Several parents made additional suggestions, such as having an opportunity to go on a second tour or changing the order in which the NICU patient care areas are shown; these demonstrate the parents’ individual needs.*<br>Healthcare workers-Knowledge and Competence<br>Healthcare professionals behaviour: *"Knowing they do care about them and they do realize they are human and not machines . . .you could feel that they really cared and worried"*<br>*Parents reported that the tour was comforting and reassuring because it gave them an opportunity to observe the type and quality of care that the infants received.*

Confidential: For Review Only

	Hearing specific information about primary nursing also helped some mothers to feel more comfortable. These mothers explained that it was reassuring to know that their questions could be answered because the primary nurse would know their infant.<br>*Most parents reported that the nurses who conducted the tours were knowledgeable and comforting. These nurses were described as compassionate, concerned, helpful, and considerate of the time parents needed to understand the information and ask questions.*<br>Staffing levels: *It was especially helpful for the parents to see so many nurses and physicians in the NICU.*<br>Relationships with others<br>Effects on family and friends: *The tour gave mothers information about the NICU they needed to share with other family members. One mother indicated that she had gained an understanding of the unit and was better prepared to talk to her child about the NICU. Three of the four mothers who were not accompanied on the tour by the fathers reported that they shared information about the NICU with the fathers, which was comforting to them. One of these mothers described her husband's reaction to their infant's admission to the NICU, "My husband was calm because I had already told him what to expect."*<br>Normality<br>Thriving: *"The tour gave me hope that he was going to be fine. Seeing babies younger than him thrive. . . and then seeing the babies approximately his age survive, thriving, and doing well."*<br>Other<br>Physical appearance: *"it's still frightening to see babies that small."*
Hanrahan<br><br>(2007)	Gastrointestinal<br>Choice of milk for feeding: *"it's like they [scn providers] didn't inform us when they were trying to feed my daughter [formula]"*<br>Parental Support<br>Care provided close to home: *"and nurses' opinions would come into it, too. if they're aware…that parents aren't showing up, including in the evenings…then that may weigh into the decision-making process…make us want to get the baby close to home because we know the parents can't get here."*<br>*"[i was glad our infant was transferred to the com-munity hospital] because of lack of transportation.Because then i [was] able to see him more."*<br>Healthcare workers-Communication<br>Communicating information effectively: *"i don't think that [it] is communicated as well that your baby's well enough to go back to a unit that isn't as intensive as the one that it's coming from."*<br>*"they were originally using all these technical terms [about back transport], and he [the infant's father] was like "What is that? slow down, we still have no idea what it is.""*<br>Keeping parents informed: *"i mean, i found at [the nicu] they explain more to you what was happening with my daughter, what they were doing, how well she was doing. You know, at [the scn] you would go there and they're just doing something. they're not—they don't have time. You know you have to approach them…and say, okay, what's going on now? how well is [my infant] doing today? You know, stuff like that. i want them [scn providers] to, you know, talk to me, let me know, don't sugar coat what's, you know, going on."*<br>*"it's like they [scn providers] didn't inform us when they were trying to feed my daughter [formula] and she was on breast milk, so she threw it all up.…they didn't tell us until we got up there."*<br>*"i asked so many questions and read all the charts every day, and i probably angered them. squeaky wheel gets the oil, as they say."*<br>Treating parents with respect *"i got yelled at by a nurse at [the scn] for rubbing my son's foot [even though that was okay at the nicu]. and rubbing his hand while he had a hold of my finger. i was just rubbing his little palm, and then she told me not to do it because it would stimulate him." "i would've liked them to greet me as a parent—you know, acknowledge me."*

Confidential: For Review Only

	"[My experience at the scn] could have been better; they could have made me feel comfortable there."<br>Healthcare workers-Knowledge and Competence<br>Staffing levels: *"[at the scn] you're not gonna have somebody there all the time, right next to your baby."*<br>*"one time we went [to the scn] and it was like eight-thirty at night. and there was only like three or four nurses in there, and it was a full room. and there were babies in every bed. and they were having a lot of problems with this one little boy, and they left all the other babies and went to him. so i ended up staying there real late because i wasn't gonna leave my daughter by herself and have something happen to her while they're just with this one child. You know, i think staffing is a really big issue [at the scn]."*<br>Identifying who is responsible for care: *"sometimes we're not real clear who to follow up with."*
Hefferman<br><br>(1999)	Gastrointestinal<br>Other gastrointestinal malformations: *"One mother, left with one surviving twin who has an ileostomy, told me that she is glad the sibling died and does not have to endure what the living twin is enduring."*<br>Surgical<br>Requirement for ileostomy: *"One mother, left with one surviving twin who has an ileostomy, told me that she is glad the sibling died and does not have to endure what the living twin is enduring."*<br>Healthcare utilisation<br>Need for lifelong care: *"MDs are making such choices for parents and when the outcome is disastrous they just expect parents to take home severely handicapped babies and deal with life-long problems."*<br>Suffering<br>Suffering: *"Three weeks of high pressure only proved to prolong her death rather than improve viability...Being one of the nurses at that bedside every day was frustrating and often agonizing. I definitely feel that even with all our technical expertise, we failed to give good and sensitive care to both this particular infant and her parents."*<br>*"The problem [with] our high tech machines is not only do they prolong life, they often prolong death. I am not saying we should never attempt to salvage VLBW babies. We have some miraculous success stories. But we should have some guidelines to follow and the decency to stop before we begin to do harm."*<br>*"It often seems that when we resuscitate these edge of viability infants it is to please the parents, especially in the IVF environment that we have today. The concern is not for the comfort of the infant; the concern is to give the parents their child no matter what."*<br>*"One mother, left with one surviving twin who has an ileostomy, told me that she is glad the sibling died and does not have to endure what the living twin is enduring."*<br>*"We poke, prod, and torture them for weeks, give a lot of false hope to the parents, stress the staff, and then they are taken off and allowed to die . . . for us it is ethically the worst part of our job."*<br>Normality<br>Normality: *"It is hard to care for very small sick infants whose potential as a normal child and adult is poor."*
Hentschel<br><br>(2006)	Healthcare workers-Communication<br>Awareness of parental views: *The data show that in 9 cases the clinical team had no knowledge of the parents' wishes.*<br>Pain<br>Pain *: Table 3 -summary of medical staff's perceptions of parents' wishes concerning RIC* [Restriction of ongoing Intensive Care] *Agree to RIC: "Avoidance of pain/suffering"*<br>Suffering

Confidential: For Review Only

	Suffering: *Wishes of parents as percieved by medical staff “Avoidance of pain/suffering” “To let the infant die in peace”*<br>Normality<br>Normality: *Wishes of parents: “A healthy child” “To take the child home”*<br>Survival<br>Survival: *-summary of medical staff's perceptions of parents' wishes concerning RIC: “Survival of the infant” “Maximum therapy” “Therapy even in case of low chance”*
Holditch-Davis<br><br>(2003)	Gastrointestinal<br>Nutritional intake: *"Ever since he's been home, we've kept records of how much he was eating, on a daily basis."*<br>Neurological<br>Seizures: *"I thought I'll time it and see how long these things last-which was anywhere from 5 to 7 minutes. And his color would change, and the way he did his eyes. They rolled. And his eyes would water, and he would tense up. I explained this to the doctor. And he was the one that said it could possibly be seizures. So we're going to take him in and have him tested. But this is something that we were told could be a possibility, and I don't see it as anything major."*<br>Infection<br>Susceptibility to infection: *"particular about her germ thing. I don't want her chewing on every-body else's toys or what have you." "The biggest worry right now is when he is going to get sick. I'm sure he's able to handle it, but at the same time, we're overly protective and don't let people in his face"*<br>Healthcare utilisation<br>Recurrent sickness: *"We've only put him with other children for the past month. The biggest worry right now is when he is going to get sick. I'm sure he's able to handle it, but at the same time, we're overly protective and don't let people in his face."*<br>Relationships with others<br>Childhood happiness: *"But to me I look at her and I don't see it all. I see this little happy little thing!"*<br>Normality<br>Normality: *"And how helpless he was and that I couldn't hold him. That was really the hardest thing."*<br>*"But I don't look at her as a premature....But to me I look at her and I don't see it all. I see this little happy little thing!"*<br>*"Is he going to be his normal size? . . . If he was supposed to be small then that's fine. He can be small. But I guess I'll never know."*<br>Survival<br>Survival *"It hurts. I didn't know, I didn't know if they were going to make it or not."*<br>*"I have a fear of them dying. I'm still scared because they were premature that that could happen to us. And grant-ed that could happen even if they weren't premature!"*<br>Growth<br>Growth *"The most difficult thing for me is the weight thing. Is he big enough? Is he not big enough? Should he have been bigger? Would he have been bigger? How big would he have been when he would, if he was born when he was supposed to be born?"*<br>*"just look at her as a little baby. . . . The doctor says she's 3 lb under weight. So that's like 20% bodyweight."*
Hussey-<br><br>Gardner (1998)	Healthcare workers-Communication<br>Developing a caring relationship: *"I couldn't get over how gentle she was. It seemed like she was truly concerned"*<br>Normality<br>Normality: *"Being reassured that he was on line for how old he was...Just reasurance he was doing well"*

Confidential: For Review Only

	Growth<br>Growth: *"She was born so early, it's nice to see that she's finally catching up with how she's growing"*<br>*"I asked more questions about where he was supposed to be..."*
Jirapaet (2006)	Respiratory<br>Mechanical ventilation: *"I couldn't find time to change the adhesive tape, that was wet by saliva, of the endotracheal tube of another baby … it caused tube dislodgment …"*<br>*"I was filling [free flow of] water into the humidifier chamber and was distracted by a call to assist a physician in an emergency case. I for-got to close the line clamp … water flowed into the baby's lungs unnoticed."*<br>Skin<br>Extravasation injuries: *"The i.v. site was covered with cloth.... leaked for a long time, skin area was inflamed and edematous like popeye's arm."*<br>*"I was unsure if the baby had an IV leakage and asked a senior nurse to confirm it. But she was busy …. I saw the baby again when there was tremendous edema."*<br>Burns; *"wasn't lazy in changing the pulse-oximeter sensor site [recommended to relocate q3-4 h] … But I didn't believe that it could really burn the skin until I had experienced it."*<br>Pressure sores: *"I had to skip changing a [hydrocephalus] baby's posi-tion for that shift … Her head developed pressure sores."*<br>Healthcare workers-Communication<br>Communicating information effectively: *"Doctor wrote a new order on the patient chart and left it on the desk, but didn't say a word …. The order should have been taken but no one noticed it."*<br>Healthcare workers-Knowledge and Competence<br>Medical errors: *"I had to draw an antibiotic from a vial that was diluted by another … without knowing that it con-tained the wrong preparation …"*<br>*"Doctor wrote 3 like 5."*<br>*"I set the infusion rate at 12 mL/min instead of 1.2 mL/min. I couldn't see the faint decimal point."*<br>Other<br>Iatrogenic harm: *"I performed a venepuncture without using a radiant warmer properly … baby developed hypothermia."*
Johnson (2007)	Parental Support<br>Parental involvement: *Being involved in caring for the infant was described as being needed by every mother in the study.*<br>Relationships with others<br>Bonding with parents: *"made me realize that I know my little girl"*<br>*Many of the mothers spoke how holding helped them "understand" their baby's needs better Mothers related feelings of "being needed."*<br>Suffering<br>Comfort: *"I will make you feel so much better in my arms,"*<br>Normality<br>Normality: *"mommy will sing to you our favorite song for as long as you want"*<br>*"loud noises the rest of the time, we can escape to be alone for an hour."*<br>*as one mother stated, "you can't learn how to be a mother from a book. You have to learn with your baby."*

Confidential: For Review Only

Jones (2015)	Gastrointestinal<br>Breastfeeding: *"it was her first time and just explaining to her how, the right way to breastfeed and attachment and things like that (Nurse 9)"*<br>*"We went through from start to finish any questions she had. We ran through a breastfeed, we had all the props out, and just with the use of the props and things it was a bit easier …It actually worked out really, really well. After that, we got the baby to breastfeed beautifully and it was nice"*<br>Parental support<br>Culture differences: *But I think sometimes some of the Aboriginal Community can also be hard to talk to. I think that's just their environment that they've been bought up in*<br>Support from healthcare workers: *They'd say "Well at the end of the day go with your feelings of what, if you feel she's feeding really well when you do a certain thing, then keep doing it and if you do something else it and doesn't work, then stop doing it,"… "You will know best because you just know how it works with your new baby"*<br>*'So it's just about giving them lots of options and supporting them to make the choice that's right for them'*<br>Support from family and friends: *For example, one woman requested that her friend be able to come into the nursery and hold her baby. The woman had no family members in Australia, and her friend was the only person providing support. The nurse caring for her baby refused her request on the grounds that the unit had a 'policy' that only parents and grandparents could hold the baby. This created distress and conflict.*<br>Healthcare workers-Communication<br>Communicating information effectively: *One of the most common and important issues for both parents and nurses was the concept of sharing 'information'. Parents 'wanted' nurses to 'provide' information and nurses 'wanted to provide' parents with information. Interestingly, fathers had a tendency to mention information first compared to mothers. From the parents' perspective they wanted to know 'everything' from what they considered to be 'general information' about for example what 'creams' they should use on their baby to how equipment worked and, of course, the condition and progress of their baby.*<br>*I feel like I'm receiving all the information that I need, so I'm really extremely happy about that even though sometimes we're not asking questions, they will give us additional information*<br>Communicating in challenging settings:*I think your very young mothers, very hard to talk to because they're babies and they don't understand, they see us more as a authority figure as in someone that could be their friend, and be helpful ……*<br>Developing a caring relationship: *Nurses who 'listened' and were 'empathic' were highly valued. Parents also used words such as 'friendly' 'approachable' and 'caring' to describe the communication style of supportive nurses; I just felt she had a way with words and made me feel really safe (Regional Mother 4).*<br>*I have been nursing for a while now, the most important thing you would do with any patient; whether it a baby, whether it an adult, you build up a rapport*<br>Keeping parents informed: *She rang the previous room, and they told her, "Well, your baby's not here anymore," so they put her through to my room, I got the phone call, to me and she was like, well, just a bit cranky at the fact her baby had been moved without any prior knowledge to it, and I coped a brunt of it (Nurse 13).*<br>Treating parents with respect: *Yeah, I think the nurse was worried about the whole touching and whatnot … Like she's very full on with protocol but she was very abrupt and didn't explain things. So she upset mum and this upset me a bit too (Metropolitan father 26).*<br>Healthcare workers-Knowledge and Competence<br>Consistency of decisions:*'Everybody had a different point of view but they were opinions, not facts. So that was huge, don't even get me*

Confidential: For Review Only

	started on that, that was just a nightmare. (Regional mother 6)'<br>Healthcare professionals behaviour: "*I asked "Can you come?" 'Cause they had to check that it's down the right tube, …. And she didn't come and do it and I had to go and get her and say, "Can you help me?" 'Cause I'm not allowed to do it by myself. And then she was talking and it took her 5 minutes to finish her conversation with the other nurse, and it wasn't a nurse related conversation it was just a casual conversation … Like I felt a bit (sic) she wasn't their priority."*<br>Normality<br>Normality: *A major focus for parents was seeking information that told them that what was happening was 'normal' and that everything was going to be 'fine'.*
Kavanaugh<br><br>(1997)	Parental Support<br>Support from family and friends: *Certain behaviors of family, friends, and health care providers were perceived by parents as supportive. Some behaviors that were described as helpful by the parents were not unique to wny specific relationship, whereas others were confined to relationships with health care providers. Three behaviors that were not specific to any particular relationship were accepting the parents' feelings and behaviors, being there, and sharing the experience.*<br>*Parents reported that they did not attend a support group because they could not share their experience in a large group or because their family provided adequate support.*<br>Support from healthcare professionals: *The other behaviors described only with respect to health care providers were giving information, providing competent care, and giving special attention.*<br>*To the parents, being there meant being available. Nurses were available to the parents by being physically present or by being close by if the parent needed the nurse, especially if the mother was alone during labor....Parents used the term "share" when they described individuals who genuinely understood their experience.*<br>*Only one mother recalled sharing the loss with someone who was not a bereaved parent. This mother described her experience with a nurse during the delivery.*<br>*Parents perceived that health care providers gave them special attention by breaking hospital rules, giving additional time, and making the parent a priority among other patients. Breaking hospital rules included expanding visitation to allow for unrestricted visits from family and friends.*<br>*Most parents could not identify the reason for inadequate pain relief. One mother stated that she was denied an epidural by an anesthesiologist, who told her that "women have been doing this for thousands of years without an epidural.*<br>Healthcare workers-Communication<br>Communicating in challenging settings: *"And I don't want some fluke off the wall, you know. You don't have to tell me. But what I'm saying is the things that have a reasonable chance of happening" the parents reported that when delivery was imminent, the obstetricians explained that the newborn would have had a better chance of survival had the pregnancy been maintained for an additional few weeks. In the other instance, the mother reported that the obstetrician told her about the likelihood of her twins' survival when she was treated for preterm labor.*<br>*Only one couple perceived that they were given an opportunity to discuss how much aggressive care they wanted for their twins. This couple was the only one to report that they felt prepared for their twins' deaths even before they were born.*<br>*Certain decisions, such as choosing the type of burial, were especially difficult because of the parents' emotional state or lack of experience. Most parents had no experience making funeral arrangements. All parents reported that it was helpful to have information, guidance, and additional time before making final decisions.*<br>Keeping parents informed: *All parents reported that they wanted health care providers to keep them informed of the mother's and*

Confidential: For Review Only

	newborn's condition and treatment plans. When health care workers' provided honest, straightforward information in understandable language, parents gained a sense of control over the situation and their fears decreased.<br>Healthcare workers-Knowledge and Competence<br>Health professional competence: *Most of the parents recalled specific incidents that they perceived as poor medical care; typically, these incidents involved technical procedures or medical knowledge...Parents expected expert knowledge, pain relief, and consistency in care: "Team" was the word used to describe staff who were organized and had communicated well among themselves, especially when transfer of care occurred between shifts or units.*<br>Relationships with others<br>Effects on family and friends: *Family and friends were perceived as available to parents when they visited or called. Parents stressed the importance of others' acknowledging their loss and being willing and unafraid to talk to them. Comments that parents perceived as inappropriate were those that minimized the loss, provided unsolicited advice, or criticized the parents on their health care.*<br>*Breaking hospital rules included expanding visitation to allow for unrestricted visits from family and friends.*
Koh (2007)	Healthcare workers-Communication<br>Using aids to communication: *Most of the mothers in both groups were positive about having their conversations with the neonatologist taped.*<br>*six of the mothers in the control group could not recall their conversations with the neonatologist*
Leavy (2015)	Skin<br>Appearance of scars: *In addition, hospitalization and different interventions in their first days of life have left marks on their bodies. Such marks may become a stigma if their peers give them a negative meaning, leading to prejudice and discrimination*<br>*"I do not like the scar on my belly [...]; I was at the beach and everyone kept staring at me like `That is a big scar!´"*<br>*Being afraid of becoming the laughingstock may make them feel insecure, thus affecting the construction of their own identity: "I think about these things (scars) when it comes to girls; it makes things more difficult because girls think you have washboard abs but your stomach is actually all flabby because of a poorly healed scar resulting from a surgery" (FG 6), said one participant in relation to a keloid resulting from a surgery. However, some take ownership of these marks and give them a new, positive meaning: "When I was young, I felt embarrassed when I had to take off my t-shirt, I thought they would all stare at my scar, and I developed a body image complex. Now I have tackled this issue and it has become part of my story, thanks to this scar I am alive" (FG 9). In this case, the scar becomes the footprint of a surgery that allowed the participant to survive, and this is more significant than the stigmatizing vision of others.*<br>Surgical<br>Appearance of scars: *"I do not like the scar on my belly [...]; I was at the beach and everyone kept staring at me like `That is a big scar!´"*<br>*"I think about these things (scars) when it comes to girls; it makes things more difficult because girls think you have washboard abs but your stomach is actually all flabby because of a poorly healed scar resulting from a surgery"*<br>*"When I was young, I felt embarrassed when I had to take off my t-shirt, I thought they would all stare at my scar, and I developed a body image complex. Now I have tackled this issue and it has become part of my story, thanks to this scar I am alive"*<br>Development-Gross motor<br>Ability to walk: *"I received lots of stimulation; otherwise, I would have ended up in a wheelchair."*<br>Development-Speech and Social<br>Language disorders: *"I also had a hard time learning to talk."*<br>Healthcare utilisation

Confidential: For Review Only

	Frequent appointments: *"I felt left out, I was always missing school because I had to go to the hospital for check-ups"*<br>*Mixed feelings are observed in those participants who have special health care needs at the hospital, from familiarity and thankfulness to rejection and discomfort: "It is a complicated subject because I am alive thanks to the hospital, but I am tired...Now I just go for scheduled check-ups, but when I am there, I just want to leave"*<br>Healthcare workers-Communication<br>Communication with ex-neonatal patients: *Some participants indicated having had a negative experience with health teams: "They said that I was not going to walk and that I was going to be blind, deaf and mute. They would give me no hope and say: 'This baby will not make it to the sixth day…´" (FG 8). "My father was told that when birth is premature, either the baby or the mother dies... but here we are, both of us are alive" (FG 5). Likewise, they felt uncertain about what aspects of their present health status were actually related to their history of premature birth.*<br>*The hospital and the relationship with the medical team play a significant role, which is sometimes contradictory. On the one hand, many participants stated that they were thankful and appreciated the bonds established in the hospital setting. On the other, they often mentioned how medical diagnoses were uncertain or pessimistic regarding their survival and potential sequelae.*<br>Psychiatric<br>Need for educational support: *Overlapping of special health care needs and school hours resulted, in some cases, in a poor school performance: "I felt left out, I was always missing school because I had to go to the hospital for check-ups" (FG 10). Repeating a grade was frequently associated with learning difficulties and an achievement gap in relation to classmates. Several interviewees had to attend a remedial education program. Most of them felt comfortable in such setting because it helped them overcome their learning difficulties and favored their socialization: "I attend a special education school because when I was in a bilingual school I could not advance to the next grade, the other kids mocked me or left me aside, they did not understand my problem. I am doing fine now" (FG 7).*<br>Relationships with others<br>Overprotective parent-child relationship: *They perceive their parents consider them to be more vulnerable and dependent on their custody than the rest of their children or their peers. This may lead to inter- and intragenerational conflicts: "I have always been treated differently than my sisters. Yes, they tend to be overprotective, that bothers me" (FG 2)... This is a traumatic experience for parents, and it is projected into their present relationship with their children...When overprotection is based on giving excessive care to preterm children, the relationship between parents and children becomes distorted. In this regard, a quadriplegic adolescent stated: "All moms left, but my mother would stay all morning at school with me [...]; I started caring for myself and I had to push her away a bit so that I could lead my own life" (FG 5).'*<br>Suffering<br>Ex-patients separation from their suffering: *For ex-preterm subjects, prematurity is more about their parents' experience than about themselves: "When I tell my story, I use the third person; my mom went through this, not me. She considers my story her own" (FG 1). "We are based on what we were told, not on what we experienced" (FG 8). Participants also emphasize their parents' suffering and uncertainty: "Parents suffer much more than us; they had to suffer waiting for us to get better and see us in that status" (FG 1).'*<br>Normality<br>Being treated normally; *"I have always been treated differently than my sisters. Yes, they tend to be overprotective, that bothers me" "I wanted to have a piercing but my mother would not let me because they gave me lots of pricks when I was hospitalized and I had suffered, but I do not remember it"*<br>Survival<br>Survival: *"I am proud of having been born preterm. I almost passed away, and so did my mom. We both made it and it was a miracle"*

Confidential: For Review Only

	Beyond life experiences, even though most participants recognize medical efforts, they attribute their survival to a miraculous and extraordinary event.<br>*"Now I have tackled this issue and it has become part of my story, thanks to this scar I am alive"* Schooling: *"I attend a special education school because when I was in a bilingual school I could not advance to the next grade, the other kids mocked me or left me aside, they did not understand my problem. I am doing fine now"*<br>Other<br>Self-identifying as premature: *'The meaning that subjects assign to having been born preterm varies according to their health status, sequelae and life experience. Those who had sequelae and special health care needs 18 in their childhood associate their preterm birth to a life of efforts and difficulties*
Lee (2005)	Parental Support<br>Culture differences: *3 families felt strongly that their stress derived from differences in the medical management approaches between the United States and their homeland.*<br>Parental involvement: *almost all of the parents expressed a desire to become more involved with the care of their infant*<br>Healthcare workers-Communication<br>Language barrier: *the stress derived from communication with HCPs was mostly related to language barriers for the new immigrant parents.*<br>Keeping parents informed: *Other parents experienced stress from...not being thoroughly informed about their infant's condition or treatment plan.*<br>Communicating information effectively: *Other parents experienced stress from unknown medical terminology*<br>Healthcare workers-Knowledge and Competence<br>Healthcare professionals behaviour: *HCP* [Healthcare professional] *behaviour as stressor for parents*<br>Identifying who is responsible for care: *2 families expressed frustration related to frequently changing bed spaces or hospital units and difficulty accessing doctors.'*<br>Relationships with others<br>Effects on family and friends: *Worry about the possibility of upsetting their own parents. Three families did not tell their own parents about their infant's problem because they were worried that it would upset the grandparents.*<br>Family resources: *3 families felt overwhelmed by a lack of resources (especially in the area of family support)*<br>Other<br>Parental perception of uncertainty: *parent's perception of infant's severity of illness -parent's perception of infant's illness and future impact (i.e. non-specific developmental issues)*<br>Physical appearance: *Both mothers and fathers found their infant's appearance and behavior to be the stressors with the most impact*
Lou (2009)	Respiratory<br>Mechanical ventilation: *"She had been put on a respirator. It was not so bad as I had imagined. I could see that she calmed down. She did not struggle so much and…But the doctors gave me a shock when I came to see her. They had taken an X-ray, and they could see that her lungs were white and stiff and I was told that she was not far from dying"*<br>Frequent respiratory illnesses: *"There were lots of masks and nebulisers during those years"*<br>Gastrointestinal<br>Oral feeding: *"It is small things.. I remember the very first time he ate porridge"*

Confidential: For Review Only

	Neurological<br>Sleep disorders: *Subsequent to an account of the son's disturbed sleep at night for several months after discharge, which was an enormous challenge to the couple, a mother described the boy as follows: "He was such a giving child, full of joy!" … adding in a low voice… "Most of the time".*<br>Skin<br>Appearance of scars: *"The only reminder today is an ugly scar"*<br>Development-Fine motor<br>Ability to feed themselves: *"It is small things.. I remember the very first time they ate porridge. In just stod there in the kitchen and cried my heart out...it was the fact that I was able to experience my children eating with a spoon. It was great!"*<br>Development-Special senses<br>Visual impairment: *At the time of the interviews, the only major sequel was in one child with seriously impaired vision.*<br>Healthcare utilisation<br>Need for frequent treatments: *"There were lots of masks and nebulisers during those years"*<br>Psychiatric<br>Dyslexia: *"We were told that when he started school he might have problems, dyslexia or learning dis-abilities."*<br>Need for educational support:*"We were told that when he started school he might have problems, dyslexia or learning disabilities."*<br>Relationships with others<br>Bonding with parents: *"He might also… When I was working in the kitchen, he might come running at full speed from the family room, hug me.. around my legs.. he was no bigger than that.. and say: "Oh, you are the very best mum".. and off he went again.. He just wanted me to know!"*<br>Normality<br>Normality: *Finally, a mother called it a developmental land-mark when an older sister dared show her irritation towards her little brother, "no longer treating him as if he were made of glass"*<br>*"They get up and try again until they succeed. That is the same way he keeps on trying."*<br>Survival<br>Survival: *"They had taken an X-ray, and they could see that her lungs were white and stiff and I was told that she was not far from dying."*<br>*"Still, I consider the most important moment in my life.. the very second I pushed the pram over the doorstep of the neonatal unit. I remember it very clearly. I had been looking forward to that for two months."*<br>Survival without disability: *"And afterwards you are worried about how they are going to survive. If they would have impairments, and so on."*
Malhotra (2015)	Respiratory<br>Mechanical ventilation: *Significant factors that make professionals categorise a baby as 'sick' High ventilator settings*<br>Oxygen dependence: *Significant factors that make professionals categorise a baby as 'sick'… Oxygen of over 30%*<br>Cardiovascular<br>Hypotension: *Significant factors that make professionals categorise a baby as 'sick' "hypotension"*<br>Presence of Patent Ductus Arteriosus: *Significant factors that make professionals categorise a baby as 'sick'…PDA*<br>Gastrointestinal<br>Necrotizing enterocolitis: *"If we are trying to stimulate the gut before it is ready making it more prone to having complications of either*

Confidential: For Review Only

	feeding intolerance or, more severely, necrotizing enterocolitis."<br>Initiating enteral feeds: *"MEF* [minimal enteral feeds] *should be initiated in first 2-3 days of life as long as the baby is stable."*<br>Healthcare workers-Knowledge and Competence<br>Consistency of decisions: *"it is difficult to have faith in the decisions when they don't even agree with each other"*
Miljeteig (2006)	Parental Support<br>Maintaining hope: *The informants described how parents thought there was hope so long as the child was in hospital, and the doctor's dilemma consisted in both keeping the parents' hope alive and in giving them a realistic picture of the situation and of probable outcomes.*<br>Healthcare workers-Communication<br>Communicating with parents: *Most of the informants reported dilemmas in relation to parents. The most frequently mentioned problem was communication with parents of poor education and low socio-economic status. Many said that parents did not understand medical terms, and were thus unable to appreciate the seriousness of their child's condition... "How do you explain 'brain-dead' to a person who does not understand what a 'brain' is?"*<br>Healthcare workers-Knowledge and Competence<br>Ethical decision making: *All of them underlined that it was, and had to be, the most senior doctor or registrar who took ethically difficult decisions. Many found that they had no influence on this matter, but all except two had in some way been involved in decisions concerning withdrawal. Some of them focused primarily on how to keep the baby alive, while others were more concerned about when to apply or withdraw treatment. In the latter cases, emphasis was laid on the available medical alternatives, on data concerning survival and treatment results for similar children at their unit, the resources available, the law against euthanasia, and the parents' situation....All the informants, except one, claimed not to be influenced by their religious beliefs. Several referred to God as the ultimate decision maker, and regarded the relevant decisions as a practical rather than an ethical challenge... Many also said that it was easier for them to handle these situations than for their Western colleagues, because they were used to seeing children die.*<br>*Whether or not to involve the parents, or to seek their co-operation in decision making, was a frequently mentioned dilemma, although not all informants were convinced that parents ought to be involved in decisions concerning withdrawal of treatment. One problem experienced was that some parents perceived the doctors as gods. These parents tended to agree to almost everything that was recommend to them and refused to participate in decision making. Many of the doctors said they felt uncomfortable in such situations. They did not want to be responsible for life and death decisions. Three doctors explained how they left the final decision to the parents, so as to avoid the sense of guilt associated with deciding on someone else's life.*<br>*Almost all the doctors at both hospitals mentioned the gender problem in India as a dilemma in their work. Several had experienced the conflict themselves. They had noted, for example, that parents found it easier to consent to the withdrawal of treatment, or that there was less interest in buying medicine, when the baby was a girl... Lack of understanding, lack of resources to take care of a daughter or other children, fear of high dowries, and the cultural stigma of having a girl were some of the factors mentioned.*<br>*Some reported that when they knew the baby would die anyway, they did not consider the withdrawal of treatment as an option...Babies in critical conditions provide learning opportunities, which doctors feel they can make use of so as to perform better next time or when the economy improves. This perspective was reported both by senior registrars and by postgraduate students.*<br>Relationships with others<br>Effects on family and friends: *In many cases of terminated treatment, the doctor had felt uneasy about continuing treatment for babies with uncertain outcomes, when they knew that the parents could not afford to provide lifelong care. Responsibility for a family's future was experienced as the worst dilemma when deciding whether or not to withdraw treatment. Where the doctors managed to save a*

Confidential: For Review Only

	premature or critically sick child, they were aware that its survival was likely to create problems for the parents, requiring money for medicine, hospital visits and care. They also considered the risk of incurring lifelong expenses for the parents if the child failed to marry because of a handicap. This would also affect other children in the family...<br>*Those doctors who were concerned about the future of children who survive talked about lack of opportunities with respect to the family rather than the child. Several doctors had experienced mothers at follow-up consultations complaining about the fact that the doctor had saved the child, as there was so much trouble with it. One doctor said that in such situations he could only comfort the mother, but he and others who reported similar experiences felt responsible, even though they had only been doing their jobs.*<br>*"Who am I, a third person, to decide? His brother and sister might not go to school because of this."*<br>*Some underlined that these decisions are different in India compared to Western countries, where guidelines are based on the assumption that the parents or society will be able to cope with the longterm consequences. Many of them found that their own guidelines did not take their local social and economic realities sufficiently into account.*<br>Survival<br>Survival without disability; *"India needs productive people; we are creating the disabled."*<br>*Others said they were aware of the burdens that might result for society by treating high-risk babies. None had withdrawn treatment on these grounds, yet it was a perspective that concerned them when deciding to continue treatment.*<br>Other<br>Healthcare resources: *Limited resources were a common problem for our informants. Part of the dilemma was not being able to offer the best treatment to all, regardless of how many patients there were at the unit. Another aspect was the frustration of not being able to follow the national guidelines or the recommendations in Western textbooks and journal articles. Some had had the experience of having to withdraw, or of helping to withdraw, ventilation from a child in order to give the ventilator to another child with a better prognosis. In these cases it was known that the first child would most probably die anyway, or would only survive with a severe handicap even if given the best treatment available.*<br>*No one mentioned over-treatment as a dilemma in Indian NICUs, although many had a strong sense of wasting resources when critically ill babies were given costly, and highly intensive, care. They experienced this situation as frustrating, and some pointed out that 50% of the unit's resources were used on the 1–2% who were most sick, and whom they hardly managed to save anyway.*<br>*Limited resources for treatment were also a consequence of the poor economy of parents. The doctors reported how they had to balance the need for expensive medication and equipment (which the parents must buy for their child) with their perception of the parents' economy. They wanted to spare the parents the humiliation of having to refuse the best treatment or of not being able to act on recommendations to go to a private hospital, options which would probably result in economic ruin or the starvation of other children at home. Many of the doctors described personally trying to help out in such cases, by looking for funding or giving the parents money or medicine. Some also reported persuading the better off parents to buy things other babies could also benefit from. Another dilemma mentioned was that some children were transferred from private hospitals because the parents could no longer pay for treatment there*
Miracle (2004)	Gastrointestinal<br>Breastfeeding: *"They told me how breast milk was good, helped the babies to grow and help fight infections. Maybe because I had a small baby, and I feel that it helps mature his digestive system. And he's so tiny and not supposed to be digesting food this way, so I feel it's probably that much easier on his system-what nature meant for him to eat."*<br>*"I'm glad I'm doing it. I feed good that I'm doing it. I feel good about myself. It makes me feel good about myself that I'm doing it for him."*<br>*"I didn't feel forced at all. They tell you all the facts about it, how great it is for the baby, how much healthier it is, and they let me know how much better it is for the baby than formula. So I'm happy they do that. No, I didn't feel forced. I needed that push in the right direction*

Confidential: For Review Only

	and just to see the outcome and to hear people say, "Wow, that is good,"and "Wow, look how good you did." To see all that and hear all that, it makes you feel good."<br>*"Also, they've been telling me babies cannot digest the formula like the breastmilk."*<br>Infection<br>Prevention of infections: *"The nurse was talking to me about how breast milk fights off infections"*<br>*"She told me of the fact that they were earlier and didn't have the capabilities of fighting off infections, and they needed that from the milk, from me."*<br>*"There is a mother [peer counselor] that brings her baby with her to Milk Club, and she said her baby didn't have any colds or ear infections that 1st year. That story helped me. It's a success story."*<br>*"The neonatologist came in and said that it was to their benefit to grow quicker, they could tolerate it more, their immune system-that's what is best for them. That's all she needed to say, it was decided by sundown."*<br>Healthcare workers-Communication<br>Developing a caring relationship: *"So it makes me feel comfortable, even with the doctors. I think it was Dr. Wallace, she said (while I was pumping), "How's it going?" And it makes me feel good that we talk to each other like family."*<br>Normality<br>Normality: *"And he's so tiny and not supposed to be digesting food this way, so I feel it's probably that much easier on his system-what nature meant for him to eat."*<br>Growth<br>Growth: *"The doctors were explaining to me that my breast milk would help her gain weight."*<br>*"The neonatologist came in and said that it was to their benefit to grow quicker,"*
Moyer (2010)	Healthcare utilisation<br>Inappropriate treatments: *Community providers (including PCPs, home health nurses, pharmacists, and community emergency rooms) may lack the required knowledge and skills to manage complex infants, leading to suboptimal office-based care and perceived overutilisation of the emergency system.*<br>Healthcare workers-Communication<br>Communication about discharge: *Parents/care givers may be inadequately prepared for home care and management of fragile neonates due to a lack of consistent and early communication between parents and NICU staff and lack of coordinated educational and social services support programmes prior to discharge.*<br>Healthcare workers-Knowledge and Competence<br>Lack of consistency: *Healthcare providers (attending physicians, consulting physicians and other healthcare providers) in the NICU tend to act in isolation, which results in lack of a standardised, coordinated and comprehensive plan of care. Community providers (including PCPs, home health nurses, pharmacists, and community emergency rooms) may lack the required knowledge and skills to manage complex infants, leading to suboptimal office-based care and perceived overutilisation of the emergency system.*<br>Healthcare professional competence: *Community providers (including PCPs, home health nurses, pharmacists, and community emergency rooms) may lack the required knowledge and skills to manage complex infants,*
Orfali (2004)	Gastrointestinal<br>Oral feeding: *"I think his quality of life will be poor; he can't even eat. He will be profoundly retarded"*<br>Neurological

Confidential: For Review Only

	Significant IVH: *''Although she has a grade IV bleed, the resident says that she moves and looks around, and he thinks the odds are quite good.''*<br>*"The neurological outcome is never known (when the baby has a bleed). Ethically, if the baby is severely compromised, what do you do?I've talked to people who have seen kids with grade IV bleeds who come out fine. It makes it hard to make the decision"*<br>*"She has an IVH grade IV, but she moves quite well and things might turn up better then expected"*<br>Development-Special senses<br>Hearing impairment: *"This kid is in really bad condition. I told the parents that he will never be able to see, to hear and I will get more data to show them how bad things are. Because in this particular case, there is no doubt!"*<br>Visual impairment: *"This kid is in really bad condition. I told the parents that he will never be able to see, to hear and I will get more data to show them how bad things are. Because in this particular case, there is no doubt!"*<br>Healthcare workers-Knowledge and Competence<br>Ethical decision making: *Medical authority was exercised in limiting available options. As an American pediatrician commented: "I think they [parents] have played a part in dealing with the decision as much as we [doctors] have played a part in fixing them.''*<br>Survival<br>Survival with disability: *"I think his quality of life will be poor; he can't even eat. He will be profoundly retarded"*<br>*"At 2 cm, we know that the outcome is very dismal, that the kid will be severely handicapped"*<br>Survival without disability: *"I have always thought we should limit treatment instead of giving (the parents) a handicapped child. Especially as we know very well what happens on the long run for a couple. Statistically, there are many divorces and things like that"*
Rasmussen<br><br>(2016)	Neurological<br>Brain injury (not further specified):*''Brain injury is laden with a lot more emotions and moral concerns for sure . . . neurologic or neurodevelopmental impairment in the long term is much more associated with a negative outcome compared to compromise of another organ that can be supported with medical therapies in the long term.''*<br>Healthcare workers-Communication<br>Communicating in challenging settings:*"I see prognosis affecting families medical decisions . . . they do their best. I mean, it's not easy but they do their best to factor in their values and their lives. And they can't really speak for this individual who's never spoken to them before. But I think they do their best. And it's hard. It's a hard situation to be in."*<br>Other<br>Overall health state: *"By neurological prognosis I mean that we try to think of the whole life consequence. We talk about, you know, strength and cognitive capacity, but also life and communication and feeing yourself and getting around."*
Reyna (2006)	Gastrointestinal<br>Choking during feeding *"the only concern I have is, I don't want them to choke. I'm fearful of choking."* Feeding difficulties: *The first theme was interpreting infant behaviors (Table 2). This theme included recognizing infant feeding readiness, hunger, and satiation cues. Feeding readiness included mothers' consideration of their infants' motor and behavior state organization before a feeding, particularly the infants' level of alertness. Hunger cues were the behaviors mothers used to describe hunger in their infants. Similarly, satiation cues were cues recognized by mothers to indicate the infants had fed "enough" and was satisfied.*<br>Feeding practices: *The second theme was managing the evolving feeding process (Table 2). This involved the mother's evaluation of her infant's skill at bottle feeding, meeting the demands of the feeding schedule, and controlling her time and resources. This theme included the mother's perception of how successful she was at balancing her infant's frequent feedings with everyday demands such as*

Confidential: For Review Only

	grocery shopping, doing laundry, and caring for other children. In addition, a mother's previous experience with bottle feeding and how she used the support of other family members to manage this process was part of this theme.<br>*Realizing knowledge gaps was the third theme (Table 2). It involved understanding the nutritional needs of their infants, such as when to advance the feeding volume and when to omit a feeding. It included mothers' feelings of how prepared they felt at discharge and the reality of caring for the infant at home. Another a part of this theme was what the mother had learned from her initial experiences and how she managed anxiety about caring for her infant after discharge.*<br>*Before discharge, all infants in this study were on scheduled feedings with a prescribed feeding volume. Routine discharge instructions for the mother included advancing the infant's feeds as tolerated to an "ad libitum" schedule. Mothers had difficulty understanding these instructions and seemed hesitant to liberalize their infant's intake after discharge. They worried both about giving too much formula at a feeding or about missing a feeding...Despite concerns regarding feeding volume, 26% (8) of the infants were noted to have "spitting through their nose." This typically occurred as the feeding volume was advanced. None of the mothers recognized this as a problem or associated it with overfeeding or difficulty with suck-swallow-breathe coordination.'*<br>Parental Support<br>Parental competence: *The second theme was managing the evolving feeding process (Table 2). This involved the mother's evaluation of her infant's skill at bottle feeding, meeting the demands of the feeding schedule, and controlling her time and resources. This theme included the mother's perception of how successful she was at balancing her infant's frequent feedings with everyday demands such as grocery shopping, doing laundry, and caring for other children. In addition, a mother's previous experience with bottle feeding and how she used the support of other family members to manage this process was part of this theme*<br>*All of the mothers identified having the support of a husband, a significant other, or a family member(s); however, the mothers were the infants' primary care-givers. Mothers reported that family members were uncomfortable feeding their infants. Furthermore, mothers also reported that they were uncomfortable having other people feed their infants.*<br>Healthcare workers-Communication<br>Communicating information effectively: *The information provided in the NICU regarding feedings and well-baby care was considered appropriate and helpful. Mothers who were able to visit frequently while their infants were hospitalized had many opportunities to feed their infants before discharge and verbalized more comfort with the feeding process once home.*
Rohsiswatmo<br><br>(2014)	Infection<br>Sepsis: *Decrease blood stream infections Selected as key performance indicator*<br>Healthcare workers-Knowledge and Competence<br>Healthcare professional competence: *Processes thought to improve infection rates in NICU neonates: 'Education-socialization of employees; competence test; placement of employees according to competence; employee career path; renumeration'*<br>Survival<br>Survival: *Decrease mortality rate selected as key performance indicator*<br>Other<br>Duration of admission: *Decrease length of stay selected as key performance indicator*
Saigal (1999)	Development-Gross motor<br>Ability to walk: *Needs assistance to walk (from equipment/person)*<br>Development-Fine motor<br>Difficulties with activities of daily living: *Needs assistance/to use special equipment to eat, dress/bathe or use the toilet*

Confidential: For Review Only

	Development-Special senses<br>Hearing impairment: *deaf,*<br>Visual impairment: *Blind*<br>Development-Speech and Social<br>Language disorders: *Unable to talk*<br>Psychiatric<br>Mood disorders: *Occasionally fretful, angry, irritable, anxious, depressed, or experiencing 'night terrors'*<br>Need for educational support: *Learns schoolwork very slowly and needs special help*<br>Pain<br>Pain: *Sometimes has pain*<br>Normality<br>Normality: *Happy and not worried most of the time*<br>Other<br>Overall health state: *It appears that HPs* [Healthcare Professional] *and parents view the mild to moderately disabled health states similarly, but parents were more accepting of the severely disabled health states than HPs.*
Saunders<br><br>(2003)	Gastrointestinal<br>Breastfeeding: *Benchmarking questions identified: how do you encourage breastfeeding on your unit?*<br>Parental Support<br>Parental involvement: *Benchmarking questions identified: Does your unit welcome parents 24 hours/day? How are families encouraged and supported to participate in routine care of the infant? What provisions for privacy around the bedside do you offer? Are families encouraged to make their infants immediate environment as homelike as possible?*<br>Healthcare workers-Communication<br>Communication about discharge: *Benchmarking questions identified: Does your hospital provide teaching support for outside personnel? How ready do you feel you are to care for your infant after discharge from the hospital?*<br>Pain<br>Pain management: *Benchmarking questions identified: What tools are used for pain assessment?*<br>Suffering<br>Suffering: *Benchmarking questions identified; Are ventilated infants routinely sedated?*<br>Normality<br>Normality: *Benchmarking questions identified: During your infant's hospital stay, how often did you feel like a parent? Are families encouraged to make their infants immediate environment as homelike as possible?*
Sawyer (2014)	Healthcare workers-Communication<br>Communicating information effectively: *I was given all the information I needed Encouragement and reassurance,*<br>Healthcare workers-Knowledge and Competence<br>Healthcare professionals behaviour: *Staff professionalism and empathy The staff put me at ease The staff made me feel cared for as an individual*<br>*Staff being confident and in control Staff being calm in a crisis*

Confidential: For Review Only

Schneiderman (1997)	Suffering<br>Suffering: *Providing futile life-sustaining treatments "ridiculous," "dumb," "inappropriate," "insane," "outrageous," "frightening," "a teaching example of futility," "an example of what's wrong with medicine today,"*
Sharek (2003)	Respiratory<br>Chronic Lung Disease: *Outcomes identified by CLD focus group: Chronic Lung Disease rate Oxygen requirement at discharge home Pneumothorax rate*<br>Oxygen dependence: *Outcomes identified by CLD focus group: Chronic Lung Disease rate Oxygen requirement at discharge home Pneumothorax rate*<br>Pneumothorax: *Outcomes identified by CLD focus group: Chronic Lung Disease rate Oxygen requirement at discharge home Pneumothorax rate*<br>Development-Special senses<br>Retinopathy of Prematurity: *Outcomes identified by CLD focus group: ROP rates*
Short (2003)	Development-Gross motor<br>Need for physical therapy: *significantly more BPD children received...occupational, and physical therapy*<br>Development-Speech and Social<br>Language disorders: *significantly more BPD children received speech-language therapy*<br>Parental Support<br>Support from healthcare professionals: *Parents of children with a history of BPD reported that more of their children had occupational and physical therapy, including children who were without neurologic complications. The prolonged hospitalizations experienced by children with BPD and the frequent interactions of families with medical personnel may result in increased access and opportunities for services for parents of children with BPD.*<br>Healthcare utilisation<br>Frequent readmissions: *Parents of children with a history of BPD reported that more of their children had occupational and physical therapy, including children who were without neurologic complications. The prolonged hospitalizations experienced by children with BPD and the frequent interactions of families with medical personnel may result in increased access and opportunities for services for parents of children with BPD.*<br>Psychiatric<br>Need for educational support; *BPD children were more likely to be enrolled in lower grades than VLBW or term children. lacement in special education ser-vices, regardless of type, differentiated the groups, with 54% of the BPD, 37% of the VLBW, and 25% of the term children participating in special education.*
Sisk (2010)	Gastrointestinal<br>Choice of milk for feeding: *Universally, mothers perceived the pumping schedule as a huge weight on their shoulders because of the time it required. Mothers often spoke of the conflict between taking care of themselves and their other responsibilities and the need to pump...Mothers often delayed pumping to be at their infant's bedside for medical rounds, and it was unpredictable when those rounds would occur. Pumping at the bedside was an option; however, many mothers or their spouses were uncomfortable with this.*<br>*Attitudes toward milk expression influenced mothers' willingness to pump.*<br>*Anticipation of breastfeeding provided support for milk expression. Mothers who planned to breastfeed as opposed to exclusively bottle*

Confidential: For Review Only

	feed their breast milk viewed pumping as something they had to do.<br>Parental Support<br>Support from fathers: *Fathers ranged from being very supportive, "[we're] in this together," to being deterrents or completely absent.*<br>Parental ability to work: *Seven mothers returned to work within 3 weeks after delivery; none had paid maternity leave. They liked being back at work because it helped occupy their minds, but they reported being exhausted*<br>Coping with maternal illness: *Physical and mental challenges. The majority (59%) of mothers experienced a pregnancy-related medical complication, and almost half (44%) were treated with magnesium sulfate, a medication given to treat preterm labor and prevent seizures in severe preeclampsia. Mothers who received this medication stated that they had difficulty comprehending the pumping instructions or did not feel well enough to pump....*<br>*Mothers, especially those who were delivered by cesarean section, complained of extreme fatigue and desire for sleep in the first few days after delivery, and this interfered with pumping frequency and their ability to remember the pumping instructions*<br>*...Feeling anxious and stressed were common complaints during the immediate postpartum period. Lack of preparation for the preterm delivery and disruption of plans as well as concern for their infant's health, job, and finances distracted mothers from pumping frequently. For some mothers, however, anxiety regarding their infants' health motivated them to frequently pump.*<br>Barriers to parental involvement: *Lack of privacy was identified as a barrier to establishing a pumping schedule. Frequent interruptions and the flow of hospital staff, family, and friends interfered with their ability to learn to use the breast pump, relax during pumping, and pump frequently. Mothers disliked telling their visitors to leave, and they disliked pumping in front of them. Mothers stated that hospital staff members often were unable or unwilling to provide the amount of privacy they desired.*<br>*-availability of hospital grade breast pump helped transition from BF in hosp to BF at home*<br>*-pumping at home without stimulus of infant was more difficult*<br>*-transport to hospital to feed infants difficult*<br>Support from family and friends: *All mothers identified supportive people in their social networks. These included family members, friends, church members, and hospital staff. Practical assistance with chores (ie, child care for older children, meal preparation, and house cleaning) was critical to their success. The recognition and praise mothers received also motivated them to continue pumping.*<br>*-mothers relied on rides to hospital from family and friends, which sometimes worked, sometimes didn't in terms of feeding*<br>Balancing caring for themselves and their baby: *Universally, mothers perceived the pumping schedule as a huge weight on their shoulders because of the time it required. Mothers often spoke of the conflict between taking care of themselves and their other responsibilities and the need to pump...Mothers often delayed pumping to be at their infant's bedside for medical rounds, and it was unpredictable when those rounds would occur. Pumping at the bedside was an option; however, many mothers or their spouses were uncomfortable with this.*<br>*Anticipation of breastfeeding provided support for milk expression. Mothers who planned to breastfeed as opposed to exclusively bottle feed their breast milk viewed pumping as something they had to do*
Smith (2006)	Gastrointestinal<br>Breastfeeding: *"Because I think I understood the importance of what my wife was trying to do. It was extremely important for our babies, who are very fragile and sick, to receive breast milk."*<br>Parental Support<br>Support from fathers; *"I helped her as much as I could. I cleaned and carried the pump when necessary. I cleaned the pump kit when she was finished pumping, labelled the bottles, cleaned the bottles, transported the milk to the hospital, assisted with storage, and assisted her in whatever way I could while she was pumping."*

Snowdon<br><br>(1997)	Parental Support<br>Support from faith: *"We regard Philippa as a gift from God and so when she was ill, you know we prayed that--we obviously prayed that we wanted to keep her but we knew that God would do what was best for her and for us and so I think although it was very worrying and very traumatic, there was a sort of peace underneath that actually"*<br>Healthcare workers-Communication<br>Communicating in challenging settings: *"Nobody can play God. You're making a decision on somebody's life and they don't really know so the computer decides and that's the best way because you're not blaming anybody then are you? You don't really want the onus on yourself really but I suppose the medical profession can't.., keep the onus on themselves"*<br>Survival<br>Survival: *" I think any parent wants to try anything, you know, they don't sit down and think it's research, there could be side effects or there could be abnormalities that might come up. I think as parents...your first instinct is to save your baby"*<br>*"I suppose what they're saying is that er if at the end of the day conventional really really really isn't going to work for him then that would be absolutely ideal because.., the chances are maybe he would be picked"*<br>*"I suppose trials have to be a bit heartless, but you'd think that when the baby looks like they're dying, you'd think they'd just say... "Oh hell" you know "'let's try the ECMO, see if it saves this baby" but with that sort of a trial they can't do that can they? They have to say, "Well look, this baby looks like it's dying but I'm sorry it's getting conventional treatment and that"*<br>*"your first instinct is to save your baby"*<br>*"we obviously prayed that we wanted to keep her "*<br>Other<br>Inclusion in research: *"I feel desperately sorry for parents who, you know, were turned down particularly if their child doesn't live. I think it would be hard but I can see that it is necessary in case...the research shows that ECMO is actually detrimental to children"*<br>Iatrogenic harm: *"I suppose trials have to be a bit heartless, but you'd think that when the baby looks like they're dying, you'd think they'd just say... "Oh hell" you know "'let's try the ECMO, see if it saves this baby" but with that sort of a trial they can't do that can they? They have to say, "Well look, this baby looks like it's dying but I'm sorry it's getting conventional treatment and that"*
Snowdon<br><br>(2014)	Parental Support<br>Coping with maternal illness: *All women who had a CS, some of whom needed additional interventions, such as Laura, who underwent surgical removal of her placenta, and Caitlin, who had reparative bowel surgery after damage caused during delivery, had to deal with the aftermath of their birth and surgery and their initial experiences in the NICU at the same time...Where women were debilitated postnatally this could limit their contact with their babies. Although a number were taken to the NICU in a wheelchair or their bed, when babies were moved to another hospital it was not always possible for the women to accompany them.*<br>*The mode and pace of delivery also impacted upon the men. Some of the men did not arrive at the hospital in time for the birth, as events could be unexpected and fast moving. When women underwent an emergency CS, men could be excluded from the birth, a potentially isolating and anxiety-provoking experience.*<br>Parental involvement: *Once babies were delivered and taken to the NICU, there was usually a lag before parents were able to visit. Some of the parents were frustrated at not being allowed to go to the NICU with their baby and the wait for news or to be allowed to visit could seem interminable.*<br>Healthcare workers-Communication<br>Communicating in challenging settings: *When they arrived at hospital in preterm labour, or were admitted for observation and bed rest, some had difficult conversations with clinical staff about the implications of their gestational stage for decisions about care. NICUs*

Confidential: For Review Only

	commonly set boundaries around the care that they offer, as their facilities determine their lower thresholds for care...Parents who were around the 23-/24-week cusp not only had to contend with the worry of whether or not their babies would survive and in what condition, but also they were faced with the possibility that intensive care would not be mobilised in their case. At the borderline of viability they had to wait to see whether their baby attempted to breathe to find out whether or not care would be initiated. This sense of ambiguity could be heightened when women were given antenatal steroids to help to mature their babies' lungs and improve their chances of survival, while also being faced with the possibility of no active care being offered postnatally.<br>Keeping parents informed: *Once babies were delivered and taken to the NICU, there was usually a lag before parents were able to visit. Some of the parents were frustrated at not being allowed to go to the NICU with their baby and the wait for news or to be allowed to visit could seem interminable.'*<br>Survival<br>Survival: *this trial spoke directly to their most pressing concerns; the threat to survival and the possibility of disability*<br>*The overwhelming sense from the parents interviewed for BRACELET was that at the time they wanted their babies to be cared for and for everything possible to be done. A minority of the parents who were facing extremely preterm birth came to their own decisions about care, which pre-empted NICU policy on admission.*<br>Survival without disability: *this trial spoke directly to their most pressing concerns; the threat to survival and the possibility of disability*<br>Other<br>Inclusion in research: *Parents interest in a trial appeared to develop over time as their bereavement receded. Although most had experienced little contact with a trial over the years, parents were often interested in the research, and some would have liked more contact and information than they actually received. Trial communications were valued as a source of information, as an acknowledgement of loss and the contribution to research, as a connection back to their baby and a form of commemoration. Some parents had kept trial paperwork in their baby's memory box.*<br>*Danielle's experiences of antenatal recruitment to BOOST-II UK were strikingly similar to Beverley's but they described different positions on altruism and enrolment. For Danielle her sense of altruism and the potential benefits for others was sustained throughout her association with the trial, but for Beverley it was more transient and dissipated somewhat once she was able to engage with the reality of her daughter's initial survival, condition and needs.*<br>*Discussion of the trial therefore required emotionally strained parents and physically debilitated mothers to engage not only with the information and choice offered to them in relation to cooling and the trial, but also confronted them at an early stage in their experience with the possibility of brain damage and disability for their newborn. Parents still reeling from delivery had to think about both the immediate and the long-term future.*<br>*Clearly parents come to neonatal intensive care via a range of different clinical pathways. Their reactions to the environment and to their babies are similarly varied and highly personal, bringing together emotions from recent and sometimes more distant events. It is against this background of threat, initial survival and initiation of care that they made their decisions about trial participation.*<br>*Ivan mentioned INIS right in the middle of this difficult time, as a direct response to the threat of serious infection for their baby. This places the trial into a therapeutic framework, and parents spoke of their hopes that INIS would make a difference, but by this point the gravity of their situation was sometimes clear and the trial could seem like a long shot rather than a magic bullet.*<br>*The offer of the trial, however, was disassociated from the crisis that the parents had experienced, partly because of the flexibility over timing and partly because the intervention related to a routine management of care.*<br>*Three main models of the potential effect of a trial could be identified in the data (Figure 16), which were characterised in the analysis as: -might help, won't harm -might help, might harm -won't harm, might help others'*

Confidential: For Review Only

Squitieri (2013)	Development-Fine motor<br>Difficulties with activities of daily living: *“A lot of times I have to put myself in certain positions to do things, like opening a jar- I have to squeeze it in between my arms instead of gripping it with my hand. And when I type, I have to position my fingers in a different way on the keyboard so I can reach the keys.”*<br>*Most adolescents were extremely functional and able to accomplish basic activities of daily living due to the high degree of learned compensation. Many females reported difficulty grooming their hair*<br>Parental Support<br>Support from family and friends: *“My mother in law and my mother both would watch my older daughter that first year quite a bit while I would take my daughter to therapy”*<br>Healthcare utilisation<br>Frequent appointments: *Many parents commented on the time commitment of formal therapy visits. Depending on the ease of access to a nearby therapist, many adolescents would miss school and their parent would need to take off from work.*<br>Relationships with others<br>Peer acceptance: *Peer acceptance and fear of being teased or excluded from social groups was a prominent theme throughout our interviews. In addition, many adolescents commented on the importance and positive influence of obtaining support from other adolescents with NBPP through various camps, therapy groups, or online.*<br>Psychological coping: *Despite their impairments, most adolescents had a positive view of themselves overall. Negative coping mechanisms were more common among younger adolescents and adolescents with greater levels of impairment. Older adolescents generally dealt with their impairments in a constructive manner either through learned compensation techniques, acceptance, or anticipation and alteration of activities if they believed their impairment would limit them in any way.*<br>Family resources: *All participants in our study had some form of insurance coverage. However, some parents reported frustrations regarding the cumbersome process of talking with insurers to confirm coverage or extended therapy visits.*<br>Effects on family and friends: *Almost all parents acknowledged the emotional adjustment of other family members in response to raising a child with physical impairment. Many relied on the help of extended family. Few parents reported negative effects on parental/sibling relationships.*<br>Pain<br>Chronic pain: *Occasionally some patients or their parents reported hypersensitivity or mild discomfort with therapy exercises or prolonged activity. No patients reported significant pain that impaired daily living. “She occasionally complains of pain in her shoulder area. Maybe 1-2 times per month."*<br>Suffering<br>Comfort: *Patient priorities: To sleep more comfortably*<br>Normality<br>Normality: *“My arm used to make me mad and sad because I couldn’t be like everybody else… ‘why can’t I raise my hand like this?’ and stuff like that."*<br>Other<br>Physical appearance: *“I got teased in the fourth grade. They’d call me ‘overblown bicep’ and ‘fat arm.’ Sometimes I would get really self-conscious and discouraged and think that my arm looks weird and that people are staring at me.”*<br>*“I don’t like wearing half sleeves or quarter length sleeves because it looks like a long sleeve on my right (palsy) arm but a quarter sleeve on my left (non-palsy) arm.”*

Confidential: For Review Only

	Almost all adolescents and parents commented on aesthetics and body image regarding arm position, length/size discrepancies, and clothing restrictions.
Stevens (2014)	Gastrointestinal<br>Oral feeding: *"(The) very first time (feeding the baby) was just great, to tell you the truth. It was an experience, just like, 'Wow'…it was just looking into him and just like, 'wow, look what we made, 'know?' It was just crazy…it was just wonderful."*<br>Relationships with others<br>Bonding with parents: *"It was pretty awesome [laughing] because (his daughter) had been in an incubator for a while and we couldn't hold her and then we actually got to hold her. Holding a baby is something that I've always wanted so to me it was priceless"*<br>Normality<br>Normality: *"It was pretty awesome [laughing] because (his daughter) had been in an incubator for a while and we couldn't hold her and then we actually got to hold her. Holding a baby is something that I've always wanted so to me it was priceless"*<br>Growth<br>Growth: *"you want to be sure that they take everything so that they're gaining weight adequately"*
Thoren (2013)	Parental Support<br>Online support: *"This group is for parents who have experienced the miracle of having a preemie. Let's share our stories to help each other through the difficult times of NICU and support each other through the even more challenging task of raising a preemie."*
Thoyre (2000)	Gastrointestinal<br>Oral feeding: *"Just get the bottle in him. That's all. "*
Turrill (2003)	
Turrill (2003)	Neurological<br>Significant IVH: *"By then.. em.. I think we're coming off the big risk of nasty big haemorrhages as well, unless something seriously goes wrong with them for some obscure reason."*<br>Development-Special senses<br>Visual impairment: *Children needing to wear hearing aids or glasses*<br>Hearing impairment: *Children needing to wear hearing aids or glasses*<br>Parental Support<br>Parental involvement: *Reduced parental contact was cited as a characteristic of increasing risk for alterations to development...Support of the family unit in hospital is essential to the future wellbeing of that family. However what that part of care has to play in the long term development outcomes of newborns who receive intensive care is unknown. Although parental support is a vital aspect of their role, relating this to developmental outcomes is misguided and again, highlights the lack of a common knowledge base supporting practice.*<br>Healthcare workers-Communication<br>Communication with parents: *"When you're talking to parents while you're doing cares and everything, you're not really talking to them,... you're having a vague conversation across the room"*<br>*Communication between professionals p.33 'Beyond the immediate admission, relationships betwen nurses and doctors and their differing roles and responsibilities appeared at times in conflict, with little team approach to care.'*<br>Healthcare workers-Knowledge and Competence<br>Healthcare professional competence: *nurses' lack of knowledge of short and long term LBW infant outcomes: lack of evidence-based*

Confidential: For Review Only

	guidelines to support nurses use; too lengthy; info not seen as important or relevant to their role; lack of time; no formal channels to disseminate information<br>*nurses lack understanding that 80% of LBW infants develop normally, and what 'normally' is, means that they don't feel able to foster that normality in NICU care*<br>*'The physical stability of babies must remain the nurses first priority when considering optimal brain function in relation to future outcomes. However, the importance of controlling the neonatal environment to increase stability appeared to have been lost. The constant intensiveness of the working environment, while recognised as not normal, has been taken for granted.*<br>*The quality of physical care given to a VLBW baby in the first 24 hours is vital to his or her survival and future development. The responses from nurses in clinical and managerial positions showed they were acutely aware of this priority...four [higher grade] nurses had changed priorities [in the scenario], with parents gaining importance over time.*<br>*Nurses recognized that their limited knowledge, in-service education and research were seen to affect their decision-making abilities, confidence and consequent autonomy. The current strategy for continuing education is affected by the fact that nurses are rarely able to leave the cot-side to take part in meetings, seminars or reflective groups due to the pressure of clinical commitments. Also access to a clinical academic neonatal pathway is not currently available within the unit's education contracts'*<br>Consistency of decisions: *Beyond the immediate admission, relationships between nurses and doctors and their differing roles and responsibilities appeared at times in conflict, with little team approach to care.*<br>Normality<br>Normality: *nurses lack understanding that 80% of LBW infants develop normally, and what 'normally' is, means that they don't feel able to foster that normality in NICU care*<br>*With the emphasis on normal development being lacking in their knowledge base, the effects of interventions appear weighted towards a failure to achieve the ideal at the start. The level of responsibility the nurses felt they held towards the baby's future, coupled with this high risk of failure, posed an unrealistic challenge.*<br>Growth<br>Growth: *nutrition relates to growth and thriving*<br>Other<br>Healthcare resources: *The level of resources was thought to have the greatest impact on the nurses' ability to carry out their role, in particular the nursing resource.*<br>*'When considering what aspects of service impacted on their ability to carry out the care they ideally wanted to give, limited available time was important. With a workforce of only 62% of the regionally-funded QIS level, the constant pressure had a great impact on managing priorities for care.*
van Zuuren<br><br>(2006)	Development-Gross motor<br>Issues of development and motor skills: *"will it stay dependent on the care of oth-ers for the rest of his life, will it remain spastic, will it be wheelchair-bound?"*<br>Development-Speech and Social<br>Language disorders: *"Will the child be able to communicate in the future"*<br>Healthcare workers-Communication<br>Communicating in challenging settings: *Uncertainty, in particular unpredictability of the outcome in the child, is seen as a complicating factor in end-of-life decisions, and, to a lesser extent, in the problem of medical damage.... Long-term follow up is advocated in order to reduce this uncertainty.*

Confidential: For Review Only

	Suffering<br>Suffering: *It was the nurses who most explicitly pointed to the direct suffering that treatment devices often impose on the child and who sometimes resented the parents' or doctor's wish to prolong treatment. For these nurses, there is often an imbalance between two important ethical principles: doing well and avoiding harm....So, clear disadvantages of doing well (treatment) are the immediate suffering of the newborn and the possible damage in the future.*<br>Other<br>Survival with disability: *"The greatest dilemma has to do with the handicaps we induce with our treatment." "A child with mild handicaps can have a good life. A child that hardly makes any developmental progress and that will be dependent on others all along .... (unfinished)."*
Vandenbussche<br><br>(1999)	Neurological<br>Neurological symptoms *-Mothers' and obstetricians' valuing of 4 types of infant outcome: -healthy infant -transient neurologic symptoms -permanent neurologic symptoms -neonatal death*<br>Survival<br>Survival: *Pregnant women and mothers valued an infant with permanent handicap significantly higher than neonatal death, and this choice was irrespective of the type of birth (all p values < 0.01). Obstetricians, on the other hand, awarded lower, albeit not statistically different median values to permanent handicap than to death.*<br>Survival with disability: *Pregnant women and mothers valued an infant with permanent handicap significantly higher than neonatal death, and this choice was irrespective of the type of birth (all p values < 0.01). Obstetricians, on the other hand, awarded lower, albeit not statistically different median values to permanent handicap than to death.*
Votteler (2005)	Genitourinary<br>Urological disorders: *Persistent urological problems concern twin A's mother as to whether he will be accepted or teased by his peers when he is older.*<br>Surgical<br>Need for multiple operations: *Three twins required scoliosis correction in later child-hood. Neurosurgical problems have been significant in both 1988 pygopagus sets, requiring long-term evaluation.*<br>Development-Gross motor<br>Ability to undertake sport: *They report feeling self-conscious of their appearance and avoid athletic activity, which calls attention to their physique. She cannot participate in physical education because of orthopedic and gait problems.*<br>Development-Special senses<br>Hearing impairment: *Social communication is difficult because of his hearing and speech problems, and he is described as having a few friends and no experience in dating.*<br>Development-Speech and Social<br>Language disorders: *Social communication is difficult because of his hearing and speech problems, and he is described as having a few friends and no experience in dating.*<br>Social difficulties: *Social communication is difficult because of his hearing and speech problems, and he is described as having a few friends and no experience in dating.*<br>Healthcare utilisation<br>Need for frequent treatments: *Three twins required scoliosis correction in later child-hood. Neurosurgical problems have been significant*

Confidential: For Review Only

	in both 1988 pygopagus sets, requiring long-term evaluation.<br>Psychiatric<br>Need for educational support: *The patient is at an age-appropriate grade level but attends resource classes in math and achieves only average grades in other areas. The girls are at an age-appropriate grade level and earn mostly As with some Bs. Both aspire to pursue college and graduate school education.*<br>Psychiatric disorder: *The other boy has been diagnosed with autism and bipolar disorder and is receiving care in psychiatry, neurology, occupational therapy, and speech. The mother is very focused on the boys' physical and emotional symptoms*<br>Normality<br>Normality: *Her parents consistently emphasized her normalcy and need to gain independence. The parents had a second child to 'normalize' life for their surviving son.*<br>Survival<br>Survival: *His mother reports a continuing sense of sadness over the neonatal death of the patient's twin. A recent first-time exposure to a photograph of this deceased infant was described as extremely meaningful and comforting. His parents report significant sorrow over the loss of twin B and the mother eventually went through counseling to work through these grief issues. Twin A's young parents are still grieving the death of twin B and are still questioning their decision making in terms of that twin's care.*<br>Other<br>Physical appearance: *They report feeling self-conscious of their appearance and avoid athletic activity, which calls attention to their physique.*
Wielenga<br><br>(2015)	Respiratory<br>Mechanical ventilation: *Research priorities identified: ...(6) respiratory and ventilation;*<br>Gastrointestinal<br>Feeding practices: *Research priorities identified: Identifying best practices in enteral feeding Identifying best practices breast feeding Improving nutrition in preterm and sick term infants*<br>Neurological<br>Neurological care: *Research priorities identified: Identifying the best care practices for infants with neurological problems*<br>Infection<br>Sepsis: *Research priorities identified: Evaluating infection prevention strategies Identifying and evaluating interventions to monitor and reduce hospital-associated infections Evaluating sepsis management and care to improve outcomes*<br>Skin<br>Skin care: *Research priorities identified: Identifying and evaluating interventions to improve skin and wound care in neonates*<br>Surgical<br>Care for surgical babies: *Research priorities identified: Identifying the best care practices for surgical infants*<br>Parental Support<br>Parental involvement: *Research priorities identified: Identifying strategies to support the needs of parents and family members Identifying and evaluating strategies to support parental attachment Identifying best practices for the implementation of family centred care Evaluating the role and involvement of parents in the care of their infant Improving end-of-life care for neonates and their families Exploring the role of parents in ethical decision making*<br>Healthcare workers-Knowledge and Competence<br>Healthcare professionals behaviour: *Research priorities identified: (3) clinical nursing care practices; (4) quality and safety; (5) ethics; and*

Confidential: For Review Only

	(8) professional issues in neonatal intensive care nursing.<br>Pain<br>Pain management: *Research priorities identified: Identifying effective interventions to prevent or reduce pain or stress Identifying best practices for pain assessment Identifying pain and/or stress guidelines*

Confidential: For Review Only

eFigure 2: Results Of Permutation Test Analysis Regarding Gestational Age

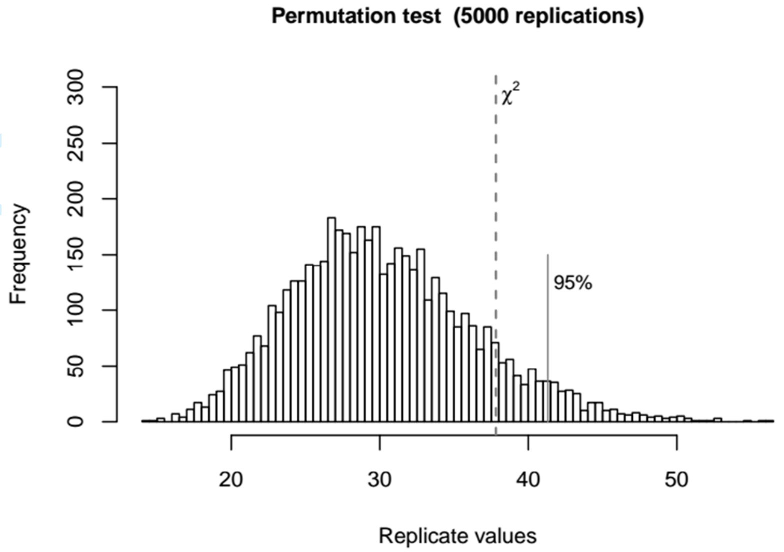

Permutation test analysis for association of gestational age of babies within a study and outcome categories identified within study. The diagram presents the null-distribution of the chi-square (goodness-of-fit) statistic obtained by simulations. The X axis describes the "replicate value" assigned to each replicate while the Y axis describes the frequency with which each replicate value arose during the 5000 replications. The distribution does not have the chi-square shape because some articles/papers contribute with more than one entry in the contingency table of parties-by-topics. The vertical dashes (χ^2) mark the value of the statistic (evaluated on the observed dataset) and the vertical solid line (95%) is the critical value, where the distribution has its 95th percentile. The value of the statistic is 37.82, the critical value is 41.31 (p-value=0.114).

For peer review only

eFigure 3: Results Of Permutation Test Analysis Regarding Stakeholder Group

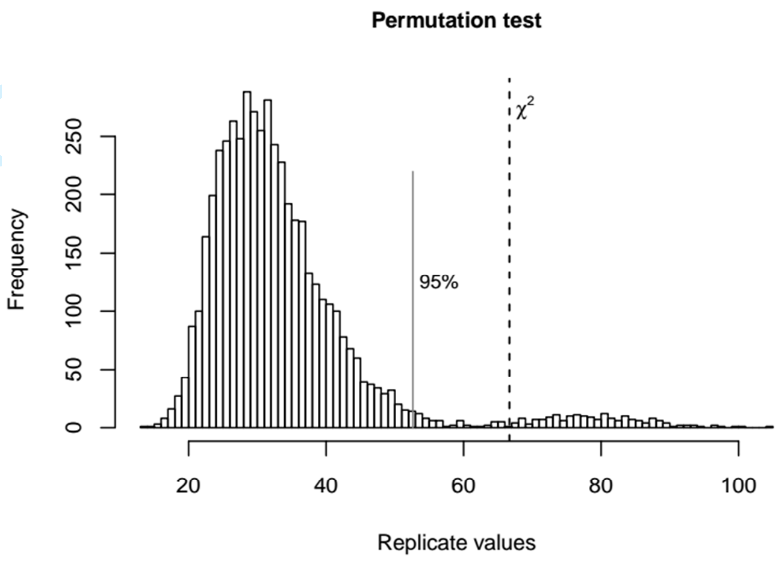

Permutation test analysis for association of stakeholder groups within a study and outcome categories identified within study. The diagram presents the null-distribution of the chi-square statistic obtained by simulations. The X axis describes the “replicate value” assigned to each replicate while the Y axis describes the frequency with which each replicate value arose during the 5000 replications. The distribution does not have the chi-square shape because some articles/papers contribute with more than one entry in the contingency table of parties-by-topics. The vertical dashes (χ^2) mark the value of the statistic (evaluated on the observed dataset) and the vertical solid line (95%) is the critical value, where the distribution has its 95th percentile. The value of the statistic is 66.67, the critical values is 52.62 (p-value=0.037).

For peer review only
